# Bifunctional Imine Reductase Cascades for the Synthesis of Saturated *N*-Heterocycles

**DOI:** 10.1021/acscatal.4c03832

**Published:** 2024-09-19

**Authors:** Jeremy I. Ramsden, Bruna Zucoloto da Costa, Rachel S. Heath, James R. Marshall, Sasha R. Derrington, Juan Mangas-Sanchez, Sarah L. Montgomery, Keith R. Mulholland, Sebastian C. Cosgrove, Nicholas J. Turner

**Affiliations:** † Manchester Institute of Biotechnology, Department of Chemistry, University of Manchester, 131 Princess Street, Manchester M1 7DN, U.K.; § Chemical Development, Pharmaceutical Technology and Development, Operations, AstraZeneca, Silk Road Business Park, Macclesfield SK10 2NA, U.K.; # School of Chemical and Physical Sciences and Keele Centre for Glycoscience, Keele University, Keele ST5 5BG, U.K.

**Keywords:** imine reductase, reductive aminase, cyclization, N-heterocycles

## Abstract

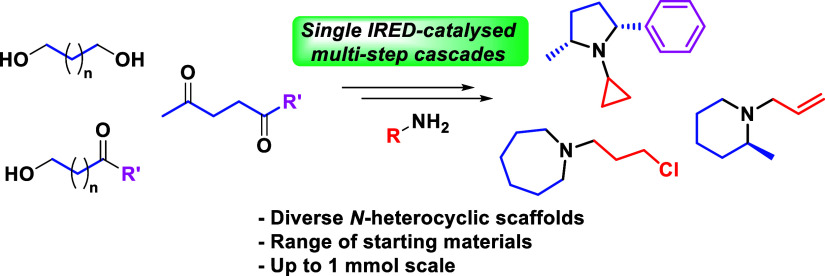

Saturated *N*-heterocycles constitute a vital scaffold for pharmaceutical chemistry but are challenging to access synthetically, particularly asymmetrically. Here, we demonstrate how imine reductases can achieve annulation through tandem inter- and intramolecular reductive amination processes. Imine reductases were used in combination with further enzymes to access unsubstituted, α-substituted, and α,α′-disubstituted *N*-heterocycles from simple starting materials in one pot and under benign conditions. This work shows the remarkable flexibility of these enzymes to have broad activity against numerous substrates derived from singlular starting materials.

## Introduction

**Figure 1:**
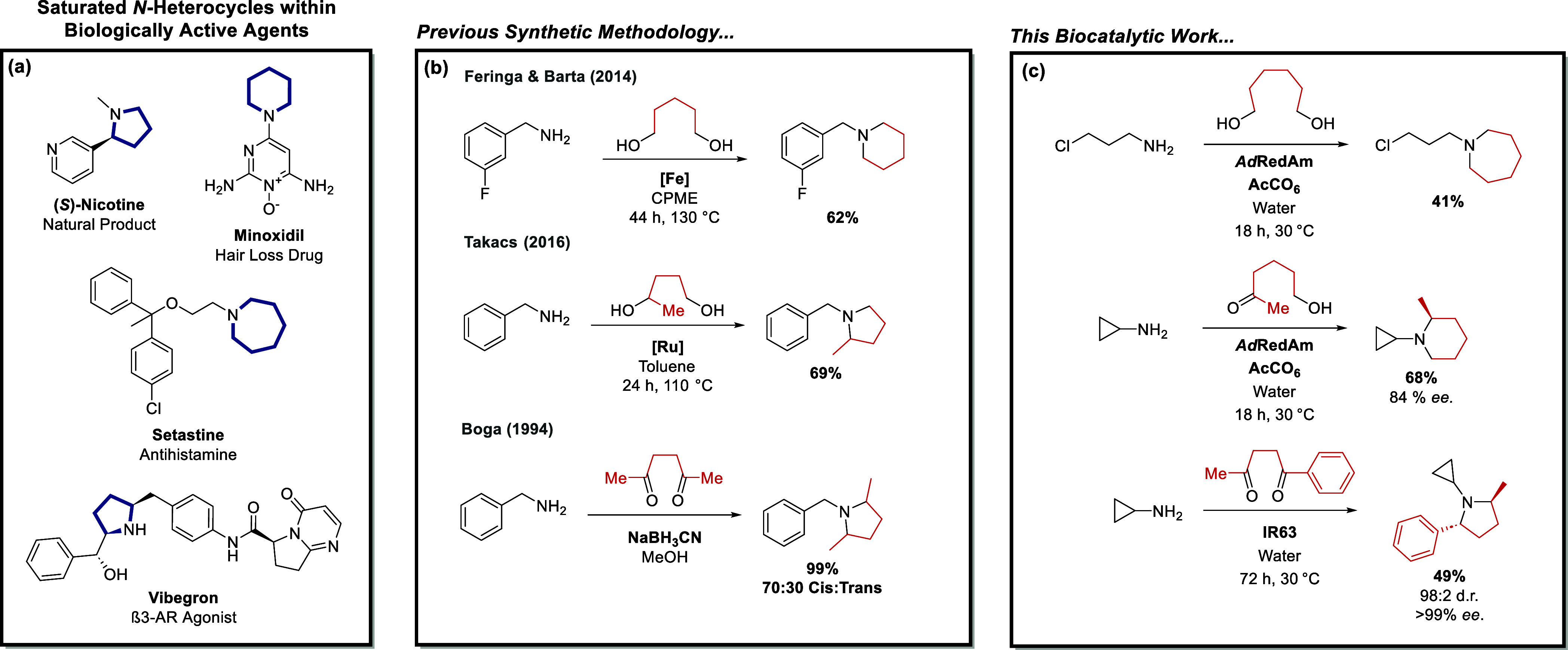
Biologically active molecules containing saturated *N*-heterocycles (a) and a comparison between previous chemocatalytic and chemical annulation methodology (b) and the work outlined in this article (c).

With half of all FDA-approved pharmaceuticals containing saturated *N*-heterocycles, improved methods for their synthesis are essential (Figure 1a).^1^ Current approaches, with limitations around stereochemical control through to sp^3^ C–H functionalization, limit access to diverse scaffolds. One specific challenge is *N*-alkylation, with methods such as reductive amination and nucleophilic substitution resulting in overalkylation and poor atom economy while depending on unsustainable reagents.^2,3^ Recently, borrowing hydrogen catalysis has seen application in the synthesis of saturated *N*-heterocycles through *N*-alkylation of amines with terminal diols; however, amine coupling partners have typically been limited to benzylamine and aniline derivatives.^4−6^ To access α-substituted *N*-heterocycles, diol substrates containing a secondary alcohol would permit retention or generation of a stereocenter. Although this has been demonstrated asymmetrically, the scope remains limited to few examples employing benzylamines, with reports proceeding with poor chemoselectivity.^7,8^ While intramolecular hydroamination offers an alternative route to these scaffolds, recent research has focused on C–H substitution at this position. Traditionally, this transformation has been performed using low-temperature lithiation chemistry,^9^ but modern methods have achieved it under preferable conditions by utilizing transition metal^10−12^ and photoredox catalysis (Figure 1b).^13,14^ Although synthesis of di-α-substituted heterocycles has been achieved through double reductive amination of diketone compounds,^15^ with key examples described in total and iminosugar syntheses,^16,17^ a general asymmetric catalytic platform is yet to be disclosed. Instead, strategies that prioritize redox-neutral cyclization chemistry (i.e., Paal-Knorr) followed by a global reduction are often preferred,^18^ despite the associated challenges in stereocontrol.

Biocatalysis has emerged as a robust platform for synthesis.^19^ It possesses distinct advantages over traditional synthetic reagents, such as operation under benign conditions, catalyst complementarity, excellent stereoselectivity, and no requirements for protecting groups. This potential has been realized most in amine synthesis, with multiple pharmaceutical process methods described, including contributions from Merck,^20^ GSK,^21^ Pfizer,^22,23^ Novartis,^24^ and others.^25^ While enzymes such as amine oxidases (MAOs)^26^ and imine reductases (IREDs)^27^ provide options for setting stereocenters in preformed *N*-heterocycles, cyclization through *N*-alkylation by a single enzyme has been long unfeasible due to known options such as transaminases (TAs)^28^ and amine dehydrogenases (AmDHs)^29^ being limited solely to primary amination. In 2017, an imine reductase subclass (reductive aminases, RedAms) capable of catalyzing full aqueous reductive amination was reported.^30^ This synthetic potential has since been realized through industrial application in the synthesis of several drug candidates on a process scale.^25^

One-pot cascade reactions showcase the potential of biocatalysts for their synthesis. This has been exemplified in heterocycle synthesis, where TA-IRED combinations have generated saturated *N*-heterocycles from dicarbonyl substrates.^31−33^ Recent metagenomic IRED panels have also increased the number of applications of these enzymes in cascades.^34,35^ This includes setting stereocenters following cyclization,^36−39^*N*-alkylation with redox surrogate reagents through a hydrogen borrowing approach,^40^ and combination with alcohol oxidase or carboxylic acid reductase for *N*-alkylated products.^41^ Recent work also demonstrated the combination of chemoenzymatic approaches with amine oxidases,^42^ and the use of aldolases to synthesize aminopolyols.^43^ There has also been disclosure of multifunctional biocatalysts that simultaneously mediated several synthetic steps in one go.^44^ Herein, we further demonstrate this multifunctional ability of IREDs to form *N*-heterocycles through a sequential inter–intramolecular reductive amination ring closure approach in the first report of these specific types of transformations (Figure 1c). This approach showcases the multifunctionality of RedAms,^44^ with the biocatalyst operating on two individual substrates derived from the starting material in a one-pot cascade fashion.

## Results and Discussion

### Oxidation-IRED Cascades

**Scheme 1:**
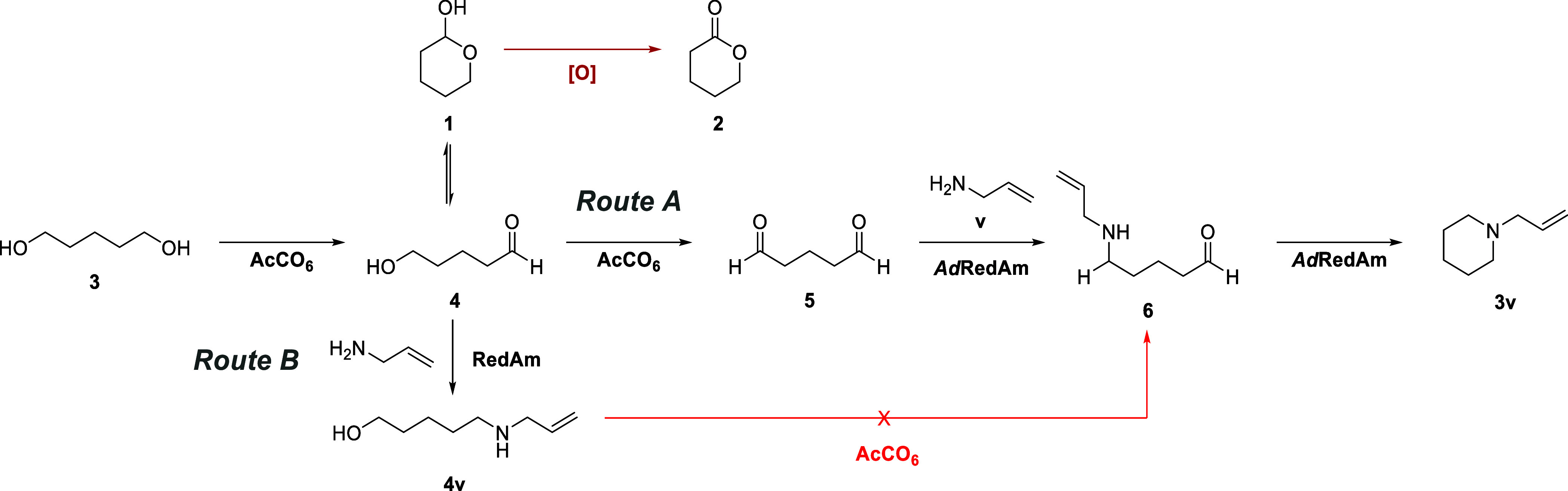
Potential Biocatalytic Routes for AcCo_6_*Ad*Redam Catalyzed Amine Diol Annulation

In a previous paper describing the engineering of AcCO6, we noted a general trend of high specific activity for terminal diol substrates.^45^ Inspired by this, we sought to compare our enzyme cascades to the chemical borrowing hydrogen literature, which inspired us to pursue a biocatalytic equivalent to the reported diol annulation chemistry. While alcohol oxidases have been previously applied to the oxidation of diols,^46^ access to the dialdehyde is often limited by overoxidation or the interception of intermediate hemiacetal tautomers to form lactones (Scheme 1).^47^

Initial analytical scale biotransformations analyzed by GC-MS showed no presence of carboxylic acids or δ-valerolactone **2** when 1,5-pentanediol **3** was subjected to oxidation by AcCO_6_, although glutaraldehyde **5** was also unobserved. We next sought to test the coupling of **3** with allylamine **v** under the standard AcCO_6_–RedAm cascade conditions outlined previously,^41^ with the better expressing “*Ad*RedAm” used in lieu of “*Asp*RedAm”. Unfortunately, we observed no product from this biotransformation. Hypothesizing that perhaps the intermediate hemiacetal intermediate tautomer **1** may be intercepted and oxidized by the glucose dehydrogenase (GDH) enzyme “CDX-901” required for cofactor recycling, we measured the specific activity of CDX-901 cell-free extract (CFE) with 5-hydroxypentanal **4** through an NADPH formation assay. The CDX-901 CFE was found to be active toward the oxidation of **4** and was replaced with the GDH from*Thermoplasma acidophilum*(*Ta*GDH).^48^ The side activity was not confirmed as being due to CDX-901 itself or another enzyme present in the crude preparation. To our delight, when the biotransformation was repeated with *Ta*GDH, we observed the formation of 1-allylpiperidine **3v**. We speculate that **5** was not observed due to its known role as an enzyme cross-linking reagent and that without further reaction it may simply cross-link enzymes involved in its formation. This clearly demonstrated the versatility of this class of enzyme, mediating two distinct reductive amination reactions, first using a primary amine donor and then a secondary amine donor. The bifunctionality was of interest, leading us to probe the reaction pathway, given the number of potential reaction partners (Scheme 1). The potential intermediate *N*-allyl-5-hydroxypentan-1-amine **4v** was synthesized and subjected alongside **4** to an oxidase activity screen as outlined previously.^45^ As **4** was shown to be a substrate for AcCO_6_ while **4v** was not (shown in red), we concluded that “Route A” was the mechanism by which the heterocycle was formed. Following this, we sought to establish the product scope for this cascade reaction.

Initially, a panel of diol substrates was subjected to activity screening (see Supporting Information). Alongside previously tested aliphatic diols, diethylene glycol and *N*-methyl-diethanolamine were screened to investigate whether the methodology may be applied to form morpholines and piperazines. While diethylene glycol and *N*-methyl-diethanolamine were moderately active substrates for the engineered and wild-type choline oxidases, respectively, applying them under IRED- cascade conditions suggested no product formation by GC-MS analysis. Despite this, correct masses for heterocyclic products were successfully detected from the panel of aliphatic diols (Table 1). While the formation of saturated heterocycles was observed for 6–8 membered rings, 1,4-butanediol **7** largely yielded products of mass consistent with pyrroles, possibly through a Paal-Knorr reaction. Incubating **9** with a primary amine and lone AcCO6 did not yield a possible pyrrole product, indicating the involvement of the IRED in this transformation. Both *N*-methylpyrrolidine **7iii** and *N*-methylpiperidine **3iii** were undetected by GC-MS, but we speculate this is due to coretention with the solvent due to the low boiling point. While the majority of products were observed as single peaks in GC chromatograms, no reaction conversions are claimed due to the potential for undetectable side reactions such as enzyme cross-coupling. Our attention then turned to the synthesis of α-substituted *N*-heterocycles. 5-Oxohexanol **13** and 6-oxoheptanol **14** were synthesized and found to be substrates for AcCO6. Subjecting these substrates to conditions identical to those utilized in the terminal diol work yielded a range of asymmetric heterocycles (Table 1). Where possible, *ee* and absolute configuration were determined using chiral GC-FID, although these assignments are largely limited due to challenges in achieving the separation of nonderivitizable products and accessing enantiomerically pure standards. The use of aryl ketones **15** and **16** yielded enamine substrates, with *Ad*RedAm unable to mediate the reduction of the more stable conjugated intermediate. Chemical reduction of these substrates still enabled a synthetic approach to 2-aryl substituted saturated *N*-heterocycles, but not in the same fashion as described above (see Supporting Information).

**Table 1:**
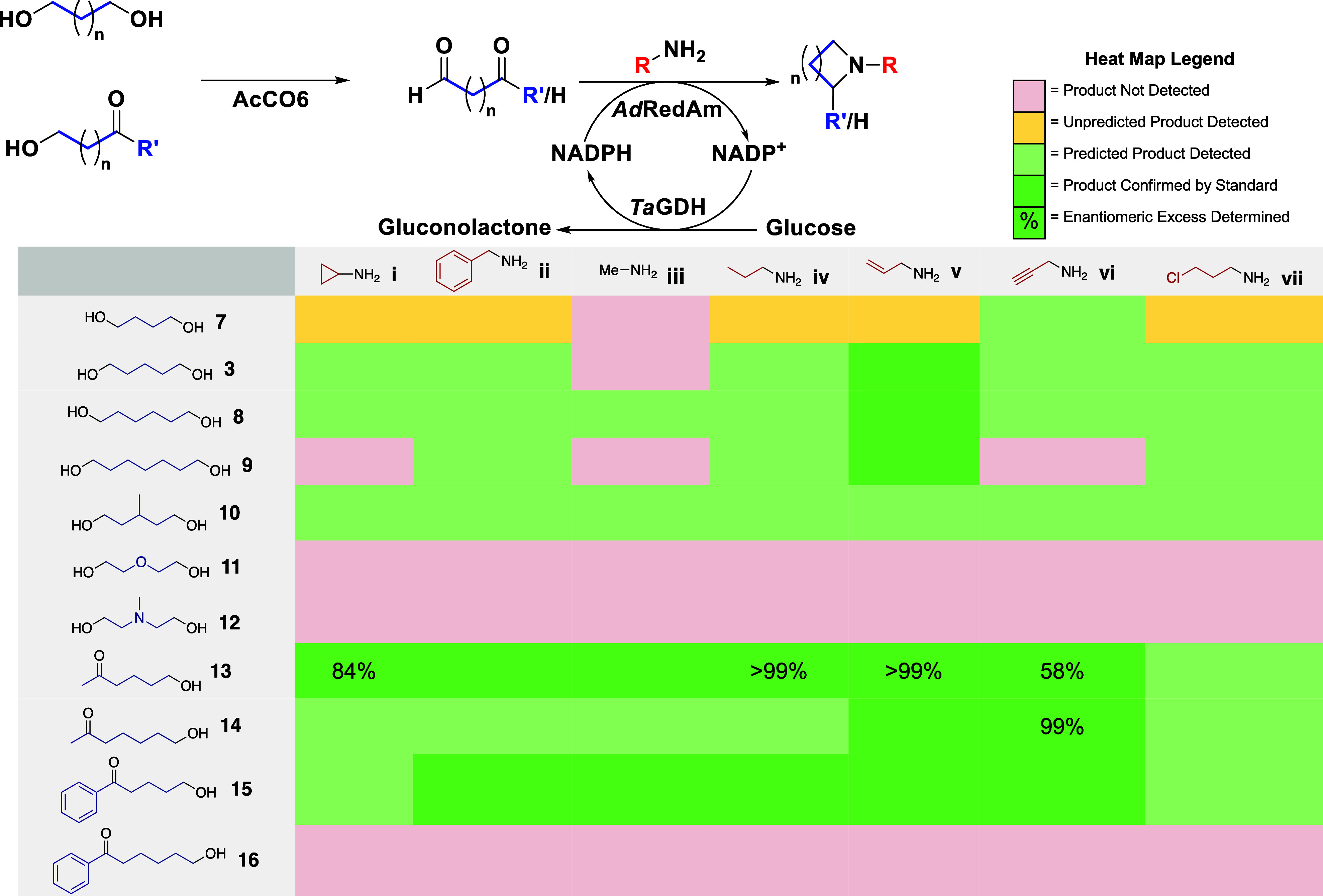
Activity Heat Map for the AcCO_6_-AdRedAm Catalyzed Synthesis of *N*-Heterocycles^a^

a Reaction conditions: 10 mM alcohol, 100 mM amine, 0.1 mM NADP^+^, 80 mM glucose, 1 mg mL^–1^*Ad*RedAm, 1 mg mL^–1^ AcCO_6_, 0.5 mg mL ^–1^*Ta*GDH, 0.5 mg mL ^–1^, 50 mg mL^–1^ 6-HDNO whole cells*, 40 mM NH_3_BH_3_*, 2% (v/v) DMSO, 100 mM pH 7.0 KPi buffer, 500 μL reaction volume, 30 °C, 250 rpm, 18 h. *Substrates 15 and 16 only.

**Scheme 2:**
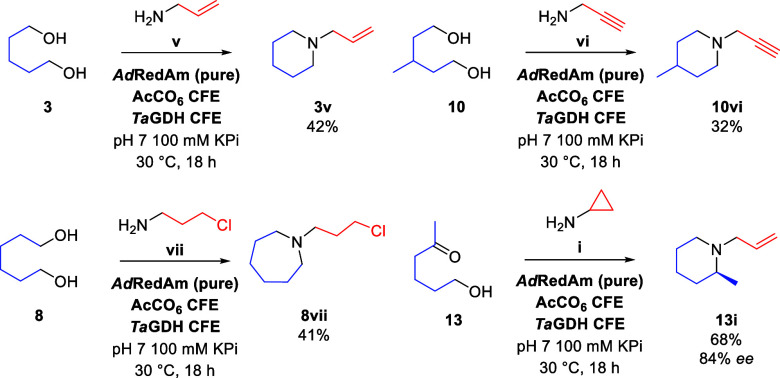
Preparative Scale AcCO6-IRED Cascade Reactions 10 mM diol, 100 mM amine, 200 μM NADP^+^, 80 mM glucose. See Supporting Information for full experimental details.

Following the establishment of the product scope, our attention moved to synthesis on a preparative scale. Through using a calibration curve, we were able to determine conditions that would yield piperidines and azepanes with high conversion, although we were not able to produce pyrroles or azocanes at conversions that were synthetically useful. Preparative scale reactions were performed on a 1 mmol scale with *N*-heterocycle products isolated by distillation (Scheme 2). Preparative scale synthesis was again performed on a 1 mmol scale on keto-alcohol precursor **13**, with (*S*)-*N*-cyclopropyl-2-methylpiperidine **13i** isolated in good yield and *ee* following distillation.

### IRED-Mediated Diketone Cyclization

We next sought to extend the approach to disubstituted *N*-heterocycles, specifically the synthesis of *N*-alkylated 2,5-disubstituted pyrrolidines from 1,4-diketones via a three-step one-pot cascade catalyzed solely by an IRED. Initially, *Ad*RedAm afforded *N*-methylpyrrolidine **21iii** in good to high conversions (>80%) from 2,5-decadione and methylamine, with no pyrrole formation observed. However, low diastereomeric ratios were observed under all conditions tested. The reactions also did not prove amicable toward scaling, with byproducts consistently observed.

In an attempt to improve the stereochemical outcome, 30 IREDs were selected from a metagenomic panel and screened against two 1,4-diketone substrates (**21** and **22**) and four amine partners (**i**, **iii**, **v**, **vi**) (see Supporting Information for complete set of IREDs and screening results).^35^ As a result, we were pleased to obtain all eight *N*-alkylated 2,5-dissubstituted pyrrolidines from the combination of the two diketones and four amines evaluated, in good to high conversions within 24 h (Table 2).

**Table 2:**
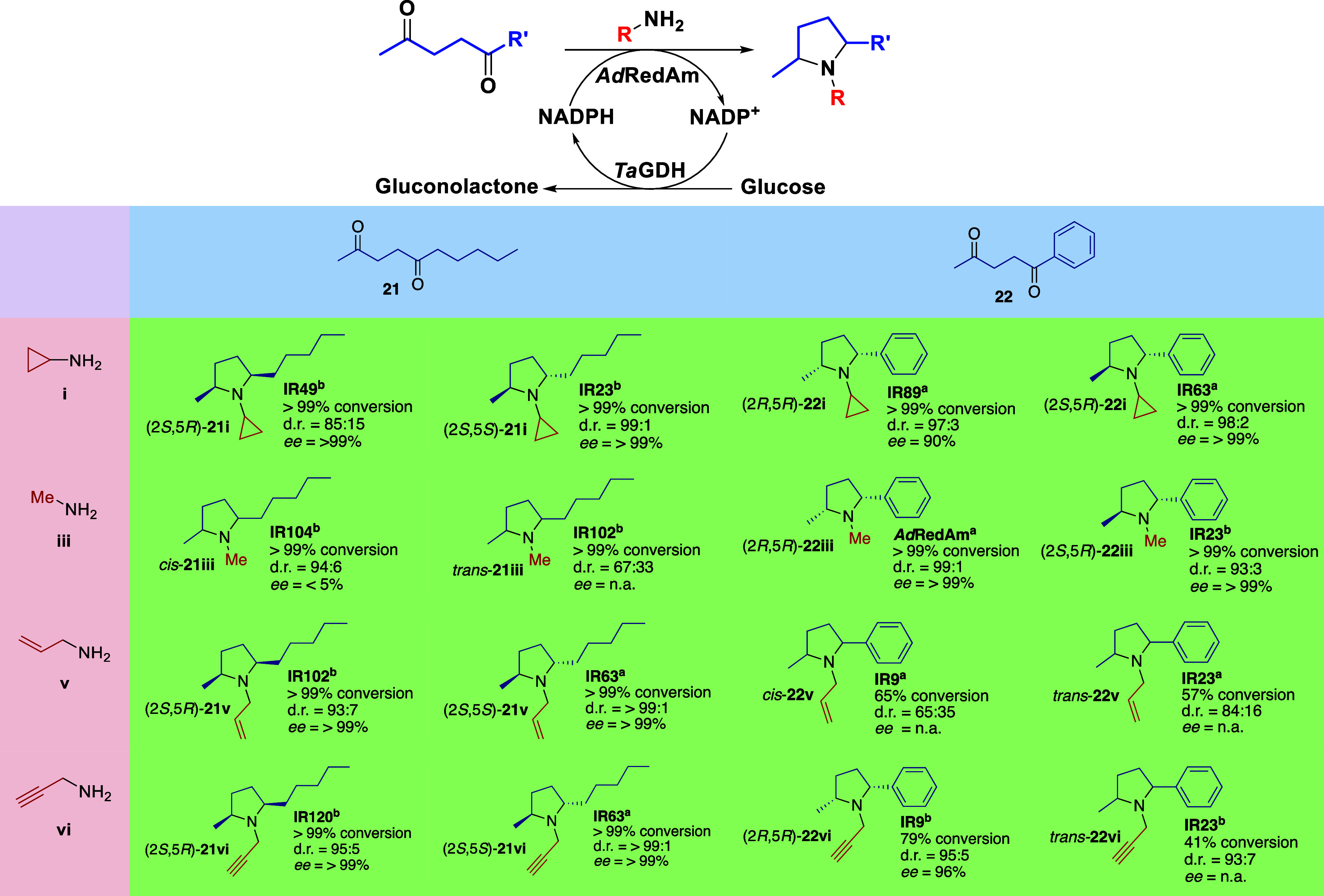
Product Scope of Diketone Annulation^a^

a Reaction conditions: 5 mM diketone, 100 mM amine, 0.5 mM NADP^+^, 50 mM glucose, 1 mg mL^–1^ purified IRED^a^ or 5 mg mL^–1^ IRED lyophilized cell-free extract, 0.5 mg mL^–1^ GDH (CDX-901), 1% (v/v) DMSO, 100 mM Tris buffer pH 9.0, 500 μL reaction volume, 30 °C, 250 rpm, 24 h.

To our surprise, the proposed cascade also allowed us to access both *cis* and *trans* pyrrolidines in good to excellent diastereo- and enantioselectivities, dependent on the biocatalyst utilized. These results show that some IREDs retain absolute enantiopreference in both steps, affording *trans* products (e.g., *2S,5S*-**21i** from IR23), while others switch selectivity to afford the *cis* products (e.g., *2S,5R*-**21i** from IR49). These findings indicate that the substrate binding modes differ broadly among the evaluated IREDs and the acyclic–cyclic imine intermediates. Furthermore, access to both diastereoisomers suggests that the diastereoselectivity is primarily governed by the enzyme and not chemically dictated by the previously defined stereocenter.

**Scheme 3:**
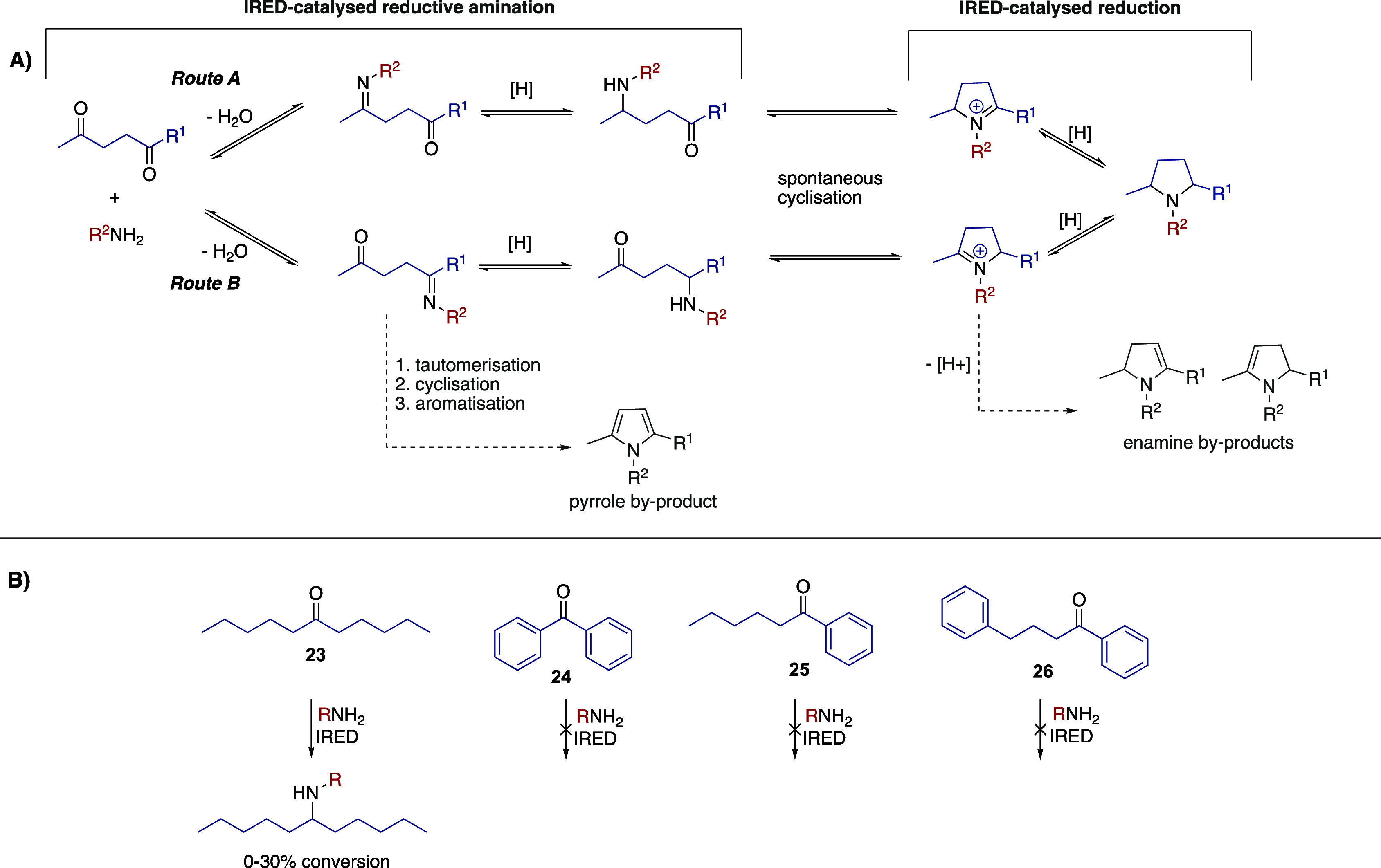
Investigating the Mechanism of IRED Catalyzed Diketone Annulation (A) Potential routes and byproducts thereof. (B) Bulky substrates evaluated.

Our proposed route assumed that the first reductive amination step occurs exclusively at the less hindered ketone moiety (Route A, Scheme 3a). To test this hypothesis, we evaluated four ketones (**23**–**26,**Scheme 3b) of analogous hindrances and electronic effects to the bulkier side of our diketones. None of the phenyl ketones (**24**–**26**) underwent reductive amination (see Supporting Information), implying that for diketone **22,** the cascade occurs exclusively through Route A (Scheme 3a). Conversely, amine products were observed when using pentyl ketone **23** as the substrate, indicating that Route B could occur simultaneously with Route A for reactions using diketone **21**. However, despite the presence of products, low conversions (10–30%) were obtained, which when combined with the fact that the selected IREDs showed high diastereo- and excellent enantioselectivies (Table 2), led us to conclude that Route A is the fastest, and most likely, the sole pathway followed in reactions using diketone **21**.

**Scheme 4:**
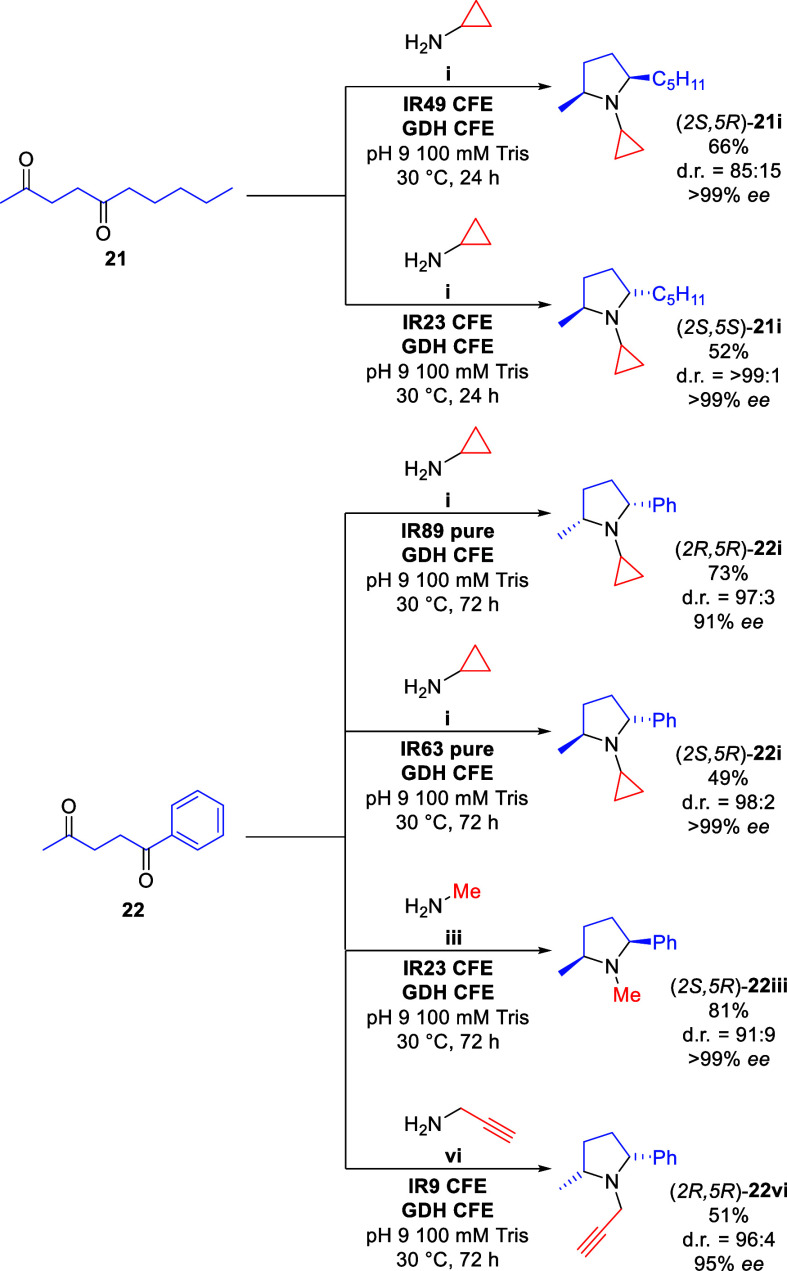
Preparative Scale Biocatalytic Diketone Annulation 6 mM diketone, 100 mM amine, 250 μM NADP^+^, 100 mM glucose. See Supporting Information for full experimental details.

Finally, to demonstrate the synthetic applicability of the investigated cascade, a series of preparative-scale syntheses were performed. Attempts to intensify reactions were unsuccessful, and consequently, the same conditions used on the analytical scale were applied for the preparative-scale reactions. Using our three-step one-enzyme cascade, six *N*-alkylated 2,5-disubstituted pyrrolidines were successfully synthesized on a 0.3 mmol scale (Scheme 4). Both diastereoisomers of pyrrolidines **21i** were synthesized with excellent enantioselectivities and good yields within 24 h, but 72 h was required for most other substrates. Typically, full substrate consumption was observed after 24 h and the enamine intermediate/byproduct was observed along with the desired product, followed solely by production of the desired product in the following 24–48 h.

## Conclusions

The work presented here has demonstrated the bifunctionality of a series of wild-type IREDs, able to catalyze multiple reductive aminations in a cascade sequence derived from the same starting materials. We have expanded the biocatalytic reaction toolbox through the demonstration of IRED-catalyzed annulation reactions and have demonstrated how enzyme engineering may influence catalysis with the first alcohol oxidase capable of generating dialdehydes with complete chemoselectivity. Through applying these enzymes in combination, four distinct synthetic steps were achieved within one pot, and transformations previously unique to precious metal-catalyzed borrowing hydrogen chemistry requiring forcing conditions are achieved at 30 °C in water with an expanded amine scope. The ability to form α-substituted heterocycles is a transformation underrepresented in heterogeneous catalysis, and here we demonstrate it with stereocontrol. Finally, by extending this work to the synthesis of *N*-alkylated α,α-disubstituted pyrrolidines from simple diketone starting materials, we have demonstrated a remarkable ability of these biocatalysts to build complexity with complete diastereomeric control. While there are limits to the synthetic application of our methodology, the recent expansion of our imine reductase panel demonstrated in the diketone cascade and the potential for the engineering of these enzymes toward this chemistry presents enormous potential. With the unprecedented speed with which IREDs have moved from discovery to industrial application, we are hopeful that this novel methodology may achieve a similar impact.

## Supporting Information Available

Materials, experimental procedures, analytic methods, GC traces and spectra, and ^1^H and ^13^C NMR spectra (PDF)

## Abbreviations

RedAm: reductive aminase
IRED: imine reductase
GDH: glucose dehydrogenase
AcCO_6_: alcohol oxidase
6-HDNO: 6-hydroxy-D-nicotine oxidase
TA: transaminase
CAR: carboxylic acid reductase
