# Supplementary material for: Bifunctional Imine Reductase Cascades for the Synthesis of Saturated N-Heterocycles
Source: ACS Catal. 2024 Sep 19;14(19):14703–10. doi: 10.1021/acscatal.4c03832 (PMC11459430; doi:10.1021/acscatal.4c03832)
Supplement: Supplementary file 1 — cs4c03832_si_001.pdf [file cs4c03832_si_001.pdf]

SUPPORTING INFORMATION

Imine Reductase Cascades for the Synthesis of Saturated *N*-Heterocycles

Jeremy I. Ramsden,[†] Bruna Z. Costa,[†] Rachel S. Heath,[†] James R. Marshall,[†] Sasha R. Derrington,[†] Juan Mangas-Sanchez,[†] Sarah L. Montgomery,[†] Keith R. Mulholland[§], Sebastian C. Cosgrove*[,†] and Nicholas J. Turner*[,†]

[†] Manchester Institute of Biotechnology, Department of Chemistry, University of Manchester. 131 Princess Street, Manchester M1 7DN, United Kingdom.

[§] Chemical Development, AstraZeneca, Silk Road Business Park, Macclesfield SK10 2NA, United Kingdom.

*Corresponding author: nicholas.turner@manchester.ac.uk; s.cosgrove@keele.ac.uk
Phone: +44 (161) 3065173. Fax: +44 (161) 2751311.

Table of Contents **Page**

Materials S2

General Methods S2

Biocatalyst Production Protocols S2

Analytical Scale Biotransformations S4

Optimisation of Reaction Conditions....................S5

IRED Screening for Diketone Cyclisation....................S7

Preparative Scale Biotransformations....................S12

Synthesis of Substrates and Chemical Standards....................S27

Specific Activity Screens S61

GC-MS Traces S62

Determination of Enantiomeric Excess S95

Materials

Commercially available chemicals and solvents were purchased from Sigma-Aldrich (Poole, Dorset, UK), Fluorochem (Hadfield, Derbyshire, UK), Alfa Aesar (Karlsruhe, Germany) or Prozomix (Haltwhistle, Northumberland, UK). (*R*)-2-phenylpiperidine was purchased from Apollo Scientific (Manchester, UK). Commercially available biocatalysts were supplied by Prozomix in crude cell-free extract form. 6-HDNO and (*R*)-IRED whole cells were produced according to procedures outlined in the literature.[1,2] CDX-901 was purchased directly from Codexis (CA, USA).

General Methods

Column chromatography was carried out using silica gel (Sigma-Aldrich, 230-400 mesh). Thin-layer chromatography (TLC) was performed on Merck Silica gel 60 F254 on aluminium foils and using phosphomolybdic acid ethanolic solution as stain. A Bruker Avance III 400 spectrometer (B_0 = 9.4 T) was used to record NMR spectra with chemical shifts reported in ppm relative to residual protic solvent signals. Coupling constants (*J*) are reported in Hz to the nearest 0.1 Hz.

High-resolution mass spectrometry (HRMS) was recorded using a Waters LCT time-of-flight mass spectrometer, connected to a Waters Alliance LC (Waters, Milford, MA, USA). Data were processed with Waters Masslynx software.

GC-FID analysis was performed on an Agilent 6850 GC (Agilent, Santa Clara, CA, USA) with a flame ionization detector (FID) and autosampler equipped with a CP-Chirasil-DEX CB column (25 m x 0.25 mm x0.25 μm, Agilent, Santa Clara, CA, USA).

GC-MS analysis was performed using an Agilent 7890B Series GC with 5977B MS-EI detector (Agilent, Santa Clara, CA, USA) in positive mode at a constant He flow. This instrument was also used for low resolution mass spectrometry. All compounds were analysed using an Agilent HP-1ms (30 m x column x 0.32 mm inner diameter x 0.25 μm, Agilent, Santa Clara, CA, USA).

Chiral HPLC was performed using an Agilent (Agilent, Santa Clara, CA, USA) system equipped with a G1312A binary pump, G1379A degasser, a G1367A well plate autosampler unit, a G1316A temperature-controlled column compartment and a G1315C diode array detector. A CHIRALCEL OD-H (25 mm x 4.6 mm x 5 μm, Daicel, Osaka Japan) column was used. An injection volume of 10 μL was used and chromatograms were monitored at 265 nm.

Biocatalyst Production Protocols

IRED expression

A single colony of *E. coli* BL21 (DE3) bearing the IRED plasmid was inoculated into 20 mL LB broth supplemented with kanamycin (35 μg/mL) and incubated at 37°C and 200 rpm for 18h. The overnight culture was used to inoculate a 2 L baffled Erlenmeyer flask containing 400 mL Terrific Broth (TB) supplemented with kanamycin (35 μg/mL). The culture was grown at 37°C and 200 rpm for 1.5-2 h (or when the OD_{600} reached 0.6 - 0.8). The recombinant protein expression was then induced by adding isopropyl β-D-1-thiogalactopyranoside (IPTG, 0.1 mM final concentration) and incubating at 23°C and 160 rpm. After 24 h, the cells were harvested by centrifugation (4°C, 4000 rpm, 20 min) and stored at -20 °C.

IRED lyophilised cell-free extract preparation

Cell pellets were resuspended in 100 mM NaPi buffer pH 7.0 (0.2 g/mL) and lysed, in an iced bath, by ultrasonication (6 cycles, 60 sec ON, 99 sec OFF, at 16 microns) using a MSE Soniprep 150 sonicator equipped with a 9.5 mm sonication probe. The resulting lysate was centrifuged (4 °C, 18,000 rpm, 60 min) and the obtained soluble fraction was transferred to a 250 mL round bottom flask and flash frozen using liquid nitrogen. The frozen lysate was then lyophilised for 24-48 h and the obtained cell-free extract powder was stored at -20 °C.

IRED purification from cell pellets

Cell pellets were resuspended in *buffer 1* (0.2 g/mL) and lysed, in an iced bath, by ultrasonication (6 cycles, 60 sec ON, 99 sec OFF, at 16 microns) using a MSE Soniprep 150 sonicator equipped with a 9.5 mm sonication probe. The resulting lysate was centrifuged (4 °C, 18,000 rpm, 60 min) and the obtained soluble fraction was loaded onto a 5 mL HiTrap Ni-NTA column (Qiagen) previously equilibrated with *buffer 1*. The loaded column was washed with *buffer 2* (25 mL) and *buffer 3* (15 mL) and the His_6-tagged protein was then eluted using *buffer 3* (12-20 mL, collected in 2 mL fractions). The elution fractions containing protein (>2mg/mL, determined at 280 nm) were combined, and then concentrated and buffer exchanged into *buffer 1* using a Vivaspin centrifugal concentrator MWCO 10,000 (GE Healthcare). Protein concentration was estimated by absorbance at 280 nm using a Nanodrop 1000 (Thermo Scientific). The purified and concentrated enzymes (1 mL aliquots at 10 mg/mL) were snap-frozen using liquid nitrogen and stored at -80 °C.

[Buffers used: *buffer 1* (100 mM Tris-HCl pH 8.0); *buffer 2* (30 mM imidazole, 100 mM Tris-HCl, pH 8.0); *buffer 3* (60 mM imidazole, 100 mM Tris-HCl, pH 8.0); *buffer 4* (300 mM imidazole, 100 mM Tris-HCl, pH 8.0)]

***Ad*RedAm purification from lyophilised cell-free extract**

Biocatalyst containing cell free extract (1g) was dissolved in 30 mL of a buffer composed of 90% *buffer A* and 10% *buffer B*. Using an ÄKTA purifier (GE Healthcare), a 5 mL His-Trap Crude FF column (GE Healthcare) charged with 0.1 M nickel sulphate was equilibrated with 90% *buffer A* 10% *buffer B*. The cell free extract solution was then loaded onto the column using a super-loop and subjected to a stepwise program of 90% *buffer A* 10% *buffer B* at 5 mL/min for 10 minutes, 80% *buffer A* 20% *buffer B* at 5 mL/min for 10 minutes before the protein was finally eluted through 100% *buffer B* at 5 mL/min for 20 minutes, collecting in 3 mL fractions. The fractions were analysed for protein content using a NanoDrop 1000 (Thermo Scientific) and fractions containing protein were concentrated and buffer exchanged into *buffer C*, by spin column (Vivaspin 20, 30 kDa cutoff, GE Healthcare). The protein was then snap-frozen using liquid nitrogen before storage at -80 °C.

[Buffers used: *buffer A* (100 mM pH 7.0 KPi buffer containing 300 mM NaCl); *buffer B* (100 mM pH 7.0 KPi buffer containing 300 mM NaCl and 300 mM imidazole); *buffer C* (100 mM pH 7.0 KPi buffer)].

Production of the Glucose Dehydrogenase from *Thermoplasma acidophilum*

A glycerol stock containing recombinant BL21(DE3) *E. coli* carrying a pET28a vector with the codon optimised GDH gene inserted was used to inoculate 6 mL of lysogeny broth (LB) medium (1% tryptone, 0.5% yeast extract, 1% NaCl) treated with kanamycin to a final concentration of 50 µg/mL in a 50 mL falcon tube before incubation for 16 h at 37 °C. This overnight culture was then used to inoculate a 600 mL culture of terrific broth (TB) medium (1.2% tryptone, 2.4% yeast extract, 0.5% glycerol) also treated with kanamycin to a final concentration of 50 µg/mL in a 2-litre baffled flask. This culture was grown at 37 °C shaking at 200 rpm for 2 h before the addition of L-rhamnose to a final 0.5% w/v concentration to induce gene expression. The culture was incubated at 28 °C and shaken at 200 rpm for 18 h before cells were harvested by centrifugation at 4000 rpm and 4 °C and re-suspended in 100 mM pH 7 KPi buffer before further centrifugation at 4000 rpm and 4 °C to yield the washed cell pellet. The cell pellet was again suspended in 100 mM pH 7 KPi buffer before the cells were lysed by ultrasonication (20 x 20 sec ON, 20 sec OFF cycles) using a Soniprep 150 (MSE UK Ltd.). The lysed cells were then incubated at 80 °C for 15 minutes before centrifugation at 18,000 rpm for 40 minutes. The resulting supernatant was then flash frozen using liquid nitrogen before freeze drying to yield the biocatalyst.

Analytical Scale Biotransformations

Cyclisation of Terminal Diol and Keto-Alcohol Substrates

Reactions were performed in a 500 µL reaction mixture containing 10 mM alcohol, 100 mM amine (in 100 mM KPi buffer adjusted to pH 7.0), 0.1 mM $NADP^+$, 80 mM glucose, 1 mg/mL *Ad*RedAm (purified in 100 mM pH 7.0 KPi buffer), 1 mg/mL AcCO6 (purified in 100 mM pH 7.0 KPi buffer), 1 mg/mL CDX-*Ta*GDH (cell free heat treated extract in 100 mM pH 7.0 KPi buffer) and 2% (v/v) DMSO. The reaction volume was made up to 500 µL with 100 mM pH 7.0 KPi buffer in a 2 mL Eppendorf tube and incubated at 30 °C with shaking at 200 rpm for 24 h. Reactions were adjusted to pH 12 with 5 M NaOH (50 µL) before extraction into either MTBE (for GC-FID/GC-MS) or Hexane (for HPLC) (600 µL x 2) with centrifugation to improve the separation of phases before the organic extracts were combined. The organic extracts were dried over $MgSO_4$ before GC-FID, GC-MS or HPLC analysis.

Cyclisation and Ammonia Borane Reduction of Benzoyl Alcohol Substrates

Reactions were performed to the general analytical procedure described above and after the initial 24 h reaction period were treated with a 1 M solution of ammonia borane in 100 mM pH 7.0 KPi buffer to reach a final concentration of 40 mM ammonia borane. The reactions were then continued for a further 16 hours before being subjected to work-up.

Cyclisation and Deracemisation of Benzoyl Alcohol Substrates

Reactions were performed to the general analytical procedure described above and after the initial 24 h reaction period were treated with 400 µL of a stock solution containing ammonia borane, catalase from bovine liver and whole 6-HDNO cells in 100 mM pH 7.0 KPi buffer to reach a final concentration of 40 mM ammonia borane, 0.5 mg/mL catalase from bovine liver and 50 mg/mL 6-HDNO whole cells. The reactions were then continued for a further 16 h before being subjected to work-up.

Cyclisation of Diketone Substrates

Enzymatic reactions were performed in a 500 μL-scale in 1.5 mL vials containing the diketone substrate (**21** or **22**, 5 mM), IRED (1 mg/mL of purified enzyme or 5 mg/mL of lyophilised cell-free extract), glucose dehydrogenase (CDX-901, 0.5 mg/mL), $NADP^+$ (0.5 mM), glucose (50 mM) and amine (100 mM) in Tris buffer (100 mM, pH 9). Reactions were incubated at 200 rpm and 30 °C for 24 h. Reactions were quenched by adding 5 M NaOH (20 μL) followed by extraction with *tert*-butyl methyl ether (MTBE, 500 μL). The organic layer was dried over $MgSO_4$ anhydrous and analysed by GC-MS for enzymatic conversion and chiral GC-FID for enantiomeric excess.

Optimisation of Reaction Conditions

AcCO6/*Ad*RedAm-catalysed Cyclisation

Although products were detected by GC-MS, often as single peaks, the difficulty in detecting the substrates and intermediates and the complexity of these cascade reactions would make determination of conversion from this data unsuitable. Therefore, a model reaction was optimised through the use of a product calibration curve.

Prior to the application of the terminal diol cyclisation methodology on a preparative scale, reaction parameters were varied on an analytical scale to determine the conditions most appropriate for the biocatalytic synthesis of *N*-allylpiperidine. Conversion was determined by extracting into MTBE spiked with 4.16 mM dodecane. A calibration curve for conversion to product was generated by performing extractions of 1-10 mM of chemical standard in buffer with 2% v/v DMSO to simulate reaction conversions from 10-100%. These conversions were plotted against their integration relative to the dodecane peak and a trendline was generated to yield a formula for estimating conversion to product in biotransformations.

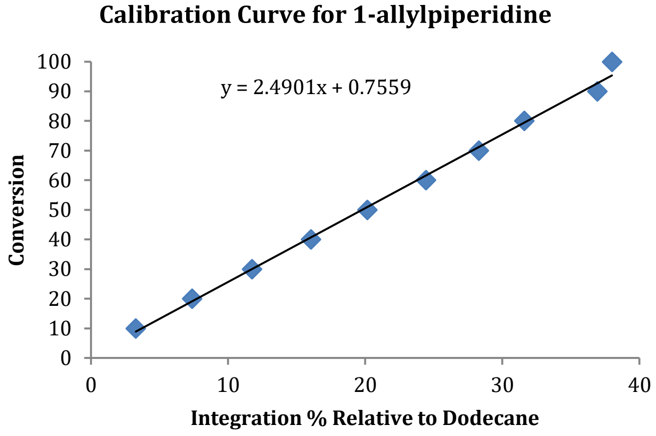

Equivalents of Amine

Loading of allylamine was varied to determine the effect on conversion. It was observed that a high equivalence of allylamine was optimal for conversion, but that very high loading leads to a fall in concentration.

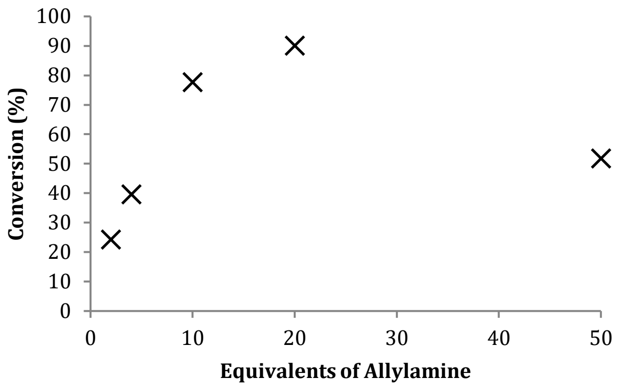

DMSO Concentration

The v/v percentage concentration of DMSO was varied to determine the effect on conversion. It was observed that the concentration of DMSO had little effect on conversion.

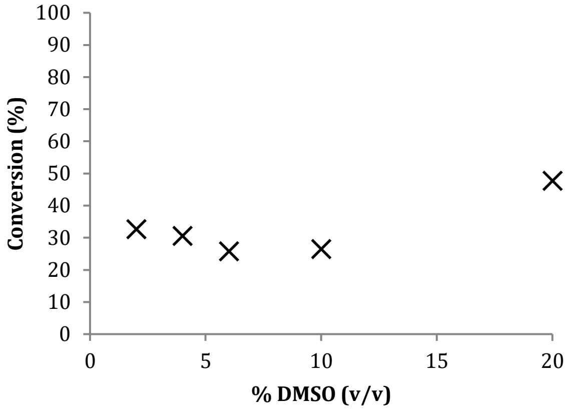

Buffer Concentration

The concentration of reaction buffer was varied to determine the effect on conversion. It was observed that changing the concentration of buffer had little effect on conversion.

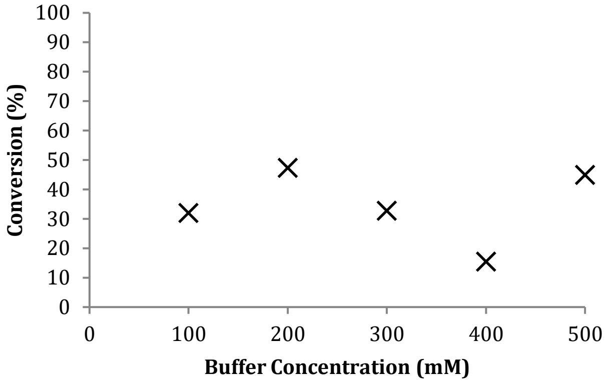

Substrate Concentration

Higher substrate concentrations were tested to determine whether the reaction could be intensified. It was observed that higher concentrations of substrate simply caused the reaction to fail.

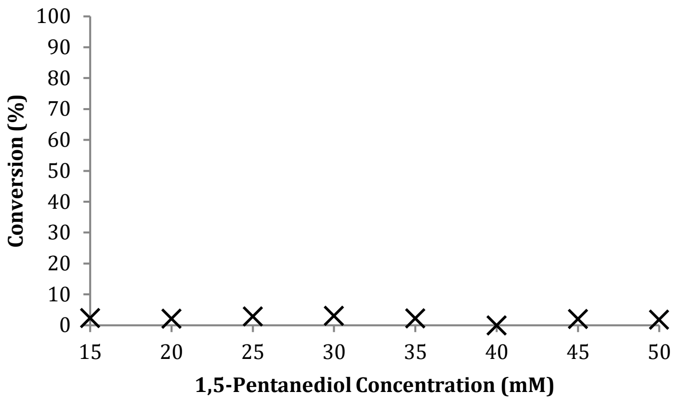

Biocatalyst Loading

Both the formulation and concentration of the two biocatalysts was varied to determine the ideal conditions for use in a preparative scale reaction. As was observed in the previous $AcCO_6$ and RedAm cascade manuscript,[3] $AcCO_6$ cell free extract (CFE) in combination with purified RedAm was suitable for these reactions.

Table S1. Biocatalyst loading evaluation.

**$AcCO_6$ CFE and *Ad*RedAm CFE**		**Pure $AcCO_6$ and *Ad*RedAm CFE**		**$AcCO_6$ CFE and Pure *Ad*RedAm**	
Concentration (mg /mL)	Conversion (%)	Concentration (mg/mL)	Conversion (%)	Concentration (mg /mL)	Conversion (%)
10 + 10	24	1 + 10	27	10 + 1	88
10 + 8	43	1 + 8	30	10 + 0.8	80
10 + 6	51	1 + 6	38	10 + 0.6	77
10 + 4	55	1 + 4	38	10 + 0.4	67
10 + 2	49	1 + 2	16	10 + 0.2	47

IRED Screening for Diketone Cyclisation

Thirty IREDs were screened following the general analytical scale procedure for cyclisation of diketone substrates. All IRED lyophilised cell-free extracts (cfe) are commercially available and were kindly supplied by Prozomix (Haltwhistle, UK). Thirteen IREDs were also analysed as purified enzymes (IR6, IR9, IR13, IR16, IR23, IR24, IR49, IR50, IR62, IR63, IR89, IR102, IR110).

Products/by-products distribution, consumption of substrate and conversion to the desired pyrrolidines were estimated by GC-MS (Table S2). A variety of by-products was observed in the screening, especially when using propargylamine as amine partner. However, only structures for pyrroles and cyclic enamines, the most common and abundant by-products, were proposed based on EI/MS fragmentation. A sum of all other by-products is reported as "Other(s)" on Table S2. Examples of analytical results and EI/MS spectra can be found in the "GC-MS traces" session.

Based on the conversion to the desired pyrrolidine and diastereomeric ratio results from the screening, enzymes were further selected for preparative-scale reactions.

Table S2. IRED screening for *N*-alkylated 2,5-disubstituted pyrrolidine synthesis

Reduction amination cyclisation cascade - IRED screening

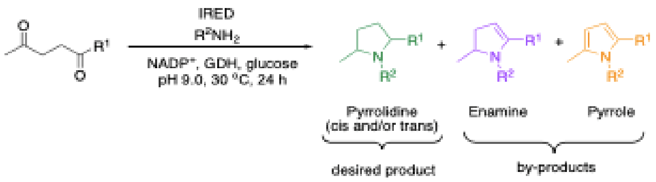

			Substrate consumption (%)	Conversion to desired product (%)	Product/intermediate/by-product distribution (%)				
IRED	Substrate	amine			*cis*-Pyrrolidine	*trans*-Pyrrolidine	Enamine	Pyrrole	Other (s)
IR6 (cfe)	**21**	**iii**	39	2	2	0	0	33	4
IR6 (cfe)	**21**	**v**	45	29	9	20	12	4	0
IR6 (cfe)	**21**	**i**	98	82	3	79	11	5	0
IR6 (cfe)	**21**	**vi**	100	50	6	44	0	4	46
IR6 (cfe)	**22**	**iii**	19	3	3	0	4	12	0
IR6 (cfe)	**22**	**v**	30	5	3	2	23	2	0
IR6 (cfe)	**22**	**i**	100	1	1	0	96	3	0
IR6 (cfe)	**22**	**vi**	100	19	11	8	0	0	81
IR6 (pur)	**21**	**iii**	26	2	2	0	0	24	0
IR6 (pur)	**21**	**v**	44	16	3	13	0	28	0
IR6 (pur)	**21**	**i**	100	92	3	89	2	6	0
IR6 (pur)	**21**	**vi**	100	61	15	46	0	1	38
IR6 (pur)	**22**	**iii**	16	5	5	0	0	7	4
IR6 (pur)	**22**	**v**	9	4	2	2	0	5	0
IR6 (pur)	**22**	**i**	100	0	0	0	97	3	0
IR6 (pur)	**22**	**vi**	100	27	15	12	0	0	73
IR9 (cfe)	**21**	**iii**	100	100	57	43	0	0	0
IR9 (cfe)	**21**	**v**	100	100	61	39	0	0	0
IR9 (cfe)	**21**	**i**	100	100	45	55	0	0	0
IR9 (cfe)	**21**	**vi**	100	100	80	20	0	0	0
IR9 (cfe)	**22**	**iii**	100	100	97	3	0	0	0
IR9 (cfe)	**22**	**v**	62	56	34	22	6	0	0
IR9 (cfe)	**22**	**i**	100	56	10	46	44	0	0
IR9 (cfe)	**22**	**vi**	100	80	73	7	0	0	20
IR9 (pur)	**21**	**iii**	100	100	58	42	0	0	0
IR9 (pur)	**21**	**v**	100	100	64	36	0	0	0
IR9 (pur)	**21**	**i**	100	100	52	48	0	0	0
IR9 (pur)	**21**	**vi**	100	100	89	11	0	0	0
IR9 (pur)	**22**	**iii**	100	100	98	2	0	0	0
IR9 (pur)	**22**	**v**	65	65	42	23	0		0
IR9 (pur)	**22**	**i**	100	84	18	66	0	3	13
IR9 (pur)	**22**	**vi**	80	59	38	21	0	0	21
IR13 (cfe)	**21**	**iii**	30	4	4	0	1	25	0
IR13 (cfe)	**21**	**v**	54	45	14	31	0	5	4
IR13 (cfe)	**21**	**i**	100	30	4	26	64	6	0
IR13 (cfe)	**21**	**vi**	100	44	11	33	0	2	54
IR13 (cfe)	**22**	**iii**	20	3	3	0	3	11	3
IR13 (cfe)	**22**	**v**	38	5	2	3	21	3	9
IR13 (cfe)	**22**	**i**	100	2	1	1	86	5	7
IR13 (cfe)	**22**	**vi**	97	14	8	6	0	0	83
IR13 (pur)	**21**	**iii**	17	0	0	0	0	17	0
IR13 (pur)	**21**	**v**	5	0	0	0	0	5	0
IR13 (pur)	**21**	**i**	100	50	7	43	31	19	0
IR13 (pur)	**21**	**vi**	99	31	12	19	0	0	68
IR13 (pur)	**22**	**iii**	0	0	0	0	0	0	0
IR13 (pur)	**22**	**v**	8	0	0	0	8	0	0
IR13 (pur)	**22**	**i**	100	0	0	0	100	0	0
IR13 (pur)	**22**	**vi**	100	15	5	10	1	0	84
IR16 (cfe)	**21**	**iii**	100	100	56	44	0	0	0
IR16 (cfe)	**21**	**v**	100	100	68	32	0	0	0
IR16 (cfe)	**21**	**i**	100	70	31	39	30	0	0
IR16 (cfe)	**21**	**vi**	100	100	82	18	0	0	0
IR16 (cfe)	**22**	**iii**	100	97	92	5	0	3	0
IR16 (cfe)	**22**	**v**	46	35	22	13	9	2	0
IR16 (cfe)	**22**	**i**	100	43	4	39	55	2	0
IR16 (cfe)	**22**	**vi**	100	72	68	4	0	0	28
IR16 (pur)	**21**	**iii**	100	100	56	44	0	0	0
IR16 (pur)	**21**	**v**	100	100	66	34	0	0	0
IR16 (pur)	**21**	**i**	100	96	47	49	4	0	0
IR16 (pur)	**21**	**vi**	100	100	87	13	0	0	0
IR16 (pur)	**22**	**iii**	100	100	95	5	0	0	0
IR16 (pur)	**22**	**v**	46	42	26	16	4	0	0
IR16 (pur)	**22**	**i**	99	66	6	60	33	0	0
IR16 (pur)	**22**	**vi**	100	80	69	11	0	0	20
IR23 (cfe)	**21**	**iii**	40	25	2	23	0	15	0
IR23 (cfe)	**21**	**v**	49	46	5	41	0	3	0
IR23 (cfe)	**21**	**i**	100	100	0	100	0	0	0

IR23 (cfe)	21	vi	32	13	0	13	0	9	10
IR23 (cfe)	22	iii	100	100	14	86	0	0	0
IR23 (cfe)	22	v	53	38	5	33	9	2	4
IR23 (cfe)	22	i	100	21	2	19	60	5	14
IR23 (cfe)	22	vi	74	39	3	36	1	0	34
IR23 (pur)	21	iii	63	63	5	58	0		0
IR23 (pur)	21	v	64	53	1	52	0	11	0
IR23 (pur)	21	i	100	100	0	100	0	0	0
IR23 (pur)	21	vi	66	52	1	51	0	10	4
IR23 (pur)	22	iii	94	89	8	81	0		5
IR23 (pur)	22	v	73	57	9	48	0	7	9
IR23 (pur)	22	i	100	13	0	13	85	2	0
IR23 (pur)	22	vi	79	41	12	29	0	0	38
IR24 (cfe)	21	iii	29	0	0	0	0	29	0
IR24 (cfe)	21	v	6	0	0	0	0	6	0
IR24 (cfe)	21	i	24	0	0	0	11	13	0
IR24 (cfe)	21	vi	43	0	0	0	0	10	33
IR24 (cfe)	22	iii	10	0	0	0	0	10	0
IR24 (cfe)	22	v	2	0	0	0	0	2	0
IR24 (cfe)	22	i	37	0	0	0	32	5	0
IR24 (cfe)	22	vi	32	0	0	0	0	0	32
IR49 (cfe)	21	iii	40	0	0	0	0	40	0
IR49 (cfe)	21	v	50	50	31	19	0	0	0
IR49 (cfe)	21	i	100	100	87	13	0	0	0
IR49 (cfe)	21	vi	35	30	13	17	0	5	0
IR49 (cfe)	22	iii	10	0	0	0	0	10	0
IR49 (cfe)	22	v	2	2	2	0	0	0	0
IR49 (cfe)	22	i	9	9	9	0	0	0	0
IR49 (cfe)	22	vi	2	2	2	0	0	0	0
IR49 (pur)	21	iii	32	0	0	0	0	32	0
IR49 (pur)	21	v	56	28	18	10	0	28	0
IR49 (pur)	21	i	100	100	85	15	0	0	0
IR49 (pur)	21	vi	65	60	25	35	0	5	0
IR49 (pur)	22	iii	6	0	0	0	0	6	0
IR49 (pur)	22	v	2	0	0	0	0	2	0
IR49 (pur)	22	i	25	21	21	0	0	4	0
IR49 (pur)	22	vi	9	9	9	0	0	0	0
IR50 (cfe)	21	iii	27	7	7	0	0	20	0
IR50 (cfe)	21	v	49	44	23	21	0	5	0
IR50 (cfe)	21	i	100	100	19	81	0	0	0
IR50 (cfe)	21	vi	95	42	18	24	0	5	48
IR50 (cfe)	22	iii	14	2	2	0	0	11	1
IR50 (cfe)	22	v	9	7	1	6	0	2	0
IR50 (cfe)	22	i	73	5	5	0	55	6	7
IR50 (cfe)	22	vi	60	33	2	31	0	0	27
IR50 (pur)	21	iii	76	66	57	9	0	10	0
IR50 (pur)	21	v	83	61	30	31	0	22	0
IR50 (pur)	21	i	100	100	26	74	0	0	0
IR50 (pur)	21	vi	100	64	30	34	0	0	36
IR50 (pur)	22	iii	60	50	19	31	0	10	0
IR50 (pur)	22	v	20	17	3	14	0	3	0
IR50 (pur)	22	i	84	70	55	15	0	6	8
IR50 (pur)	22	vi	59	40	5	35	0	0	19
IR62 (cfe)	21	iii	38	0	0	0	0	38	0
IR62 (cfe)	21	v	14	8	4	4	0	6	0
IR62 (cfe)	21	i	33	5	2	3	4	24	0
IR62 (cfe)	21	vi	47	37	0	37	0	10	0
IR62 (cfe)	22	iii	11	0	0	0	0	11	0
IR62 (cfe)	22	v	2	0	0	0	0	2	0
IR62 (cfe)	22	i	37	2	2	0	22	8	5
IR62 (cfe)	22	vi	31	2	2	0	0	0	29
IR62 (pur)	21	iii	34	0	0	0	0	34	0
IR62 (pur)	21	v	31	27	0	27	0	4	0
IR62 (pur)	21	i	33	16	5	11	9	8	0
IR62 (pur)	21	vi	66	60	0	60	0	6	0
IR62 (pur)	22	iii	0	0	0	0	0	0	0
IR62 (pur)	22	v	0	0	0	0	0	0	0
IR62 (pur)	22	i	100	84	0	84	16	0	0
IR62 (pur)	22	vi	20	20	0	20	0	0	0
IR63 (cfe)	21	iii	35	10	3	7	10	11	4
IR63 (cfe)	21	v	22	15	0	15	0	7	0
IR63 (cfe)	21	i	0	0					
IR63 (cfe)	21	vi	34	24	0	24	0	10	0
IR63 (cfe)	22	iii	8	0	0	0	0	8	0
IR63 (cfe)	22	v	2	0	0	0	0	2	0
IR63 (cfe)	22	i	52	0	0	0	23	8	21
IR63 (cfe)	22	vi	0	0	0	0	0	0	0
IR63 (pur)	21	iii	100	90	22	68	7	3	0
IR63 (pur)	21	v	89	82	2	80	0	7	0
IR63 (pur)	21	i	100	100	7	93	0	0	0

IR63 (pur)	21	vi	100	100	0	100	0	0	0
IR63 (pur)	22	iii	82	62	19	43	0	9	11
IR63 (pur)	22	v	14	11	0	11	0	3	0
IR63 (pur)	22	i	100	100	2	98	0	0	0
IR63 (pur)	22	vi	33	26	2	24	0	0	7
IR85 (cfe)	21	iii	31	0	0	0	0	31	0
IR85 (cfe)	21	v	5	0	0	0	0	5	0
IR85 (cfe)	21	i	14	0	0	0	4	10	0
IR85 (cfe)	21	vi	13	0	0	0	0	13	0
IR85 (cfe)	22	iii	14	0	0	0	0	14	0
IR85 (cfe)	22	v	2	0	0	0	0	2	0
IR85 (cfe)	22	i	5	0	0	0	0	5	0
IR85 (cfe)	22	vi	2	0	0	0	0	0	2
IR89 (cfe)	21	iii	27	12	9	3	2	13	0
IR89 (cfe)	21	v	19	11	11	0	0	8	0
IR89 (cfe)	21	i	90	67	35	32	0	23	0
IR89 (cfe)	21	vi	27	5	5	0	0	10	12
IR89 (cfe)	22	iii	43	15	15	0	0	13	15
IR89 (cfe)	22	v	3	1	1	0	0	2	0
IR89 (cfe)	22	i	71	32	32	0	23	12	4
IR89 (cfe)	22	vi	9	3	3	0	0	0	6
IR89 (pur)	21	iii	100	100	83	17	0	0	0
IR89 (pur)	21	v	56	53	46	7	0	3	0
IR89 (pur)	21	i	100	100	52	48	0	0	0
IR89 (pur)	21	vi	100	62	42	20	0	0	38
IR89 (pur)	22	iii	100	100	100	0	0	0	0
IR89 (pur)	22	v	14	14	14	0	0	0	0
IR89 (pur)	22	i	100	100	97	3	0	0	0
IR89 (pur)	22	vi	60	47	29	18	0	0	13
IR92 (cfe)	21	iii	25	0	0	0	0	25	0
IR92 (cfe)	21	v	0	0	0	0	0	0	0
IR92 (cfe)	21	i	0	0	0	0	0	0	0
IR92 (cfe)	21	vi	21	0	0	0	0	21	0
IR92 (cfe)	22	iii	4	0	0	0	0	4	0
IR92 (cfe)	22	v	0	0	0	0	0	0	0
IR92 (cfe)	22	i	3	0	0	0	0	3	0
IR92 (cfe)	22	vi	0	0	0	0	0	0	0
IR102 (cfe)	21	iii	100	100	32	68	0	0	0
IR102 (cfe)	21	v	95	95	89	6	0	0	0
IR102 (cfe)	21	i	100	100	85	15	0	0	0
IR102 (cfe)	21	vi	94	88	57	31	0	6	0
IR102 (cfe)	22	iii	31	25	3	22	0	6	0
IR102 (cfe)	22	v	9	7	4	3	2	0	0
IR102 (cfe)	22	i	100	40	31	9	60	0	0
IR102 (cfe)	22	vi	15	15	1	14	0	0	0
IR102 (pur)	21	iii	100	100	33	67	0	0	0
IR102 (pur)	21	v	100	100	91	9	0	0	0
IR102 (pur)	21	i	100	100	83	17	0	0	0
IR102 (pur)	21	vi	100	100	53	47	0	0	0
IR102 (pur)	22	iii	51	44	7	37	0	7	0
IR102 (pur)	22	v	20	17	9	8	0	3	0
IR102 (pur)	22	i	100	100	72	28	0	0	0
IR102 (pur)	22	vi	47	47	21	26	0	0	0
IR104 (cfe)	21	iii	100	100	94	6	0	0	0
IR104 (cfe)	21	v	68	68	37	31	0	0	0
IR104 (cfe)	21	i	100	100	34	66	0	0	0
IR104 (cfe)	21	vi	75	65	37	28	0	10	0
IR104 (cfe)	22	iii	55	53	50	3	0	2	0
IR104 (cfe)	22	v	12	9	5	4	3	0	0
IR104 (cfe)	22	i	100	36	12	24	50	3	11
IR104 (cfe)	22	vi	34	34	20	14	0	0	0
IR110 (cfe)	21	iii	34	0	0	0	0	34	0
IR110 (cfe)	21	v	10	0	0	0	0	10	0
IR110 (cfe)	21	i	17	0	0	0	0	17	0
IR110 (cfe)	21	vi	19	4	3	1	0	15	0
IR110 (cfe)	22	iii	9	0	0	0	0	9	0
IR110 (cfe)	22	v	0	0	0	0	0	0	0
IR110 (cfe)	22	i	5	1	1	0	0	4	0
IR110 (cfe)	22	vi	0	0	0	0	0	0	0
IR110 (pur)	21	iii	80	30	30	0	0	25	25
IR110 (pur)	21	v	68	0	0	0	0	34	34
IR110 (pur)	21	i	77	51	14	37	0	13	13
IR110 (pur)	21	vi	30	14	8	6	0	8	8
IR110 (pur)	22	iii	14	0	0	0	0	7	7
IR110 (pur)	22	v	0	0	0	0	0	0	0
IR110 (pur)	22	i	0	0	0	0	0	0	0
IR110 (pur)	22	vi	0	0	0	0	0	0	0
IR111 (cfe)	21	iii	49	21	16	5	0	28	0
IR111 (cfe)	21	v	30	24	24	0	0	6	0
IR111 (cfe)	21	i	20	8	8	0	0	12	0

IR111 (cfe)	**21**	vi	29	16	16	0	0	13	0
IR111 (cfe)	**22**	iii	16	0	0	0	0	16	0
IR111 (cfe)	**22**	v	2	0	0	0	0	2	0
IR111 (cfe)	**22**	i	5	0	0	0	0	5	0
IR111 (cfe)	**22**	vi	1	0	0	0	0	1	0
IR112 (cfe)	**21**	iii	28	0	0	0	0	28	0
IR112 (cfe)	**21**	v	8	0	0	0	0	8	0
IR112 (cfe)	**21**	i	22	0	0	0	0	22	0
IR112 (cfe)	**21**	vi	15	0	0	0	0	15	0
IR112 (cfe)	**22**	iii	14.5	3.5	1.8	1.7	0	11	0
IR112 (cfe)	**22**	v	3	3	0	3	0	0	0
IR112 (cfe)	**22**	i	18	16	2	14	0	2	0
IR112 (cfe)	**22**	vi	0	0	0	0	0	0	0
IR117 (cfe)	**21**	iii	100	100	40	60	0	0	0
IR117 (cfe)	**21**	v	100	100	22	78	0	0	0
IR117 (cfe)	**21**	i	100	100	61	39	0	0	0
IR117 (cfe)	**21**	vi	100	100	4	96	0	0	0
IR117 (cfe)	**22**	iii	53	43	5	38	0	10	0
IR117 (cfe)	**22**	v	33	27	10	17	4	2	0
IR117 (cfe)	**22**	i	100	44	30	14	45	5	6
IR117 (cfe)	**22**	vi	25	18	3	15	0	1	6
IR120 (cfe)	**21**	iii	100	100	74	26	0	0	0
IR120 (cfe)	**21**	v	80	78	76	2	0	2	0
IR120 (cfe)	**21**	i	99	95	81	14	0	4	0
IR120 (cfe)	**21**	vi	99	95	90	5	0	4	0
IR120 (cfe)	**22**	iii	25	14	10	4	0	11	0
IR120 (cfe)	**22**	v	9	9	3	6	0	0	0
IR120 (cfe)	**22**	i	9	5	5	0	0	4	0
IR120 (cfe)	**22**	vi	30	30	3	27	0	0	0
IR150 (cfe)	**21**	iii	35	0	0	0	0	35	0
IR150 (cfe)	**21**	v	10	0	0	0	0	10	0
IR150 (cfe)	**21**	i	11	0	0	0	0	11	0
IR150 (cfe)	**21**	vi	7	0	0	0	0	7	0
IR150 (cfe)	**22**	iii	6	0	0	0	0	6	0
IR150 (cfe)	**22**	v	0	0	0	0	0	0	0
IR150 (cfe)	**22**	i	0	0	0	0	0		0
IR150 (cfe)	**22**	vi	0	0	0	0	0		0
IR171 (cfe)	**21**	iii	40	0	0	0	0	40	0
IR171 (cfe)	**21**	v	13	0	0	0	0	13	0
IR171 (cfe)	**21**	i	28	0	0	0	15	13	0
IR171 (cfe)	**21**	vi	11	4	4	0	0	7	0
IR171 (cfe)	**22**	iii	6	0	0	0	0	6	0
IR171 (cfe)	**22**	v	0	0	0	0	0	0	0
IR171 (cfe)	**22**	i	0	0	0	0	0		0
IR171 (cfe)	**22**	vi	0	0	0	0	0		0
IR172 (cfe)	**21**	iii	28	0	0	0	0	28	0
IR172 (cfe)	**21**	v	15	0	0	0	0	15	0
IR172 (cfe)	**21**	i	57	38	34	4	5	14	0
IR172 (cfe)	**21**	vi	18	0	0	0	0	18	0
IR172 (cfe)	**22**	iii	8	0	0	0	0	8	0
IR172 (cfe)	**22**	v	4	4	4	0	0	0	0
IR172 (cfe)	**22**	i	100	16	16	0	79	5	0
IR172 (cfe)	**22**	vi	0	0	0	0	0		0
IR202 (cfe)	**21**	iii	40	19	14	5	0	21	0
IR202 (cfe)	**21**	v	81	72	21	51	0	9	0
IR202 (cfe)	**21**	i	67	41	5	36	11	15	0
IR202 (cfe)	**21**	vi	100	100	17	83	0	0	0
IR202 (cfe)	**22**	iii	28	17	17	0	0	11	0
IR202 (cfe)	**22**	v	36	34	6	28	1	1	0
IR202 (cfe)	**22**	i	80	22	2	20	36	7	15
IR202 (cfe)	**22**	vi	100	65	25	40	0	0	35
IR249 (cfe)	**21**	iii	0	0	0	0	0	0	0
IR249 (cfe)	**21**	v	0	0	0	0	0	0	0
IR249 (cfe)	**21**	i	0	0	0	0	0	0	0
IR249 (cfe)	**21**	vi	0	0	0	0	0	0	0
IR249 (cfe)	**22**	iii	0	0	0	0	0	0	0
IR249 (cfe)	**22**	v	0	0	0	0	0	0	0
IR249 (cfe)	**22**	i	0	0	0	0	0	0	0
IR249 (cfe)	**22**	vi	0	0	0	0	0	0	0
IR282 (cfe)	**21**	iii	0	0	0	0	0	0	0
IR282 (cfe)	**21**	v	0	0	0	0	0	0	0
IR282 (cfe)	**21**	i	0	0	0	0	0	0	0
IR282 (cfe)	**21**	vi	0	0	0	0	0	0	0
IR282 (cfe)	**22**	iii	0	0	0	0	0	0	0
IR282 (cfe)	**22**	v	0	0	0	0	0	0	0
IR282 (cfe)	**22**	i	0	0	0	0	0	0	0
IR282 (cfe)	**22**	vi	0	0	0	0	0	0	0
IR355 (cfe)	**21**	iii	100	100	62	38	0	0	0
IR355 (cfe)	**21**	v	100	100	72	28	0	0	0
IR355 (cfe)	**21**	i	100	78	43	35	7	0	15

IR355 (cfe)	21	vi	100	70	62	8	0	0	30
IR355 (cfe)	22	iii	100	83	83	0	0	0	17
IR355 (cfe)	22	v	73	24	6	18	30	4	15
IR355 (cfe)	22	i	100	5	3	2	89	6	0
IR355 (cfe)	22	vi	100	21	18	3	0	0	79
IR356 (cfe)	21	iii	32	2	2	0	0	30	0
IR356 (cfe)	21	v	9	0	0	0	0	9	0
IR356 (cfe)	21	i	34	11	11	0	11	12	0
IR356 (cfe)	21	vi	15	0	0	0	0	15	0
IR356 (cfe)	22	iii	16	1	1	0	0	15	0
IR356 (cfe)	22	v	2	0	0	0	0	2	0
IR356 (cfe)	22	i	6	0	0	0	0	6	0
IR356 (cfe)	22	vi	1	0	0	0	0	1	0
IR361 (cfe)	21	iii	70	58	25	33	0	0	12
IR361 (cfe)	21	v	55	51	5	46	0	0	4
IR361 (cfe)	21	i	100	89	42	47	0	0	11
IR361 (cfe)	21	vi	100	71	3	68	0	0	29
IR361 (cfe)	22	iii	100	100	84	16	0	0	0
IR361 (cfe)	22	v	0	0					
IR361 (cfe)	22	i	99	8	2	6	76	6	9
IR361 (cfe)	22	vi	100	76	58	18	0	0	24
AdRedAm (cfe)	21	iii	90	78	52	26	12	0	0
AdRedAm (cfe)	21	v	99	68	51	17	0	5	26
AdRedAm (cfe)	21	i	100	60	31	29	0	28	12
AdRedAm (cfe)	21	vi	100	52	44	8	0	0	48
AdRedAm (cfe)	22	iii	84	46	46	0	0	6	32
AdRedAm (cfe)	22	v	36	14	4	10	3	7	12
AdRedAm (cfe)	22	i	99	4	2	2	74	7	14
AdRedAm (cfe)	22	vi	100	22	14	8	0	0	78
AdRedAm (pur)	21	iii	100	100	62	38	0	0	0
AdRedAm (pur)	21	v	100	100	69	31	0	0	0
AdRedAm (pur)	21	i	100	91	47	44	0	0	9
AdRedAm (pur)	21	vi	100	76	69	7	0	0	24
AdRedAm (pur)	22	iii	100	100	100	0	0	0	0
AdRedAm (pur)	22	v	53	41	9	32	0	12	0
AdRedAm (pur)	22	i	100	8	5	3	90	2	0
AdRedAm (pur)	22	vi	86	8	8	0	0	0	78

Preparative Scale Biotransformations

Synthesis of (*R*)-2-Methylazepane Hydrochloride

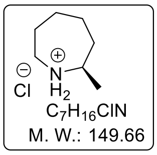

To pH 7 100 mM KPi Buffer (25 mL) in a 50 mL falcon tube were added fresh recombinant *E. coli* BL21(DE3) cells overexpressing the (*R*)-IRED from *Streptomyces sp.* GF3587 (1.5 g), glucose (225 mg, 1.25 mmol), and 7-methyl-3,4,5,6-tetrahydro-2H-azepin-1-ium chloride (80 mg, 0.54 mmol). The solution was incubated in an orbital shaker at 30 °C and 200 rpm for 24 h before basification to pH 12 with the addition of 5 M NaOH. The crude product was then extracted into diethyl ether (2 x 20 mL) with intermediate centrifugation to improve the separation of phases. The combined organic extracts were then dried, filtered and treated with HCl in diethyl ether (2 M, 2 mL). The solvent was removed by rotary evaporation and the resulting salt was washed with hexane to yield pure (*R*)-2-methylazepane as a pink solid (58 mg, 70%). **^{1}H NMR** (400 MHz, methanol-d_4) δ 3.51 – 3.37 (m, 1H), 3.30 (s, 1H), 3.22 – 3.11 (m, 1H), 2.04 – 1.78 (m, 5H), 1.72 – 1.57 (m, 3H), 1.37 (d, *J* = 6.7 Hz, 3H). **^{13}C NMR** (101 MHz, Methanol-d_4) δ 56.39, 46.27, 34.27, 27.42, 26.00, 25.59, 20.48. ***m/z* (±ES)** (freebase) 98.1 $[M-CH_3]^+$, 113.1 $[M]^+$.

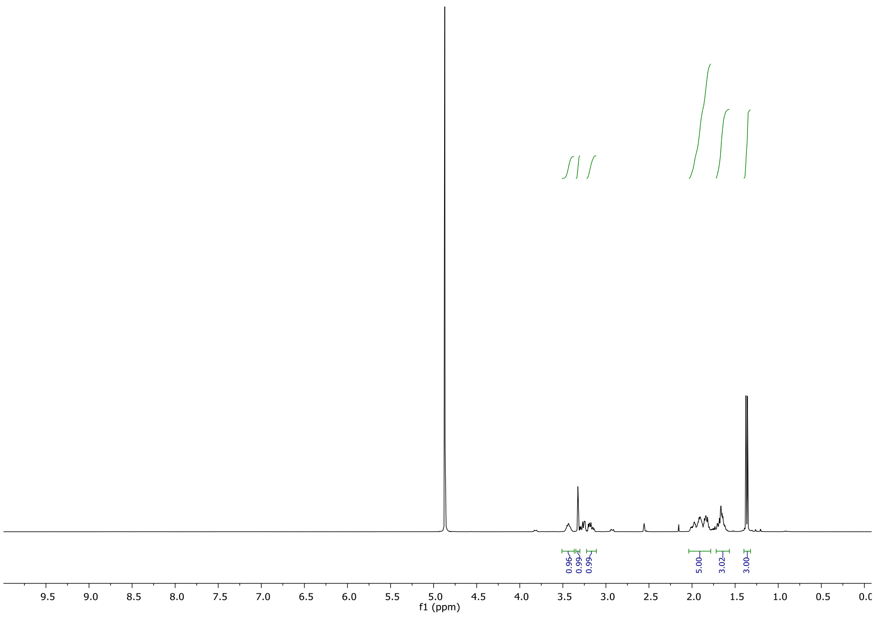

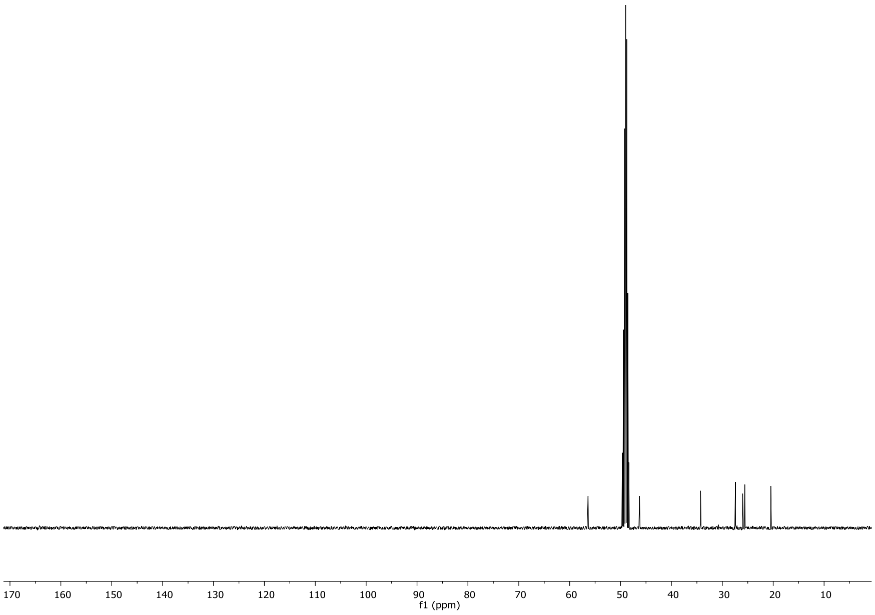

Synthesis of *N*-allylpiperidine hydrochloride (3v)

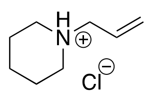

3v
$C_8H_{15}N.HCl$
M.W.: 161.67

To a 500 mL glass bottle were added 1,5-pentanediol (1.0 mmol, 104 mg), $NADP^+$ (15 mg, 20 µmol), glucose (1.4 g, 7.7 mmol), *Ad*RedAm (400 mg, cell free extract), *Ta*GDH (50 mg, cell free extract), $AcCO_6$ (1 g, cell free extract), DMSO (2 mL), a pH adjusted stock solution of allylamine (1M in 100 mM pH 7.0 KPi buffer, 10 mL) and 100 mM pH 7.0 KPi buffer (88 mL). The bottle was sealed before incubation at 30 °C in an orbital shaker at 250 rpm for 18h. The reaction was decanted into falcon tubes and basified to a pH of 12.0 with 5 M NaOH solution before extraction into diethyl ether (2 x 4 x 20 mL) with intermediate centrifugation to improve the separation of phases (4 °C, 4000 rpm, 20 min). The combined organic extracts were dried over $MgSO_4$ and filtered before the solvent was removed by rotary evaporation to give the crude product. The crude product was purified by Kugelrohr distillation followed by the addition of

a solution of HCl in diethyl ether (2 M, 2 mL). The solvent was then removed by rotary evaporation to yield the pure hydrochloride salt as a white solid (68 mg, 0.42 mmol, 42%). **^{1}H NMR** (400 MHz, Methanol-*d*$_4$) δ 5.90 (ddt, *J* = 17.3, 10.2, 7.2 Hz, 1H), 5.61 – 5.43 (m, 2H), 3.65 (d, *J* = 7.2 Hz, 2H), 3.42 (ddd, *J* = 12.1, 3.9, 2.0 Hz, 2H), 2.83 (td, *J* = 12.8, 12.0, 2.4 Hz, 2H), 1.93 – 1.80 (m, 2H), 1.80 – 1.64 (m, 3H), 1.49 – 1.36 (m, 1H). **^{13}C NMR** (101 MHz, Methanol-*d*$_4$) δ 127.95, 126.80, 60.52, 54.00, 24.49, 22.88. ***m/z* (±ES)** (freebase) 98.1, [M-C_2H_3]$^+$, 110.1 [M-CH_3]$^+$, 125.1 [M]$^+$.

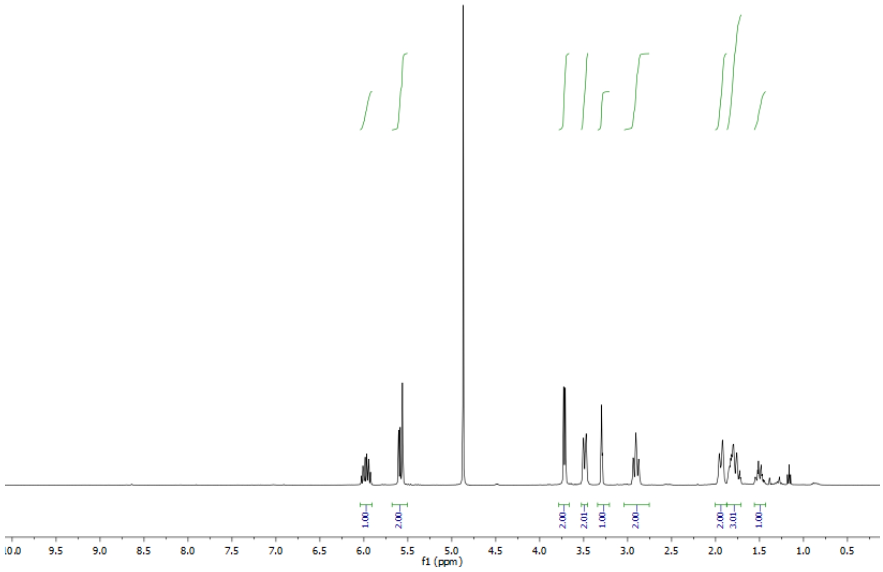

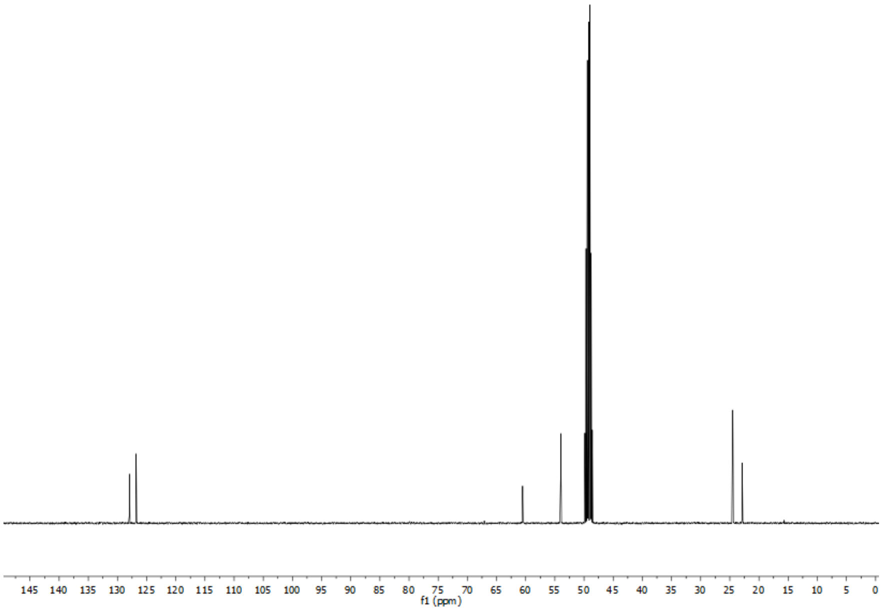

Synthesis of *N*-propargyl-4-methylpiperidine hydrochloride (12vi)

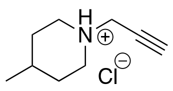

12vi
$C_9H_{15}N.HCl$
M.W.: 173.68

To a 500 mL glass bottle were added 3-methylpentane-1,5-diol (1.0 mmol, 118 mg), NADP$^+$ (15 mg, 20 μmol), glucose (1.4 g, 7.7 mmol), *Ad*RedAm (40 mg, purified enzyme), *Ta*GDH (50 mg, cell free extract), AcCO$_6$ (1 g, cell free extract), DMSO (2 mL), a pH adjusted stock solution of propargylamine (1M in 100 mM pH 7.0 KPi buffer, 10 mL) and 100mM pH 7.0 KPi buffer (88 mL). The bottle was sealed before incubation at 30 °C in an orbital shaker at 250 rpm for 18h. The reaction was decanted into falcon tubes and basified to a pH of 12.0

with 5 M NaOH solution before extraction into diethyl ether (2 x 4 x 20 mL) with intermediate centrifugation to improve the separation of phases (4 °C, 4000 rpm, 20 min). The combined organic extracts were dried over $MgSO_4$ and filtered before the solvent was removed by rotary evaporation to give the crude product. The crude product was purified by Kugelrohr distillation followed by the addition of a solution of HCl in diethyl ether (2 M, 2 mL). The solvent was then removed by rotary evaporation to yield the pure hydrochloride salt as a pale yellow solid (68 mg, 0.42 mmol, 42%). **^{1}H NMR** (400 MHz, Methanol-*d*$_4$) δ 4.53 (dd, *J* = 10.8, 2.3 Hz, 1H), 4.11 (ddd, *J* = 11.7, 4.8, 1.4 Hz, 1H), 3.99 (qd, *J* = 16.5, 2.6 Hz, 2H), 3.58 (td, *J* = 12.2, 2.3 Hz, 1H), 3.20 (t, *J* = 2.6 Hz, 2H), 1.96 (ddt, *J* = 12.2, 4.0, 2.0 Hz, 1H), 1.78 (tdd, *J* = 15.5, 9.1, 3.7 Hz, 1H), 1.62 (ddq, *J* = 13.5, 3.8, 1.8 Hz, 1H), 1.30 – 1.11 (m, 2H), 1.02 (d, *J* = 6.5 Hz, 3H). **^{13}C NMR** (101 MHz, Methanol-*d*$_4$) δ 85.21, 78.90, 74.56, 68.86, 37.84, 34.22, 33.96, 29.97, 21.99. ***m/z* (±ES)** 94.1 [M-C_3H_7]$^+$, 109.1 [M-C_2H_4]$^+$, 137.1 [M]$^+$.

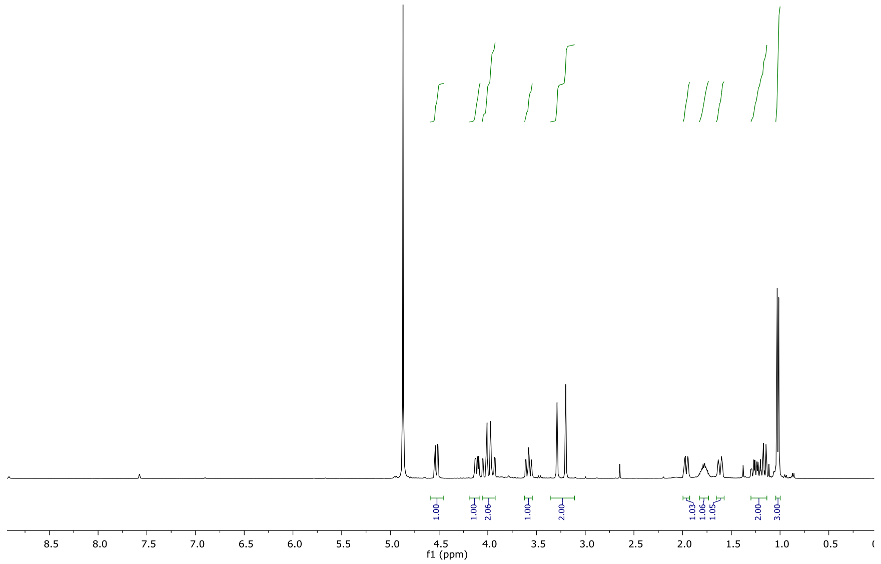

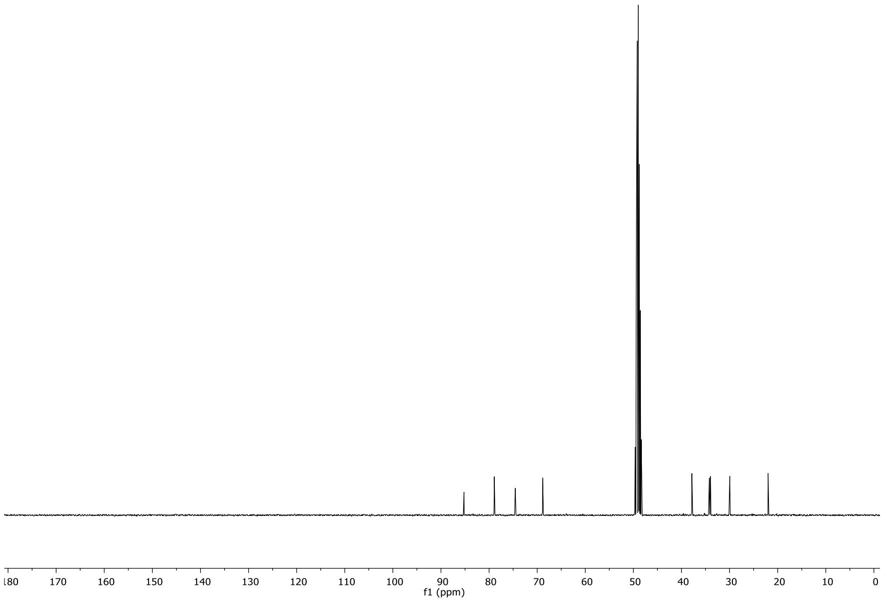

Synthesis of *N*-(3-chloropropyl)azepane hydrochloride (10vii)

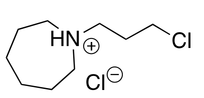

10vii
$C_9H_{18}ClN.HCl$
M.W.: 212.16

To a 500 mL glass bottle were added 1,6-hexanediol (1.0 mmol, 118 mg), $NADP^+$ (15 mg, 20 µmol), glucose (1.4 g, 7.7 mmol), *Ad*RedAm (60 mg, purified enzyme), *Ta*GDH (50 mg, cell free extract), $AcCO_6$ (1 g, cell free extract), DMSO (2 mL), a pH adjusted stock solution of 3-chloropropylamine (1 M in 100 mM pH 7.0 KPi buffer, 10 mL) and 100 mM pH 7.0 KPi buffer (88 mL). The bottle was sealed before incubation at 30 °C in an orbital shaker at 250 rpm for 18h. The reaction was decanted into falcon tubes and basified to a pH of 12.0 with 5 M NaOH solution before extraction into diethyl ether (2 x 4 x 20 mL) with intermediate centrifugation to improve the separation of phases (4 °C, 4000 rpm, 20 min). The combined organic extracts were dried over $MgSO_4$ and filtered before the solvent was removed by rotary evaporation to give the crude product. The crude product was purified by Kugelrohr distillation followed by the addition of a solution of HCl in diethyl ether (2 M, 2 mL). The solvent was then removed by rotary evaporation to yield the pure hydrochloride salt as a pale yellow solid (87 mg, 0.41 mmol, 41%).**^{1}H NMR** (400 MHz, methanol-d_4) δ 3.74 – 3.61 (m, 2H), 3.48 (ddd, J = 13.5, 7.3, 3.1 Hz, 2H), 3.33 – 3.27 (m, 3H), 3.21 (ddd, J = 13.5, 8.6, 3.0 Hz, 2H), 2.32 – 2.15 (m, 2H), 1.93 (tdd, J = 16.0, 9.5, 4.0 Hz, 4H), 1.77 – 1.65 (m, 4H). **^{13}C NMR** (101 MHz, Methanol-d_4) δ 56.42, 56.08, 42.36, 28.42, 27.30, 24.84. ***m/z* (±ES)** 112.1 $[M-C_2H_4Cl]^+$, 146.1 $[M-C_2H_5]^+$, 175.1 $[M]^+$.

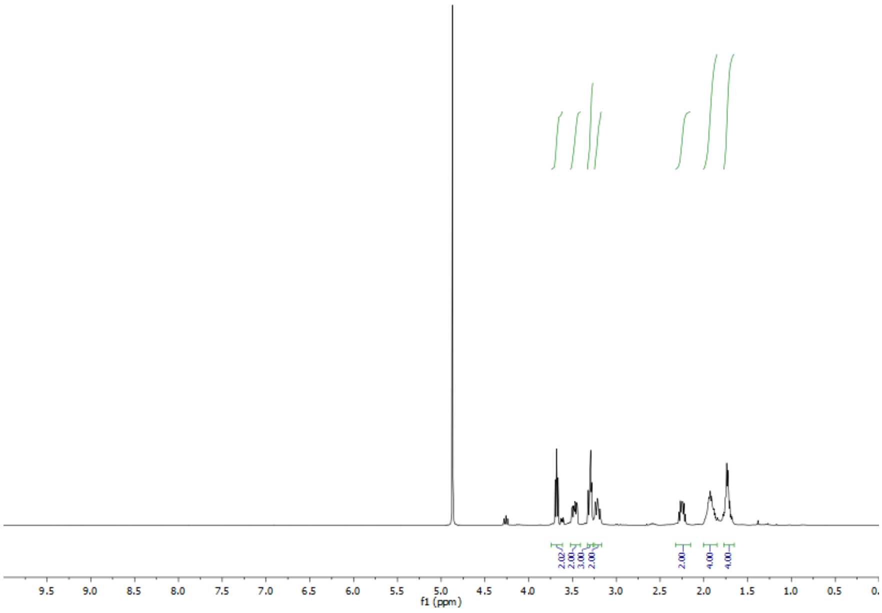

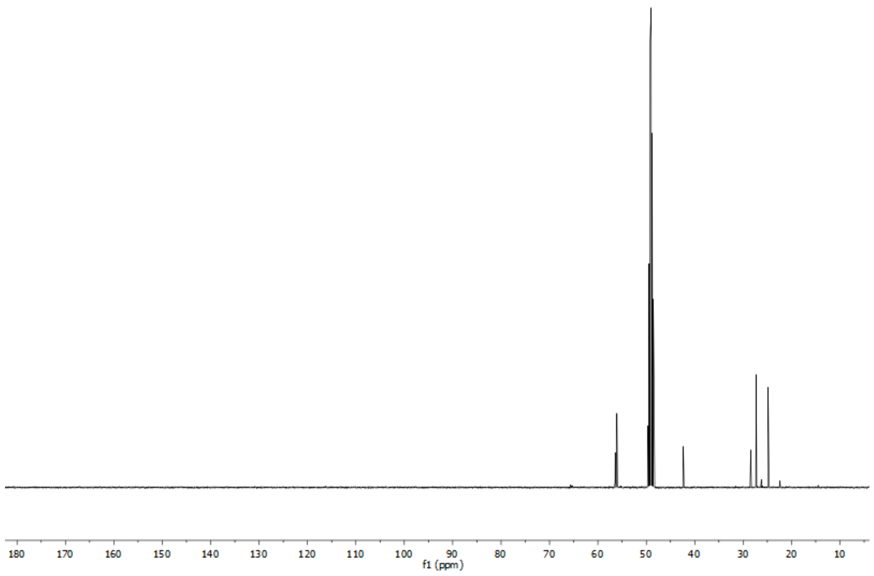

Synthesis of (S)-*N*-cyclopropyl-2-methylpiperidine hydrochloride (*S*-13i)

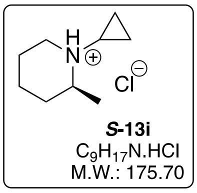

To a 500 mL glass bottle were added 5-oxohexanol (1.0 mmol, 116 mg), $NADP^+$ (15 mg, 20 µmol), glucose (1.4 g, 7.7 mmol), *Ad*RedAm (60 mg, purified enzyme), *Ta*GDH (50 mg, cell free extract), $AcCO_6$ (1 g, cell free extract), DMSO (2 mL), a pH adjusted stock solution of cyclopropylamine (1M in 100 mM pH 7.0 KPi buffer, 10 mL) and 100 mM pH 7.0 KPi buffer (88 mL). The bottle was sealed before incubation at 30 °C in an orbital shaker at 250 rpm for 18h. The reaction was decanted into falcon tubes and basified to a pH of 12.0 with 5M NaOH solution before extraction into diethyl ether (2 x 4 x 20 mL) with intermediate centrifugation to improve the separation of phases (4 °C, 4000 rpm, 20 min). The combined organic extracts were dried over $MgSO_4$ and filtered before the solvent was removed by rotary evaporation to give the crude product. The crude product was then purified by Kugelrohr distillation before the addition of 2M HCl in diethyl ether. The diethyl ever was then removed by rotatory evaporation to yield the final product as a yellow solid (119 mg, 0.68 mmol, 68%) *ee* 84%. **^{1}H NMR** (400 MHz, Methanol-*d*$_4$) δ 3.63 (ddt, *J* = 12.6, 4.1, 2.0 Hz, 1H), 3.45 – 3.38 (m, 1H), 3.14 (td, *J* = 12.7, 3.1 Hz, 1H), 2.74 (tt, *J* = 7.7, 4.3 Hz, 1H), 1.96 (ddq, *J* = 17.7, 6.5, 3.5 Hz, 2H), 1.87 – 1.78 (m, 2H), 1.70 – 1.58 (m, 2H), 1.53 (d, *J* = 6.6 Hz, 3H), 1.25 (dtd, *J* = 10.9, 6.7, 4.1 Hz, 1H), 1.12 (qd, *J* = 7.0, 2.2 Hz, 1H), 0.99 – 0.88 (m, 2H). **^{13}C NMR** (101 MHz, Methanol-*d*$_4$) δ 64.89, 56.35, 39.32, 32.88, 24.36, 23.03, 18.92, 8.08, 3.32. ***m/z* (±ES)** 97.1 $[M-C_3H_6]^+$, 124.1 $[M-CH_3]^+$, 139.1 $[M]^+$.

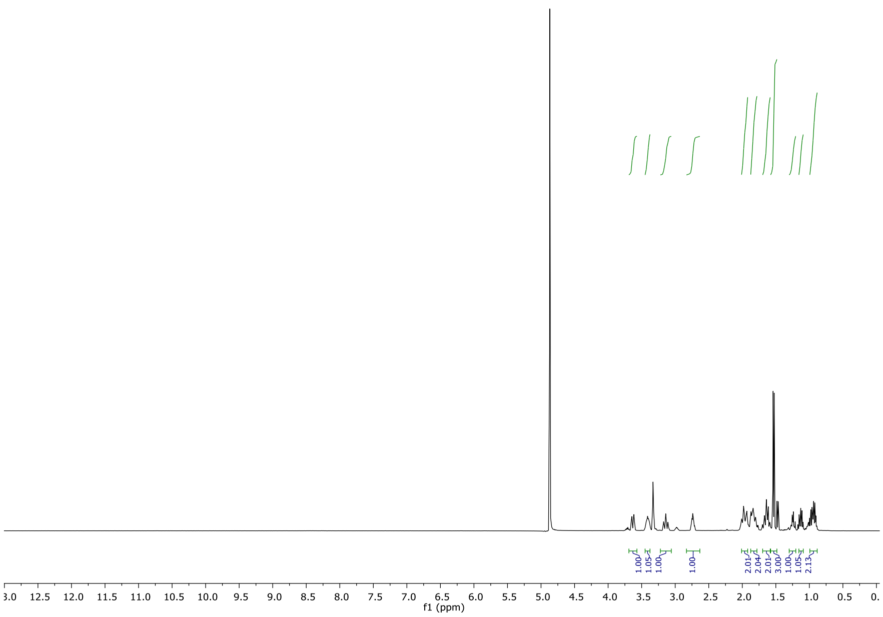

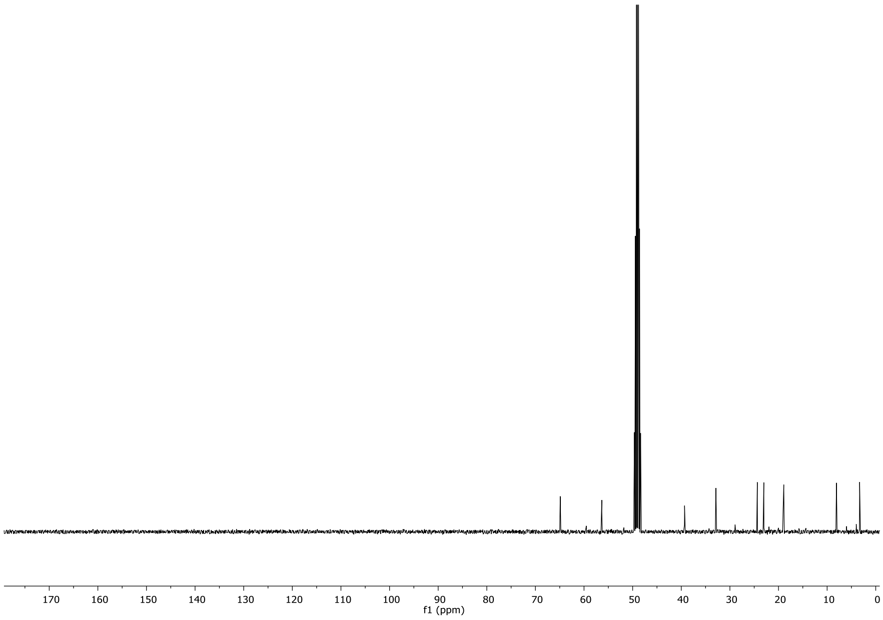

Synthesis of *N*-allyl-6-oxoheptanamine (14v)

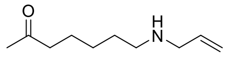

14v
$C_{10}H_{19}NO$
M.W.: 169.27

To a 500 mL glass bottle were added 6-oxoheptanol (1.0 mmol, 116 mg), $NADP^+$ (15 mg, 20 μmol), glucose (1.4 g, 7.7 mmol), *Ad*RedAm (60 mg, purified enzyme), *Ta*GDH (50 mg, cell free extract), $AcCO_6$ (1 g, cell free extract), DMSO (2 mL), a pH adjusted stock solution of allylamine (1 M in 100 mM pH 7.0 KPi buffer, 10 mL) and 100mM pH 7.0 KPi buffer (88 mL). The bottle was sealed before incubation at 30 °C in an orbital shaker at 180 rpm for 18h. The reaction was decanted into falcon tubes and basified to a pH of 12.0 with 5 M NaOH solution before extraction into diethyl ether (2 x 4 x 20 mL) with intermediate centrifugation to improve the separation of phases (4 °C, 4000 rpm, 20 min). The combined organic extracts were dried over $MgSO_4$ and filtered before the solvent was removed by rotary evaporation to give the crude product. The crude product was then purified by Kugelrohr distillation to yield the final product as a clear oil (101 mg, 0.60 mmol, 60%) **^{1}H NMR** (400 MHz, chloroform-*d*) δ 5.89 (ddt, *J* = 16.5, 10.2, 6.0 Hz, 1H), 5.16 (dq, *J* = 17.2, 1.7 Hz, 1H), 5.07 (dq, *J* = 10.2, 1.5 Hz, 1H), 3.23

(dt, J = 6.0, 1.5 Hz, 2H), 2.59 (t, J = 7.2 Hz, 2H), 2.42 (t, J = 7.4 Hz, 2H), 2.12 (s, 3H), 1.63 – 1.46 (m, 4H), 1.35 – 1.30 (m, 2H). **^{13}C NMR** (101 MHz, Chloroform-*d*) δ 209.25, 137.11, 115.88, 52.68, 49.33, 43.77, 30.05, 27.03, 23.80. ***m/z* (±ES)** 70.1 [M-$C_6H_{11}O$]$^+$, 169.1 [M]$^+$.

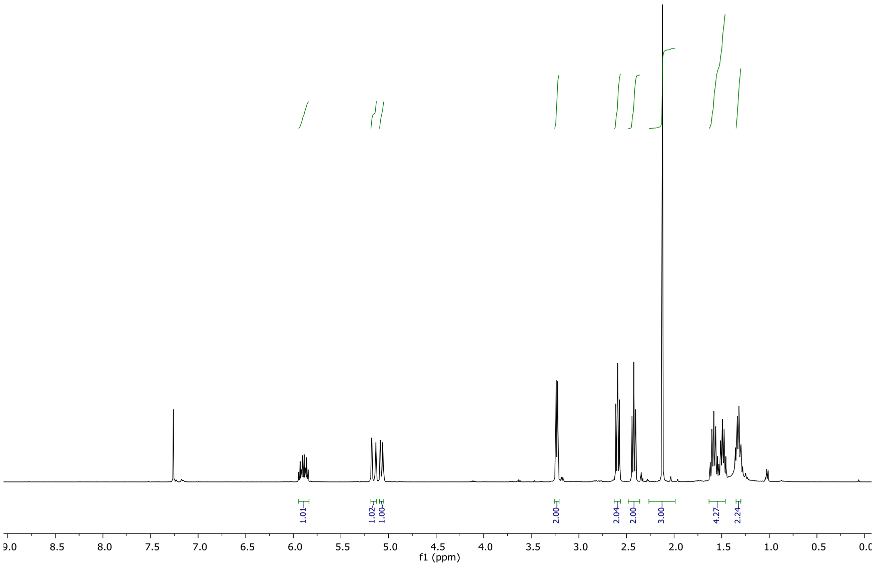

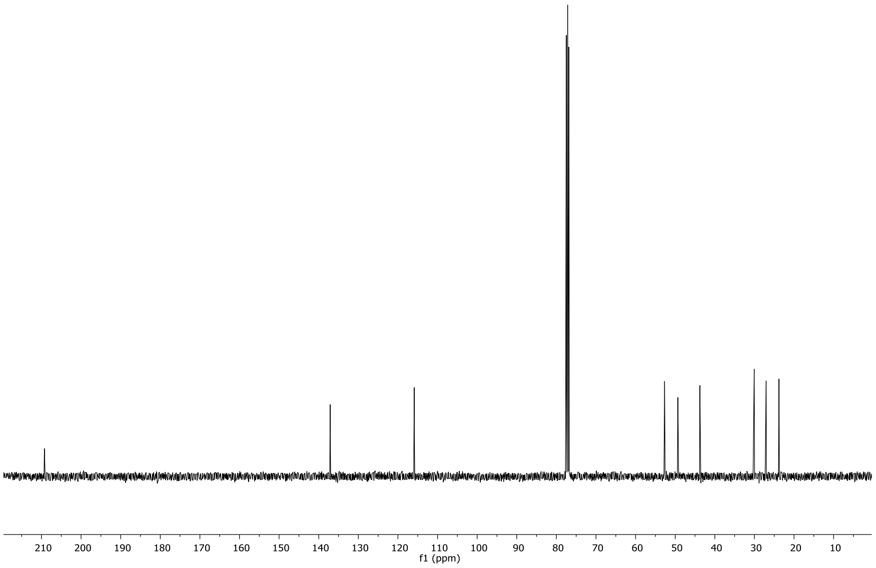

Synthesis of *N*-propargyl-2-phenylpiperidine (15vi)

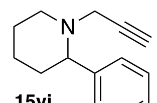

15vi
$C_{14}H_{17}N$
M.W.: 199.30

To a 500 mL glass bottle were added 4-benzoylbutanol (1.0 mmol, 178 mg), NADP$^+$ (15 mg, 20 µmol), glucose (1.4 g, 7.7 mmol), *Ad*RedAm (40 mg, purified enzyme), *Ta*GDH (50 mg, cell free extract), AcCO$_6$ (1 g, cell free extract), DMSO (2 mL), a pH adjusted stock solution of propargylamine (1 M in 100 mM pH 7.0 KPi buffer, 10 mL) and 100 mM pH 7.0 KPi buffer (88 mL). The bottle was sealed before incubation at 30 °C in an orbital shaker at 180 rpm for 24h. After 24h, ammonia borane (120 mg, 4 mmol) was added to the reaction, which was incubated for a further 18h. The reaction was decanted into falcon tubes and basified to a pH of 12.0 with 5 M NaOH solution before extraction into methyl *tert*-butyl ether (2 x 6 x 20 mL) with intermediate centrifugation to improve the separation of phases (4 °C, 4000 rpm, 20 min). The combined organic extracts were dried over $MgSO_4$ and filtered before the solvent was removed by rotary evaporation to give the crude product. The crude product was then purified

by Kugelrohr distillation (200 °C, 5 mbar) to yield the pure product as a clear oil (145 mg, 0.73 mmol, 73%). **^{1}H NMR** (400 MHz, Chloroform-*d*) δ 7.37 – 7.29 (m, 4H), 7.27 – 7.21 (m, 1H), 3.26 (dd, *J* = 11.1, 2.9 Hz, 1H), 3.23 – 3.04 (m, 2H), 3.03 – 2.95 (m, 1H), 2.56 (td, *J* = 11.4, 3.6 Hz, 1H), 2.19 (t, *J* = 2.4 Hz, 1H), 1.86 – 1.70 (m, 4H), 1.62 (tdd, *J* = 13.3, 11.1, 3.7 Hz, 1H), 1.46 – 1.31 (m, 1H). **^{13}C NMR** (101 MHz, Chloroform-*d*) δ 143.91, 128.60, 127.77, 127.35, 78.95, 73.20, 66.03, 53.19, 44.27, 35.78, 26.19, 25.01. ***m/z* (±ES)** 122.1 [M-C_6H_5]$^+$, 160.1 [M-C_3H_3]$^+$, 199.1 [M]$^+$.

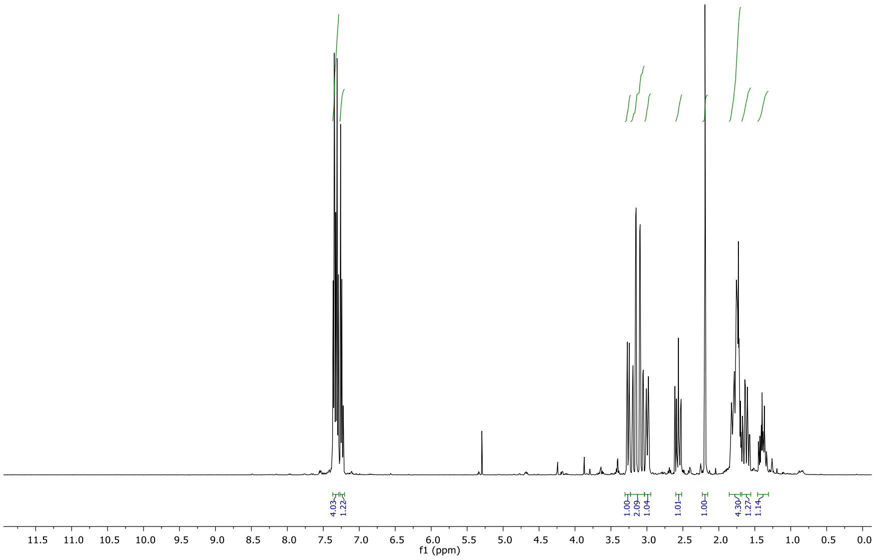

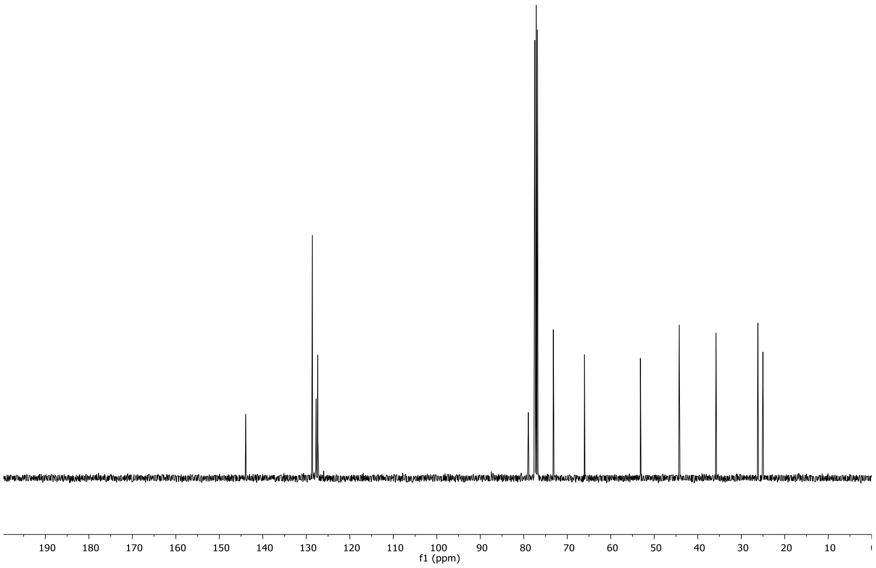

Synthesis of (*S*)-nicotine (20)

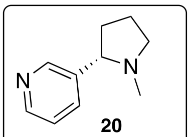

20
$C_{10}H_{14}N_2$
M.W.: 162.24

To a 500 mL glass bottle were added 3-pyridin-3-oylpropanol (1.0 mmol, 165 mg), $NADP^+$ (15 mg, 20 µmol), glucose (1.4 g, 7.7 mmol), pIRED-09 (40 mg, purified enzyme), *Ta*GDH (50 mg, cell free extract), $AcCO_6$ (1 g, cell free extract), DMSO (2 mL), a pH adjusted stock solution of methylamine (1 M in 100 mM pH 7.0 KPi buffer, 10 mL) and 100 mM pH 7.0 KPi buffer (88 mL). The bottle was sealed before incubation at 30 °C in an orbital shaker at 180 rpm for 24h. After 24h, a 50 mL stock containing 6-HDNO whole cells (3 g), ammonia borane (360 mg, 10 mmol) and catalase from bovine liver (15 mg, cell free extract) dissolved in 100 mM pH 7 KPi Buffer

was added to the reaction which was incubated for a further 18 h. The reaction was decanted into falcon tubes and basified to a pH of 12.0 with 5M NaOH solution before extraction into methyl tertiary butyl ether (2 x 6 x 20 mL) with intermediate centrifugation to improve the separation of phases (4 °C, 4000 rpm, 20 min). The combined organic extracts were dried over $MgSO_4$ and filtered before the solvent was removed by rotary evaporation to give the crude product. The crude product was then purified by flash column chromatography (DCM : MeOH, 90 : 10) to yield the pure product as a clear oil. (45 mg, 0.28 mmol, 28% yield, 99% *ee*). ^{1}**H NMR** (400 MHz, chloroform-*d*) δ 8.55 – 8.51 (m, 1H), 8.49 (dd, *J* = 4.9, 1.7 Hz, 1H), 7.70 (dt, *J* = 7.8, 2.0 Hz, 1H), 7.25 (ddd, *J* = 7.8, 4.8, 0.8 Hz, 1H), 3.25 (ddd, *J* = 9.7, 8.0, 2.1 Hz, 1H), 3.09 (t, *J* = 8.3 Hz, 1H), 2.31 (td, *J* = 9.3, 8.3 Hz, 1H), 2.23 – 2.15 (m, 4H), 2.02 – 1.91 (m, 1H), 1.88 – 1.78 (m, 1H), 1.73 (dddd, *J* = 12.6, 11.0, 8.3, 5.1 Hz, 1H). ^{13}**C NMR** (101 MHz, Chloroform-*d*) δ 149.59, 148.68, 138.67, 134.89, 123.62, 68.92, 57.03, 40.38, 35.18, 22.62. ***m/z*** **(±ES)** 84.1 $[M-C_5H_4N]^+$, 133.1 $[M-C_2H_5]^+$, 162.1 $[M]^+$.

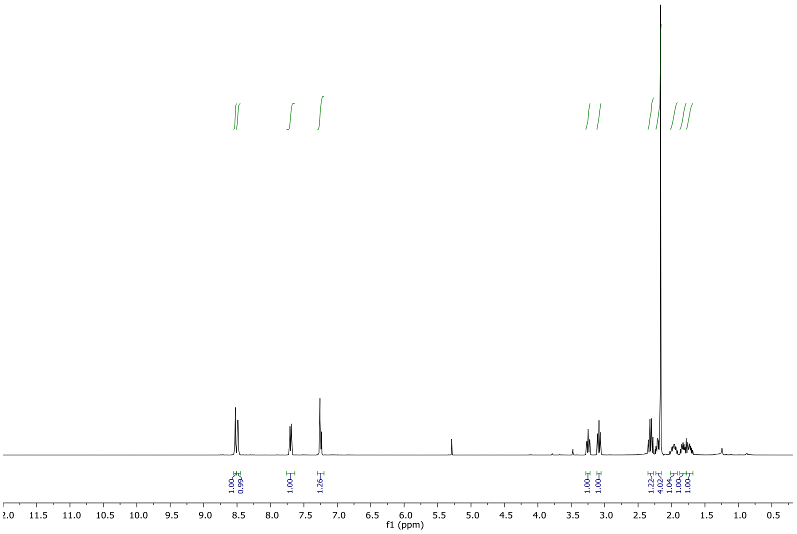

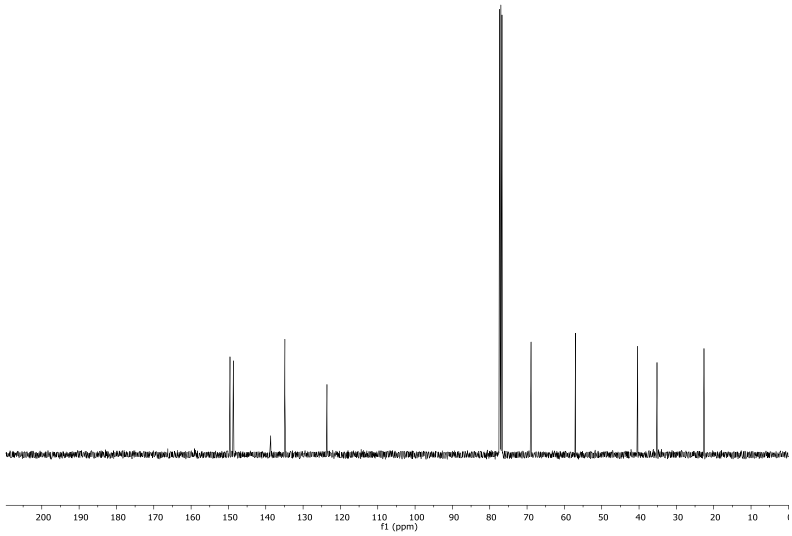

General Procedure 1: IRED-catalysed synthesis of *N*-alkylated 2,5-disubstituted pyrrolidines

N-alkylated 2,5-disubstituted pyrrolidines were synthesised *via* our IRED single-enzyme three-step cascade. Preparative scale reactions were performed in a 50 mL-scale in 250 mL flasks

containing the diketone substrate (**21** or **22**, 0.3 mmol), IRED (50 mg of purified enzyme or 250 mg of lyophilised cell-free extract), glucose dehydrogenase (CDX-901, 25 mg), $NADP^+$ (38.3 mg, 0.025 mmol), glucose (0.45 g, 2.5 mmol) and amine (5 mmol) in Tris buffer (100 mM, pH 9, 50 mL). Reactions were incubated at 80 rpm and 30 °C. After full conversion (24-72 h), reactions were quenched by adjusting the pH to >12 with 5 M NaOH, followed by extraction with MTBE (3 x 40 mL). The combined organic layers were dried over $MgSO_4$ anhydrous and the solvent was removed under reduced pressure to afford the desired pyrrolidines.

Reactions that have not shown full conversion after 72h or where the respective pyrrole was observed as a by-product were submitted to an alternative work-up protocol. First, the reaction was acidified to pH < 2 using concentrated HCl followed by extraction with MTBE (3 x 25 mL) in order to remove the remaining diketone and/or pyrrole. The extracted aqueous phase was then basified to pH > 12 using 5 M NaOH and extracted with MTBE (3 x 40 mL). The organic layers from the second step extraction were combined, dried over $MgSO_4$ anhydrous and the solvent was removed under reduced pressure to afford the desired pyrrolidines. When needed, crude products were cleaned using an Agilent Bond Elut SCX cation exchange column, following the manufacturer's protocol.

***cis*-(2S,5R)-N-cyclopropyl-2-methyl-5-pentylpyrrolidine (2S,5R-21i)**

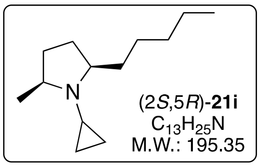

2,5-decadione (**21**, 50 mg, 0.3 mmol), cyclopropylamine (346 µL, 5 mmol) and IR49 (250 mg, lyophilised cell-free extract) were submitted to General Procedure 1 affording (2*S*,5*R*)-**21i** as a yellow oil in 66% yield (38 mg, 85:15 d.r., >99% ee). **^{1}H NMR** (400 MHz, chloroform-*d*) δ 2.57 – 2.46 (m, 1H), 2.45 – 2.35 (m, 1H), 1.97 – 1.82 (m, 2H), 1.81 – 1.70 (m, 2H), 1.39 – 1.17 (m, 9H), 1.11 (d, *J* = 6.3 Hz, 3H), 0.82 (t, *J* = 6.5 Hz, 3H), 0.44 – 0.34 (m, 4H). **^{13}C NMR** (101 MHz, Chloroform-*d*) δ 68.3, 63.2, 34.9, 34.6, 32.3, 31.3, 28.7, 26.6, 22.7, 20.4, 14.1, 4.6, 4.4. ***m/z* (±ES)** 124 $[M-C_5H_{11}]^+$, 195 $[M]^+$.

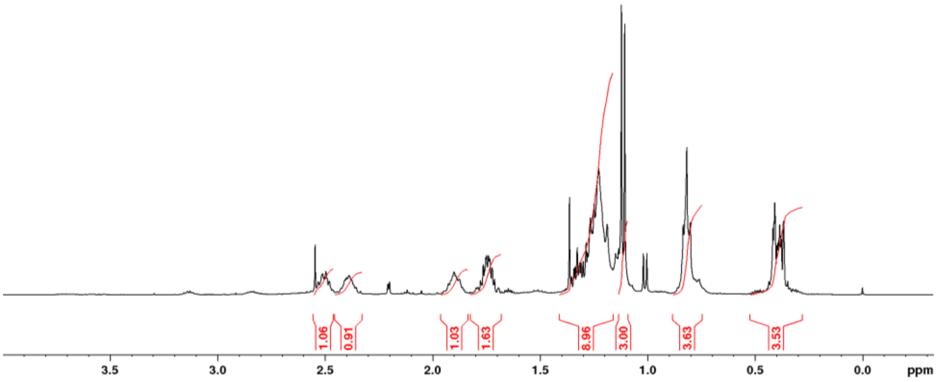

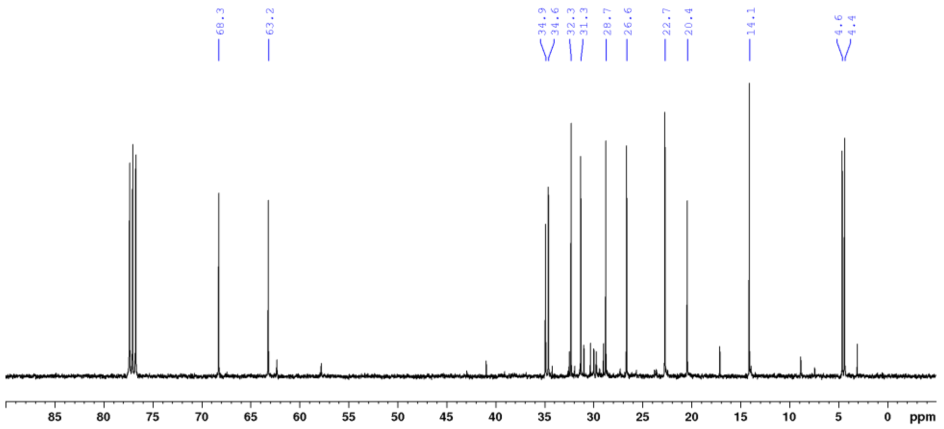

***trans*-(2S,5S)-N-cyclopropyl-2-methyl-5-pentylpyrrolidine (2S,5S-21i)**

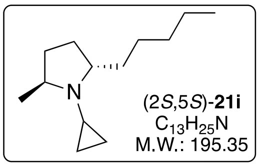

2,5-decadione (**21**, 50 mg, 0.3 mmol), cyclopropylamine (346 μL, 5 mmol) and IR23 (250 mg, lyophilised cell-free extract) were submitted to General Procedure 1 affording (2*S*,5*S*)-**21i** as a pale-yellow oil in 52% yield (30 mg, >99:1 d.r., >99% ee). **^{1}H NMR** (400 MHz, chloroform-*d*) δ 3,24 – 3.14 (m, 1H), 2.95 – 2.86 (m, 1H), 1.99 – 1.89 (m, 2H), 1.88 – 1.79 (m, 1H), 1.74 – 1.68 (m, 1H), 1.46 – 1.10 (m, 9H), 1.07 (d, *J* = 6.6 Hz, 3H), 0.88 (t, *J* = 6.7 Hz, 3H), 0.59 – 0.48 (m, 2H), 0.45 – 0.33 (m, 2H). **^{13}C NMR** (101 MHz, Chloroform-*d*) δ 62.3, 57.8, 32.5, 32.3, 31.0, 29.9, 29.0, 26.6, 22.7, 17.1, 14.1, 8.9, 3.1. ***m/z* (±ES)** 124 $[M\text{-}C_5H_{11}]^+$, 195 $[M]^+$.

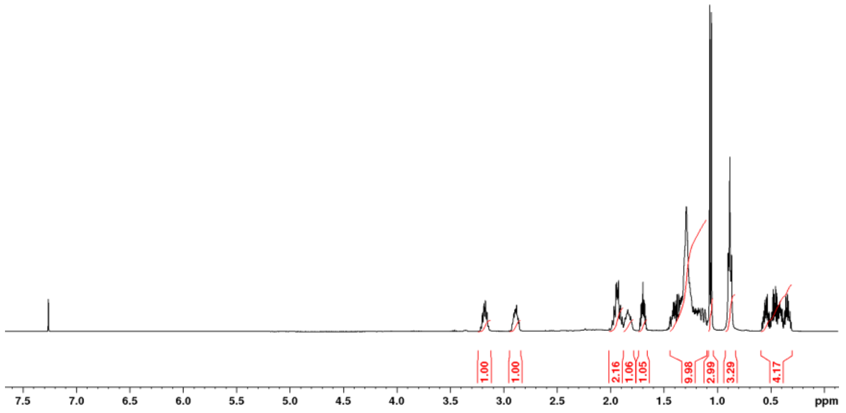

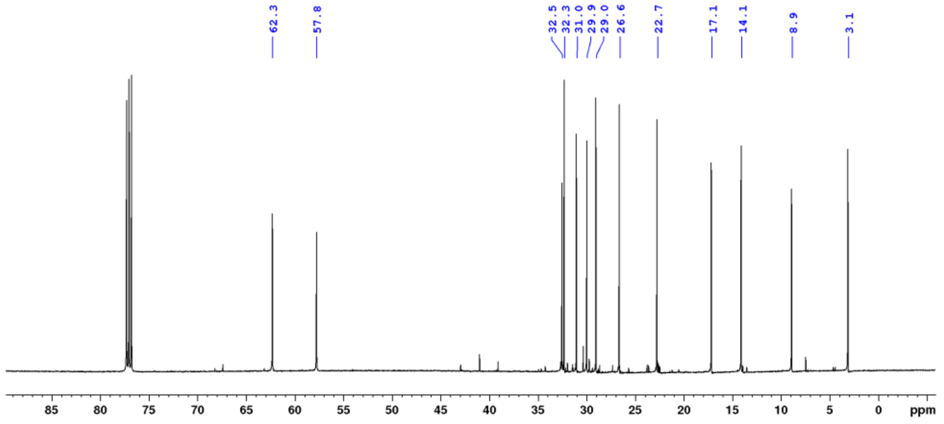

***cis*-(2*R*,5*R*)-*N*-cyclopropyl-2-methyl-5-phenylpyrrolidine (2*R*,5*R*-22i)**

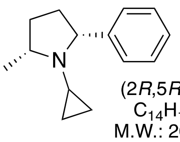

(2*R*,5*R*)-**22i**
$C_{14}H_{19}N$
M.W.: 201.31

1-phenylpentane-1,4-dione (**22**, 50 mg, 0.28 mmol), cyclopropylamine (346 µL, 5 mmol) and IR89 (50 mg, purified enzyme) were submitted to General Procedure 1 affording (2*R*,5*R*)-**22i** as a pale-yellow oil in 73% yield (42 mg, 97:3 d.r., 91% ee). **^{1}H NMR** (400 MHz, chloroform-*d*) δ 7.40 (d, *J* = 7.3 Hz, 2H), 7.33 (t, *J* = 7.3 Hz, 2H), 7.26 (t, *J* = 7.2 Hz, 1H), 3.71 (t, *J* = 8.0 Hz, 1H), 2.94 – 2.83 (m, 1H), 2.23 – 2.12 (m, 1H), 2.03 – 1.93 (m, 1H), 1.81 – 1.71 (m, 1H), 1.68 – 1.54 (m, 2H), 1.32 (d, *J* = 6.2 Hz, 3H), 0.48 – 0.35 (m, 2H), 0.04 – (-0.05) (m, 1H), (-0.2) – (-0.3) (m, 1H). **^{13}C NMR** (101 MHz, Chloroform-*d*) δ 146.3, 127.9, 126.5, 71.6, 62.9, 35.2, 33.6, 32.6, 20.8, 5.4, 3.4. ***m/z* (±ES)** 186 [M-CH_3]$^+$, 200 [M-H]$^+$, 201 [M]$^+$.

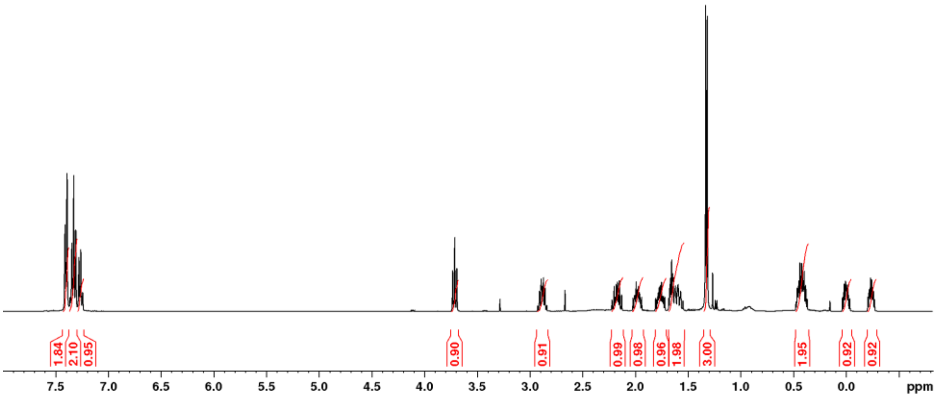

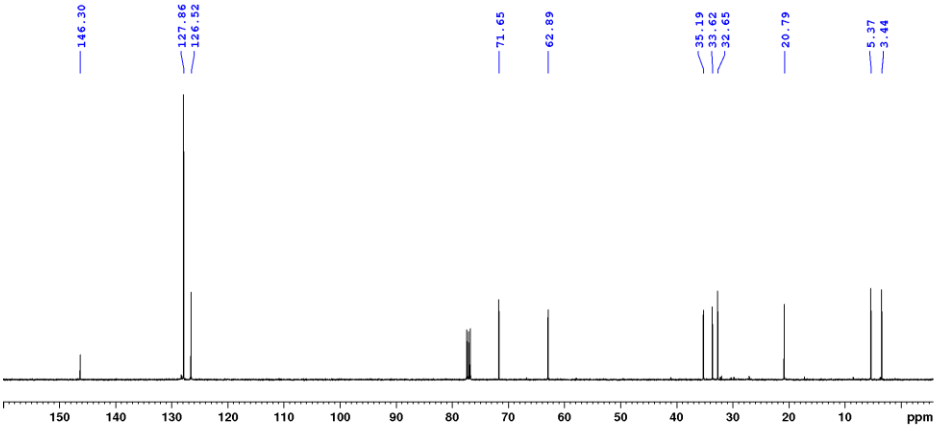

***trans*-(2*S*,5*R*)-*N*-cyclopropyl-2-methyl-5-phenylpyrrolidine (2*S*,5*R*-22i)**

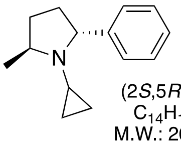

(2*S*,5*R*)-**22i**
$C_{14}H_{19}N$
M.W.: 201.31

1-phenylpentane-1,4-dione (**22**, 50 mg, 0.28 mmol), cyclopropylamine (346 µL, 5 mmol) and IR63 (50 mg, purified enzyme) were submitted to General Procedure 1 affording (2*S*,5*R*)-**22i** as a yellow oil in 49% yield (28 mg, 98:2 d.r., >99% ee). **^{1}H NMR** (400 MHz, chloroform-*d*) δ 7.40 – 7.24 (m, 5H), 4.10 (dd, *J* = 8.4, 5.0 Hz, 1H), 3.42 (sext, J = 6.4 Hz, 1H), 2.46 – 2.33 (m, 1H), 2.31 – 2.19 (m, 1H), 1.96 – 1.84 (m, 1H), 1.68 – 1.51 (m, 2H), 1.22 (d, *J* = 6.4 Hz, 3H), 0.44 – 0.14 (m, 4H). **^{13}C NMR** (101 MHz, Chloroform-*d*) δ 144.5, 128.3, 127.8, 126.7, 66.7, 57.9, 32.2, 31.9, 30.3, 17.2, 8.5, 3.8. ***m/z* (±ES)** 186 [M-CH_3]$^+$, 200 [M-H]$^+$, 201 [M]$^+$.

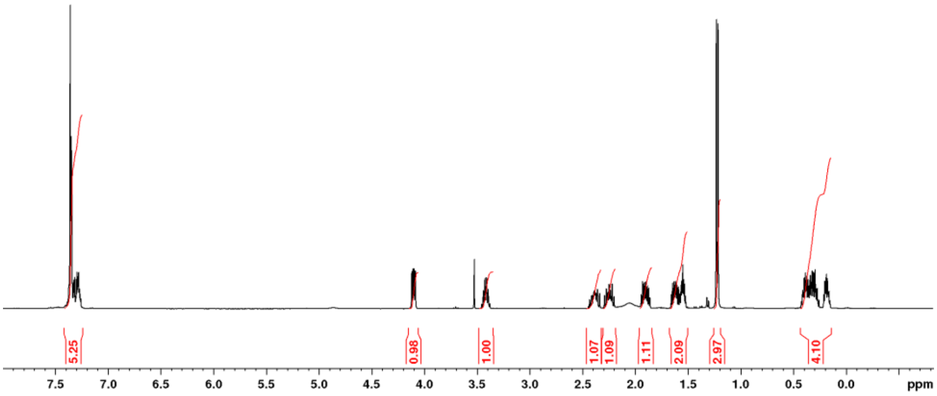

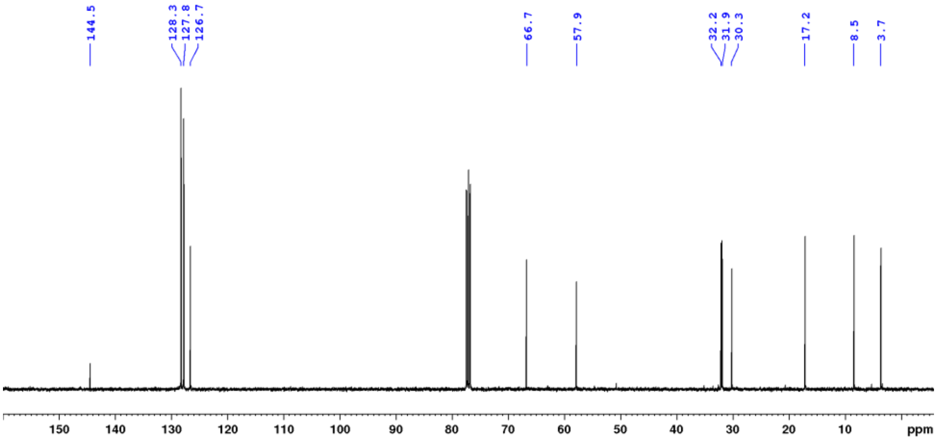

***trans*-(2S,5R)-*N*-methyl-2-methyl-5-phenylpyrrolidine (2S,5R-22iii)**

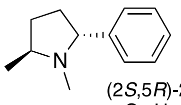

(2*S*,5*R*)-**22iii**
$C_{12}H_{17}N$
M.W.: 175.28

1-phenylpentane-1,4-dione (**22**, 50 mg, 0.28 mmol), methylamine hydrochloride (338 mg, 5 mmol) and IR23 (250 mg, lyophilised cell-free extract) were submitted to General Procedure 1 affording (2*S*,5*R*)-**22iii** as a yellow oil in 81% yield (28 mg, 91:9 d.r., >99% ee). **^{1}H NMR** (400 MHz, chloroform-*d*) δ 7.27 – 7.13 (m, 5H), 3.63 (dd, *J* = 8.1, 6.6 Hz, 1H), 3.33 – 3.25 (m, 1H), 2.29 – 2.10 (m, 2H), 2.79 (s, 3H), 1.76 – 1.67 (m, 1H), 1.52 – 1.42 (m, 1H), 0.97 (d, *J* = 6.5 Hz, 3H). **^{13}C NMR** (101 MHz, Chloroform-*d*) δ 142.7, 127.2, 126.8, 125.9, 66.0, 57.4, 34.0, 31.9, 30.7, 13.9. ***m/z* (±ES)** 98 $[M-C_6H_5]^+$, 160 $[M-CH_3]^+$, 175 $[M]^+$.

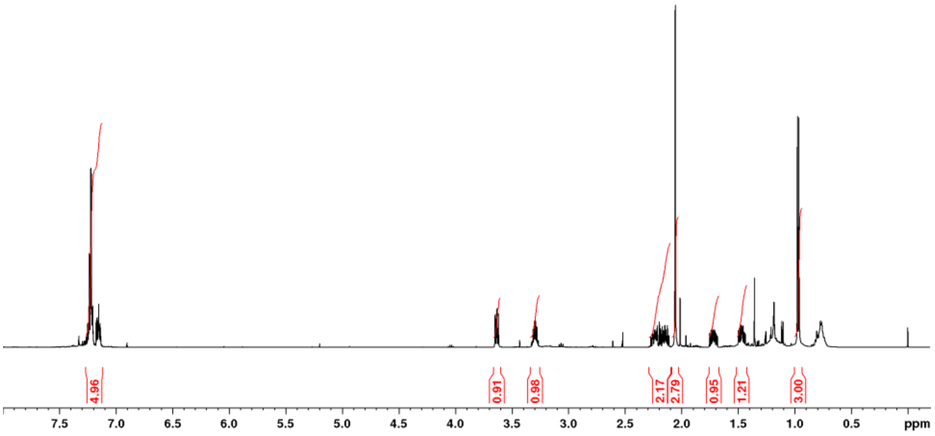

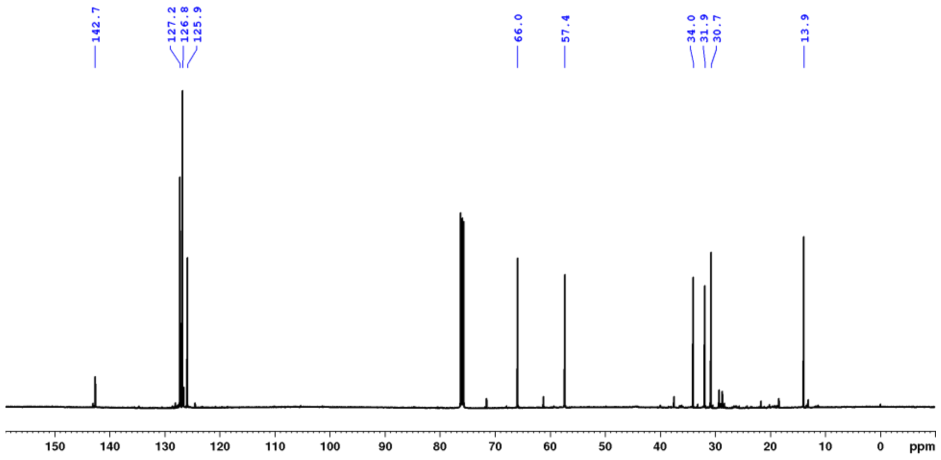

***cis*-(2*R*,5*R*)-*N*-propargyl-2-methyl-5-phenylpyrrolidine (2*R*,5*R*-22vi)**

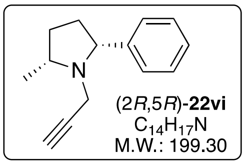

1-phenylpentane-1,4-dione (**22**, 50 mg, 0.28 mmol), propargylamine hydrochloride (458 mg, 5 mmol) and IR9 (250 mg, lyophilised cell-free extract) were submitted to General Procedure 1 affording (2*R*,5*R*)-**22vi** as a yellow oil in 51% yield (29 mg, 96:4 d.r., 95% ee). **^{1}H NMR** (400 MHz, chloroform-*d*) δ 7.29 (d, *J* = 7.3 Hz, 2H), 7.23 (t, *J* = 7.3 Hz, 2H), 7.15 (t, *J* = 7.3 Hz, 1H), 3.68 (t, *J* = 7.9 Hz, 1H), 3.38 (dd, *J* = 17.7, 2.2 Hz, 1H), 3.10 (dd, *J* = 17.7, 2.2 Hz, 1H), 2.87 (sext, *J* = 6.3 Hz, 1H), 2.06 (t, *J* = 2.2 Hz, 1H), 2.04 – 1.94 (m, 1H), 1.94 – 1.84 (m, 1H), 1.71 – 1.56 (m, 1H), 1.52 – 1.39 (m, 1H), 1.08 (d, *J* = 6.3 Hz, 3H). **^{13}C NMR** (101 MHz, Chloroform-*d*) δ 143.5, 128.4, 127.7, 127.1, 78.3, 72.6, 66.2, 56.0, 37.0, 32.7, 31.6, 19.2. ***m/z* (±ES)** 122 [M-C_6H_5]$^+$, 184 [M-CH_3]$^+$, 199 [M]$^+$.

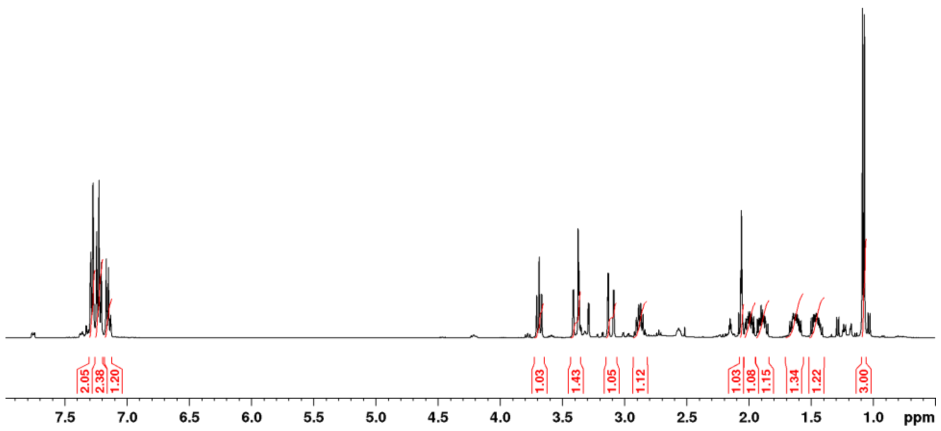

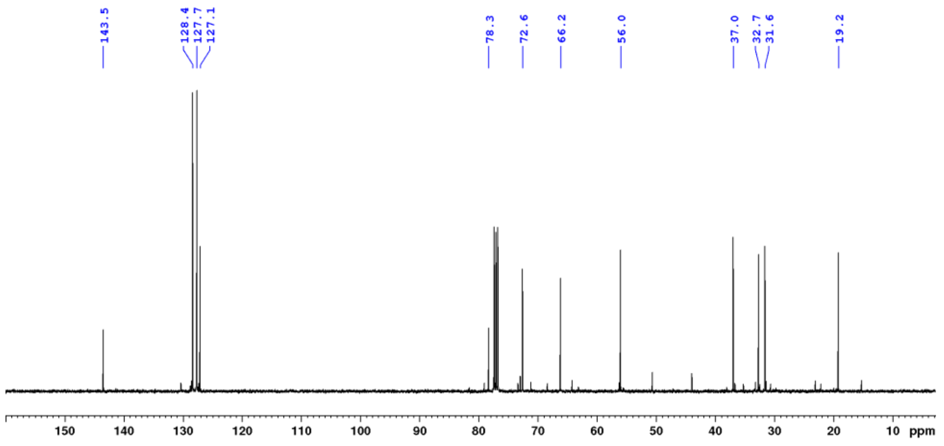

Synthesis of Substrates and Chemical Standards

Synthesis of *N*-Boc-6-oxo-heptan-1-amine

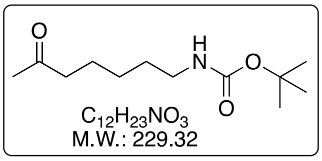

Under an atmosphere of N_2, *N*-Boc-ε-caprolactam (1.81g, 8.5 mmol) was dissolved in 2-methyltetrohydrofuran (40 mL) and stirred. The solution was cooled to -78 °C and methylmagnesium bromide (1.4 M in THF:toluene 1:3, 8.49 mL, 11.9 mmol) was added dropwise. The solution was stirred overnight at -78 °C before being allowed to reach room temperature. The reaction was quenched with the addition of saturated ammonium chloride solution before extraction with dichloromethane (3 x 50 mL). The combined organic phases were dried over $MgSO_4$ before concentration under reduced pressure to yield the crude product. The crude product was purified using a BioTage Isolera™ system running a solvent gradient of 90:10 *n*-heptane:ethyl acetate to 70:30 *n*-heptane:ethyl acetate yielding the pure product as a clear oil (870 mg, 45% yield).[2] **^{1}H NMR** (500 MHz, Chloroform-*d*) δ 4.53 (s, 1H), 3.10 (q, *J* = 6.7 Hz, 2H), 2.42 (t, *J* = 7.3 Hz, 2H), 2.12 (s, 3H), 1.57 (p, *J* = 7.4 Hz, 2H), 1.51 – 1.37 (m, 11H), 1.37 – 1.21 (m, 2H). **^{13}C NMR** (126 MHz, Chloroform-*d*) δ 209.11, 156.11, 79.20, 43.68, 40.50, 30.03, 28.55, 26.44, 23.51. **HRMS** calcd. for $C_{12}H_{23}O_3N$ 252.1576 $[M+Na]^+$, found 252.1573.

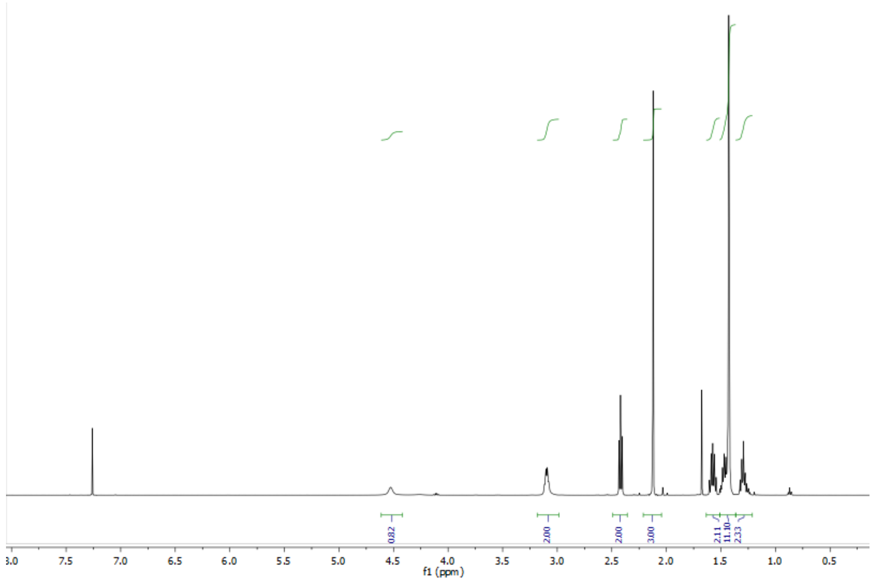

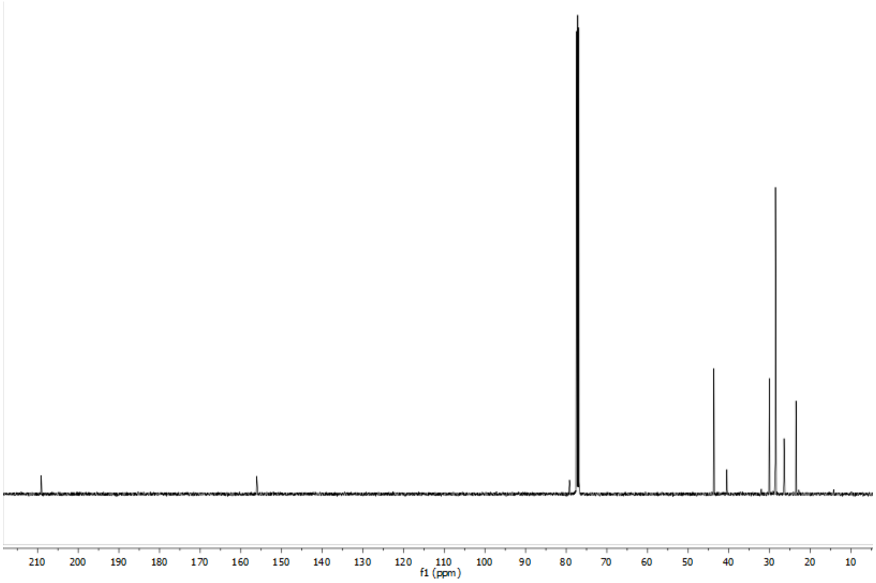

Synthesis of 2-Methylazepanium Chloride

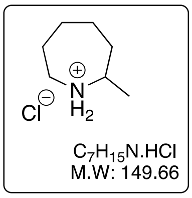

N-Boc-6-oxo-heptan-1-amine (1 g, 4.36 mmol) was dissolved in trifluoroacetic acid (6 mL) and stirred at room temperature for 4 hours. The trifluoroacetic acid was then removed by rotary evaporation to yield the crude iminium trifluoroacetate salt. Without purification, the iminium salt was dissolved in methanol (20 mL) followed by the portion-wise addition of sodium cyanoborohydride (628 mg, 10 mmol). The reaction was stirred overnight and then quenched with 1 M HCl. The methanol was removed by rotary evaporation (250 mbar, 40 °C) and the resulting aqueous solution was basified by the addition of 5 M NaOH. The basified solution was then extracted using DCM (3 x 20 mL) and the combined organic extracts were dried over $MgSO_4$, filtered and concentrated by rotary evaporation to yield the crude product. The crude product was then purified by Kugelrohr distillation (160 °C, 100 mbar) and the resulting distillate was dissolved in diethyl ether (10 mL) before the addition of 2M HCl in diethyl ether. The resulting salt was then dried by rotary evaporation to yield the final product as a white solid (384 mg, 59%). **^{1}H NMR** (400 MHz, Methanol-d_4) δ 3.43 (dqd, *J* = 9.8, 6.7, 3.2 Hz, 1H), 3.26 (ddd, *J* = 13.8, 7.0, 4.0 Hz, 1H), 3.16 (ddd, *J* = 13.8, 7.8, 4.0 Hz, 1H), 2.03 – 1.75 (m, 5H), 1.66 (dddd, *J* = 18.8, 9.3, 5.3, 2.4 Hz, 3H), 1.36 (d, *J* = 6.7 Hz, 3H). **^{13}C NMR** (101 MHz, Methanol-d_4) δ 56.36, 46.25, 34.26, 27.44, 25.98, 25.61, 20.50. ***m/z* (±ES)** (freebase) 98.1 $[M\text{-}CH_3]^+$, 113.1 $[M]^+$.

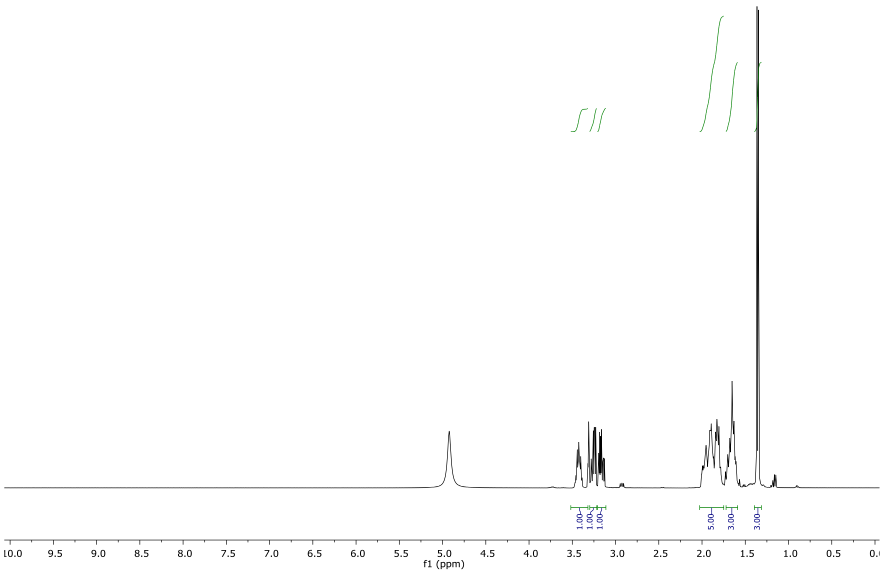

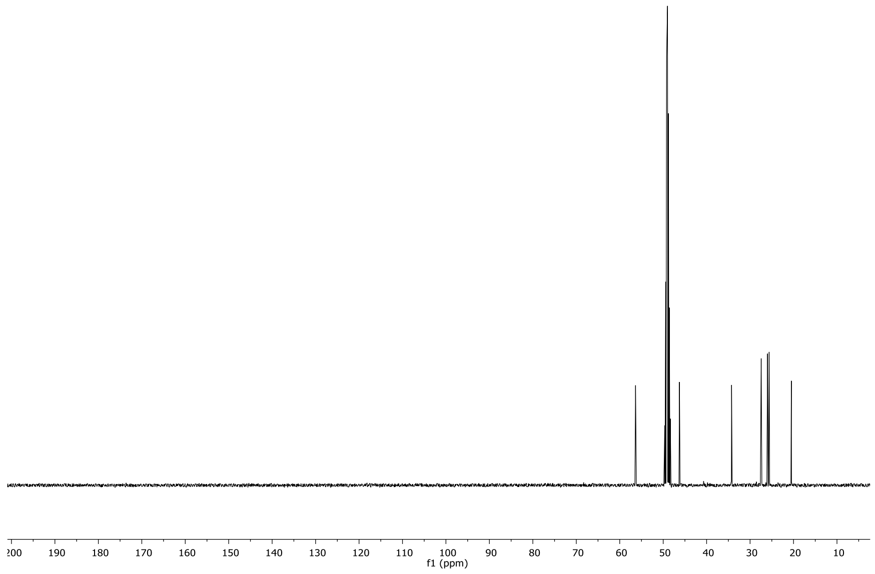

General procedure 2: *N*-alkylation of amine heterocycles

To a stirring solution of 2-methyltetrohydrofuran (20 mL) under an atmosphere of N_2 at room temperature was added sodium hydride (60% dispersion in mineral oil, 1 g, 25 mmol). To this suspension, amine heterocycle (10 mmol) was added dropwise before stirring for 1h. Following this, alkyl bromide reagent (12 mmol) was added dropwise followed by stirring overnight. The reaction was cooled to 0 °C and quenched by the dropwise addition of 2 M HCl (15 mL). The layers were separated using a separating funnel before the pH of the aqueous layer was adjusted to 10 by the addition of potassium carbonate. The aqueous layer was then extracted using DCM (3 x 20 mL) and the resulting organic extracts were dried over $MgSO_4$, filtered and concentrated by rotary evaporation to yield the crude product. For the *N*-alkylation of 2-methylazepane, ratios were unchanged, but reactions were performed on a 1.5 mmol scale.

N-*allyl-2-methylpiperidine (13v)*

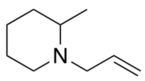

13v
$C_9H_{17}N$
M.W.:139.24

The crude product was purified by Kugelrohr distillation (200 °C, 100 mbar) to isolate the pure product as a clear oil (1.06 g, 76%) **^{1}H NMR** (400 MHz, Chloroform-*d*) δ 6.02 – 5.83 (m, 1H), 5.23 – 5.05 (m, 2H), 3.40 (ddt, *J* = 13.9, 5.6, 1.6 Hz, 1H), 3.03 – 2.80 (m, 2H), 2.31 – 2.05 (m, 2H), 1.73 – 1.48 (m, 4H), 1.37 – 1.21 (m, 2H), 1.10 (d, *J* = 6.2 Hz, 3H). **^{13}C NMR** (101 MHz, Chloroform-d) δ 135.21, 117.54, 57.53, 56.10, 52.47, 34.91, 26.28, 24.29, 19.41. ***m/z* (±ES)** 96.1 [M-C_3H_7]$^+$, 124.1 [M-CH_3]$^+$, 139.1 [M]$^+$.

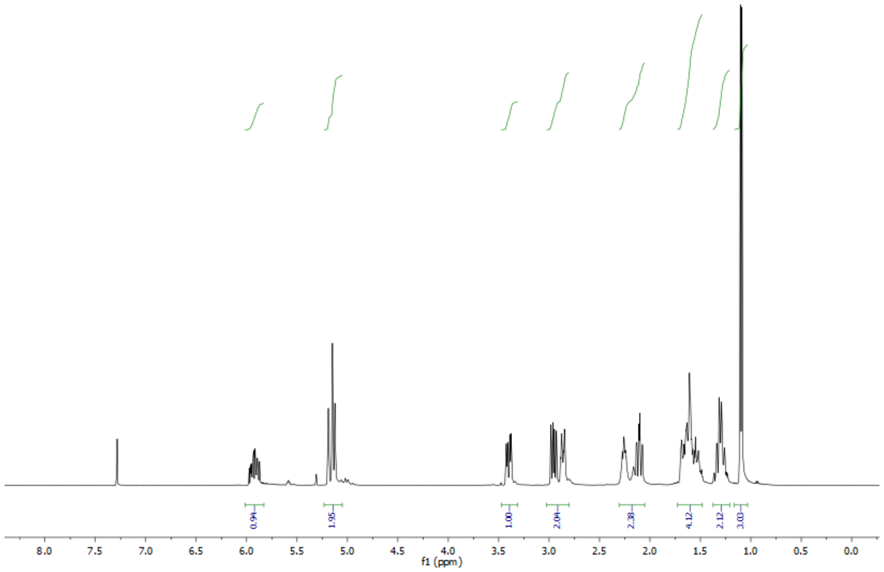

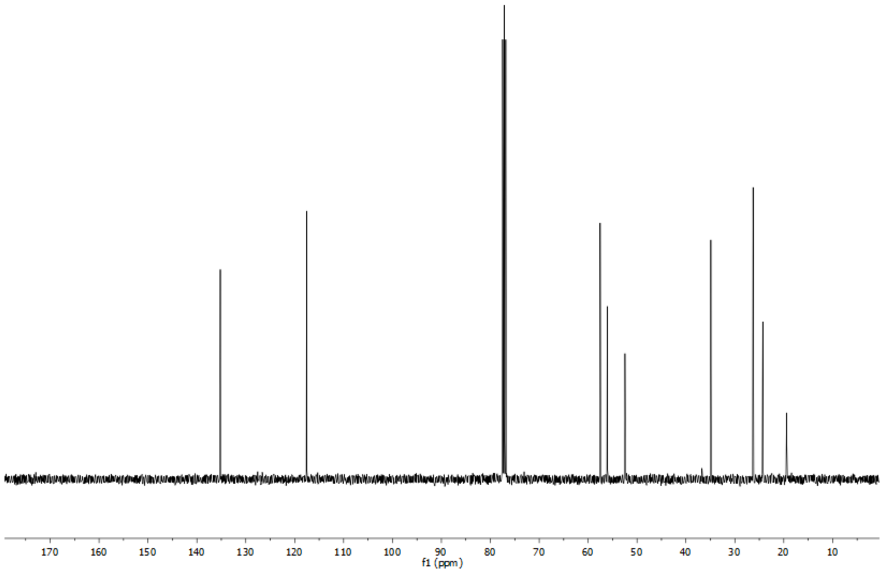

(S)-N-*allyl-2-methylpiperidine* (S-*13v*)

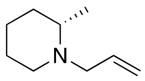

(*S*)-13v
$C_9H_{17}N$
M.W.:139.24

Enantiomerically pure (*S*)-2-methylpiperidine was applied as the starting reagent. The crude product was purified by Kugelrohr distillation (200 °C, 100 mbar) to yield the pure product as a clear oil (947 mg, 68%). **^{1}H NMR** (400 MHz, Chloroform-*d*) δ 5.90 (dddd, *J* = 17.1, 10.1, 7.9, 5.6 Hz, 1H), 5.33 – 4.94 (m, 2H), 3.38 (ddt, *J* = 13.9, 5.5, 1.6 Hz, 1H), 2.93 (ddt, *J* = 13.9, 7.9, 1.0 Hz, 1H), 2.84 (dtd, *J* = 11.6, 3.9, 1.3 Hz, 1H), 2.23 (dqd, *J* = 9.0, 6.1, 2.9 Hz, 1H), 2.08 (td, *J* = 11.3, 3.1 Hz, 1H), 1.68 – 1.46 (m, 4H), 1.34 – 1.22 (m, 2H), 1.08 (d, *J* = 6.2 Hz, 3H). **^{13}C NMR**

(101 MHz, Chloroform-*d*) δ 135.20, 117.57, 57.55, 56.10, 52.48, 34.92, 26.30, 24.31, 19.45. ***m/z* (±ES)** 96.1 [M-C_3H_7]$^+$, 124.1 [M-CH_3]$^+$, 139.1 [M]$^+$.

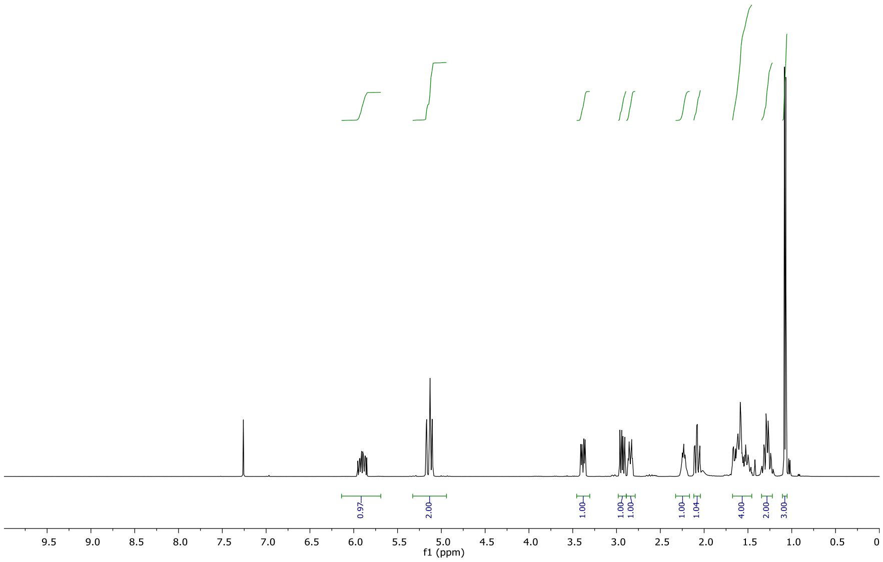

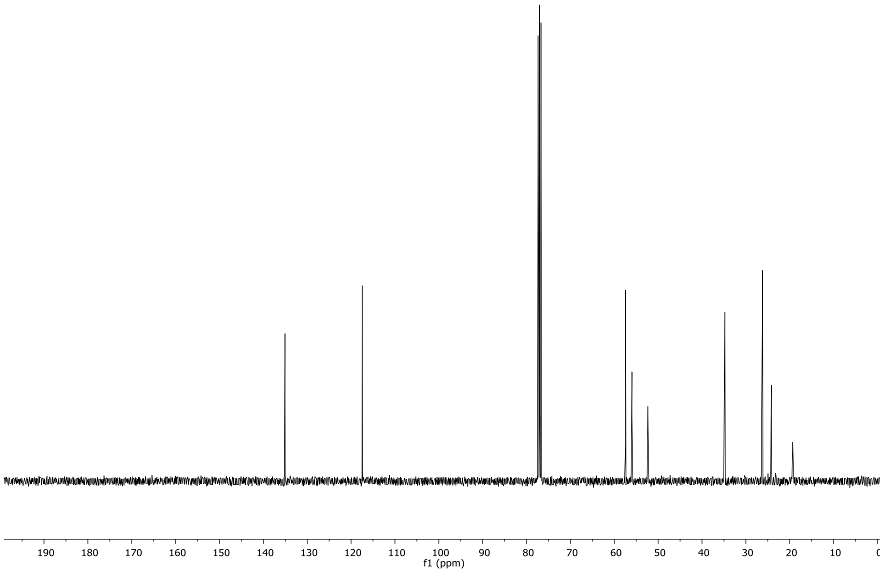

N-*propargyl-2-methylpiperidine (13vi)*

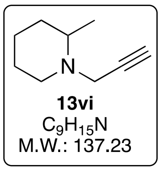

The crude product was purified by Kugelrohr distillation (180 °C, 100 mbar) to yield the pure product as a clear oil (1.11 g, 81%)**^{1}H NMR** (400 MHz, Chloroform-*d*) δ 3.66 (dd, *J* = 17.4, 2.4 Hz, 1H), 3.31 (dd, *J* = 17.4, 2.4 Hz, 1H), 2.87 – 2.63 (m, 1H), 2.45 (td, *J* = 11.6, 2.9 Hz, 1H), 2.40 – 2.29 (m, 1H), 2.18 (t, *J* = 2.4 Hz, 1H), 1.72 – 1.48 (m, 4H), 1.36 – 1.16 (m, 2H), 1.07 (d, *J* = 6.2 Hz, 3H). **^{13}C NMR** (101 MHz, Chloroform-*d*) δ 78.42, 72.97, 54.65, 53.38, 43.41, 34.87, 26.43, 24.71, 20.15. **HRMS** calcd. for $C_9H_{16}N$ 138.1277 [M+H]$^+$, found 138.1283.

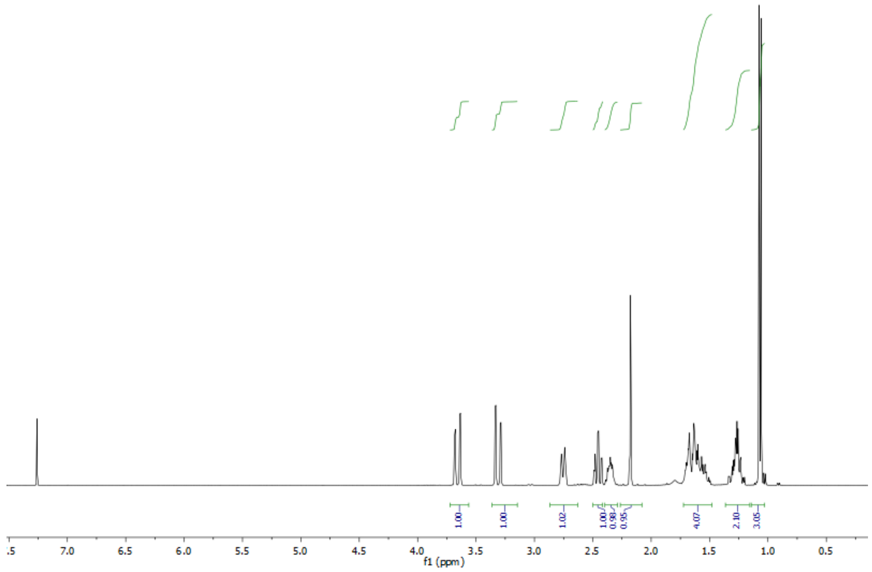

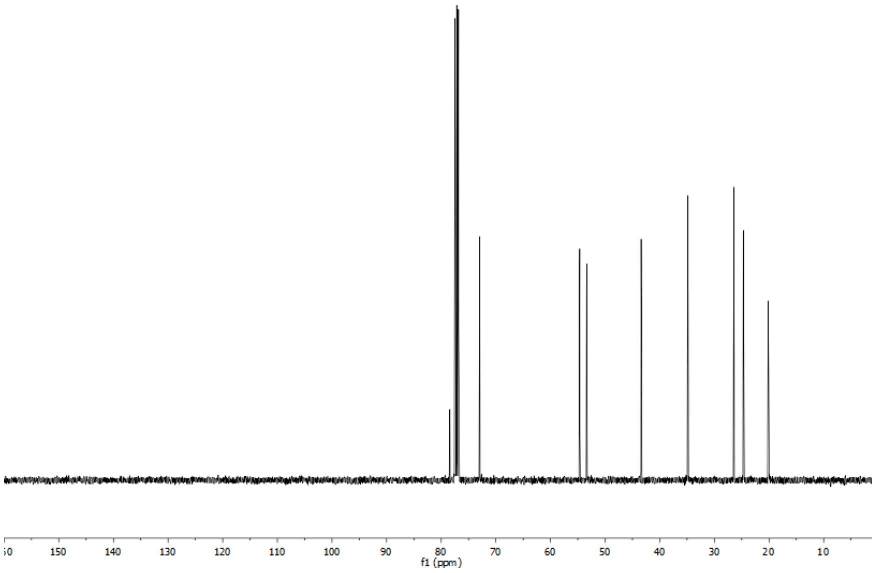

N-*allyl-2-phenylpiperidine (15v)*

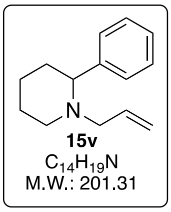

The crude product was purified using a BioTage Isolera™ system running a solvent gradient of 90:10 *n*-heptane:ethyl acetate to 70:30 *n*-heptane:ethyl acetate yielding the pure product as a clear oil (1.691 g, 84% yield). **^{1}H NMR** (500 MHz, Chloroform-*d*) δ 7.41 – 7.27 (m, 4H), 7.25 – 7.18 (m, 1H), 5.79 (dddd, *J* = 16.7, 10.6, 8.2, 4.9 Hz, 1H), 5.16 – 4.90 (m, 2H), 3.14 (ddt, *J* = 13.8, 4.8, 1.8 Hz, 2H), 3.02 (dd, *J* = 11.1, 2.9 Hz, 1H), 2.47 (ddt, *J* = 13.9, 8.2, 0.9 Hz, 1H), 2.03 (td, *J* = 11.8, 3.2 Hz, 1H), 1.84 – 1.51 (m, 5H), 1.36 (qt, *J* = 12.8, 4.3 Hz, 1H). **^{13}C NMR (**126 MHz, Chloroform-*d*) δ 145.25, 135.72, 128.52, 127.61, 126.99, 117.16, 68.91, 58.69, 53.53, 36.77, 26.17, 25.31. **HRMS** calcd. for $C_{14}H_{20}N$ 202.1590 $[M+H]^{+}$, found 202.1591.

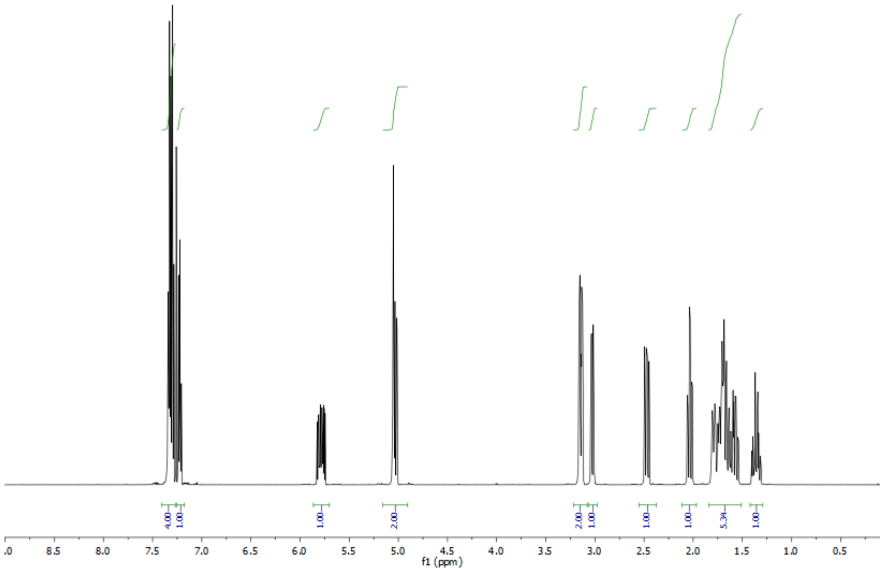

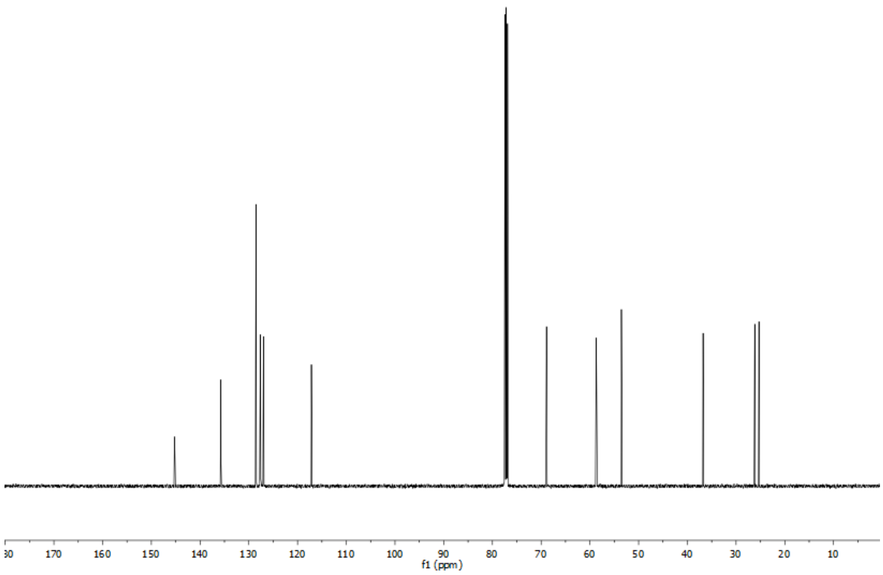

N-*propargyl-2-phenylpiperidine (15vi)*

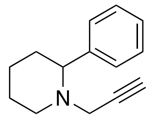

15vi
$C_{14}H_{17}N$
M.W.:199.30

The crude product was purified using a BioTage Isolera™ system running a solvent gradient of 90:10 *n*-heptane:ethyl acetate to 70:30 *n*-heptane:ethyl acetate yielding the pure product as a clear oil (1.554 g, 78% yield). **^{1}H NMR** (400 MHz, Chloroform-*d*) δ 7.40 – 7.27 (m, 4H), 7.27 – 7.21 (m, 1H), 3.26 (dd, *J* = 11.1, 2.9 Hz, 1H), 3.22 – 3.04 (m, 2H), 3.00 (ddt, *J* = 11.5, 3.1, 1.6 Hz, 1H), 2.56 (td, *J* = 11.4, 3.6 Hz, 1H), 2.19 (t, *J* = 2.4 Hz, 1H), 1.85 – 1.56 (m, 5H), 1.48 – 1.29 (m, 1H). ^{13}C NMR (101 MHz, Chloroform-*d*) δ 143.94, 128.61, 127.78, 127.35, 78.98, 73.19, 66.05, 53.20, 44.28, 35.81, 26.20, 25.02. **HRMS** calcd. for $C_{14}H_{18}N$ 200.1439 $[M+H]^{+}$, found 200.1436.

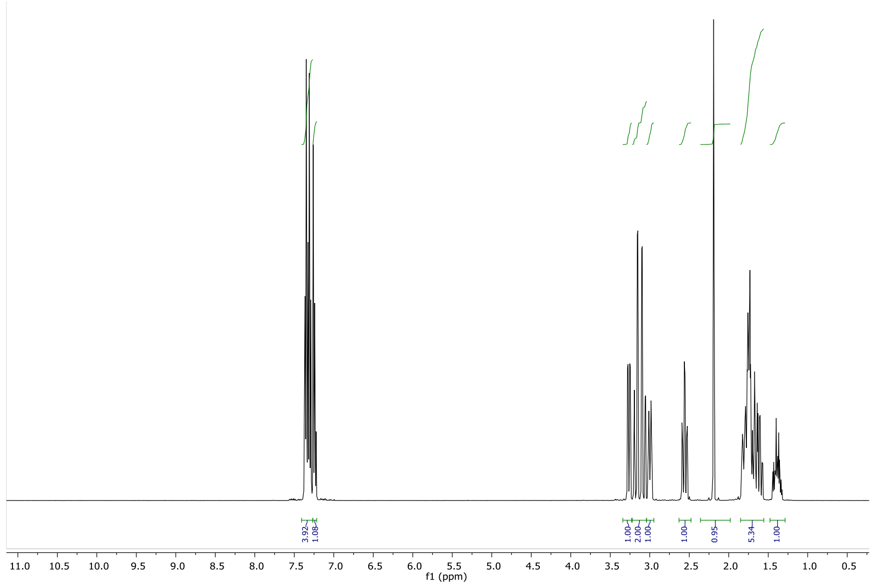

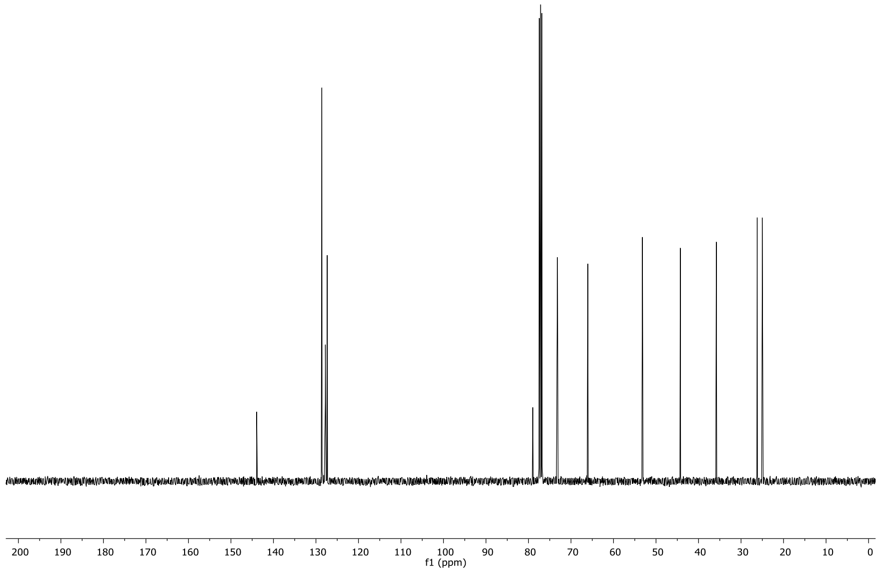

N-*allylazepane (10v)*

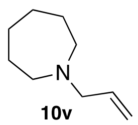

10v
$C_9H_{17}N$
M.W.: 139.24

The crude product was purified by Kugelrohr distillation (220 °C, 100 mbar) to yield the pure product as a clear oil (877 mg, 63%) **^{1}H NMR** (400 MHz, Chloroform-*d*) δ 5.88 (ddt, *J* = 16.7, 10.1, 6.4 Hz, 1H), 5.14 (dq, *J* = 17.2, 1.7 Hz, 1H), 5.09 (ddt, *J* = 10.2, 2.2, 1.2 Hz, 1H), 3.10 (dt, *J* = 6.4, 1.4 Hz, 2H), 2.67 – 2.53 (m, 4H), 1.69 – 1.53 (m, 8H). **^{13}C NMR** (101 MHz, Chloroform-*d*) δ 136.66, 116.99, 61.93, 55.73, 28.20, 27.03. **HRMS** calcd. for $C_9H_{18}N$ 140.1434 $[M+H]^+$, found 140.1446.

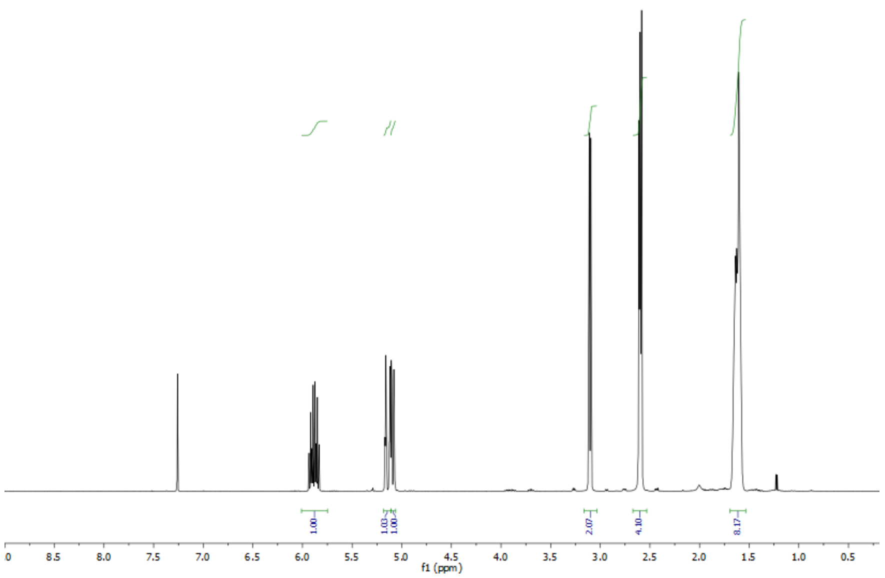

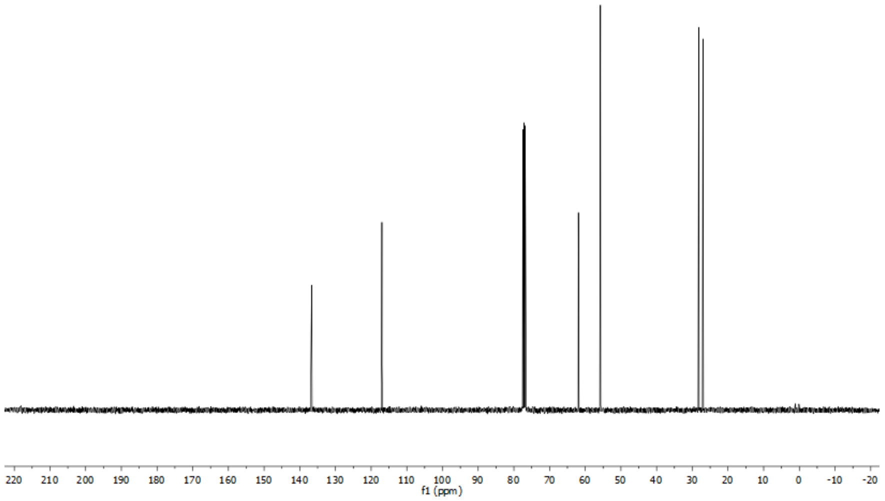

N-*allyl-2-methylazepane (14v)*

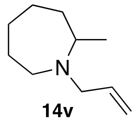

14v
$C_{10}H_{19}N$
M.W.: 153.27

Prior to alkylation 2-methylazepanium chloride (224 mg, 1.5 mmol) was freebased by the addition of 5 M NaOH before extraction by DCM (2 x 20 mL) and the removal of solvent by rotary evaporation. The crude product was purified by Kugelrohr distillation (200 °C, 100 mbar) to yield the pure product as a clear oil (99 mg, 43%) **^{1}H NMR** (400 MHz, Chloroform-*d*) δ 5.85 (ddt, *J* = 16.7, 10.1, 6.4 Hz, 1H), 5.15 (dq, *J* = 17.1, 1.7 Hz, 1H), 5.06 (ddt, *J* = 10.1, 2.3, 1.3 Hz, 1H), 3.17 (dt, *J* = 6.4, 1.4 Hz, 2H), 2.86 – 2.76 (m, 2H), 2.61 (ddd, *J* = 14.3, 6.9, 3.6 Hz, 1H), 1.77 – 1.71 (m, 1H), 1.64 – 1.51 (m, 5H), 1.43 (dtd, *J* = 14.6, 8.2, 2.2 Hz, 2H), 1.02 (d, *J* = 6.4 Hz, 3H). **^{13}C NMR** (101 MHz, Chloroform-*d*) δ 137.79, 116.24, 57.34, 56.35, 49.44, 35.75, 28.93, 28.22, 25.27, 19.68. ***m/z* (±ES)** 110.1 $[M-C_3H_7]^+$, 124.1 $[M-CH_3]^+$, 153.1 $[M]^+$.

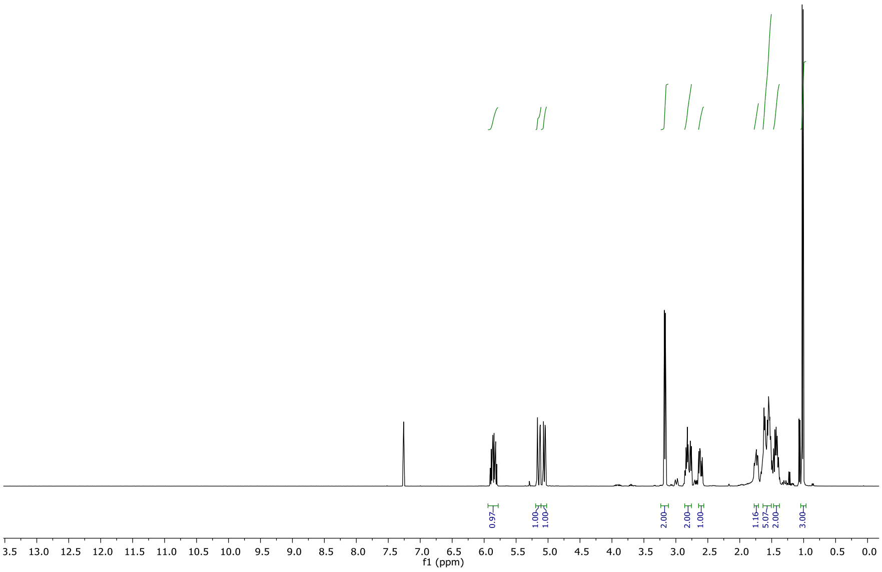

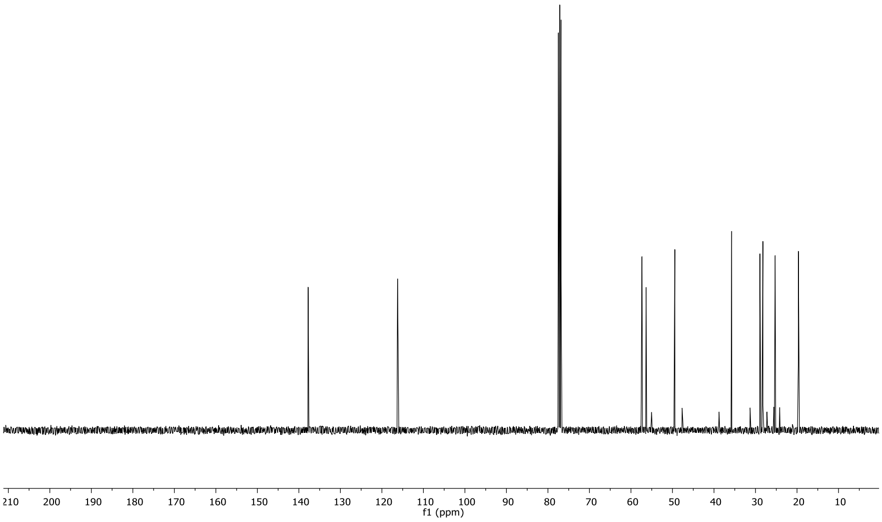

N-*propargyl-2-methylazepane (14vi)*

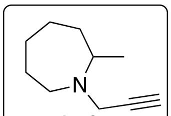

14vi
$C_{10}H_{17}N$
M.W.: 151.25

Prior to alkylation 2-methylazepanium chloride (224 mg, 1.5 mmol) was freebased by the addition of 5 M NaOH before extraction by DCM (2 x 20 mL) and the removal of solvent by rotary evaporation. The crude product was purified by Kugelrohr distillation (200 °C, 100 mbar) to yield the pure product as a clear oil (84 mg, 37%) **^{1}H NMR** (400 MHz, Chloroform-*d*) δ 3.57 – 3.36 (m, 2H), 2.97 – 2.86 (m, 1H), 2.82 (dd, J = 6.4, 4.3 Hz, 2H), 2.17 (t, J = 2.4 Hz, 1H), 1.80 – 1.51 (m, 8H), 1.08 (d, J = 6.4 Hz, 3H). **^{13}C NMR** (101 MHz, Chloroform-*d*) δ 81.31, 71.51, 56.70, 51.63, 43.81, 35.83, 28.73, 28.64, 24.55, 20.25. ***m/z* (±ES)** 108.1 $[M-C_3H_7]^+$, 136.1 $[M-CH_3]^+$, 151.1 $[M]^+$.

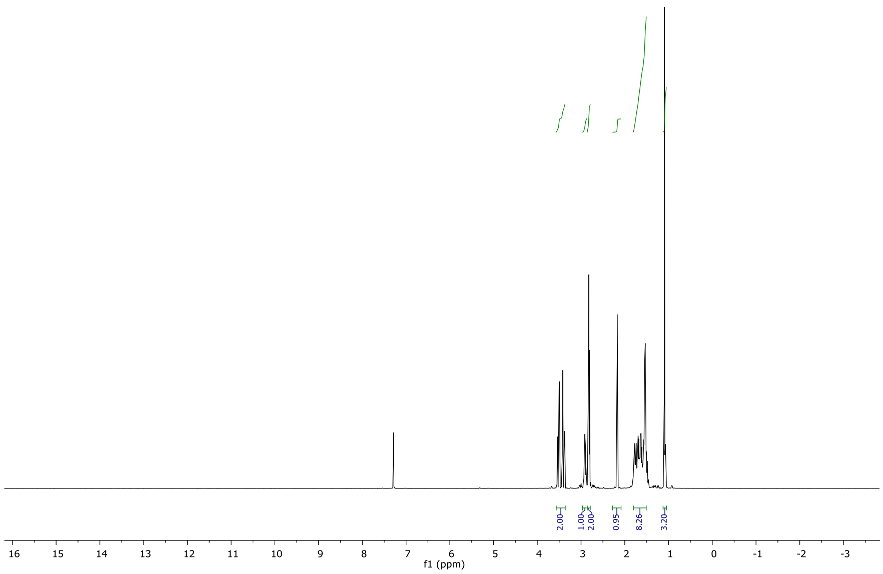

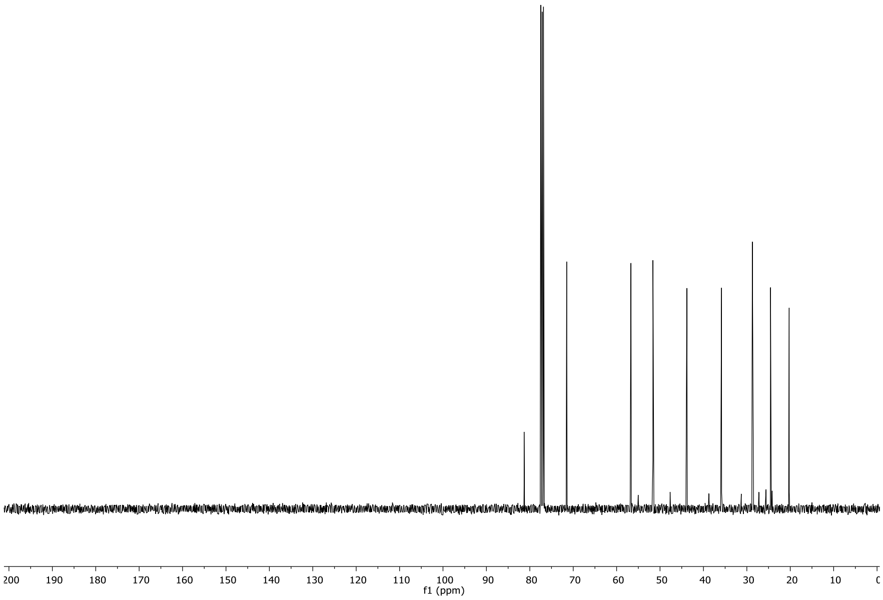

N-*allylazocane (11v)*

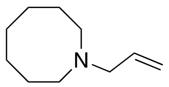

11v
$C_{10}H_{19}N$
M.W.: 153.27

Azocane was alkylated using allyl bromide. The crude product was purified by Kugelrohr distillation (180 °C, 15mbar) to yield the product as a clear oil (1.074 g, 70%). **^{1}H NMR** (400 MHz, Chloroform-*d*) δ 5.87 (ddt, *J* = 16.7, 10.1, 6.4 Hz, 1H), 5.13 (dq, *J* = 17.2, 1.7 Hz, 1H), 5.05 (dq, *J* = 10.2, 1.4 Hz, 1H), 3.09 (dt, *J* = 6.4, 1.4 Hz, 2H), 2.55 (t, *J* = 5.2 Hz, 4H), 1.67 – 1.50 (m, 10H). **^{13}C NMR** (101 MHz, Chloroform-*d*) δ 137.60, 116.19, 62.18, 53.79, 27.97, 27.67, 26.40. **HRMS** calcd. for $C_{10}H_{20}N$ 154.1590 $[M+H]^+$, found 154.1585.

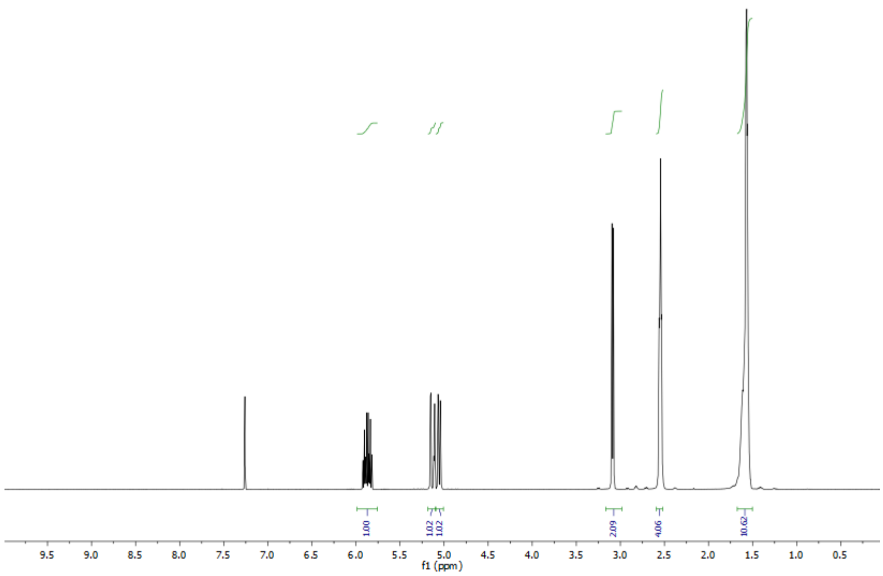

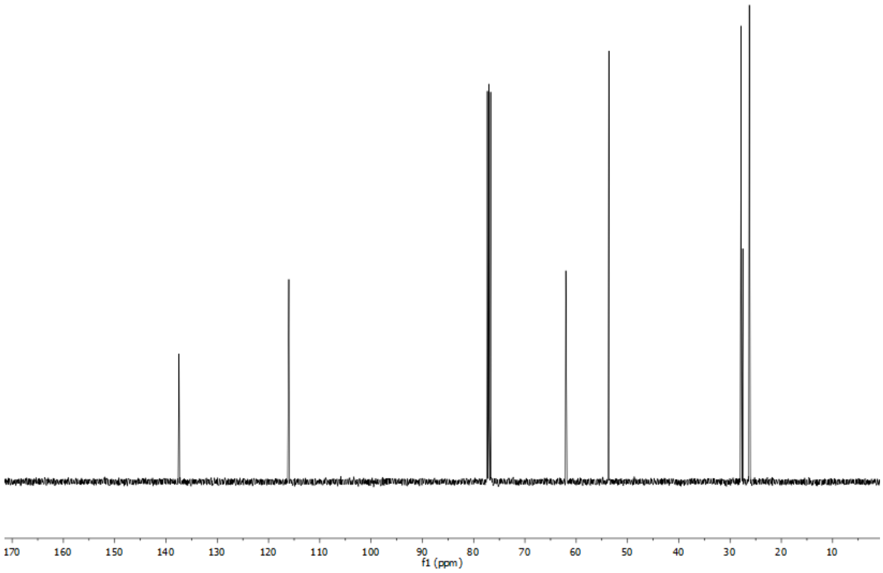

N-*allylpyrrolidine*

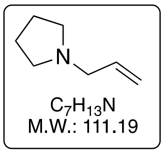

To a stirring solution of pyrrolidine (20 mL, 240 mmol) and diethyl ether (20 mL) is added allyl bromide (10.9 mL, 126 mmol) the solution is stirred overnight then filtered before the solvent is evaporated to yield the product as a yellow oil (16.798 g, 63%) **^{1}H NMR** (400 MHz, Chloroform-*d*) δ 5.91 (ddt, *J* = 16.8, 10.1, 6.5 Hz, 1H), 5.16 (dq, *J* = 17.1, 1.6 Hz, 1H), 5.06 (ddt, *J* = 10.1, 2.1, 1.2 Hz, 1H), 3.07 (dt, *J* = 6.5, 1.3 Hz, 2H), 2.53 – 2.42 (m, 4H), 1.82 – 1.70 (m, 4H). **^{13}C NMR** (101 MHz, Chloroform-*d*) δ 136.42, 116.70, 59.40, 54.13, 23.59. **HRMS** calcd. for $C_7H_{14}N$ 112.1121 $[M+H]^+$, found 112.1117.

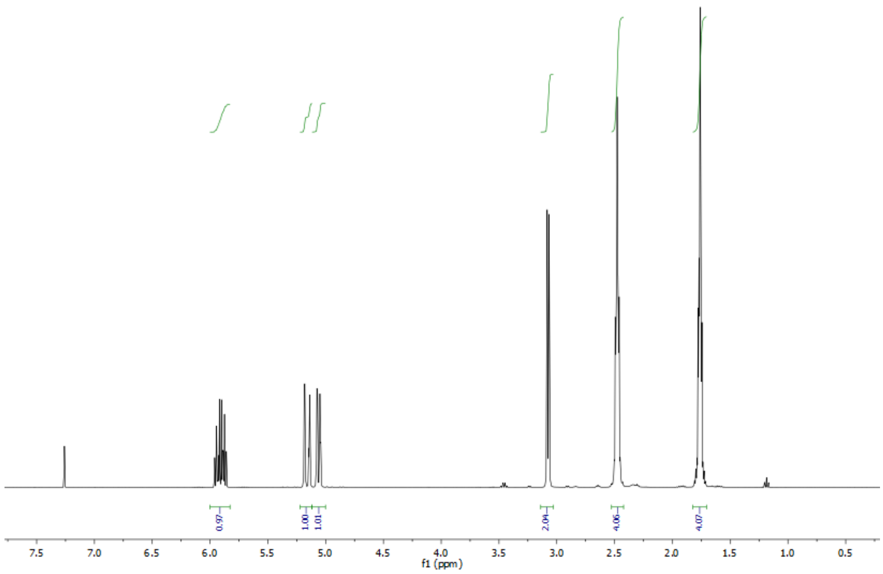

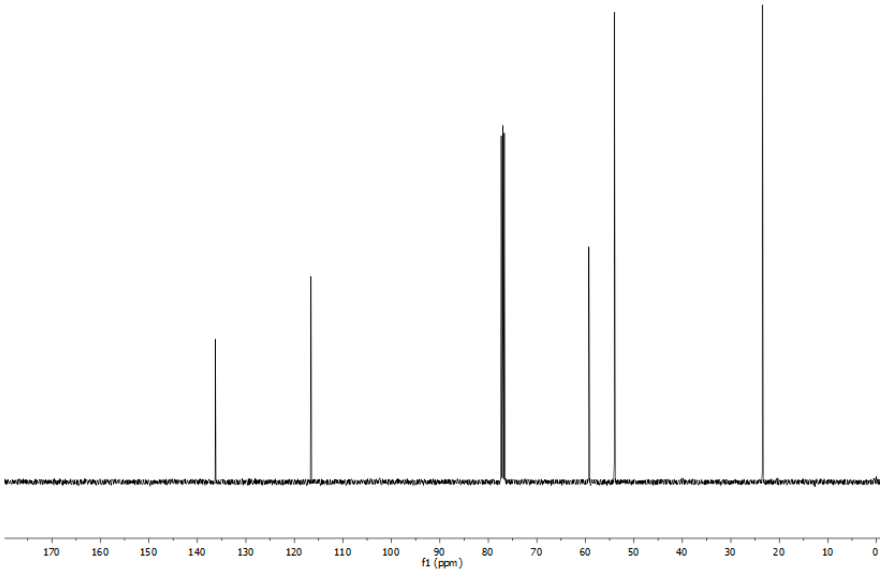

General Procedure 3: *N*-methylation of amine heterocycles

To a stirring solution of formic acid (1.5 equiv.), amine heterocycle (1.0 equiv.) is added dropwise. Formaldehyde (37% solution in H_2O, 1.5 equiv.) is added and the reaction is refluxed at 100 °C for 4 hours. The reaction mixture is cooled to room temperature before dilution with H_2O (20 mL) and basification to pH 10 with portionwise addition of K_2CO_3. The product is extracted into pentane (2 x 25mL) and dried over $MgSO_4$ before the solvent is removed by rotary evaporation to yield the final product.

1,2-Dimethylpiperidine (13iii)

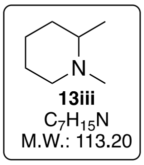

2-Methylpiperidine (3.54 mL, 30 mmol) was methylated with formaldehyde (37% in H_2O, 3.4 mL, 45 mmol) and formic acid (1.7 mL, 45 mmol) following General Procedure 3 to yield pure 1,2-dimethylpiperidine as a clear liquid (3.124 g, 92%). **^{1}H NMR** (500 MHz, Chloroform-*d*) δ 2.82 (dtd, *J* = 11.5, 3.6, 1.5 Hz, 1H), 2.25 (s, 3H), 2.08 – 1.96 (m, 1H), 1.86 (ddq, *J* = 12.5, 6.2, 3.4 Hz, 1H), 1.78 – 1.66 (m, 1H), 1.65 – 1.51 (m, 3H), 1.29 – 1.24 (m, 2H), 1.07 (d, *J* = 6.2 Hz, 3H). **^{13}C NMR**

(126 MHz, Chloroform-*d*) δ 59.50, 57.29, 43.52, 34.92, 26.49, 24.77, 20.57. **HRMS** calcd. for $C_7H_{16}N$ 114.1283 [M+H]$^+$, found 114.1276.

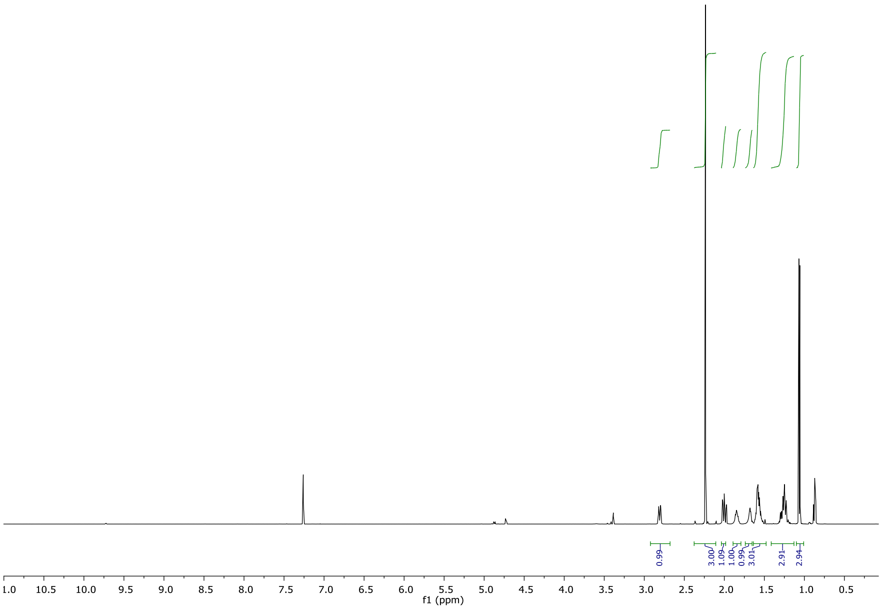

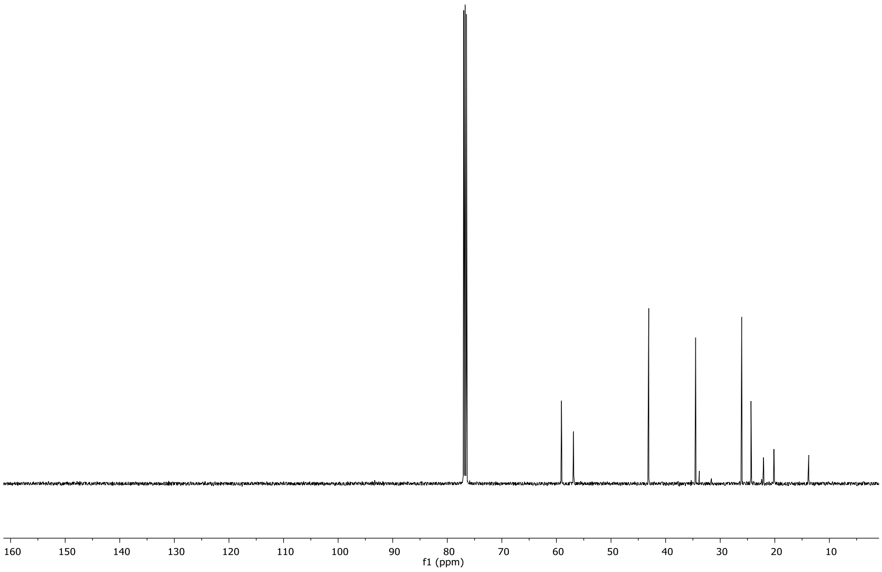

N-*Methyl-2-phenylpiperidine (15iii)*

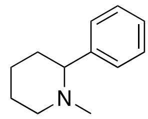

15iii
$C_{12}H_{17}N$
M.W.: 175.28

2-phenylpiperidine (1.61 mL, 10 mmol) was methylated with formaldehyde (37% in H_2O, 3.4 mL, 45 mmol) and formic acid (1.7 mL, 45 mmol) following General Procedure 3 to yield the pure product as a clear liquid (1.630 g, 93%). **^{1}H NMR** (400 MHz, Chloroform-*d*) δ 7.35 – 7.26 (m, 4H), 7.23 (ddt, *J* = 5.9, 5.1, 3.1 Hz, 1H), 3.03 (dtd, *J* = 11.7, 3.4, 1.6 Hz, 1H), 2.74 (dd, *J* = 11.1, 2.9 Hz, 1H), 2.15 – 2.06 (m, 1H), 1.99 (s, 3H), 1.84 – 1.77 (m, 1H), 1.76 – 1.68 (m, 3H), 1.59 (tdd, *J* = 13.3, 11.0, 3.8 Hz, 1H), 1.36 (dtt, *J* = 20.3, 12.4, 4.3 Hz, 1H). **^{13}C NMR** (101 MHz, Chloroform-*d*) δ 145.09, 128.50, 127.58, 127.05, 71.30, 57.72, 44.73, 36.10, 26.35, 25.16. **HRMS** calcd. for $C_{12}H_{18}N$ 176.1439 [M+H]$^+$, found 176.1432.

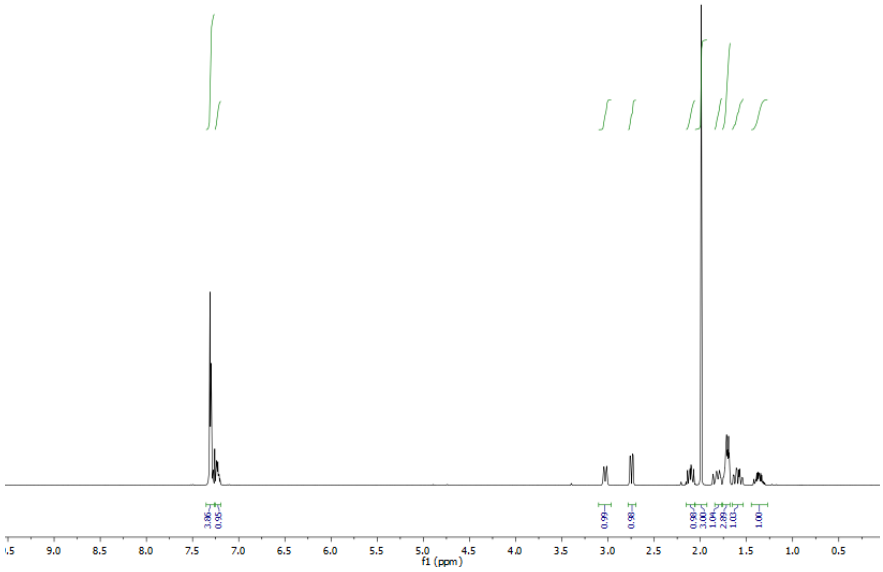

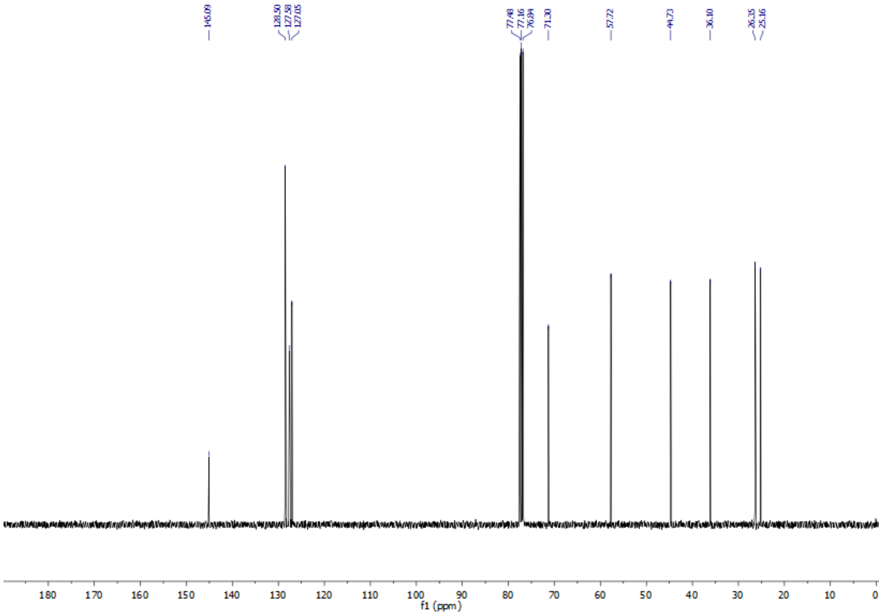

General Procedure 4: *N*-propylation of Amine Heterocycles

To a stirring suspension of K_2CO_3 (2.764 g, 20 mmol) in *N*,*N*-Dimethylformamide (10 mL) is added amine heterocycle (10 mmol) and 1-bromopropane (1 mL, 11 mmol) the reaction is heated to 80 °C and stirred for 18h before cooling to room temperature and quenching with water. The crude product is extracted with ethyl acetate (20 mL) and washed with water (3 x 20mL) and brine (20mL). The crude product is dried over $MgSO_4$ before the solvent is removed by rotary evaporation.

N-*propyl-2-methylpiperidine (13iv)*

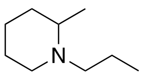

13iv
$C_9H_{19}N$
M.W.: 141.26

The crude product was purified by Kugelrohr distillation (180 °C, 100 mbar) to yield the pure product as a clear oil (748 mg, 53%). **^{1}H NMR** (400 MHz, Chloroform-*d*) δ 2.84 (dt, *J* = 11.4, 3.9 Hz, 1H), 2.59 (ddd, *J* = 12.9, 10.6, 5.5 Hz, 1H), 2.26 (dddd, *J* = 22.7, 12.2, 9.7, 5.7 Hz, 2H), 2.13 (td, *J* = 11.1, 3.2 Hz, 1H), 1.70 – 1.37 (m, 6H), 1.34 – 1.20 (m, 2H), 1.05 (dd, *J* = 6.2, 0.8 Hz, 3H), 0.85 (td, *J* = 7.4,

0.8 Hz, 3H). **^{13}C NMR** (101 MHz, Chloroform-*d*) δ 56.39, 55.95, 52.41, 34.90, 26.41, 24.31, 19.39, 18.48, 12.27. ***m/z* (±ES)** 112.1 [M-C_2H_5]$^+$, 126.1 [M-CH_3]$^+$, 141.1 [M]$^+$.

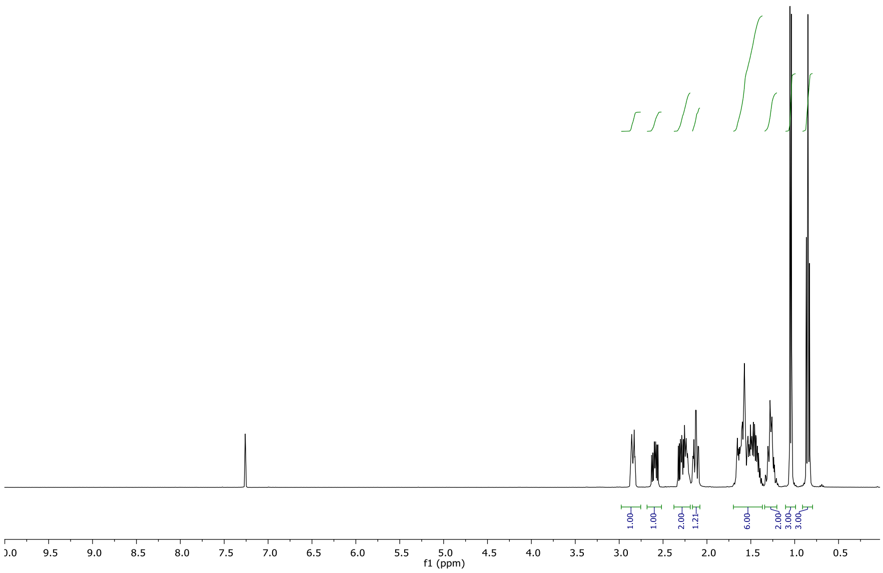

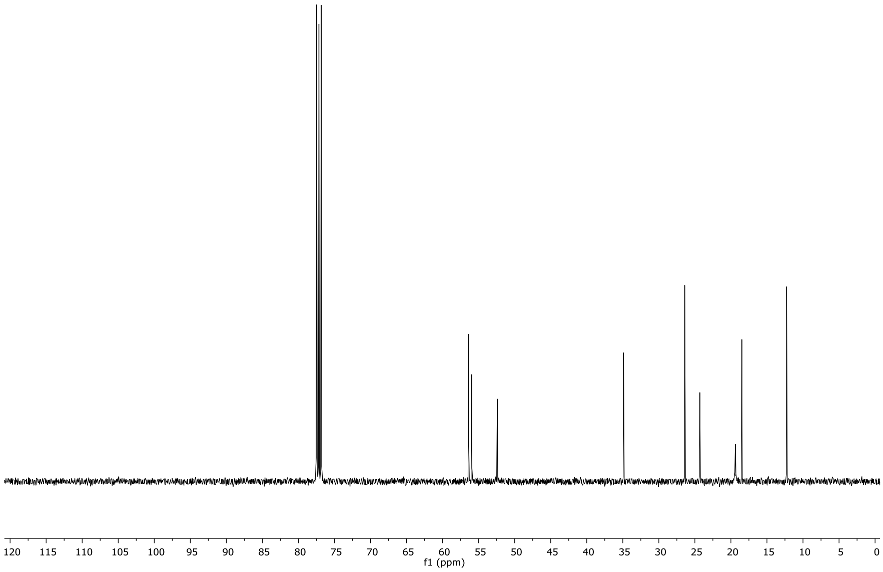

N-*propyl-2-phenylpiperidine (15iv)*

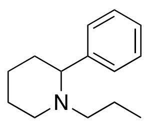

15iv
$C_{14}H_{21}N$
M.W.: 203.33

The crude product was purified using a BioTage Isolera™ system running a solvent gradient of 90:10 *n*-heptane:ethyl acetate to 70:30 *n*-heptane:ethyl acetate yielding the pure product as a clear oil (1.178 g, 58% yield). **^{1}H NMR** (500 MHz, Chloroform-*d*) δ 7.34 – 7.27 (m, 4H), 7.24 – 7.20 (m, 1H), 3.21 – 3.11 (m, 1H), 2.99 (dd, *J* = 11.1, 2.9 Hz, 1H), 2.36 (ddd, *J* = 12.6, 9.8, 6.9 Hz, 1H), 2.03 (td, *J* = 11.7, 3.2 Hz, 1H), 1.85 (ddd, *J* = 12.6, 9.3, 4.5 Hz, 1H), 1.81 – 1.75 (m, 1H), 1.73 – 1.61 (m, 3H), 1.55 (tdd, *J* = 13.3, 11.1, 3.8 Hz, 1H), 1.46 – 1.30 (m, 3H), 0.71 (t, *J* = 7.4 Hz, 3H). **^{13}C NMR** (126 MHz, Chloroform-*d*) δ 145.73, 128.40, 127.63, 126.79, 69.28, 57.48, 53.39, 36.94, 26.29, 25.33, 19.30, 11.98. **HRMS** calcd. for $C_{14}H_{22}N$ 204.1752 [M+H]$^+$, found 204.1875.

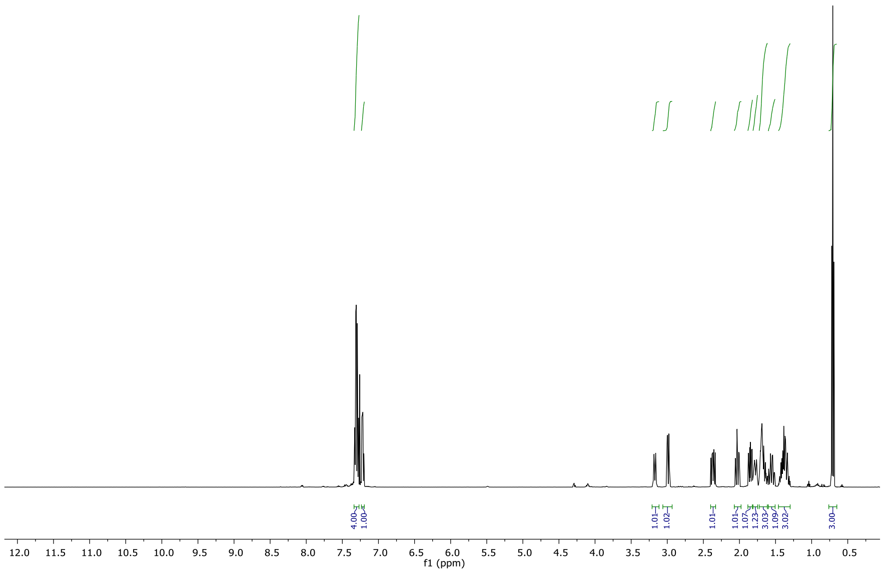

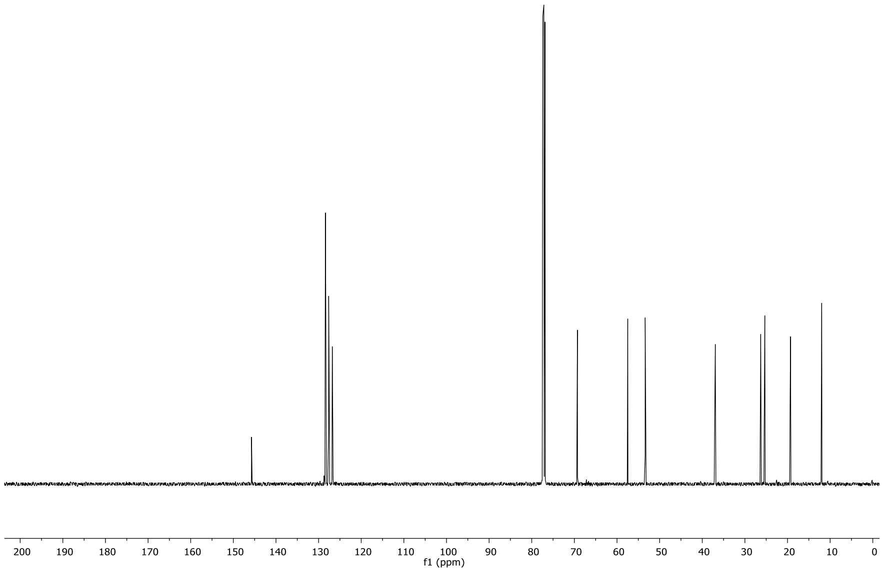

General Procedure 5: *N*-benzylation of Amine Heterocycles

To a stirring solution of benzaldehyde (5.08 mL, 50 mmol) in 2-methyltetrohydrofuran is added acetic acid (572 µL, 10 mmol) and sodium triacetoxyborohydride (5.298 g, 25 mmol). Amine heterocycle (10 mmol) is added and the reaction is stirred overnight before being quenched with 1M NaOH (20mL). The organic extract is removed before being washed with brine and dried over $MgSO_4$. The organic extracts are then filtered and the solvent removed by rotary evaporation to yield the crude product.

N-*benzyl-2-methylpiperidine (13ii)*

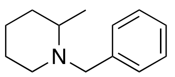

13ii
$C_{13}H_{19}N$
M.W.: 189.30

The crude product was purified using a BioTage Isolera™ system running a solvent gradient of 90:10 n-heptane:ethyl acetate to 70:30 n-heptane:ethyl acetate yielding the pure product as a clear oil (1.473 g, 78% yield). **^{1}H NMR** (400 MHz, Chloroform-*d*) δ 7.51 – 7.02 (m, 5H), 4.00 (d, *J* = 13.4 Hz, 1H), 3.20 (d, *J* = 13.4 Hz, 1H), 2.73 (dtd, *J* = 11.6, 4.0, 1.2 Hz, 1H), 2.30 (dqd, *J* = 9.1, 6.1, 2.6 Hz, 1H), 1.95 (ddd, *J* = 11.5, 10.2, 3.6 Hz, 1H), 1.64 (qdd, *J* = 6.4, 3.3, 1.6 Hz, 2H), 1.56 – 1.24

(m, 4H), 1.17 (d, *J* = 6.2 Hz, 3H). **^{13}C NMR** (101 MHz, Chloroform-*d*) δ 139.71, 129.27, 128.18, 126.74, 58.66, 56.52, 52.32, 34.90, 26.22, 24.18, 19.71. **HRMS** calcd. for $C_{13}H_{20}N$ 190.1596 $[M+H]^+$, found 190.1723.

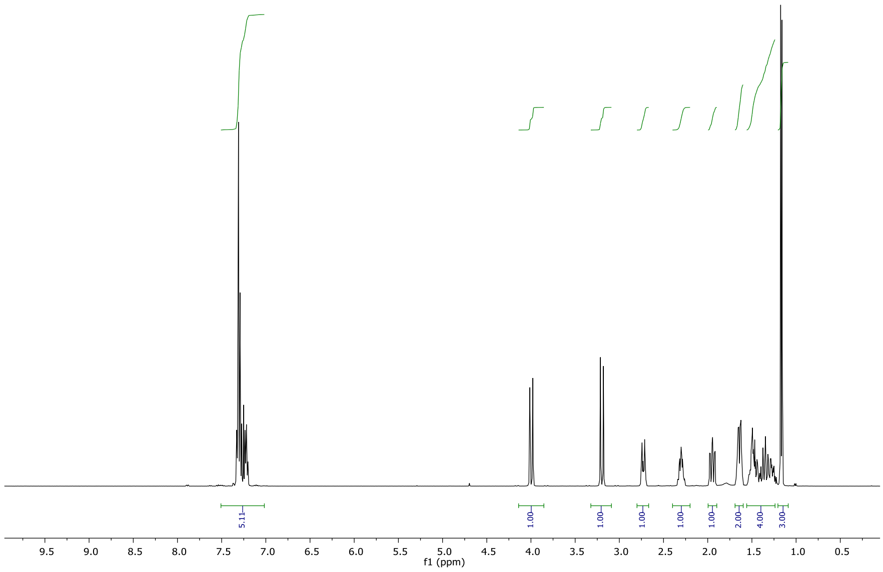

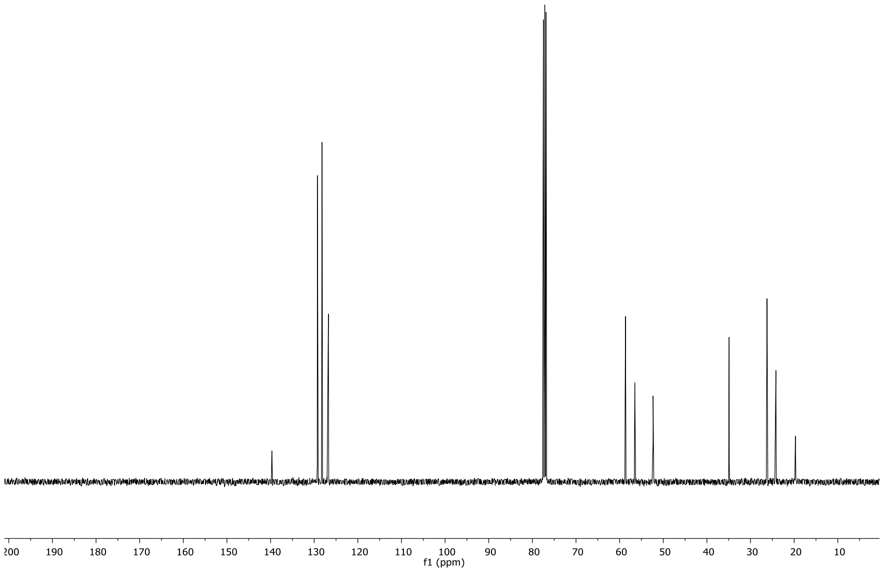

N-*benzyl-2-phenylpiperidine (15ii)*

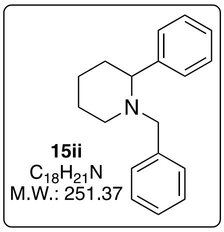

The crude product was washed with methanol to yield the pure product as a white solid (1.843 g, 73%). **^{1}H NMR** (400 MHz, Chloroform-*d*) δ 7.46 (d, *J* = 7.5 Hz, 2H), 7.33 (t, *J* = 7.5 Hz, 2H), 7.27 (d, *J* = 4.4 Hz, 4H), 7.21 (qd, *J* = 8.7, 8.2, 2.7 Hz, 2H), 3.77 (d, *J* = 13.6 Hz, 1H), 3.11 (dd, *J* = 11.1, 2.8 Hz, 1H), 3.01 – 2.93 (m, 1H), 2.81 (d, *J* = 13.5 Hz, 1H), 2.01 – 1.89 (m, 1H), 1.78 (dh, *J* = 10.7, 3.1 Hz, 2H), 1.64 – 1.55 (m, 3H), 1.44 – 1.30 (m, 1H). **^{13}C NMR** (101 MHz, Chloroform-*d*) δ 145.88, 139.97, 128.83, 128.62, 128.13, 127.59, 126.97, 126.65, 69.34, 59.93, 53.49, 37.16, 26.15, 25.38. ***m/z* (±ES)** 160.1 $[M-C_7H_7]^+$ 174.1 $[M-C_6H_5]^+$ 251.1 $[M]^+$

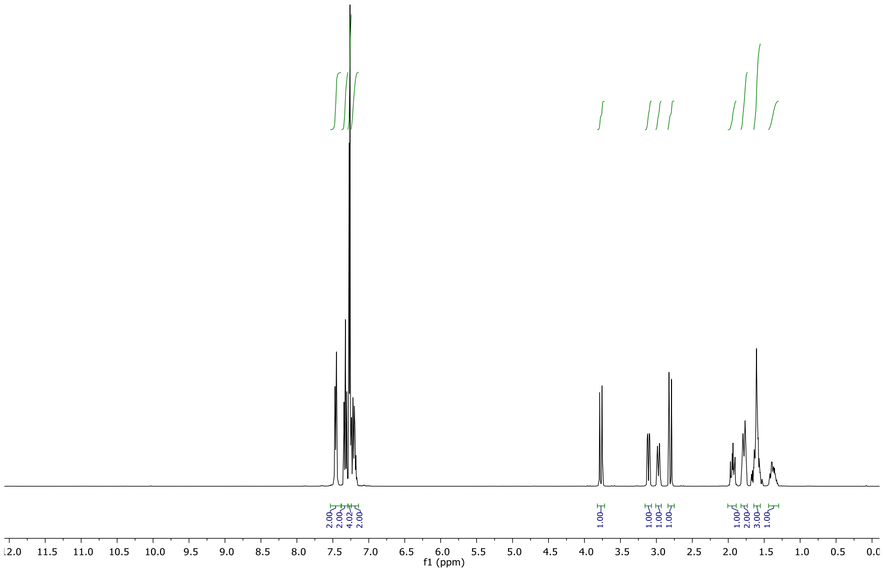

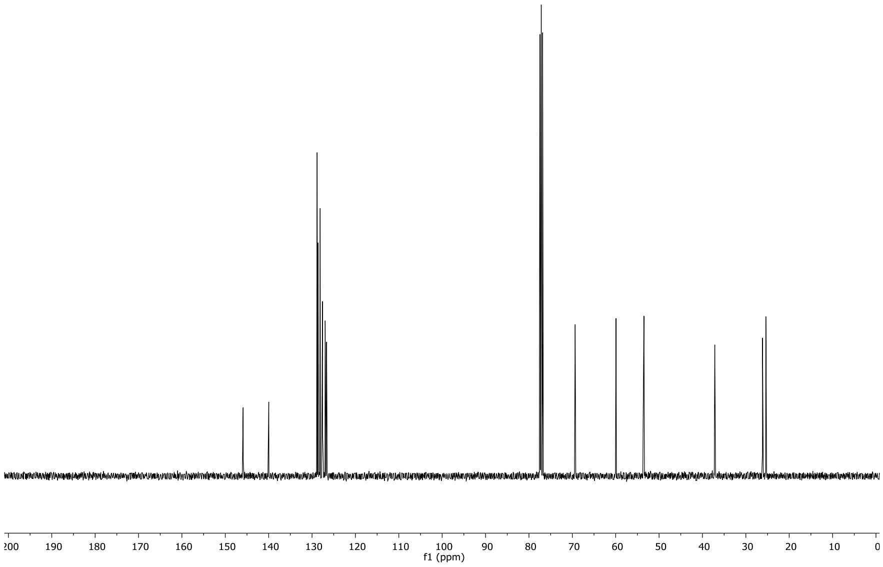

_N_-cyclopropyl-2-methylpiperidine (13i)

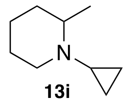

13i
$C_9H_{17}N$
M.W.: 139.24

2-Methylpiperidine (1.18 mL, 10 mmol), cyclopropylboronic acid (1.72 g, 20 mmol) and sodium carbonate (2.12 g, 20 mmol) were suspended in dichloroethane (16.7 mL), stirred and heated to 70°C. A hot suspension of copper (II) acetate (1.816 g, 10 mmol) and 2,2′-bipyridine (1.56 g, 10 mmol) in dichloroethane (83.3 mL) was added and the reaction was stirred at 70 °C for 4 hours and then allowed to reach room temperature. The reaction was quenched with the addition of a 25% solution of ammonium hydroxide (25 mL). The organic layer was separated and washed with 1 M HCl (3 x 20 mL). The combined aqueous extracts were then basified and extracted with DCM (2 x 20 mL). The combined organic extracts were dried over $MgSO_4$ and filtered before the solvent was removed by rotary evaporation to yield the crude product. The crude product was purified by Kugelrohr distillation (180 °C, 100 mbar) to yield the final product as a clear oil (361 mg, 26% yield). **^{1}H NMR** (400 MHz, Chloroform-*d*) δ 3.00 (dtd, *J* = 11.6, 3.7, 1.5 Hz, 1H), 2.26 (dqd, *J* = 9.3, 6.3, 2.6 Hz, 1H), 2.14 (td, *J* = 11.6, 3.0 Hz, 1H), 1.69 – 1.42 (m, 5H), 1.30 – 1.20 (m, 2H), 1.18 (d, *J* = 6.4 Hz, 3H), 0.70 – 0.46 (m, 2H), 0.39 (dddd, *J* = 11.0, 6.9,

5.5, 3.2 Hz, 1H), 0.33 – 0.23 (m, 1H). **^{13}C NMR** (101 MHz, Chloroform-*d*) δ 60.05, 54.79, 37.38, 34.68, 26.15, 24.26, 20.65, 10.00, 4.26. ***m/z* (±ES)** 97.1 [M-C_3H_6]$^+$, 124.1 [M-CH_3]$^+$, 139.1 [M]$^+$.

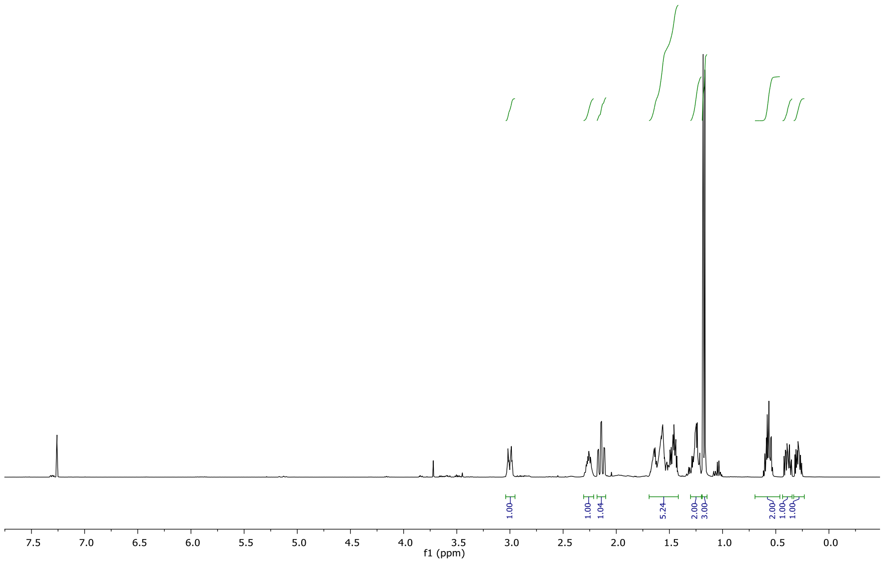

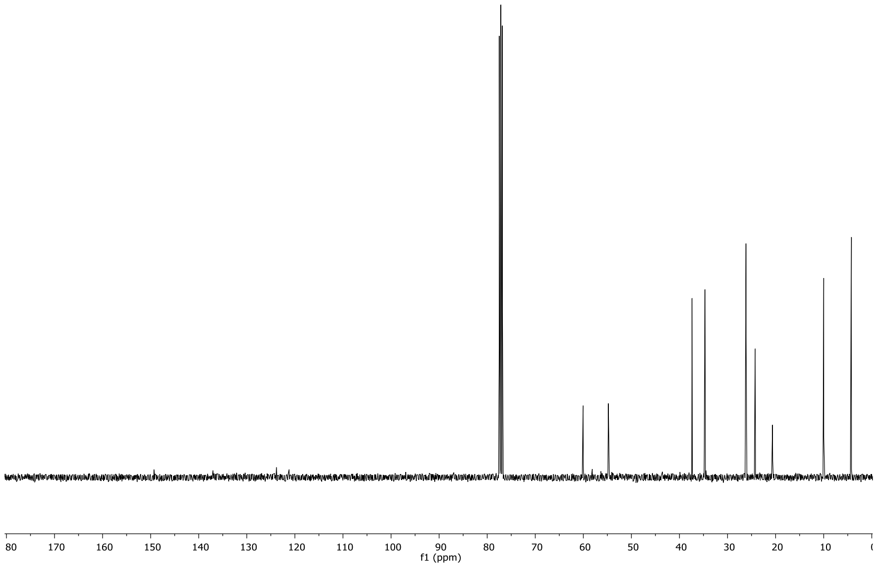

N-allyl-5-hydroxypentan-1-amine

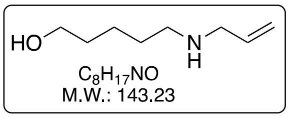

5-Hydroxypentanal (194 µL, 2 mmol) was dissolved in methanol (20 mL) followed by the addition of glacial acetic acid (114 µL, 2 mmol), allylamine (300 µL, 4 mmol) and the portionwise addition of sodium triacetoxyborohydride (636 mg, 3 mmol). The mixture was stirred overnight and then loaded crude onto an SCX cartridge. The cartridge was washed with methanol (3 x 20 mL) before elution by 7 N ammonia in methanol. The solvent was then removed to yield the pure product as a yellow oil (177 mg, 62%). **^{1}H NMR** (400 MHz, Chloroform-*d*) δ 5.90 (ddt, *J* = 16.5, 10.3, 6.0 Hz, 1H), 5.16 (dq, *J* = 17.2, 1.7 Hz, 1H), 5.08 (dq, *J* = 10.1, 1.5 Hz, 1H), 3.63 (t, *J* = 6.5 Hz, 2H), 3.24 (dt, *J* = 6.1, 1.4 Hz, 2H), 2.62 (t, *J* = 7.0 Hz, 2H), 1.54 (ddt, *J* = 21.7, 13.1, 6.6 Hz, 4H), 1.45 – 1.35 (m, 2H). **^{13}C NMR** (101 MHz, Chloroform-*d*) δ 136.94, 116.03, 62.75, 52.63, 49.34, 32.62, 29.80, 23.58. ***m/z* (±ES)** 70.1 [M-C_4H_9O]$^+$ 143.1 [M]$^+$

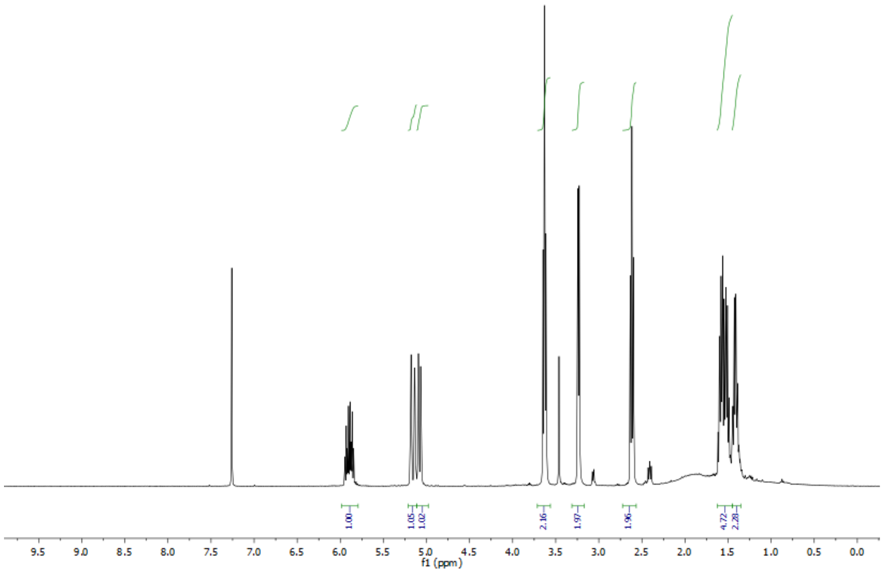

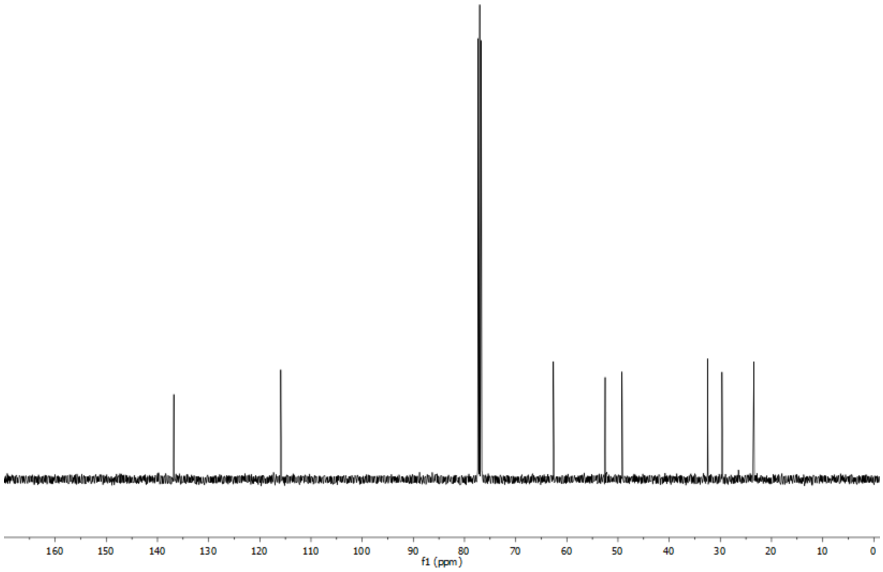

Synthesis of Keto-Alcohol Substrates

5-Oxohexanol

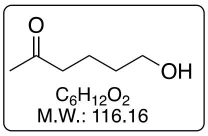

Under an atmosphere of nitrogen, δ-valerolactone (3.52 mL, 37.9 mmol) was dissolved in diethyl ether (100 mL) and cooled to -78 °C. A 1.6 M solution of methyllithium in diethyl ether (25 mL, 40 mmol) was added dropwise and the reaction was stirred for 1 hour. The reaction was quenched with the addition of a saturated solution of ammonium chloride (40 mL) and allowed to reach room temperature. The product was extracted with ethyl acetate (3 x 40 mL) and the combined organic extracts were dried over $MgSO_4$. The solvent was removed by rotary evaporation and the crude product was purified by flash column chromatography (hexane:EtOAc, 1:1) to yield the pure product as a clear oil (2.34 g, 53% yield). The product was observed to tautomerize with the lactol form on TLC and in NMR experiments. **^{1}H NMR** (400 MHz, Chloroform-*d*) δ 3.47 – 3.30 (m, 2H), 2.46 (t, *J* = 7.3 Hz, 2H), 2.13 (s, 3H), 1.83 – 1.62 (m, 4H). **^{13}C NMR**

(101 MHz, Chloroform-*d*) δ 209.17, 61.51, 43.70, 30.02, 29.66, 18.94. ***m/z* (±ES)** 98.1 [M-H_2O]$^+$, 116.1 [M]$^+$.

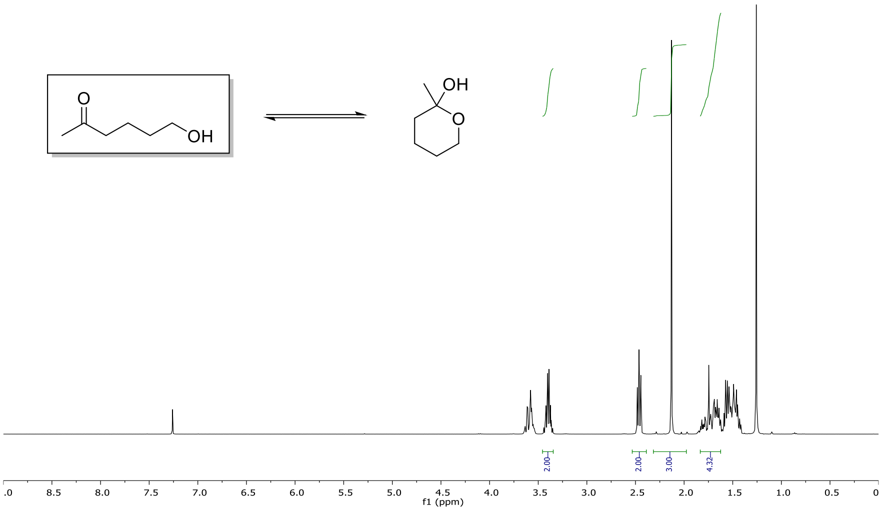

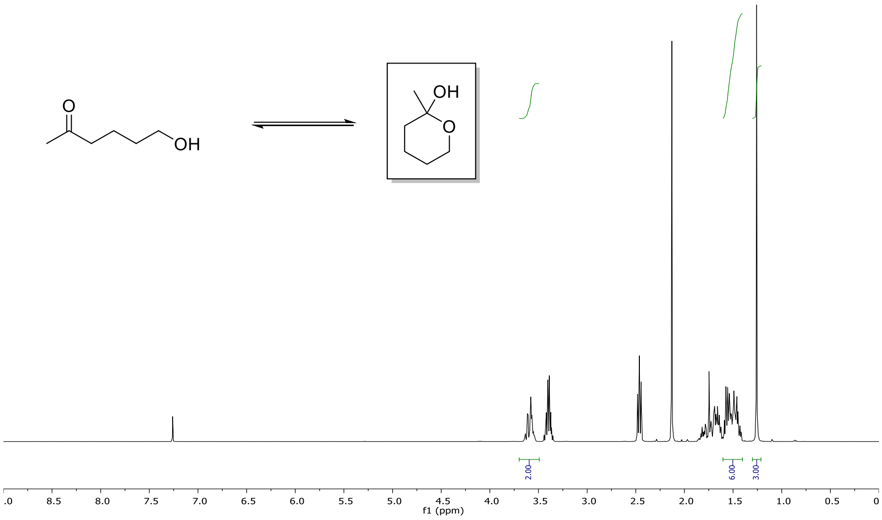

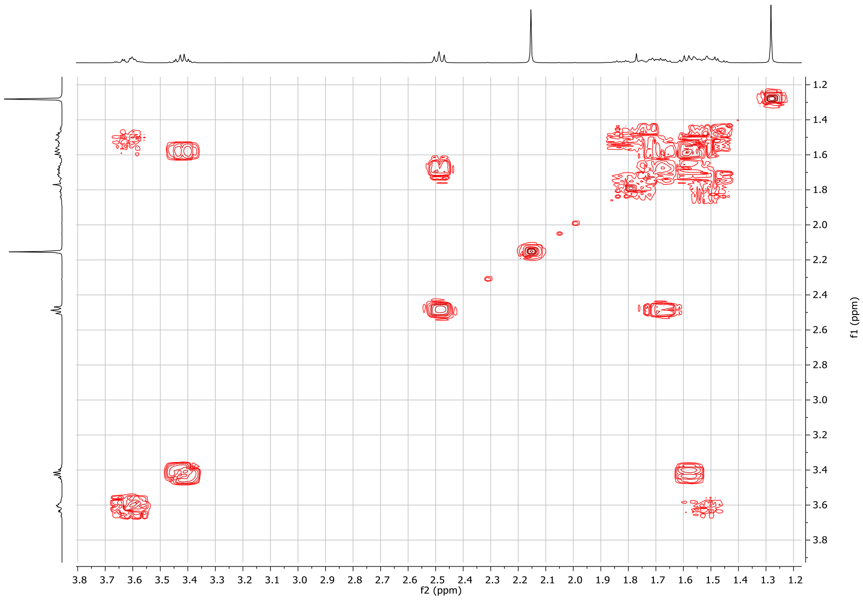

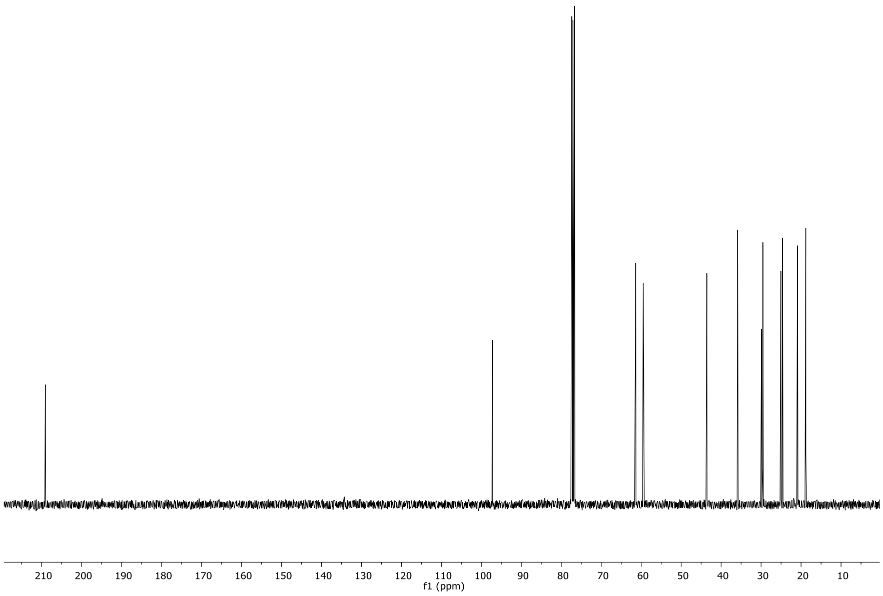

6-Oxoheptanol

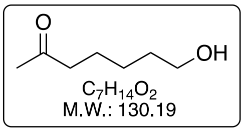

Under an atmosphere of nitrogen, ε-caprolactone (3.9 mL, 35 mmol) was dissolved in diethyl ether (100 mL) and cooled to -78 °C. A 1.6 M solution of methyllithium in diethyl ether (24 mL, 38 mmol) was added dropwise and the reaction was stirred for 1 hour. The reaction was quenched with the addition of a saturated solution of ammonium chloride (40 mL) and allowed to reach room temperature. The product was extracted with ethyl acetate (3 x 40 mL) and the combined organic extracts were dried over $MgSO_4$. The solvent was removed by rotary evaporation and the crude product was purified by flash column chromatography (DCM:methanol, 95:5) to yield the pure product as a clear oil (1.92g, 42% yield). **^{1}H NMR** (400 MHz, Chloroform-*d*) δ 3.62 (t, *J* = 6.6 Hz, 2H), 2.43 (t, *J* = 7.3 Hz, 2H), 2.12 (s, 3H), 1.62 – 1.52 (m, 4H), 1.39 – 1.30 (m, 2H). **^{13}C NMR** (101 MHz, Chloroform-*d*) δ 209.43, 62.69, 43.72, 32.51, 30.05, 25.38, 23.52. ***m/z* (±ES)** 112.1 [M-H_2O]$^+$, 130.1 [M]$^+$.

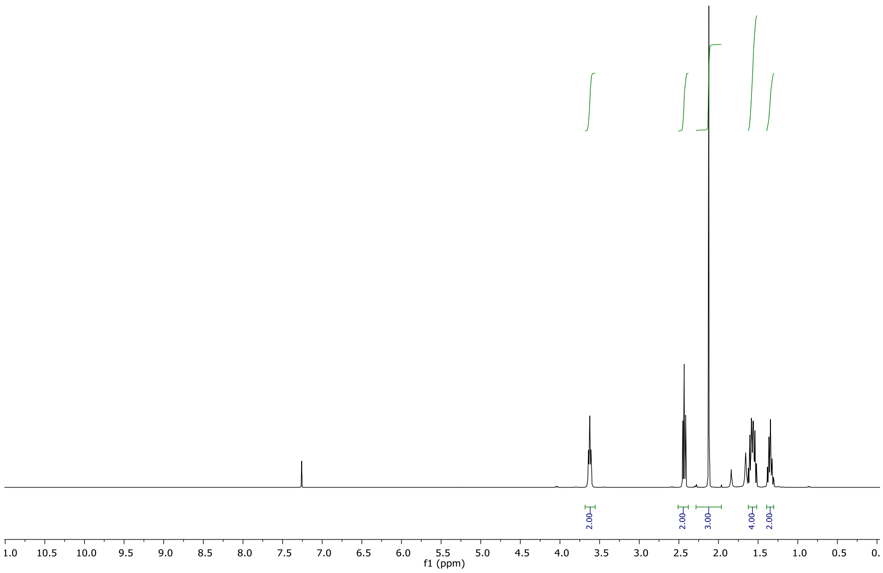

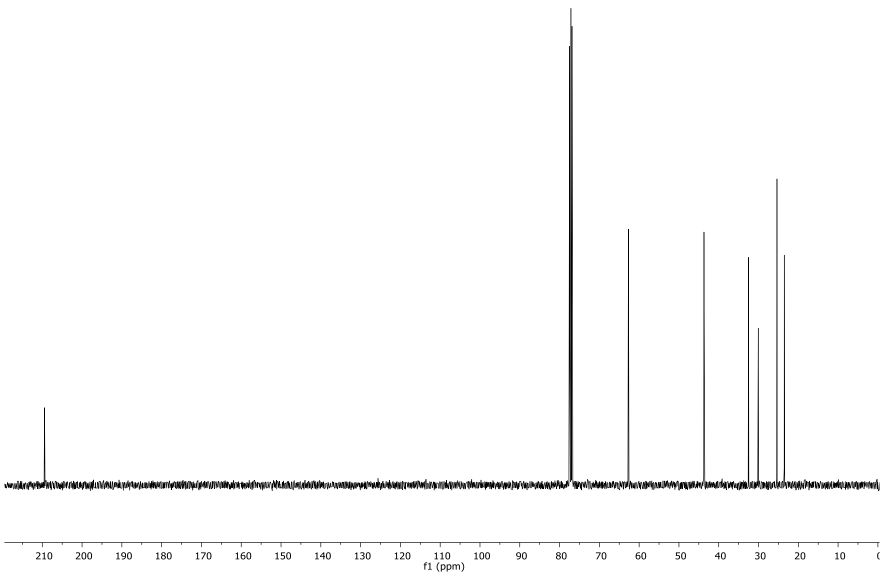

4-Benzoylbutanol

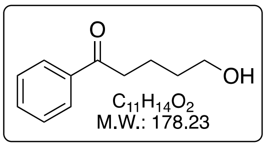

Under an atmosphere of nitrogen, bromobenzene (2.13 mL, 20 mmol) was dissolved in anhydrous diethyl ether (60 mL) and stirred. The solution was cooled to -78 °C and tertiary butyllithium (1.7 M in pentane, 14.1 mL, 24 mmol) was added dropwise over 45 minutes. The reaction was stirred at -78 °C for 30 minutes before a solution of δ-valerolactone (1.86 mL, 20 mmol) in anhydrous diethyl ether (20 mL) was added and the reaction was allowed to reach room temperature. The reaction was stirred for 2 hours before quenching with 30 mL of brine and extraction into ethyl acetate (3 x 20 mL). The combined organic layers were then dried over $MgSO_4$ before the solvent was removed to yield the crude product. The crude product was purified by flash column chromatography (hexane:EtOAc, 5:1) to yield the pure product as a clear oil (1.71 g, 48%). To promote the open-chain tautomer, NMR experiments were carried out in deuterium oxide with DMSO added for solubility. **^{1}H NMR** (400 MHz, D_2O:DMSO-*d*6, 90:10, Water Suppression Experiment) δ 8.05 – 7.98 (m, 2H), 7.72 (t, *J* = 7.4 Hz, 1H), 7.59 (t, *J* = 7.7 Hz, 2H), 3.63 (t, *J* = 6.4 Hz, 2H), 3.11 (t, *J* = 7.2 Hz, 2H), 1.77 – 1.69 (m, 2H), 1.62 (dq, *J* = 9.4, 6.3 Hz, 2H). **^{13}C NMR** (101 MHz, D_2O:DMSO-*d*6, 90:10) δ 206.56, 137.78, 135.54, 130.49, 129.79, 62.74, 39.59, 32.49, 22.02. ***m/z* (±ES)** 160.1 $[M-H_2O]^+$, 178.1 $[M]^+$.

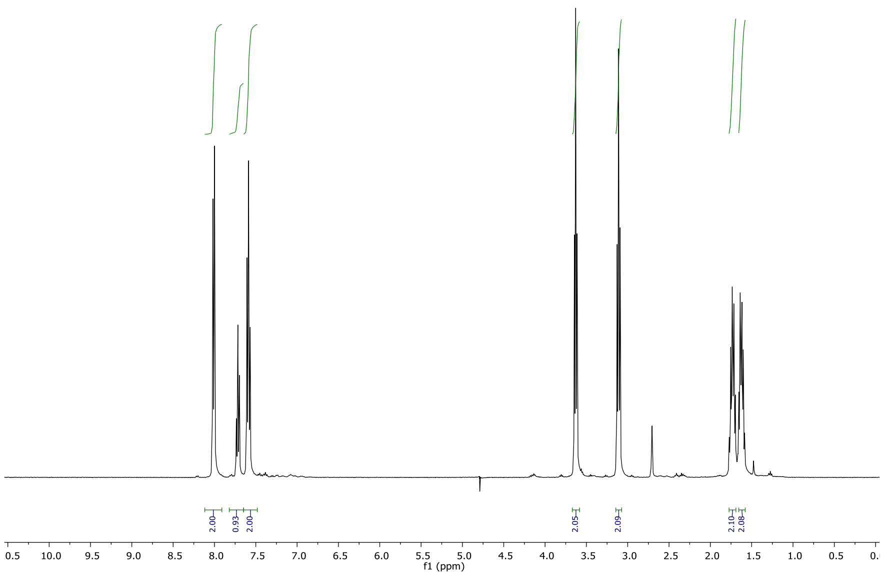

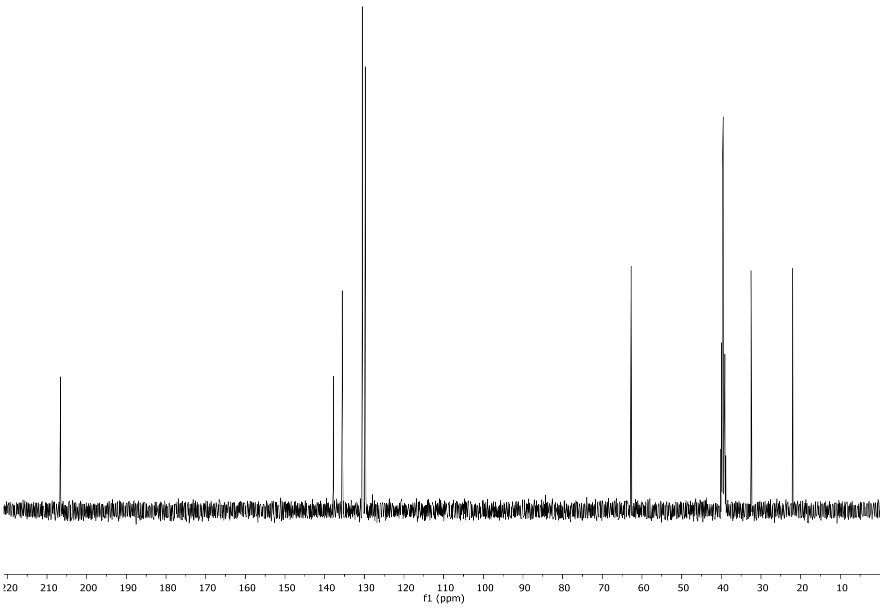

3-Pyridin-3-oylpropanol

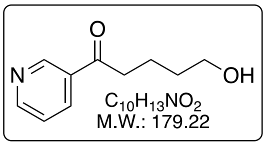

Under an atmosphere of nitrogen, 3-bromopyridine (1.93 mL, 20 mmol) was dissolved in anhydrous diethyl ether (60 mL) and stirred. The solution was cooled to -78 °C and tertiary butyllithium (1.7 M in pentane, 14.1 mL, 24 mmol) was added dropwise over 45 minutes. The reaction was stirred at -78 °C for 30 minutes before a solution of γ-butyrolactone (1.54 mL, 20 mmol) in anhydrous diethyl ether (20 mL) was added and the reaction was allowed to reach room temperature. The reaction was stirred for 2 hours before quenching with 30 mL of brine and extraction into ethyl acetate (3 x 20 mL). The combined organic layers were then dried over $MgSO_4$ before the solvent was removed to yield the crude product. The crude product was purified by flash column chromatography (hexane:EtOAc, 4:1) to yield the pure product as a yellow oil (2.21 g, 67%). NMR experiments were performed in deuterium oxide to promote the open-chain tautomer. **^{1}H NMR** (400 MHz, D_2O) δ 8.96 (dd, *J* = 2.3, 0.9 Hz, 1H), 8.65 (dd, *J* = 5.0, 1.6 Hz, 1H), 8.27 (dt, *J* = 8.1, 2.0 Hz, 1H), 7.53 (ddd, *J* = 8.1, 4.9, 0.9 Hz, 1H), 3.65

(t, *J* = 6.5 Hz, 2H), 3.10 (t, *J* = 7.3 Hz, 2H), 1.90 (p, *J* = 6.7 Hz, 2H). **^{13}C NMR** (101 MHz, D_2O) δ 202.81, 152.61, 148.37, 136.70, 132.02, 124.32, 60.78, 35.14, 25.87. ***m/z* (±ES)** 174.1 $[M\text{-}H_2O]^+$, 192.1 $[M]^+$.

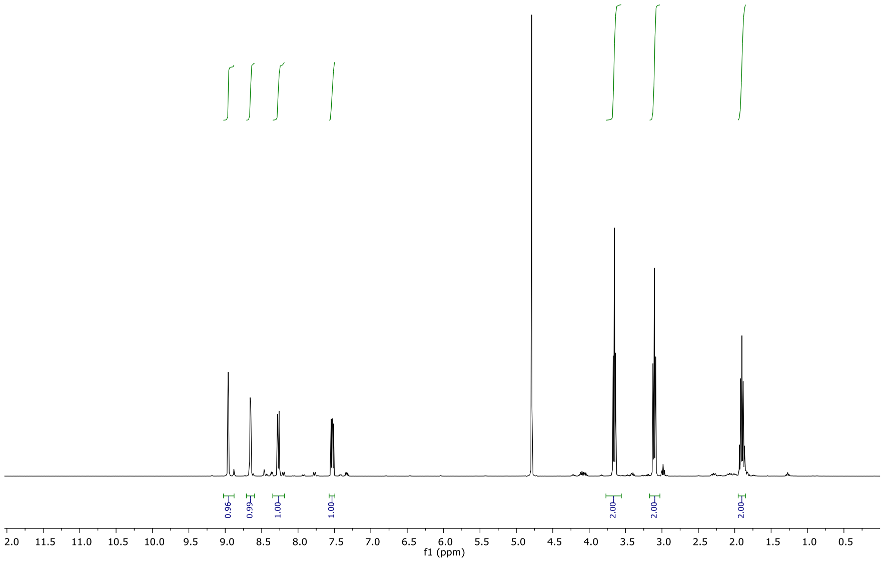

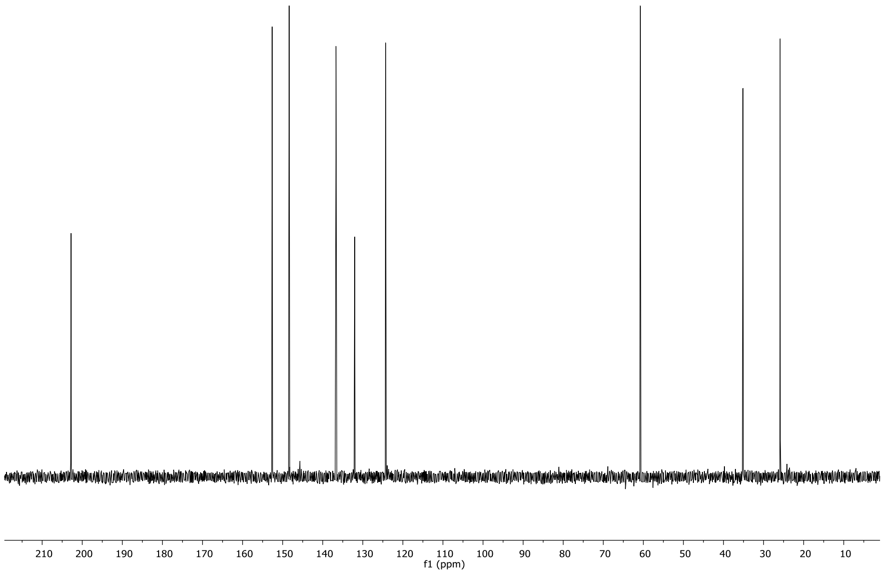

General procedure 6: Synthesis of Diketones

To a solution of lithium diisopropylamine (2 M LDA in hexane, 1.2 eq.) in THF (60 mL) at -78 °C was added dropwise a solution of methylketone (1 eq.) in THF (10.0 mL). The resulting mixture was stirred for 30 min at -78 °C. Chloroacetone (1.2 eq.) was added dropwise and the reaction mixture was stirred for 20 min at -78 °C, then allowed to warm to 0 °C (30 min) and then to r.t. After 3h, the reaction was quenched by adding brine (30 mL), following extraction with dichloromethane (3 x 30 mL). The combined organic layers were dried over anhydrous $MgSO_4$ and the solvent was removed under reduced pressure. The crude product was purified by silica gel flash column chromatography (cyclohexane:ethyl acetate gradient 9.5:0.5 to 8:2).

2,5-decadione (21)

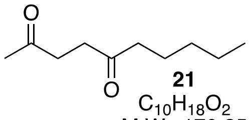

21
$C_{10}H_{18}O_2$
M.W.: 170.25

2-Heptanone (2 mL, 14.4 mmol), 2 M LDA (8.6 mL) and chloroacetone (1.39 mL) were submitted to the General Procedure 6 affording diketone **21** as a pale-yellow oil in 63% yield (1.54 g). **1H NMR** (400.13 MHz, CDCl3): δ 2.74-2.64 (m, 4H), 2.44 (t, *J* = 7.5, 2H), 2.18 (s, 3H), 1.57 (quint, *J* = 7.5, 2H), 1.37-1.19 (m, 4H), 0.88 (t, *J* = 7.3, 3H). **13C NMR** (101 MHz, CDCl3): δ 209.7 (C), 207.3 (C), 42.8 (CH2), 36.9 (CH2), 36.0 (CH2), 31.4 (CH2), 30.0 (CH3), 23.5 (CH2), 22.5 (CH2), 13.9 (CH3). **HRMS** (ESI, m/z) calcd. mass 171.1385 [M+H], found 171.1412 [M+H].

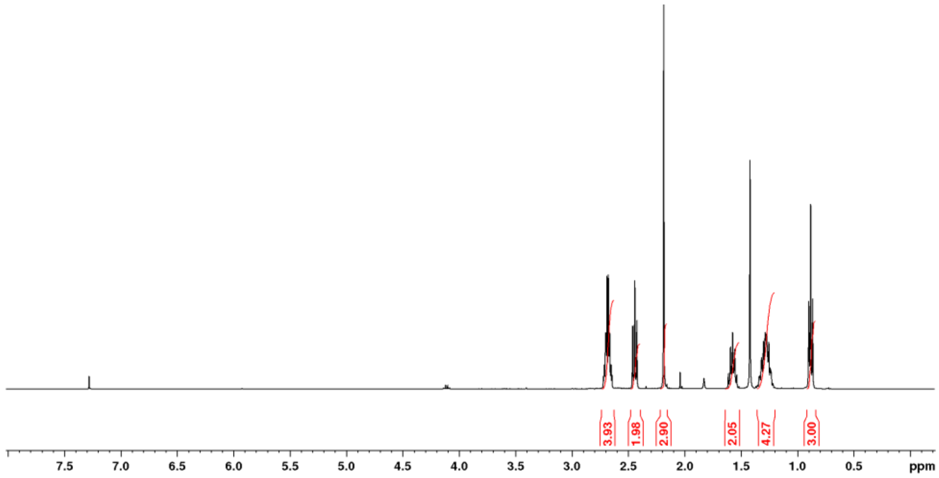

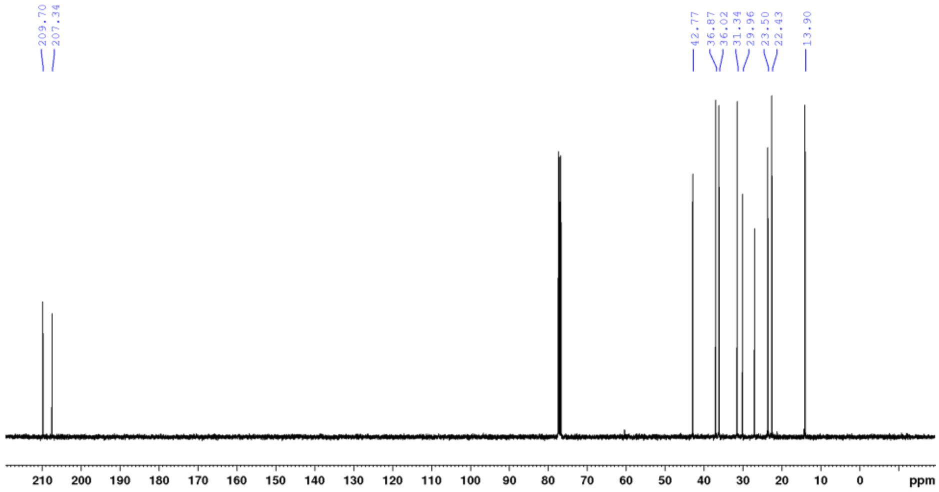

1-phenylpentane-1,4-dione (22)

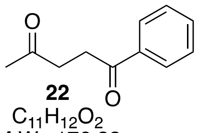

22
$C_{11}H_{12}O_2$
M.W.: 176.22

Acetophenone (2 mL, 17.1 mmol), 2 M LDA (10.6 mL) and chloroacetone (1.15 mL) were submitted to the General Procedure 6 affording diketone **22** as a pale-yellow oil i 75% yield (2.26 g). **1H NMR** (400.13 MHz, CDCl3): δ 7.98 (d, *J* = 7.5, 2H), 7.56 (t, *J* = 7.5, 1H), 7.46 (t, *J* = 7.5, 2H), 3.28 (t, *J* = 6.2, 2H), 2.89 (t, *J* = 6.2, 2H), 2.26 (s, 3H). **13C NMR** (101 MHz, CDCl3): δ 207.4, 198.5, 136.6, 133.2, 128.6, 128.1, 37.0, 32.4, 30.1. **HRMS** (ESI, m/z) calcd. mass 177.0916 [M+H]+, found 177.0940 [M+H]+.

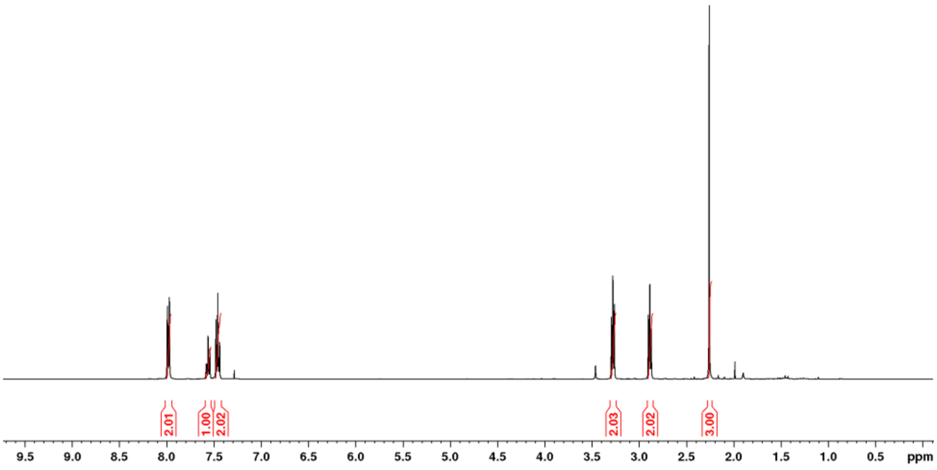

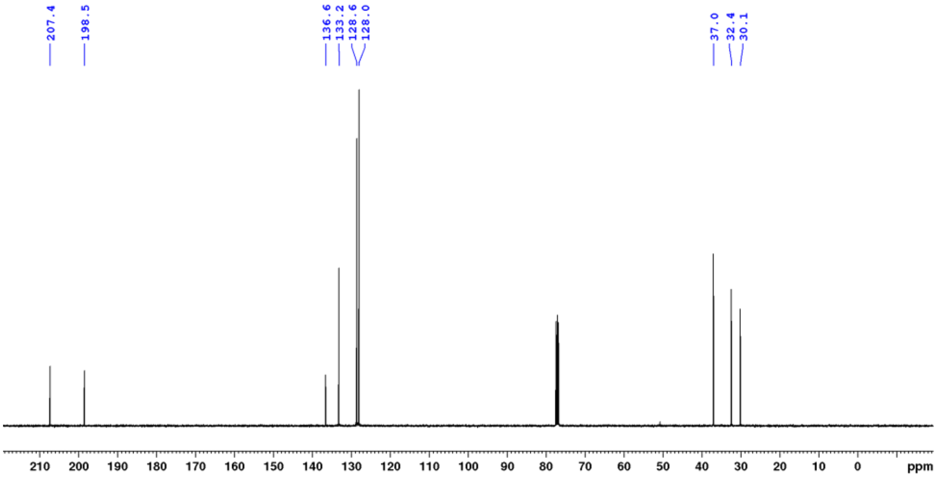

General procedure 7: Synthesis of *N*-methyl-2,5-disubstituted pyrrolidines

To a solution of diketone (**21** or **22**, 1 eq.) in methanol (5 mL) were added a solution of 2 M methylamine in methanol (**iii**, 1.2 eq), glacial acetic acid (1.2 eq.) and sodium cyanoborohydride ($NaBH_3CN$, 1.5 eq.). The resulting mixture was kept under stirring at room temperature for 18-24h. The reaction was then quenched by adding 1 M HCl (5 mL), stirring for 15 min and evaporation of the methanolic solvent. The obtained aqueous residue was then diluted with distilled water (5 mL), the pH was adjusted to 14 and the obtained aqueous solution was extracted with MTBE (3 x 10 mL). The organic phases were combined, dried over $MgSO_4$ anhydrous and concentrated under reduced pressure to afford the desired *N*-alkylated pyrrolidines without any further purification step.

1,2-dimethyl-5-pentylpyrrolidine (21iii)

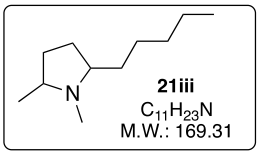

21 (100 mg, 0.59 mmol), 2 M methylamine in methanol (355 µL, 0.71 mmol), glacial acetic acid (41 µL, 0.71 mmol) and $NaBH_3CN$ (56 mg, 0.89 mmol) were submitted to general procedure 7 affording a 73:27 *cis:trans* mixture of **21iii** as a light yellow oil in 62% yield (62 mg). **1H NMR** (400.13 MHz, CDCl3): δ 2.12 + 2.00 (s, 3H, trans + cis), 1.98-0.9 (m, 15H), 0.88 + 0.76 (d, *J* = 0.8 Hz, 3H, cis + trans), 0.69-0.61 (m, 3H). **13C NMR** (101 MHz, CDCl3): δ 67.8 + 62.8 (cis + trans), 62.7 + 58.6 (cis + trans), 38.5-19.1 (13 signals, 8C), 15.4 + 14.0 (trans + cis). NMR signals are reported as cis/trans mixtures. ***m/z* (±ES)** 98 $[M-C_5H_{11}]^+$, 154 $[M-CH_3]^+$, 169

[M]$^+$.

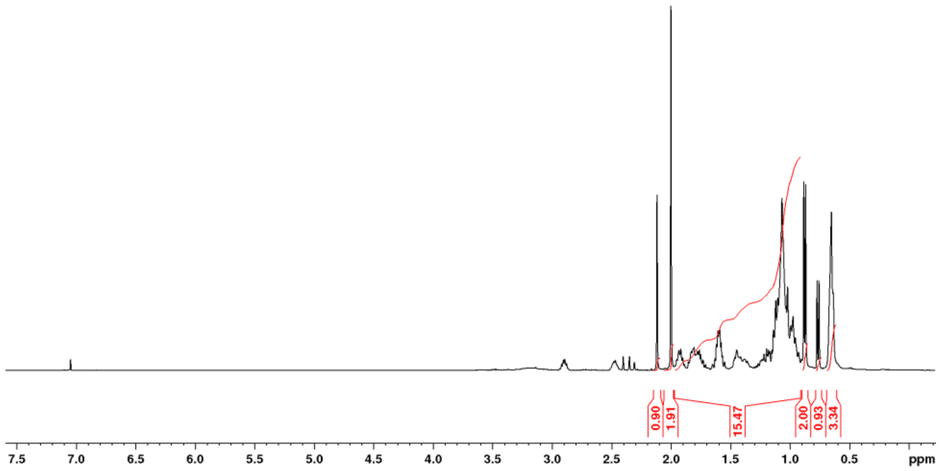

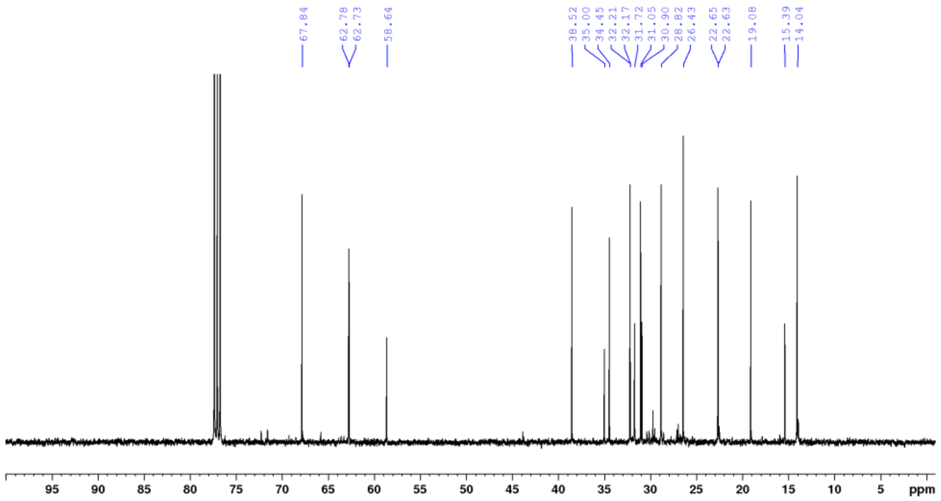

1,2-dimethyl-5-phenylpyrrolidine (22iii)

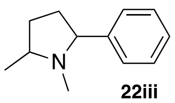

22iii
$C_{12}H_{17}N$
M.W.: 175.28

22 (100 mg, 0.57 mmol), 2 M methylamine in methanol (340 µL, 0.68 mmol), glacial acetic acid (39 µL, 0.68 mmol) and $NaBH_3CN$ (54 mg, 0.86 mmol) were submitted to general procedure 7 affording a 71:29 *cis:trans* mixture of **22iii** as a light yellow oil in 55% yield (55 mg). **1H NMR** (400.13 MHz, CDCl3): δ 7.32-7.01 (m, 5H), 3.61 + 3.06 (t, 1H, trans + cis), 2.05 + 2.01 (s, 3H, trans + cis), 3.33-3.25 + 2.36-2.08 + 2.00-1.38 (m, 5H), 1.11 + 0.95 (d, *J* = 6.2 Hz, 3H, cis + trans). **13C NMR** (101 MHz, CDCl3): δ 144.2 + 144.1 (cis + trans), 128.3-126.9 (6 signals, 5C), 77.6 + 67.0 (cis + trans), 62.3 + 58.4 (cis + trans), 35.6-31.8 (6 signals, 3C), 19.5 + 15.0 (cis + trans). NMR signals are reported as cis/trans mixtures. ***m/z* (±ES)** 98 [M-C_6H_5]$^+$, 160 [M-CH_3]$^+$, 175 [M]$^+$.

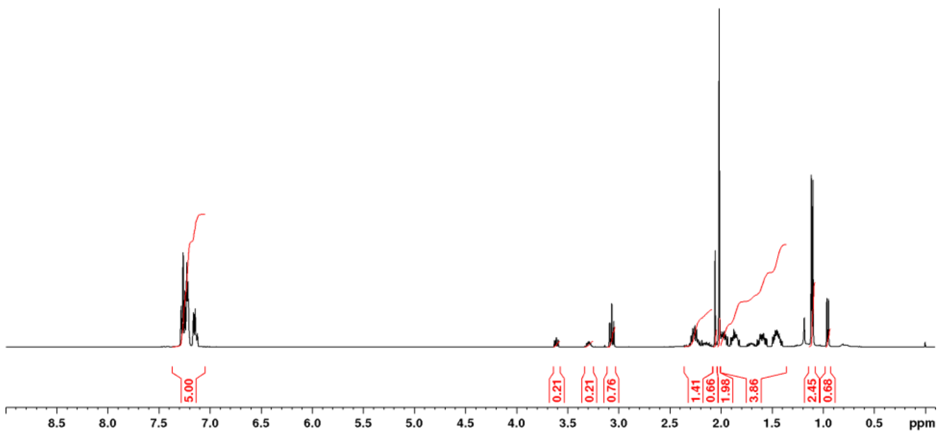

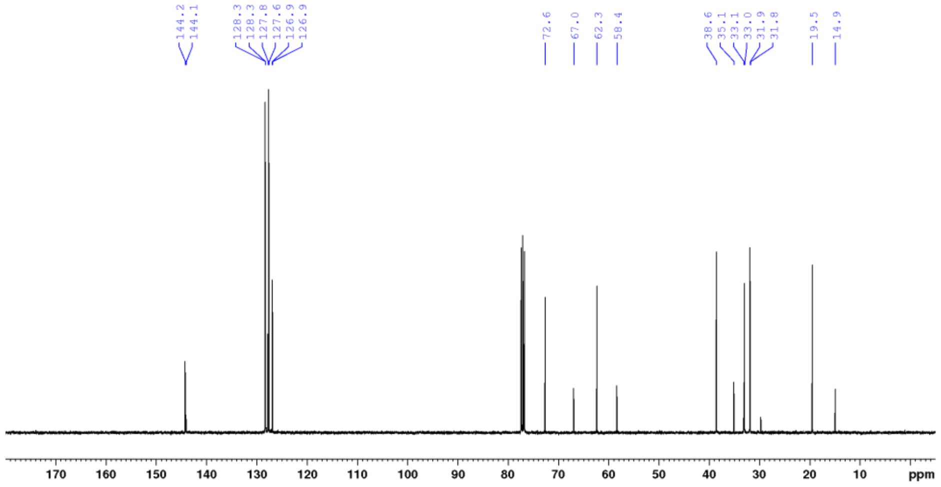

General procedure 8: Synthesis of *N*-methyl-2,5-disubstituted pyrrolidines - Allyl, cyclopropyl and propargyl derivatives

To a solution of the diketone (**21** or **22**, 1 eq.) in THF (5 mL) were added the respective alkyl amine (1.2 eq), glacial acetic acid (1.2 eq.) and sodium triacetoxyborohydride (NaBH(OAc)$_3$, 1.5 eq.). The resulting mixture was kept under stirring at room temperature for 18-24h. The reaction was then quenched by adding 1 M HCl (5 mL) and stirring for 15 min. The organic layer was separated and extracted with a further portion of 1 M HCl (5 mL). Both aqueous layers were combined, and the pH adjusted to 12, followed by extraction with MTBE (3 x 10 mL). The organic phases were combined, dried over MgSO$_4$ anhydrous and the solvent was removed under reduced pressure to afford the desired *N*-alkylated pyrrolidines without any further purification step.

1-cyclopropyl-2-methyl-5-pentylpyrrolidine (21i)

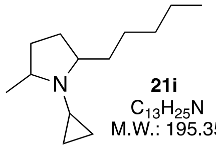

21i
$C_{13}H_{25}N$
M.W.: 195.35

21 (100 mg, 0.59 mmol), cyclopropylamine (49 µL, 0.71 mmol), glacial acetic acid (41 µL, 0.71 mmol) and NaBH(OAc)$_3$ (189 mg, 0.89 mmol) were submitted to general procedure 8 affording a 72:28 *cis:trans* mixture of **21i** as a light yellow oil in 58% yield (67 mg). **1H NMR** (400.13 MHz, CDCl3): δ 3.19-3.09 + 2.57-2.45 (m, 1H, *trans* + *cis*), 2.89-2.79 + 2.45-2.33 (m, 1H, *trans* + *cis*), 2.00-1.60 (m, 3H), 1.40-1.13 (m, 10H) 1.11 + 1.01 (d, *J* = 6.4 Hz, 3H, *cis* + *trans*), 0.82 (t, *J* = 6.8 Hz, 3H), 0.54-0.26 (m, 4H). **13C NMR** (101 MHz, CDCl3): δ 68.3 + 62.3 (*cis* +

trans), 63.2 + 57.7 (*cis* + *trans*), 35.0 – 14.0 (16 signals, 9C), 8.9 + 4.6 (*trans* + *cis*), 4.4 + 3.1 (*cis* + *trans*). NMR signals are reported as cis/trans mixtures. ***m/z* (±ES)** 124 $[M\text{-}C_6H_5]^+$, 195 $[M]^+$.

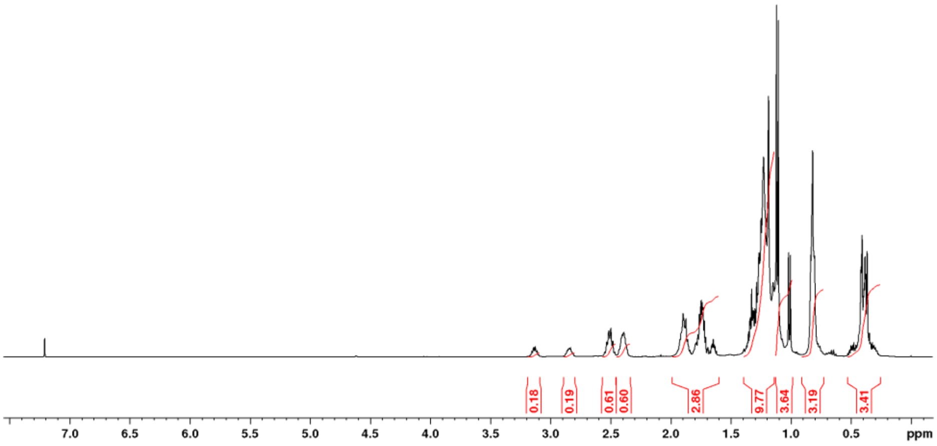

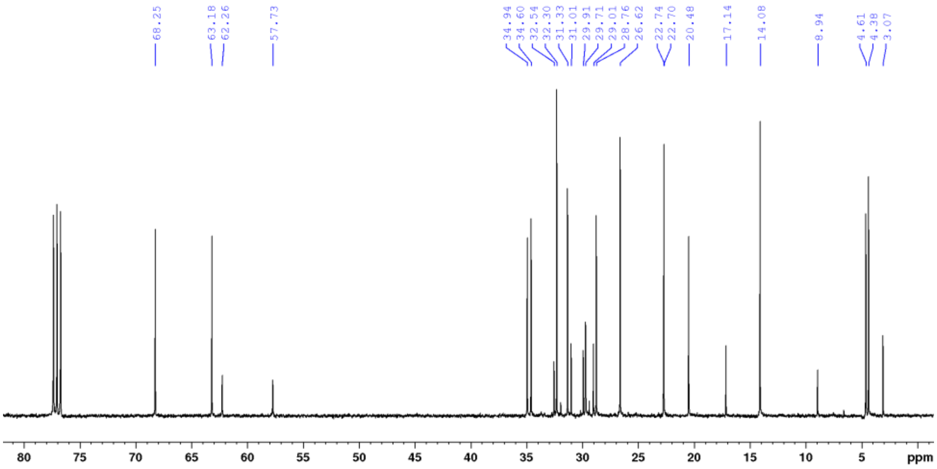

1-allyl-2-methyl-5-pentylpyrrolidine (21v)

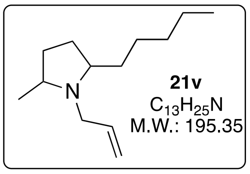

21 (100 mg, 0.59 mmol), allylamine (53 µL, 0.71 mmol), glacial acetic acid (39 µL, 0.68 mmol) and $NaBH(OAc)_3$ (189 mg, 0.89 mmol) were submitted to general procedure 8 affording a 61:39 *cis:trans* mixture of **21v** as a light yellow oil in 62% yield (72 mg). **1H NMR** (400.13 MHz, CDCl3): δ 5.92 – 5.80 (m, 1H), 5.17 – 5.98 (m, 2H), 3.30 – 1.07 (several multiplets, 16H), 1.02 + 0.87 (d, *J* = 6.2 Hz, 3H, cis + trans), 0.81 (t, *J* = 6.6 Hz, 3H). **13C NMR** (101 MHz, MHz, CDCl3): δ 137.1 + 135.3 (trans + cis), 116.8 + 116 (cis + trans), 63.7 + 60.5 (cis + trans), 58.8 + 55.7 (cis + trans), 53.6 + 51.3 (cis + trans), 35.5 – 20.0 (12 signals, 7C), 15.3 + 14.1 (trans + cis). NMR signals are reported as cis/trans mixtures. ***m/z* (±ES)** 124 $[M\text{-}C_5H_{11}]^+$, 180 $[M\text{-}CH_3]^+$, 195 $[M]^+$.

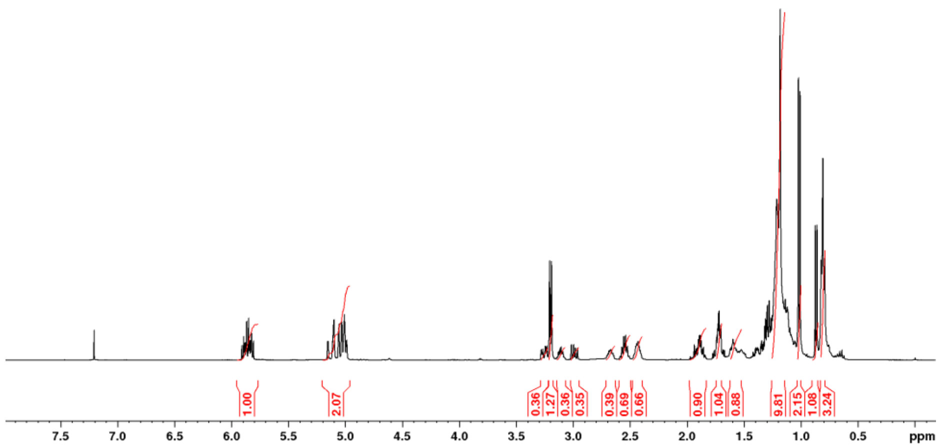

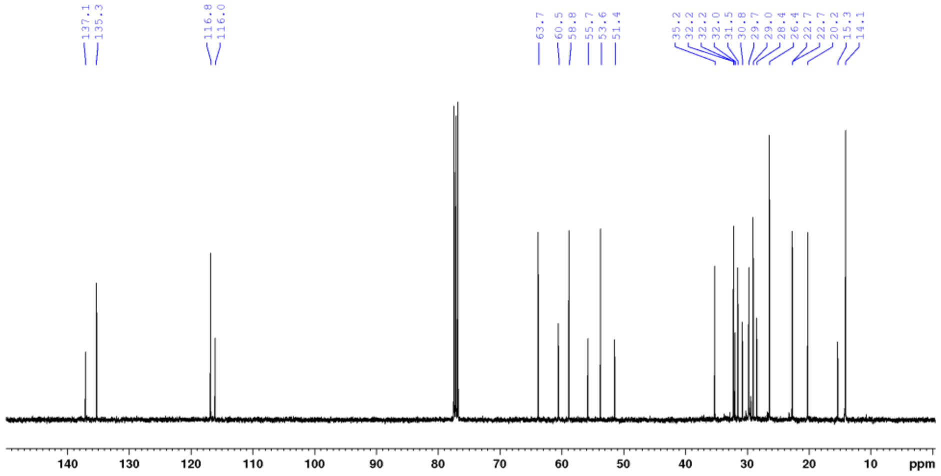

2-methyl-5-pentyl-1-(prop-2-yn-1-yl)pyrrolidine (21vi)

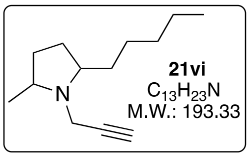

21 (100 mg, 0.59 mmol), propargylamine (46 µL, 0.71 mmol), glacial acetic acid (41 µL, 0.71 mmol) and $NaBH(OAc)_3$ (189 mg, 0.89 mmol) were submitted to general procedure 8 affording a 60:40 *cis:trans* mixture of **21vi** as a light yellow oil in 46% yield (53 mg). **1H NMR** (400.13 MHz, CDCl3): δ 3.60 – 1.10 (12 multiplets, corresponding to 17H), 1.07 + 1.02 (d, *J* = 6.2 Hz, 3H), 0.88 (t, *J* = 6.5 Hz, 3H). **13C NMR** (101 MHz, CDCl3): δ 81.5 + 78.3 (trans + cis), 72.4 + 71.3 (cis + trans), 61.5 + 60.2 (cis + trans), 56.7 + 56.3 (cis + trans), 37.5 – 18.5 (14 signals, 18C), 16.1 + 14.1 (trans + cis). NMR signals are reported as cis/trans mixtures. ***m/z*** **(±ES)** 122 $[M-C_5H_{11}]^+$, 178 $[M-CH_3]^+$, 193 $[M]^+$.

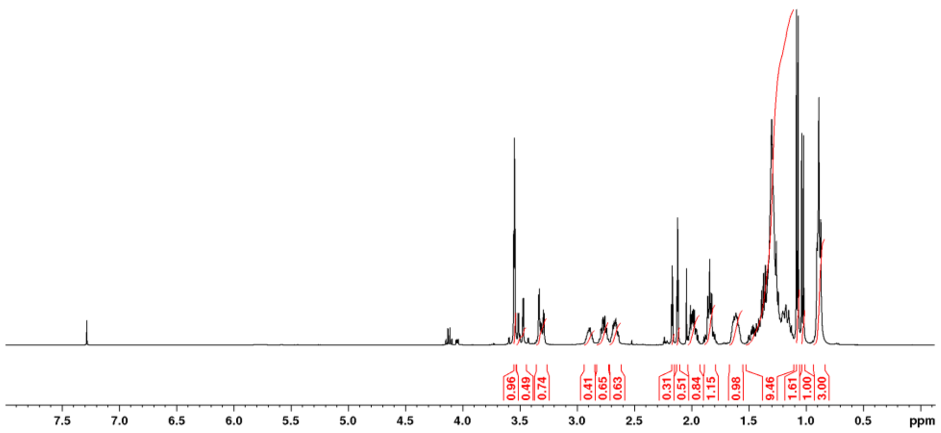

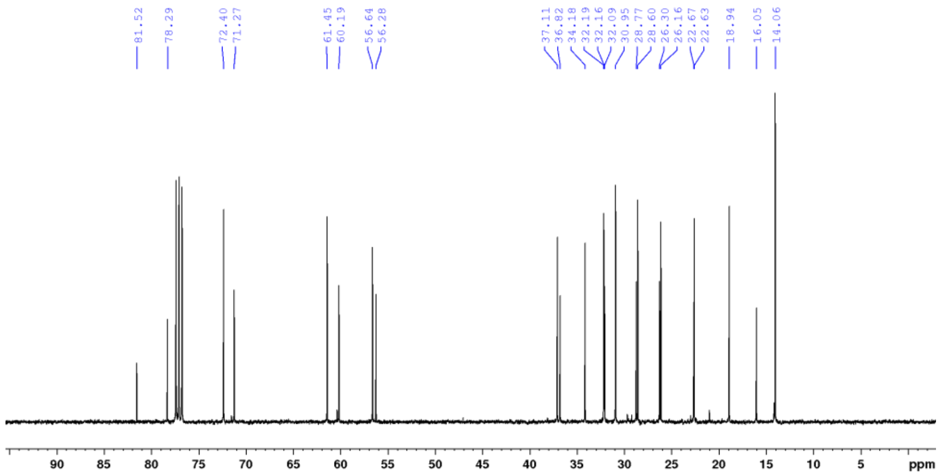

1-cyclopropyl-2-methyl-5-phenylpyrrolidine (22i)

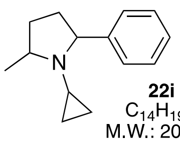

22i
$C_{14}H_{19}N$
M.W.: 201.31

22 (100 mg, 0.57 mmol), cyclopropylamine (47 µL, 0.68 mmol), glacial acetic acid (39 µL, 0.68 mmol) and $NaBH(OAc)_3$ (182 mg, 0.86 mmol) were submitted to general procedure 8 affording a 56:44 *cis:trans* mixture of **22i** as a light yellow oil in 64% yield (74 mg). **1H NMR** (400.13 MHz, CDCl3): δ 7.45 – 7.23 (m, 5H), 4.24 – 1.50 (9 multiplets, 7H), 1.32 + 1.24 (d, *J* = 6.2 Hz, 3H), 0.49 – (-0.29) (3 multiplets, 4H). **13C NMR** (101 MHz, CDCl3): δ 146.3 + 144.2 (cis + trans), 128.5 – 126.5 (4 signals, 5C), 71.7 + 66.8 (cis + trans), 62.9 + 57.9 (cis + trans), 35.5 – 5.0 (11 peaks, 10C), 3.7 + 3.4 (trans + cis). NMR signals are reported as cis/trans mixtures. ***m/z* (±ES)** 186 $[M-CH_3]^+$, 200 $[M-H]^+$, 201 $[M]^+$.

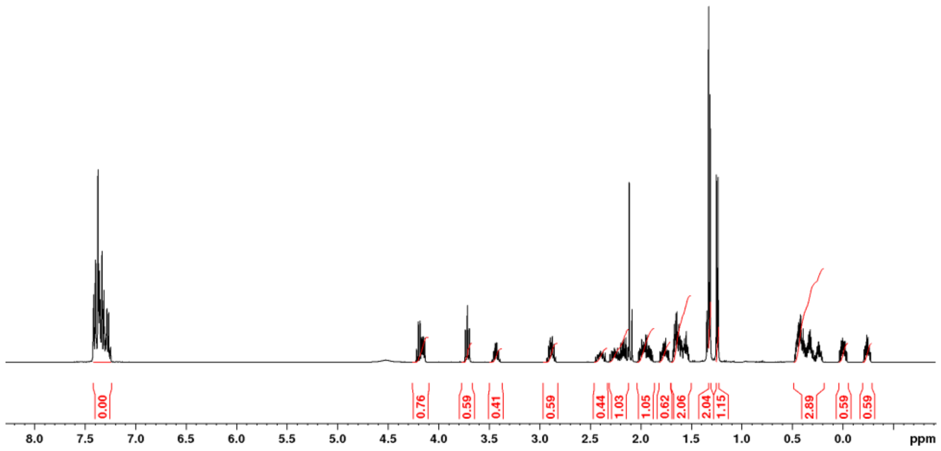

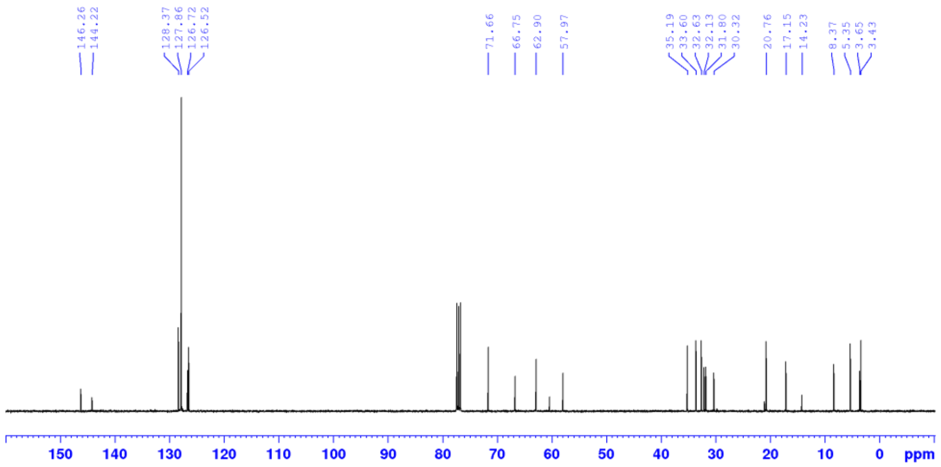

1-allyl-2-methyl-5-phenylpyrrolidine (22v)

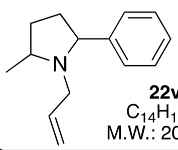

22v
$C_{14}H_{19}N$
M.W.: 201.31

22 (100 mg, 0.57 mmol), allylamine (51 µL, 0.68 mmol), glacial acetic acid (39 µL, 0.68 mmol) and $NaBH(OAc)_3$ (182 mg, 0.86 mmol) were submitted to general procedure 8 affording a 55:45 *cis:trans* mixture of **22v** as a light yellow oil in 84% yield (97 mg). **1H NMR** (400.13 MHz, CDCl3): δ 7.46 – 7.21(m, 5H), 5.93 – 5.78 (m, 1H), 5.20 – 5.01 (m, 2H), 3.85 – 1.49 (11 multiplets, 8H), 1.23 + 1.04 (d, J = 6.2 Hz, 3H). **13C NMR** (101 MHz, CDCl3): δ 145.0 – 115.5 (12 signals, 8C), 68.5 – 14.0 (12 signals, 6C). NMR signals are reported as cis/trans mixtures. ***m/z* (±ES)** 124 $[M-C_6H_5]^+$, 186 $[M-CH_3]^+$, 201 $[M]^+$.

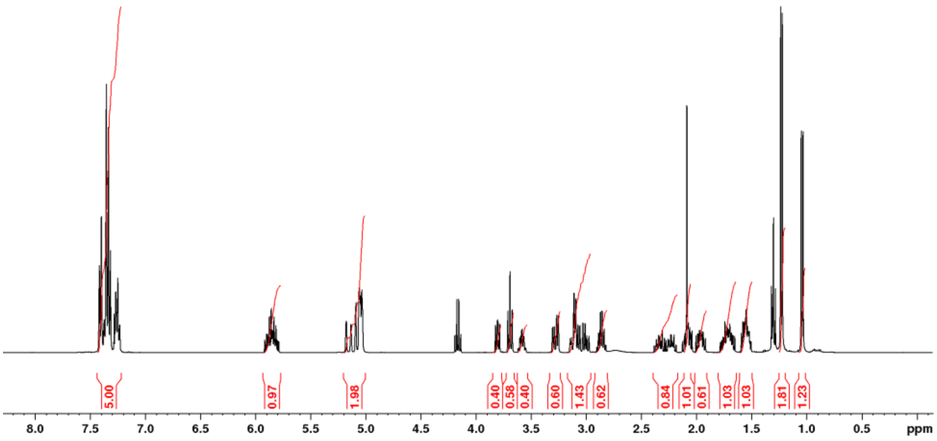

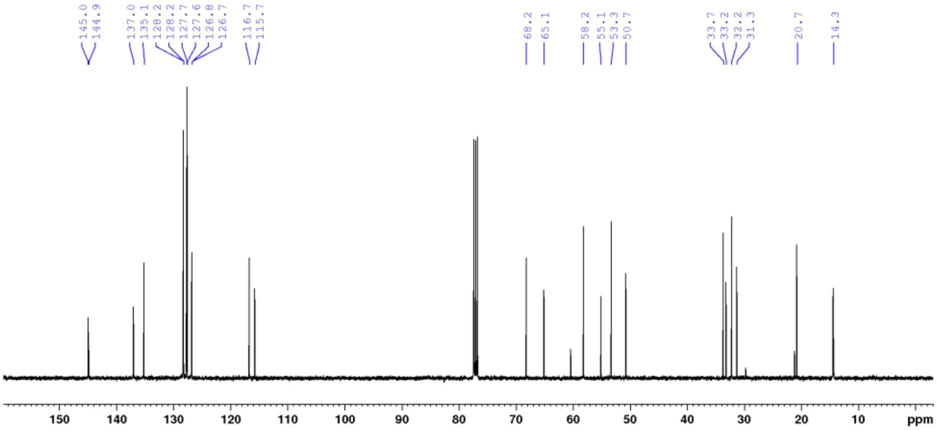

2-methyl-5-phenyl-1-(prop-2-yn-1-yl)pyrrolidine (22vi)

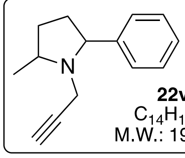

22vi
$C_{14}H_{17}N$
M.W.: 199.30

22 (100 mg, 0.57 mmol), cyclopropylamine (43 µL, 0.68 mmol), glacial acetic acid (39 µL, 0.68 mmol) and $NaBH(OAc)_3$ (182 mg, 0.86 mmol) were submitted to general procedure 8 affording a 52:47 *cis:trans* mixture of **22vi** as a light yellow oil in 46% yield (52 mg). **1H NMR** (400.13 MHz, CDCl3): δ 7.43 – 7.24 (m, 5H), 3.93 – 1.51 (14 multiplets, 9H), 1.20 + 1.15 (d, J = 6.1 and 6.4 Hz, 3H). **13C NMR** (101 MHz, CDCl3): δ 143.5 – 127.0 (8 signals, 6C), 81.6 + 78.3 (trans + cis), 72.6 – 31.4 (12 signals, 6C), 19.2 + 15.3 (cis + trans). NMR signals are reported as cis/trans mixtures. ***m/z* (±ES)** 122 $[M-C_6H_5]^+$, 184 $[M-CH_3]^+$, 199 $[M]^+$.

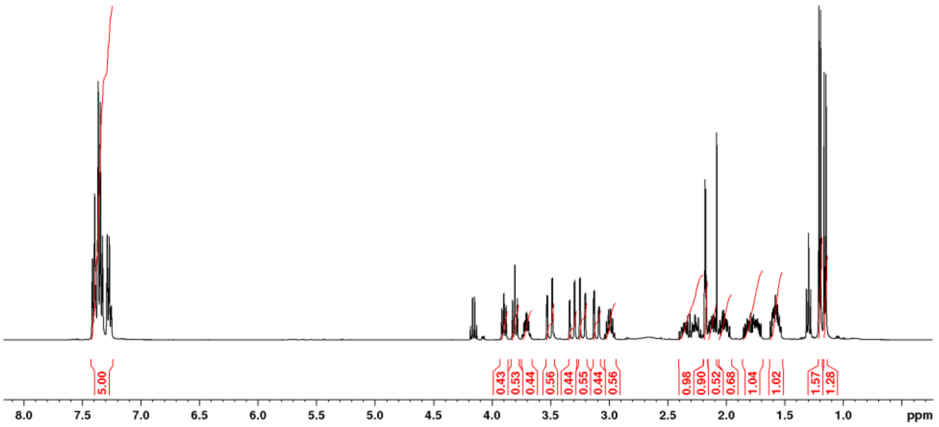

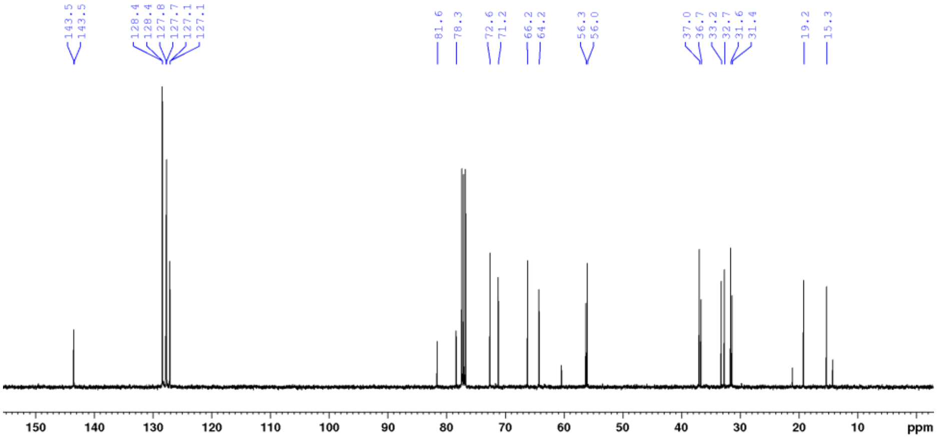

Specific Activity Screens

Diols

Substrate	SA AcCO wt	SA AcCO$_6$
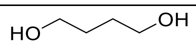	12.27	254.28
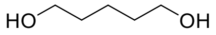	12.98	383.67
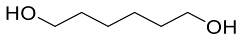	79.82	566.65
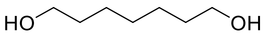	61.08	620.01
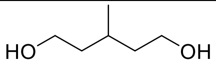	20.34	231.68
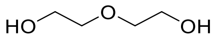	0	48.78
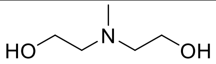	27.51	0
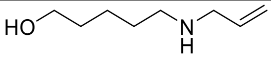	20.57	2.10
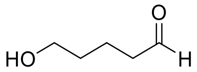	0.39	32.87

Keto-Alcohols

Substrate	SA AcCO wt (mU / mg)	SA $AcCO_6$ (mU / mg)
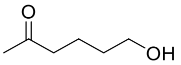	4.58	92.75
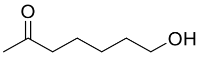	39.52	138.70
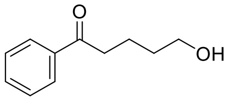	0.52	8.09
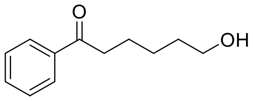	59.00	40.82
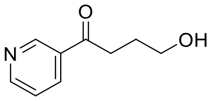	3.44	1.75

Glucose Dehydrogenase Side Reaction

As the use of the commercially available GDH CDX-901 caused terminal diol cyclisation reactions to fail, the specific activity of the CFE was measured in the presence of the intermediate 5-hydroxypentanal to test the hypothesis that the lactol tautomer may be subject to oxidation by this enzyme. Activity was observed with the CDX-901 lysate, but not with the alternative *Ta*GDH CFE or the empty pet-28b CFE control.

Substrate	SA CDX-901 CFE (mU / mg)	SA *Ta*GDH CFE (mU / mg)	SA Empty pET-28b CFE (mU / mg)
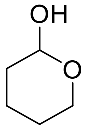	21.26	0	0

<u>GC-MS Traces</u>

$AcCO_6$/*Ad*RedAm-catalysed Cyclisation

GC-MS method: Agilent HP-1ms column (30 m × 0.32 mm × 0.25 µm), 1 mL·min^{-1}, 50 to 200 °C at 5 °C·min^{-1}, then hold for 2 min.

1-Propylpyrrole

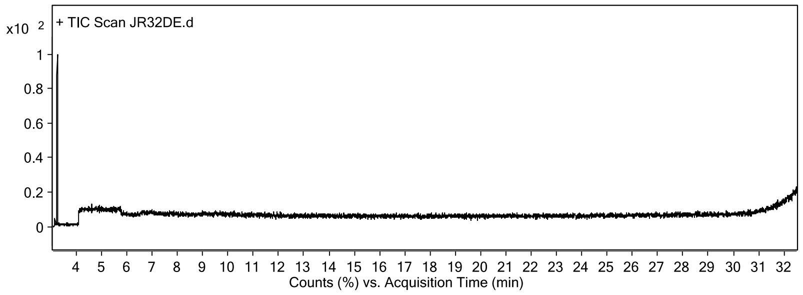

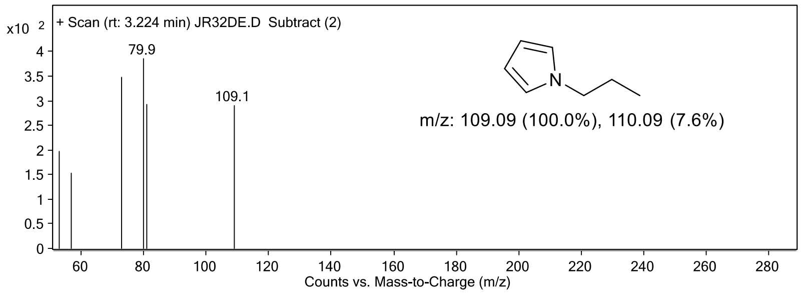

1-Allylpyrrole

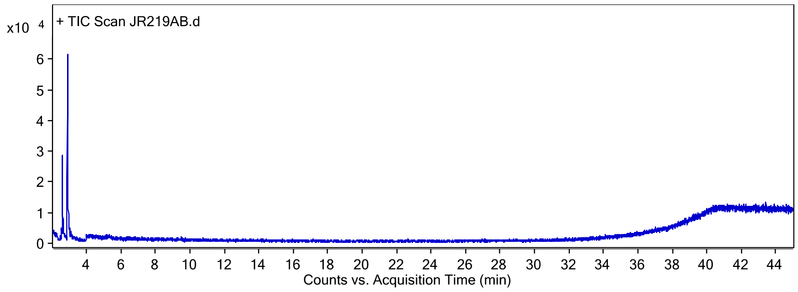

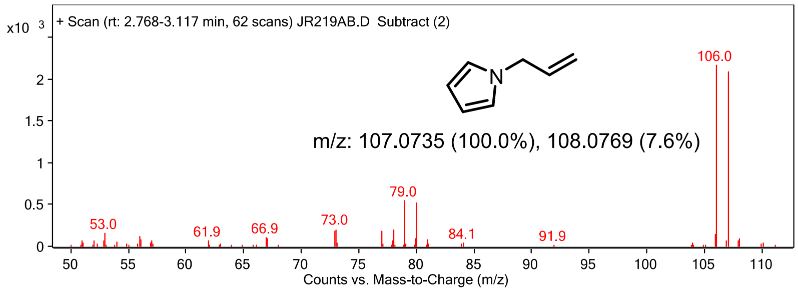

1-Cyclopropylpyrrole

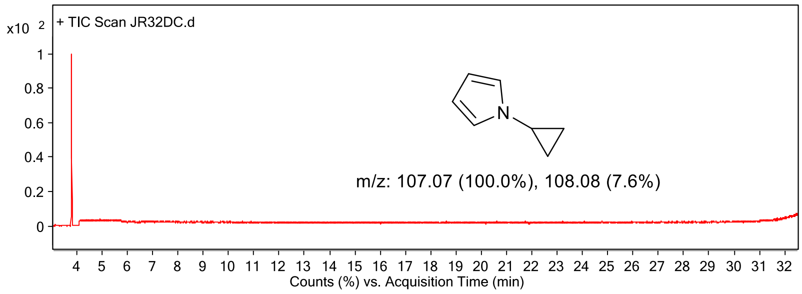

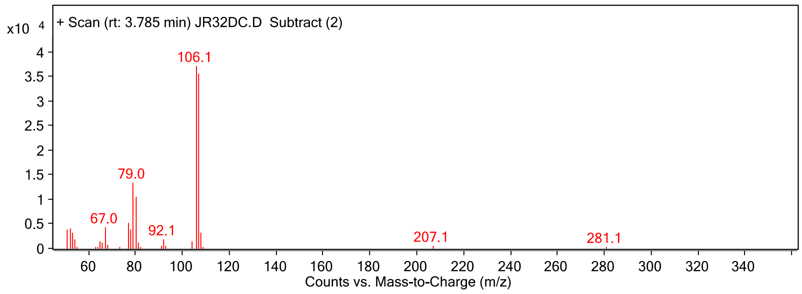

1-Propargylpyrrolidine

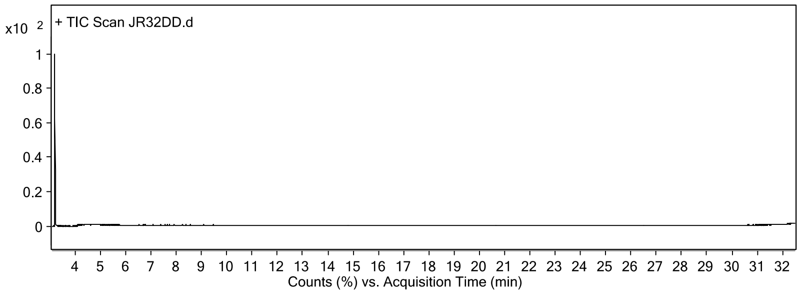

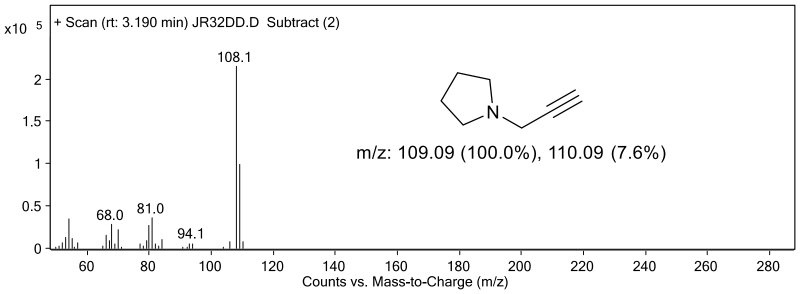

1-Benzylpyrrole

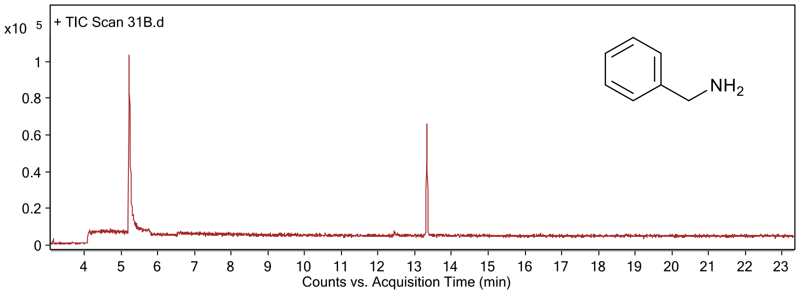

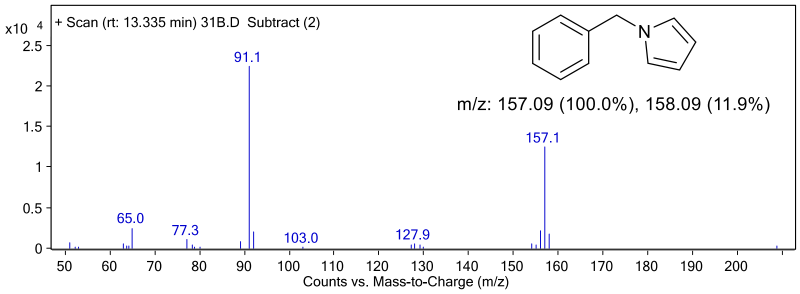

Chemical Standard:

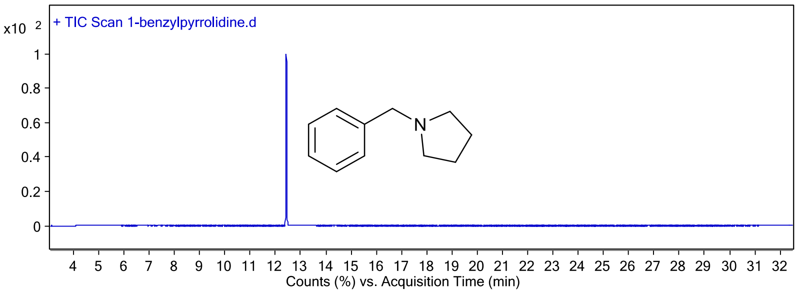

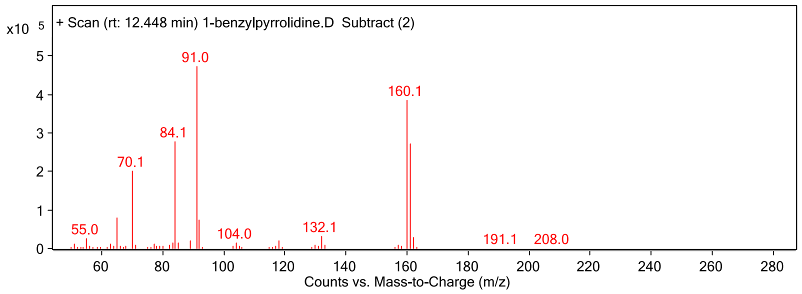

1-(3-Chloropropyl)-pyrrole

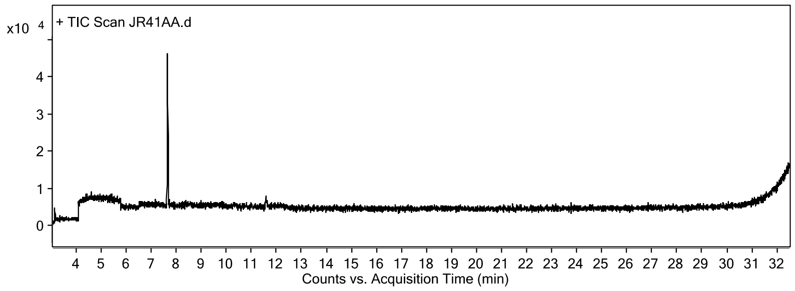

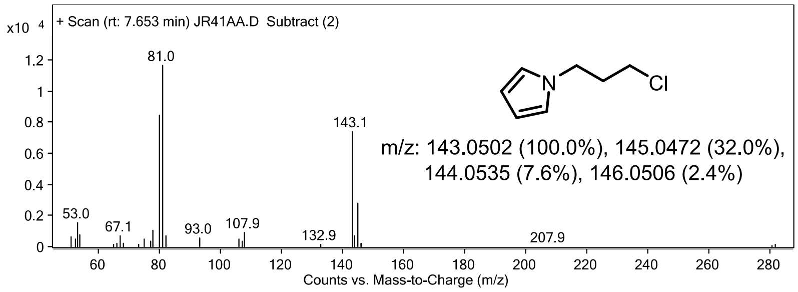

No RedAm Control

A control reaction was run in which *Ad*RedAm was excluded to analyse whether the reductive aminase plays a role in the formation of electronically equivalent pyrrole derivatives. GC Analysis showed that 1-benzylpyrrole does not form in significant amount when *Ad*RedAm is not present.

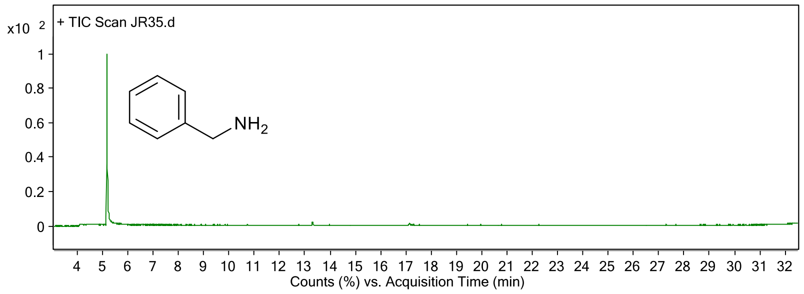

1-Propylpiperidine

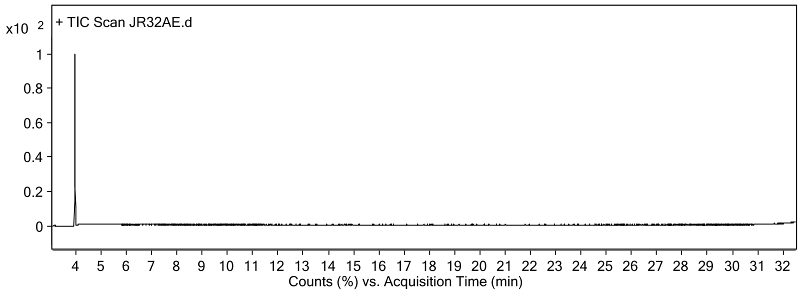

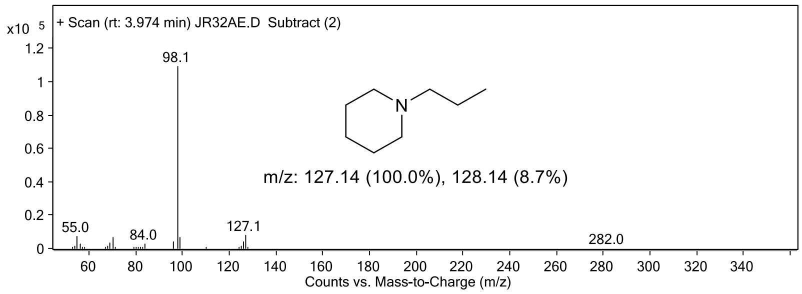

1-Allylpiperidine

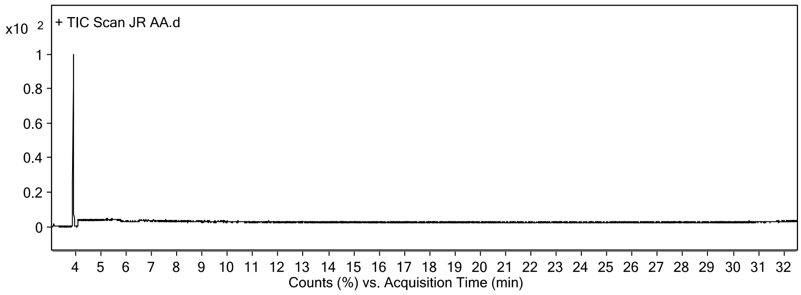

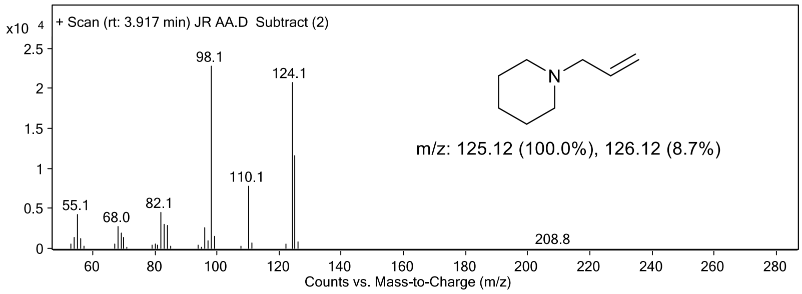

1-Propargylpiperidine

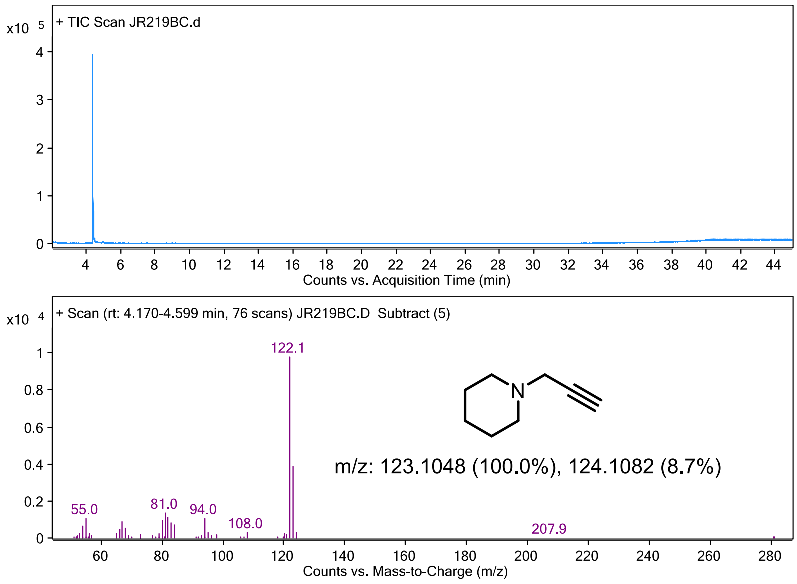

1-Cyclopropylpiperidine

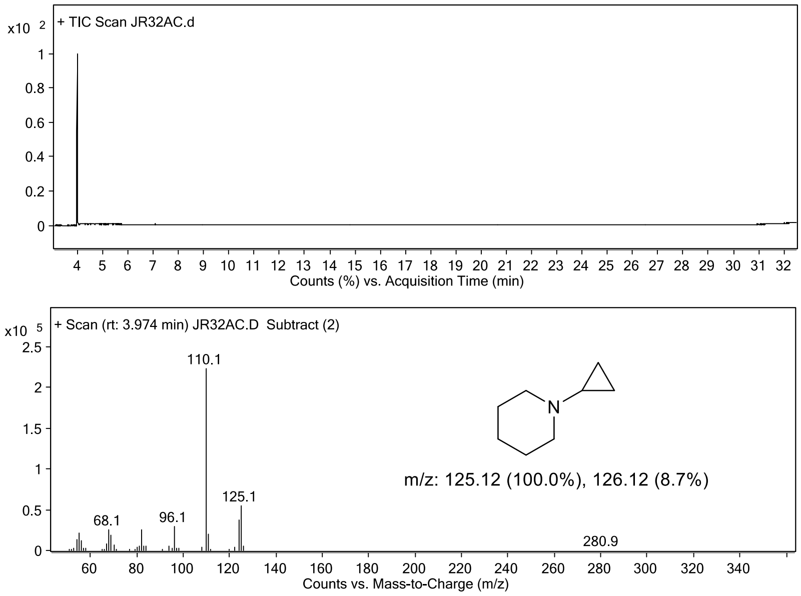

1-Benzylpiperidine

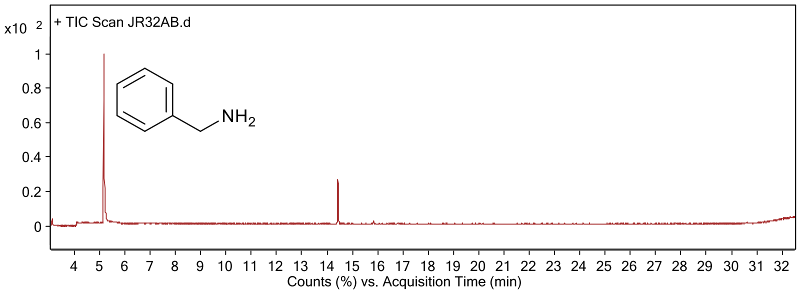

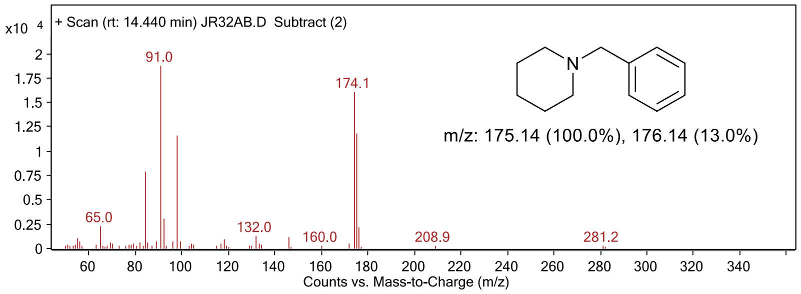

1-(3-Chloropropyl)piperidine

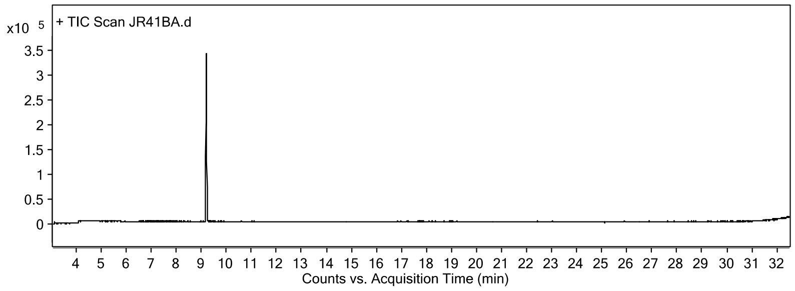

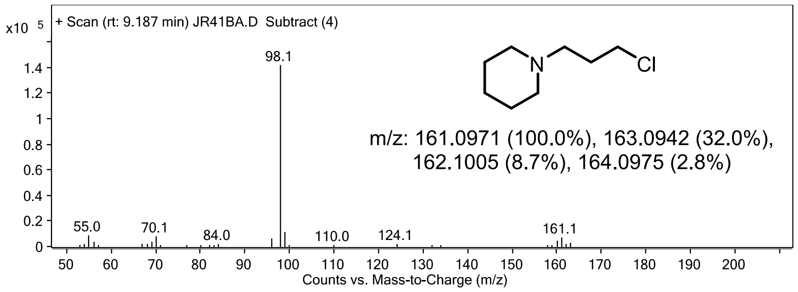

1-Propyl-4-methylpiperidine

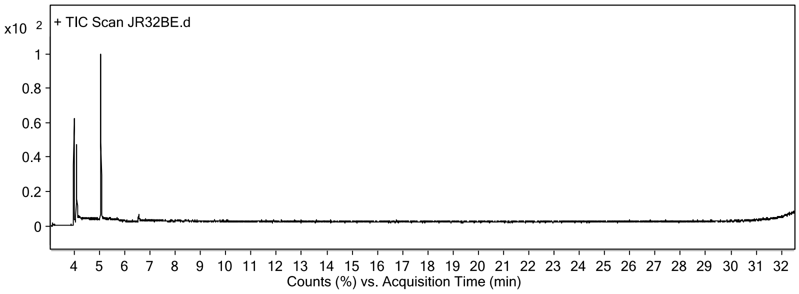

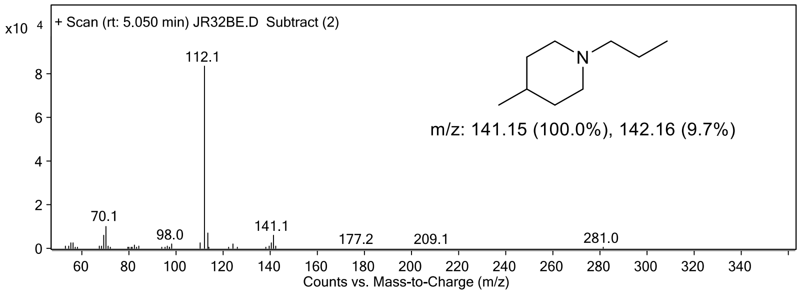

1-Allyl-Methylpiperidine

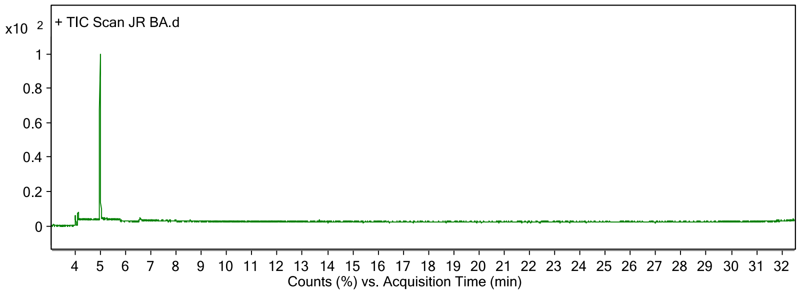

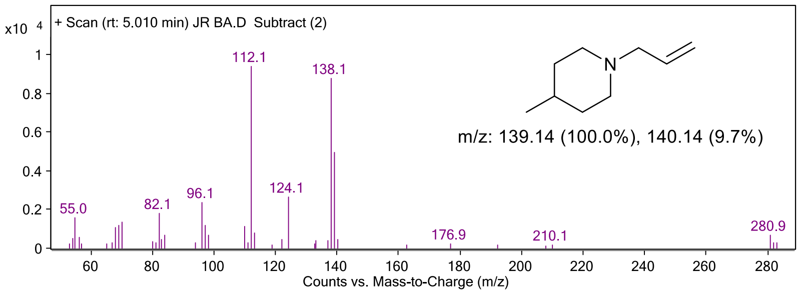

1-Cyclopropyl-4-methylpiperidine

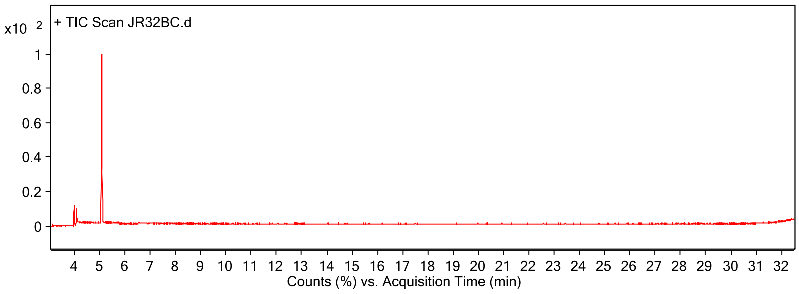

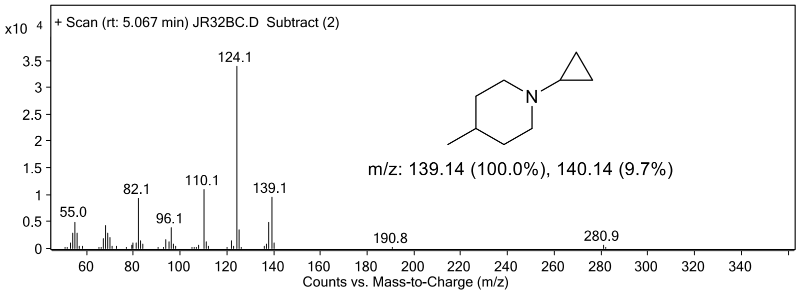

1-Propargyl-4-Methylpiperidine

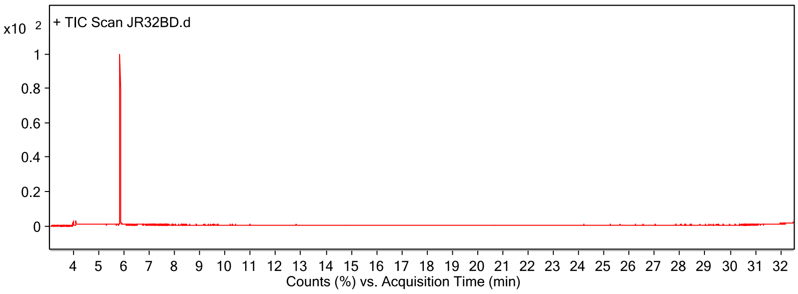

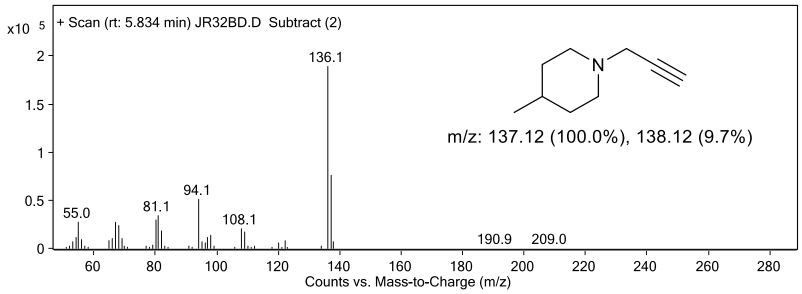

1-Benzyl-4-methylpiperidine

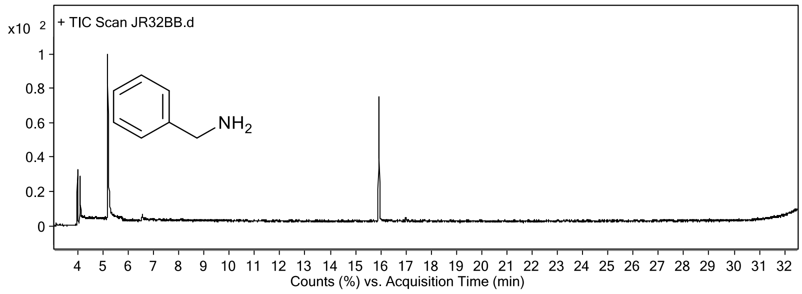

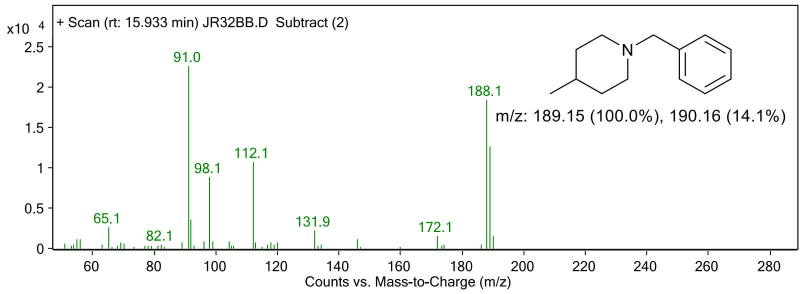

1,4-Methylpiperidine

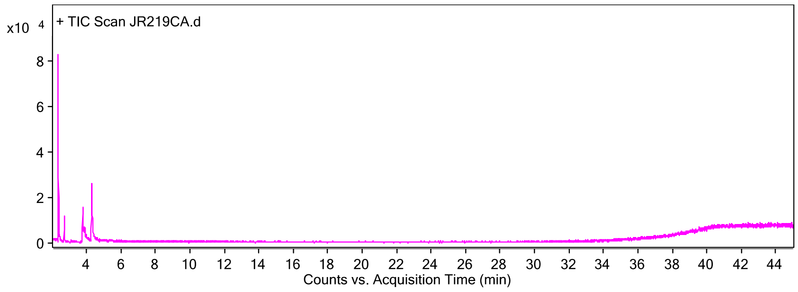

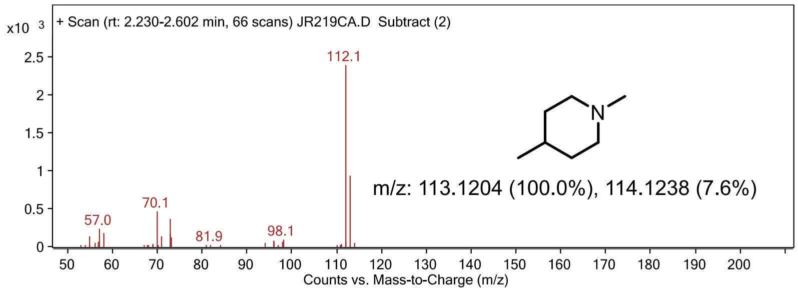

1-(3-Chloropropyl)-4-methylpiperidine

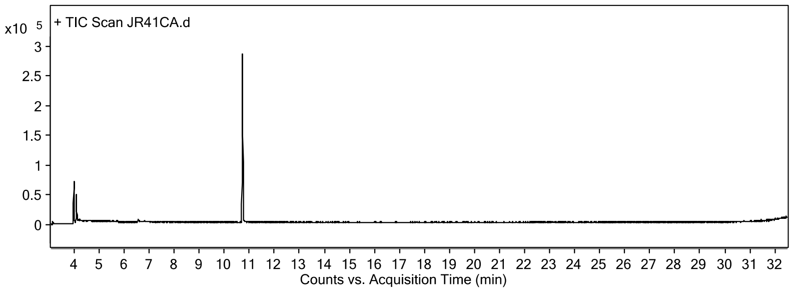

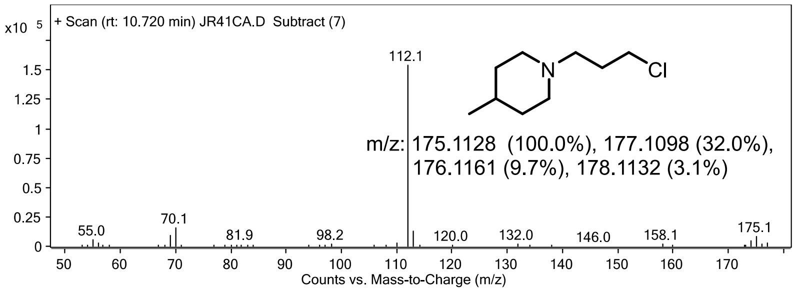

1-Propylazepane

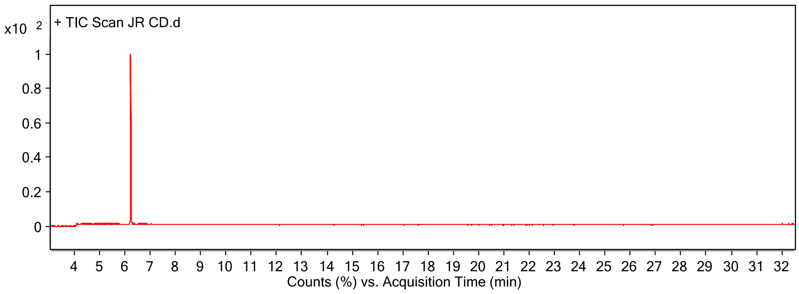

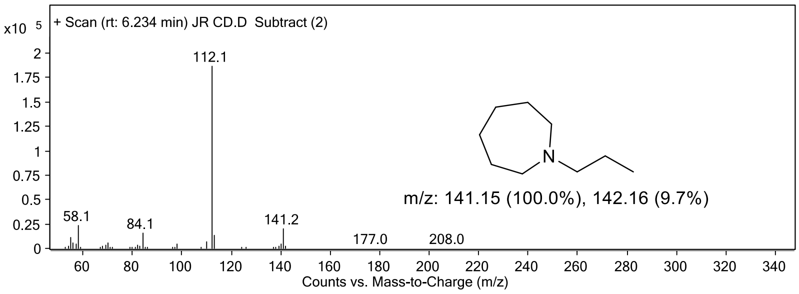

1-Allylazepane

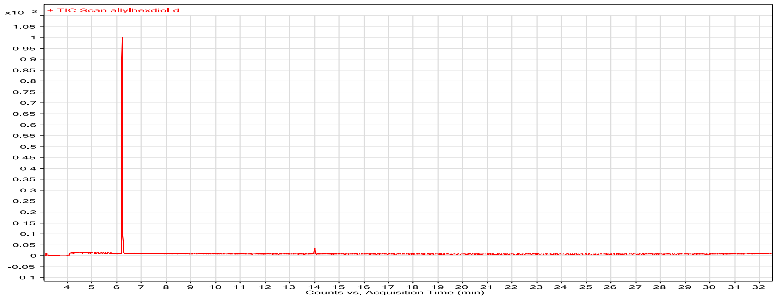

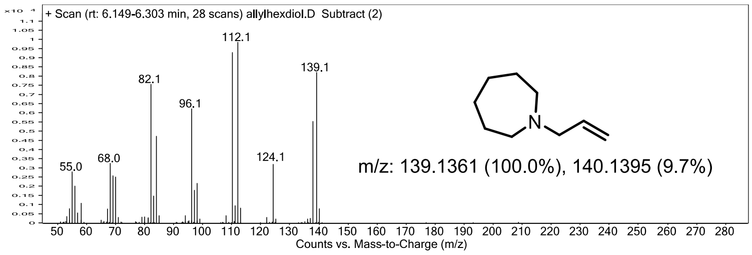

1-Cyclopropylazepane

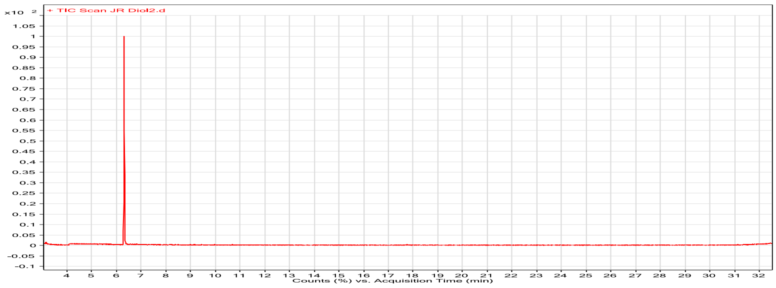

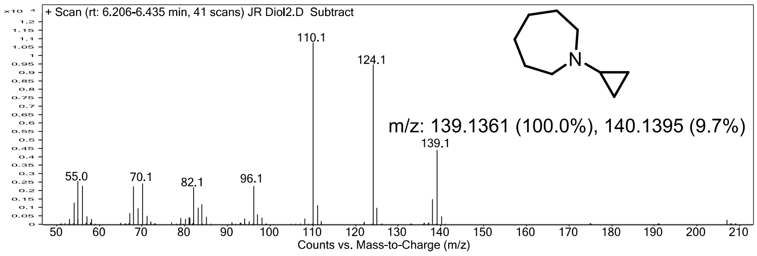

1-Propargylazepane

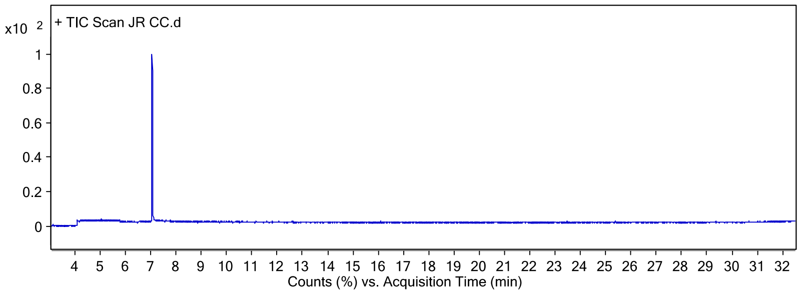

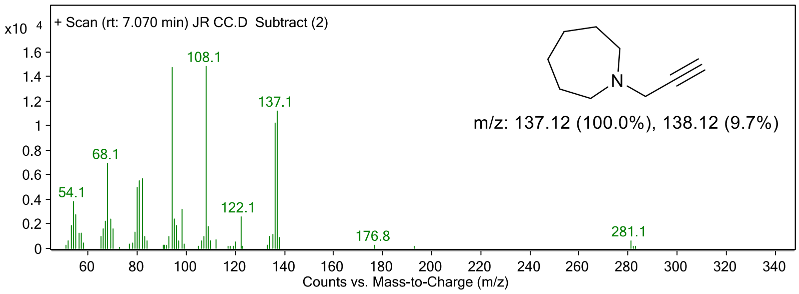

1-Benzylazepane

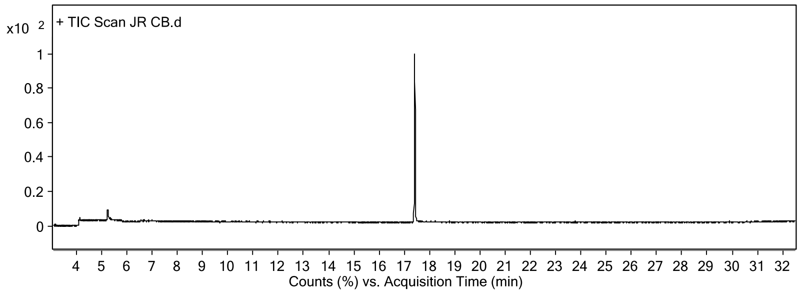

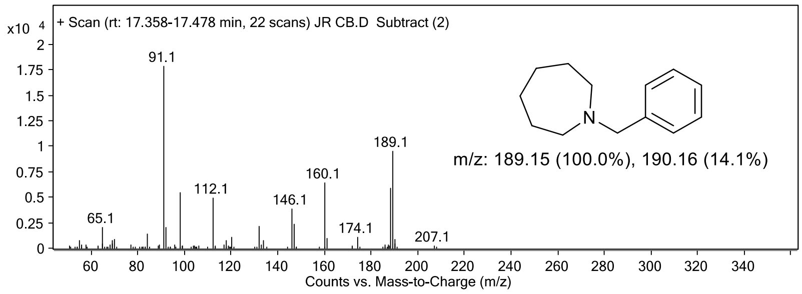

1-Methylazepane

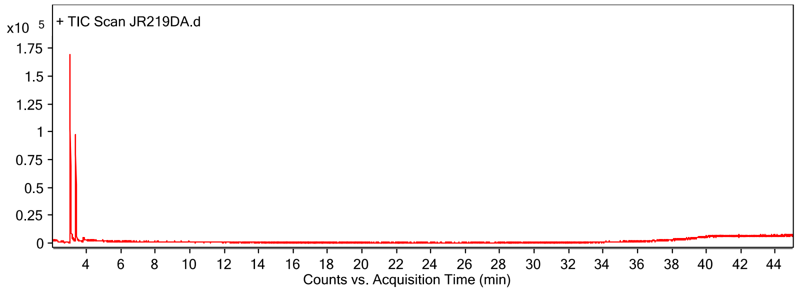

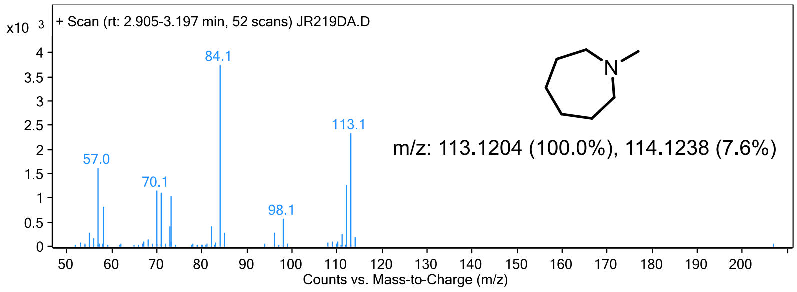

1-(3-Chloropropyl)azepane

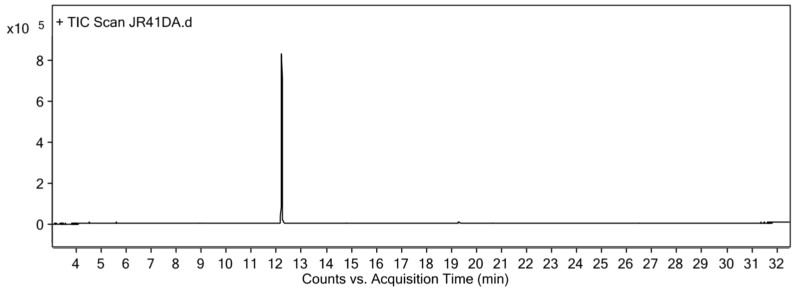

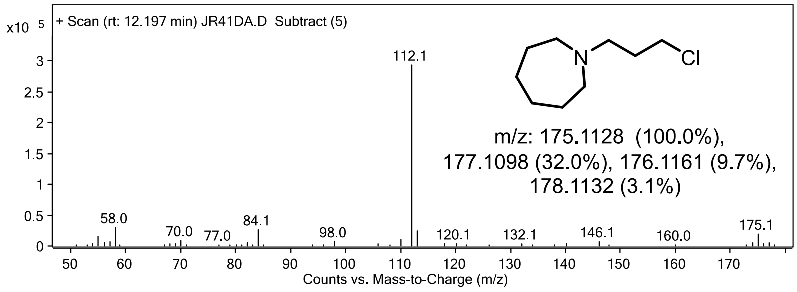

1-Propylazocane

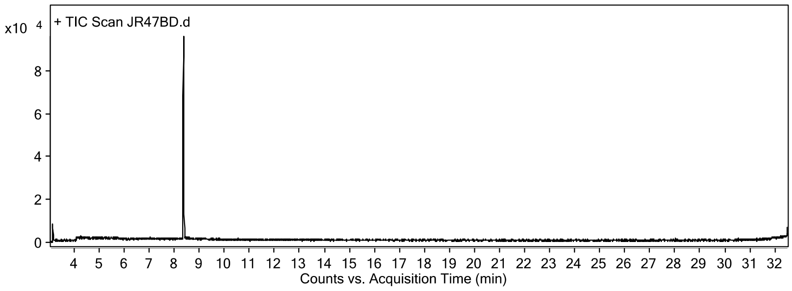

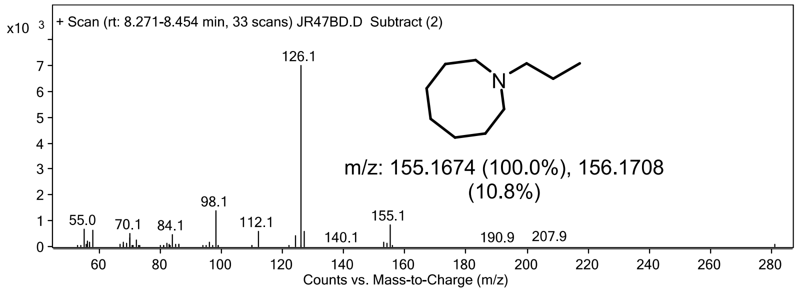

1-Allylazocane

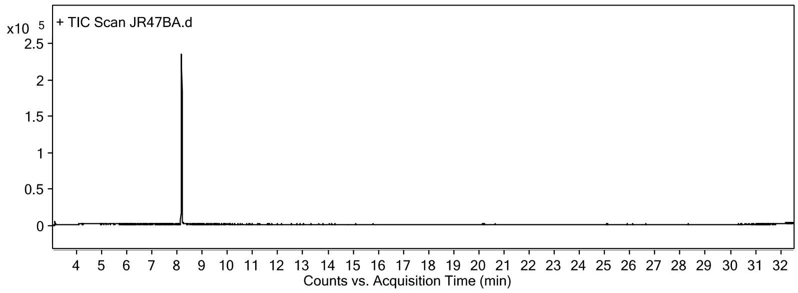

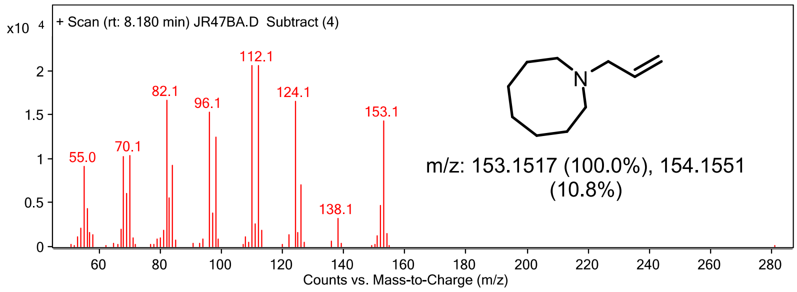

1-Benzylazocane

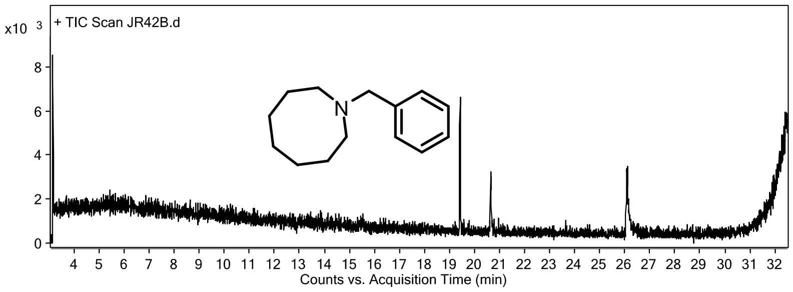

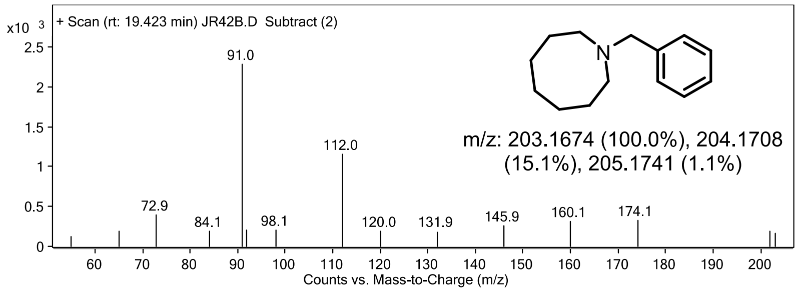

1-(3-Chloropropyl)azocane

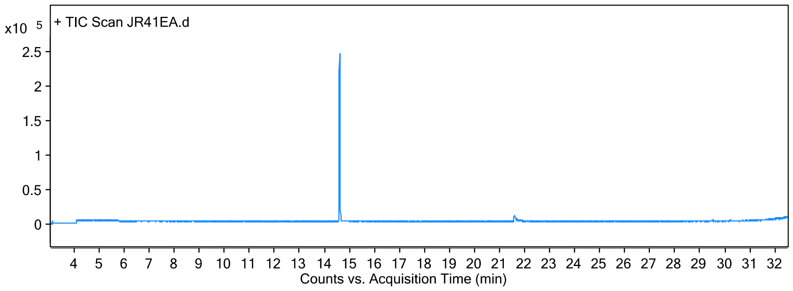

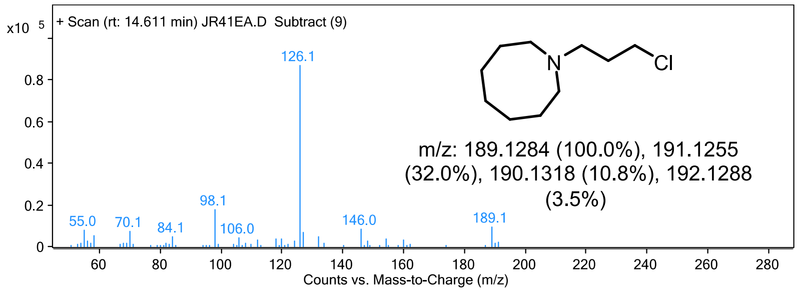

1,2-dimethylpiperidine

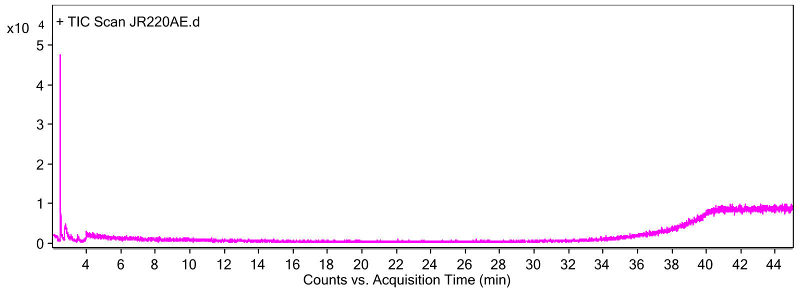

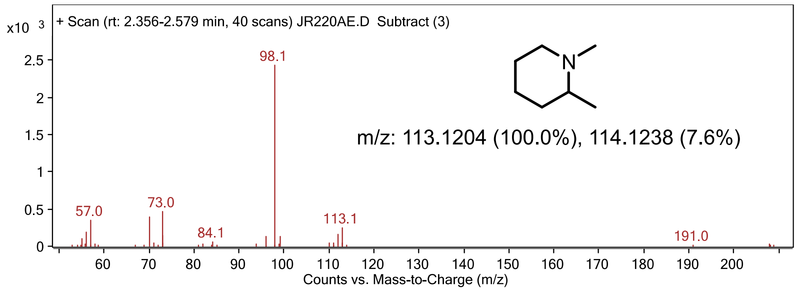

N-propyl-2-methylpiperidine

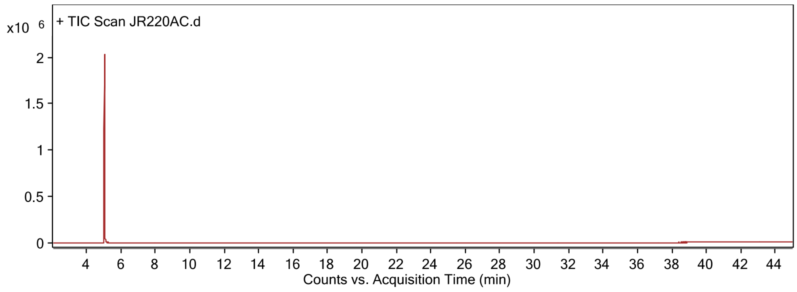

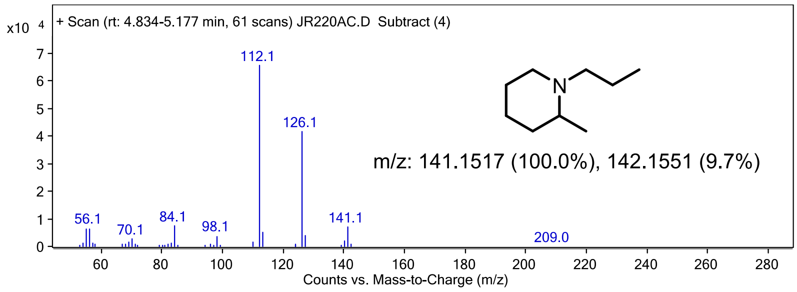

N-allyl-2-methylpiperidine

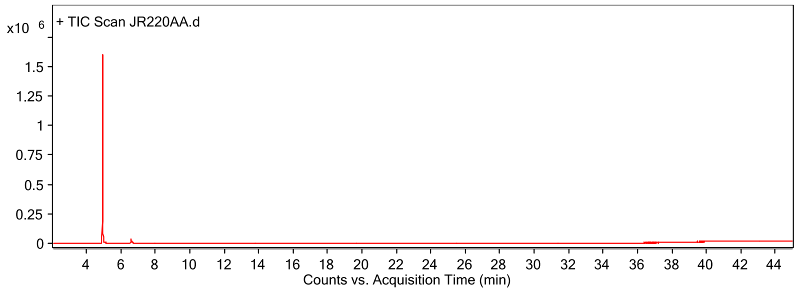

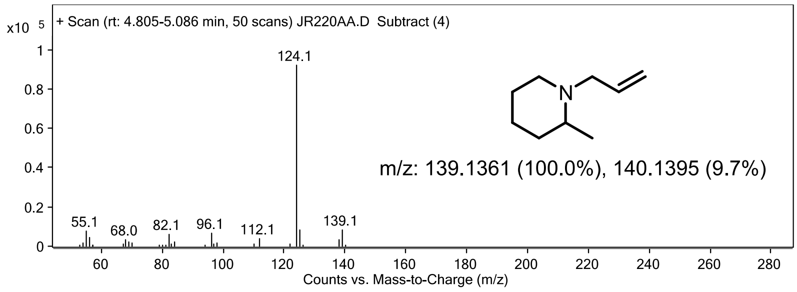

N-propargyl-2-methylpiperidine

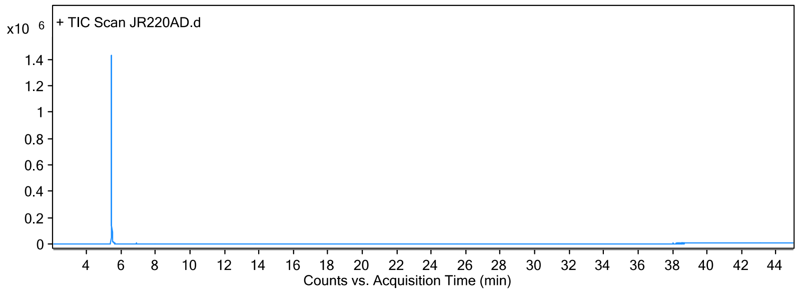

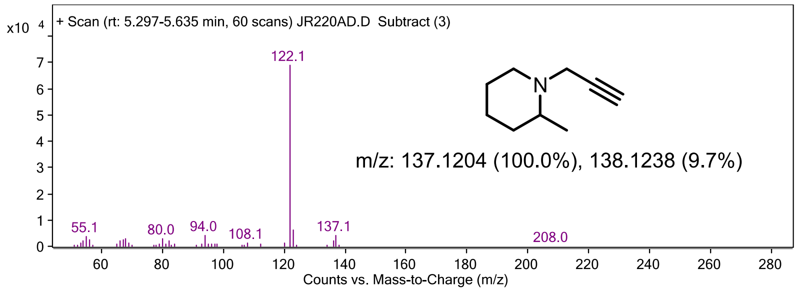

N-cyclopropyl-2-methylpiperidine

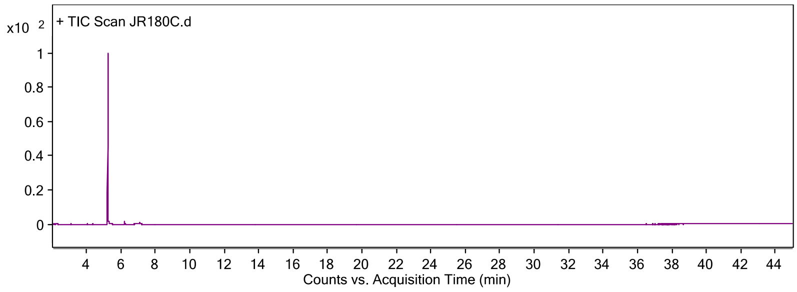

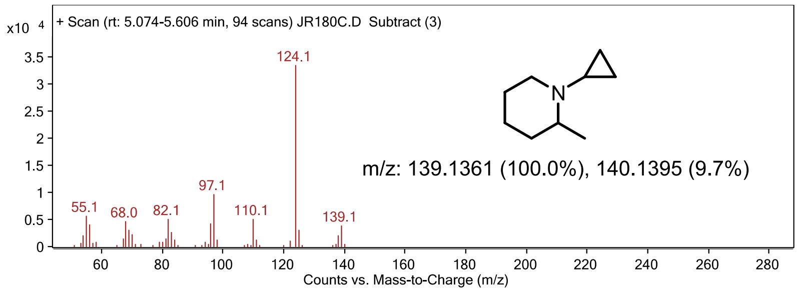

N-(3-chloropropyl)-2-methylpiperidine

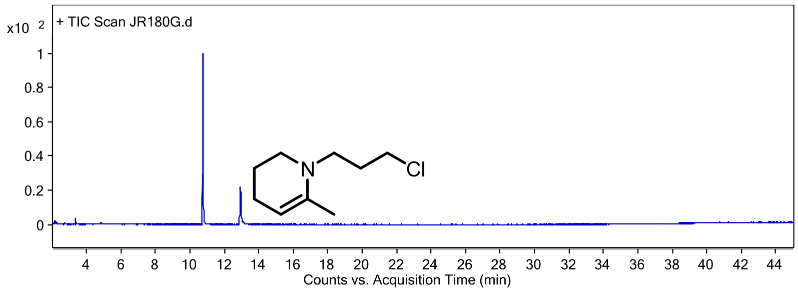

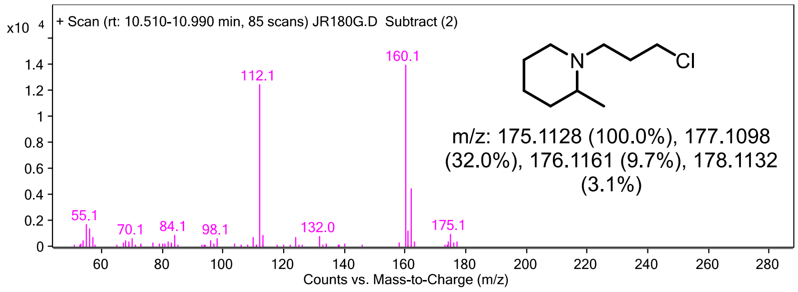

N-benzyl-2-methylpiperidine

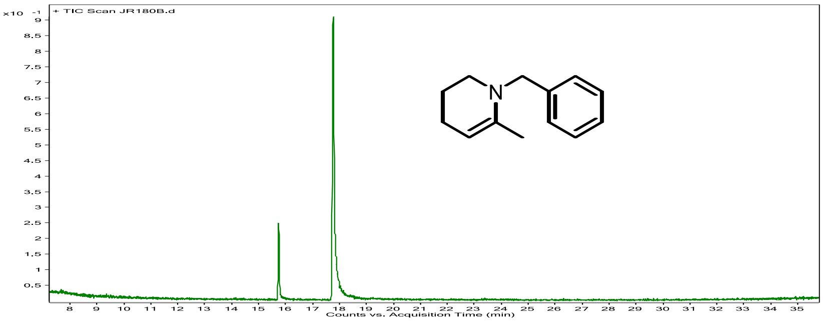

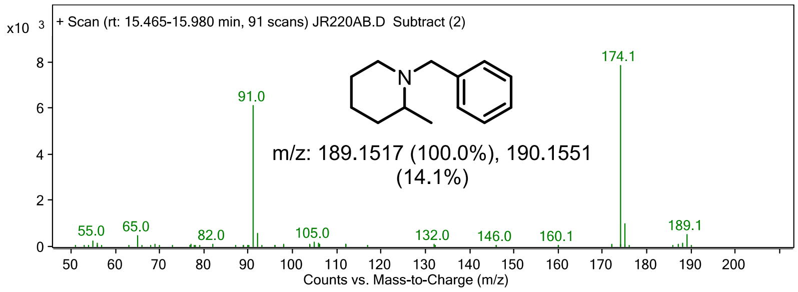

1,2-dimethylazepane

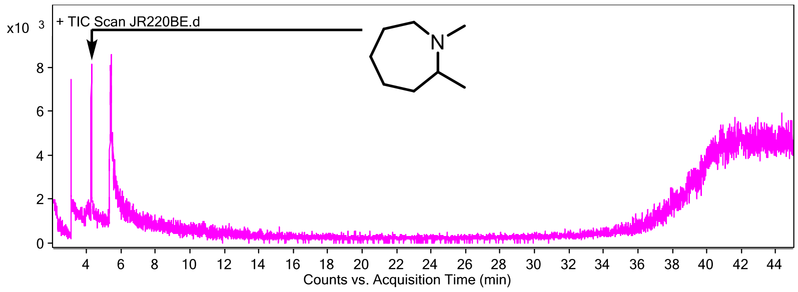

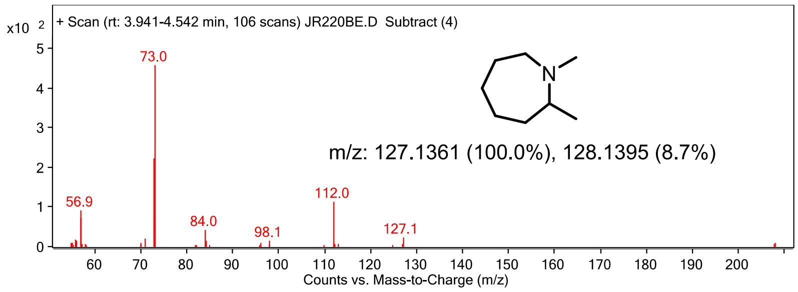

N-propyl-2-methylazepane

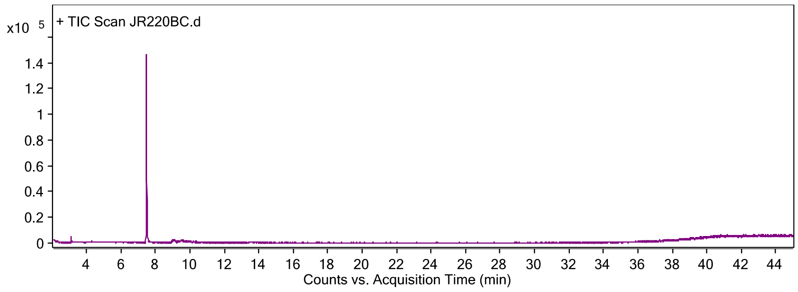

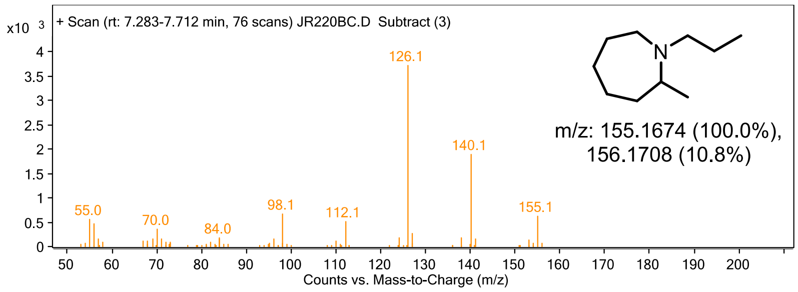

N-allyl-2-methylazepane

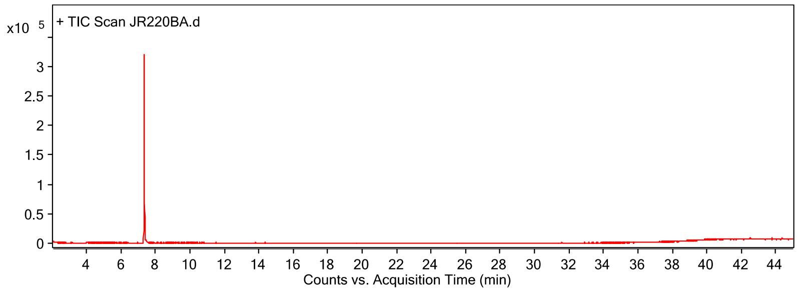

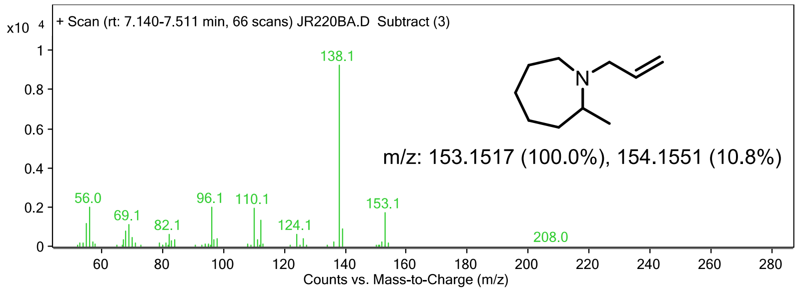

N-propargyl-2-methylazepane

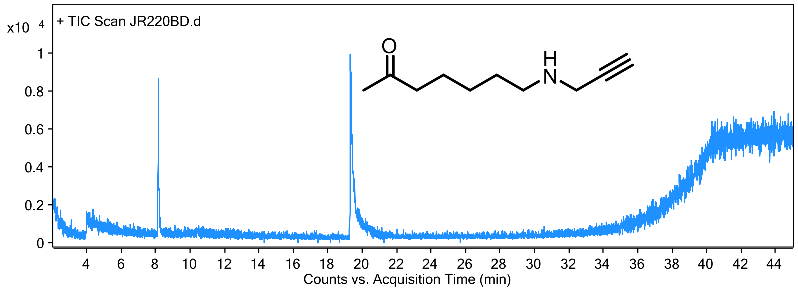

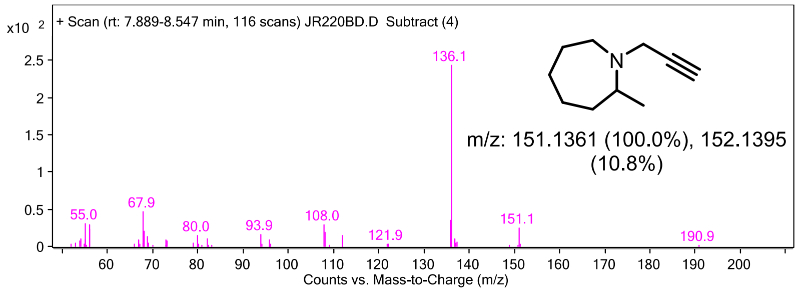

N-cyclopropyl-2-methylazepane

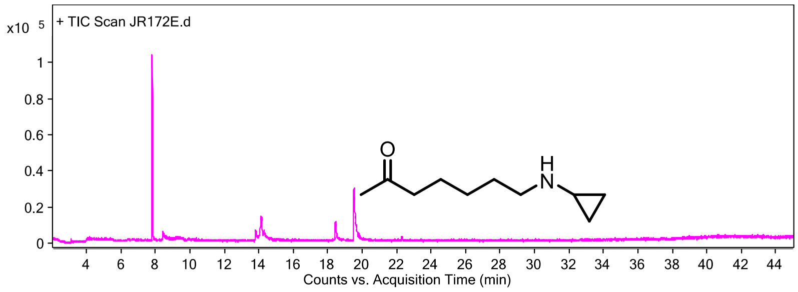

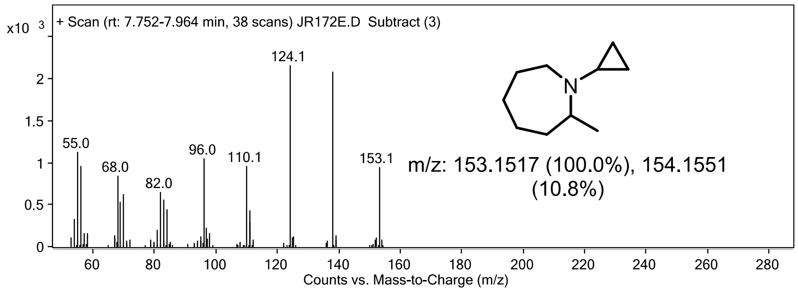

N-(3-chloropropyl)-2-methylazepane

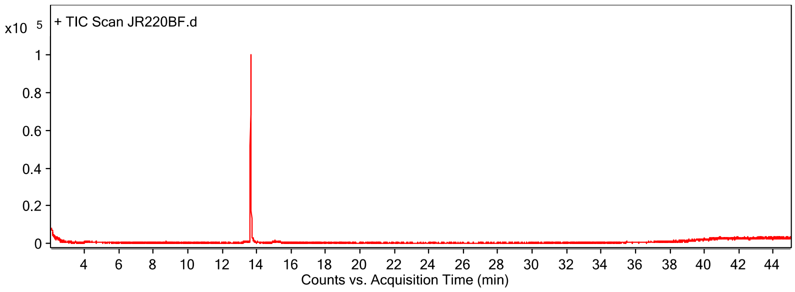

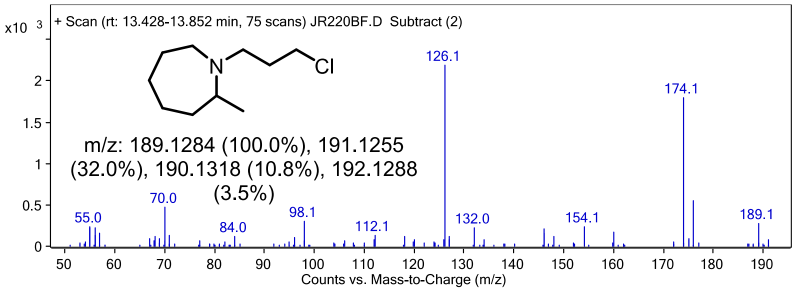

N-benzyl-2-methylazepane

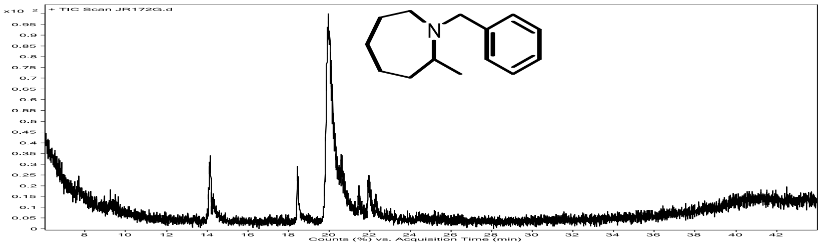

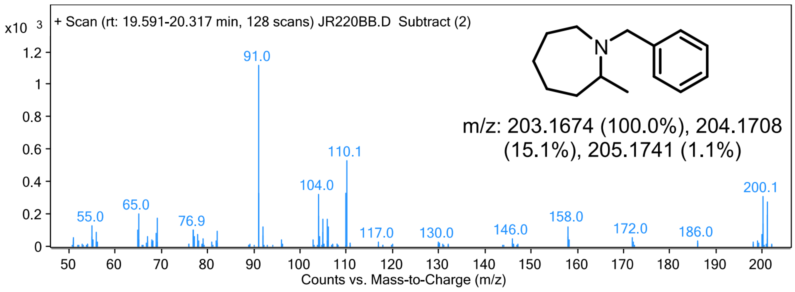

N-methyl-2-phenylpiperidine

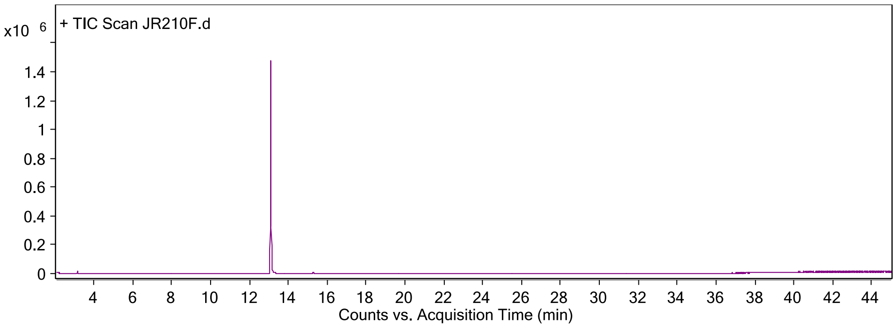

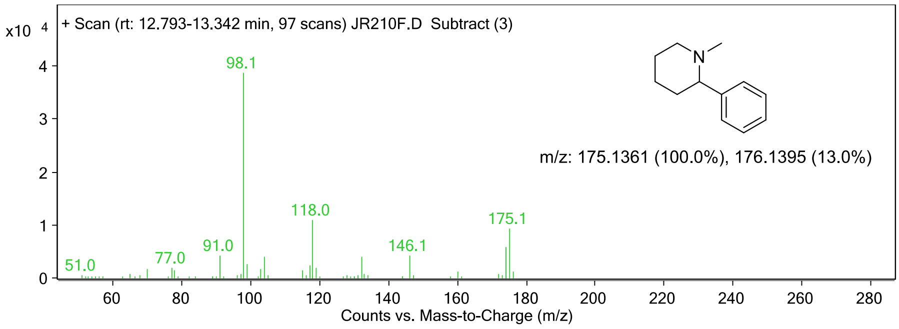

N-propyl-2-phenylpiperidine

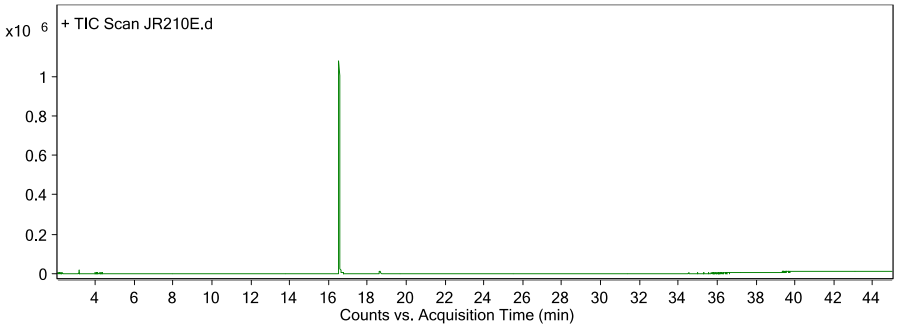

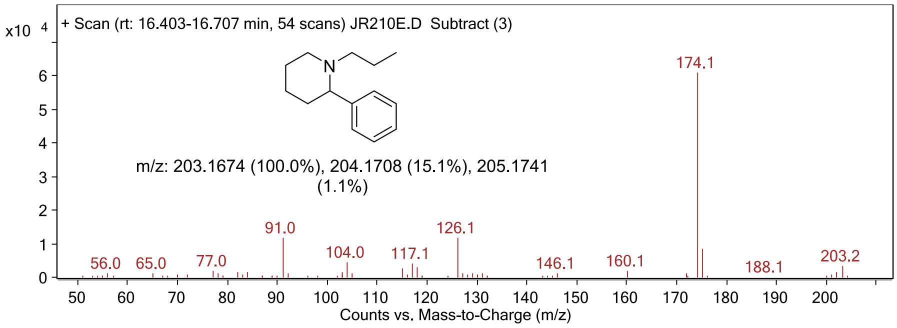

N-allyl-2-phenylpiperidine

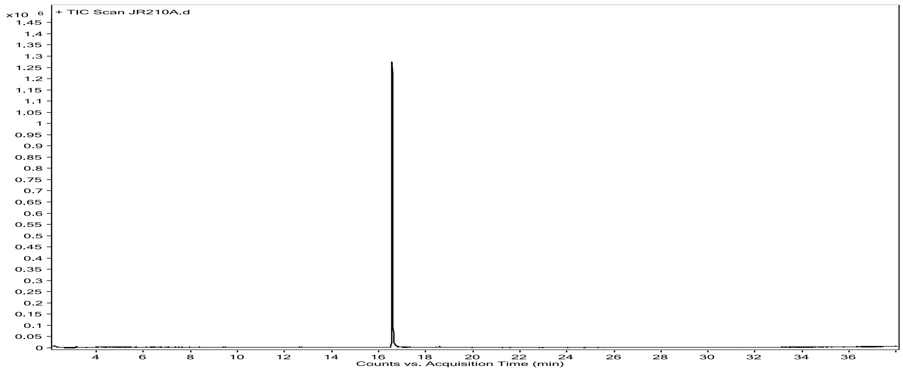

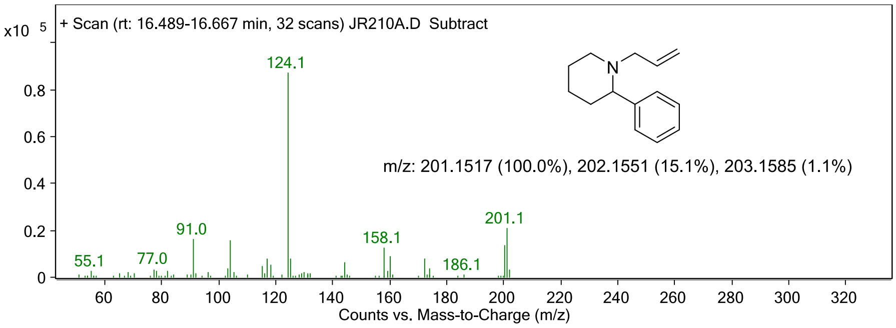

N-propargyl-2-phenylpiperidine

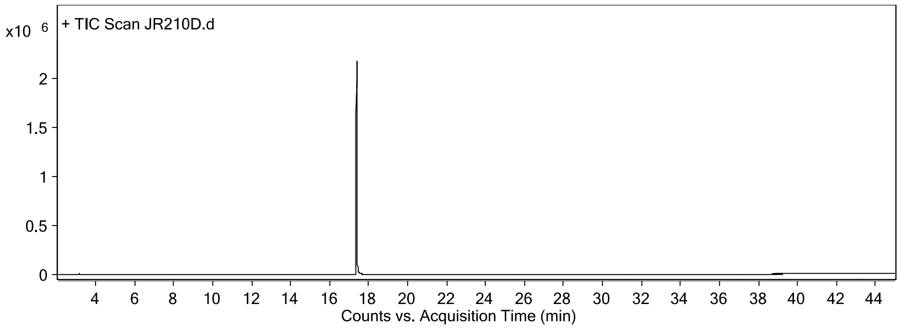

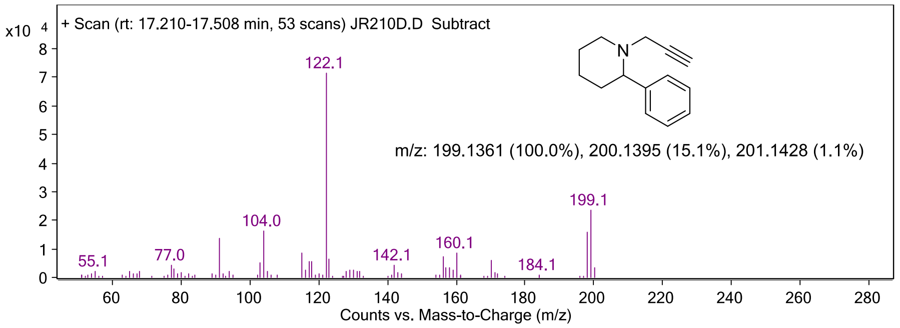

N-cyclopropyl-2-phenylpiperidine

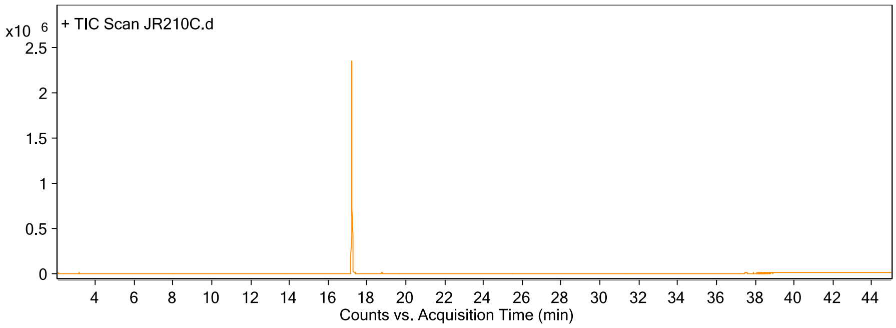

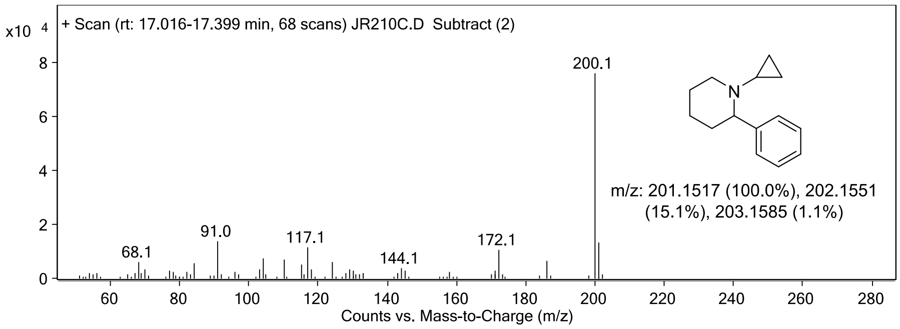

N-(3-chloropropyl)-2-phenylpiperidine

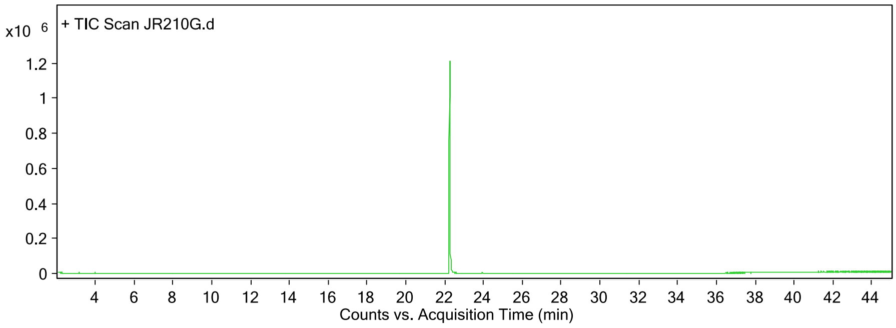

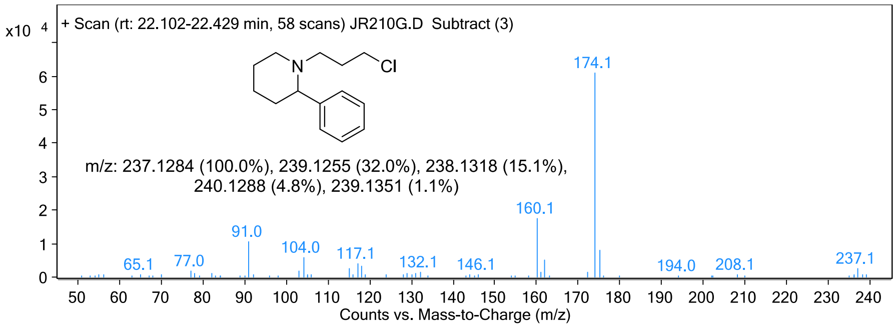

N-benzyl-2-phenylpiperidine

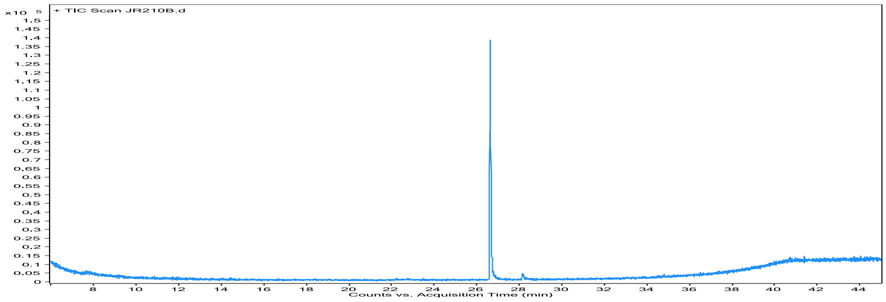

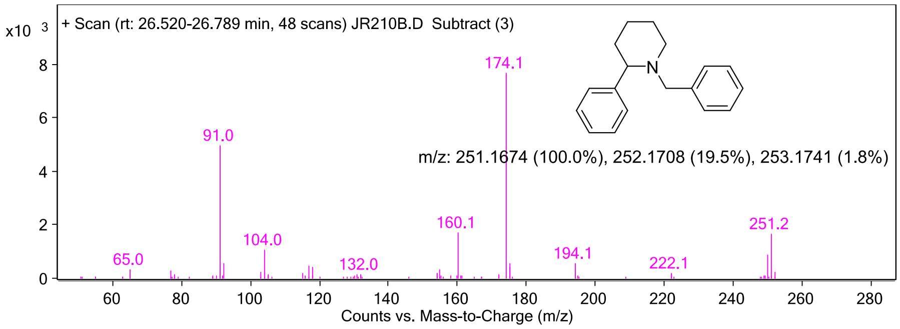

IRED-Catalysed Diketone Cyclisation: *N*-alkylated disubstituted pyrrolidines synthesis

- *GC-MS traces examples of biotransformations using diketones* ***21*** *and* ***22*** *as substrates showing the best conversion to the desired pyrrolidines.*
- *Structures were confirmed by comparison with synthetic standards.*
- *GC-MS method: Agilent HP-1ms column (30 m × 0.32 mm × 0.25 µm), 1 mL·min^{-1}, 50 °C (hold 3 min), then 50 to 290 °C at 10 °C·min^{-1}.*

cis-*N*-cyclopropyl-2-methyl-5-pentylpyrrolidine (cis-21i)

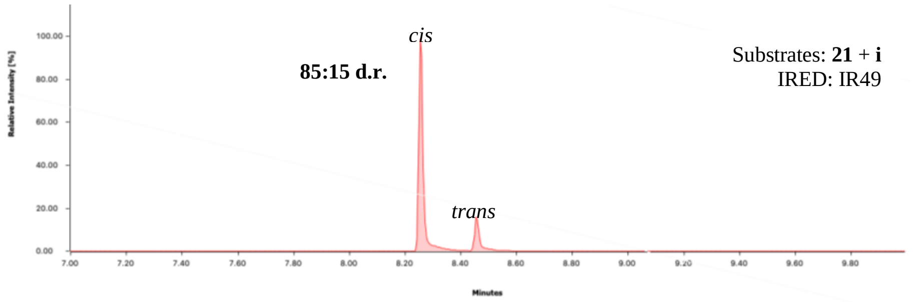

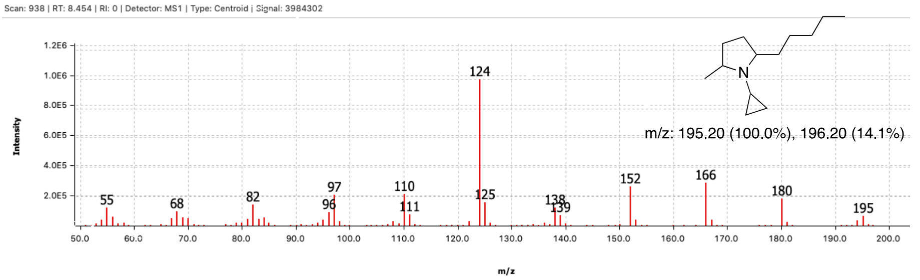

trans-*N*-cyclopropyl-2-methyl-5-pentylpyrrolidine (trans-21i)

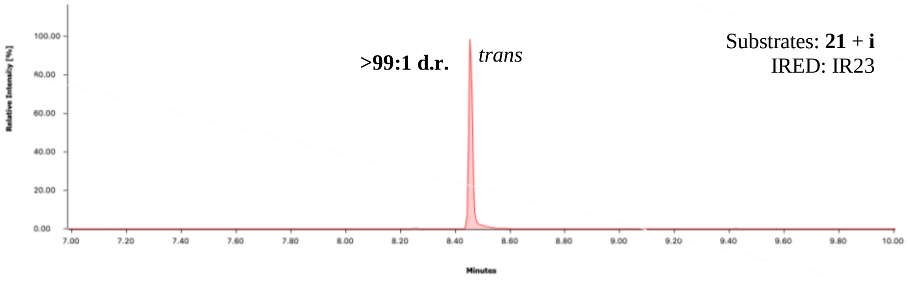

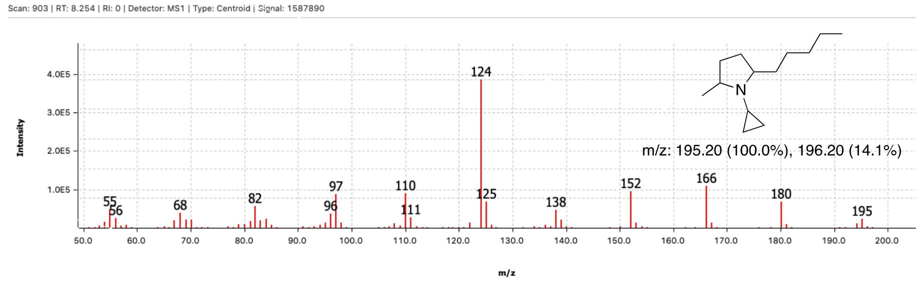

cis-*N*-methyl-2-methyl-5-pentylpyrrolidine (cis-21iii)

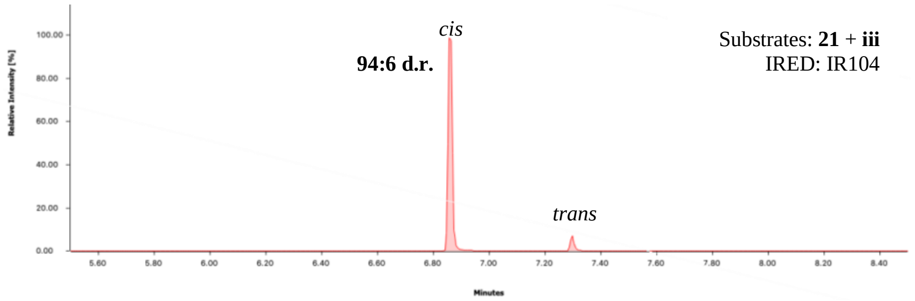

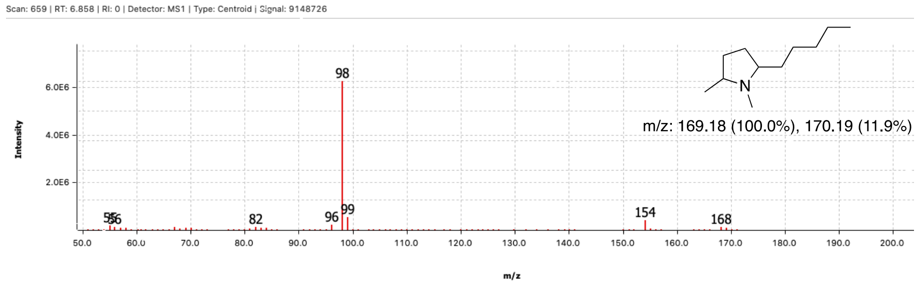

trans-*N*-methyl-2-methyl-5-pentylpyrrolidine (trans-21iii)

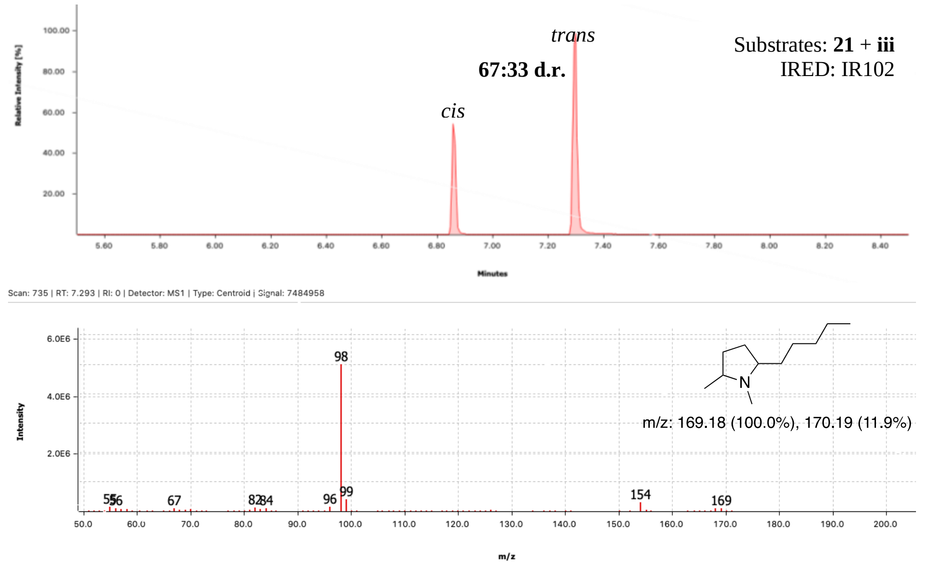

cis-*N*-allyl-2-methyl-5-pentylpyrrolidine (cis-21v)

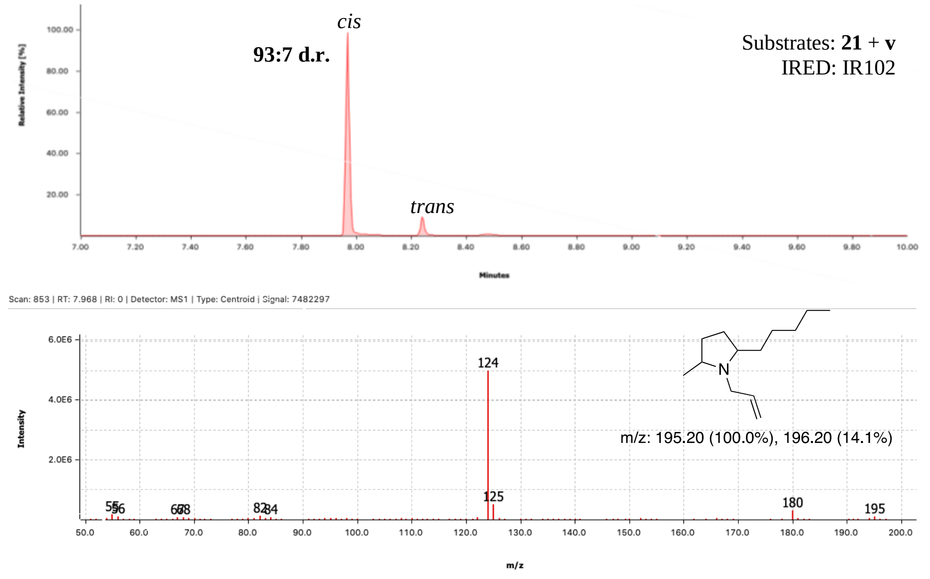

trans-*N*-allyl-2-methyl-5-pentylpyrrolidine (trans-21v)

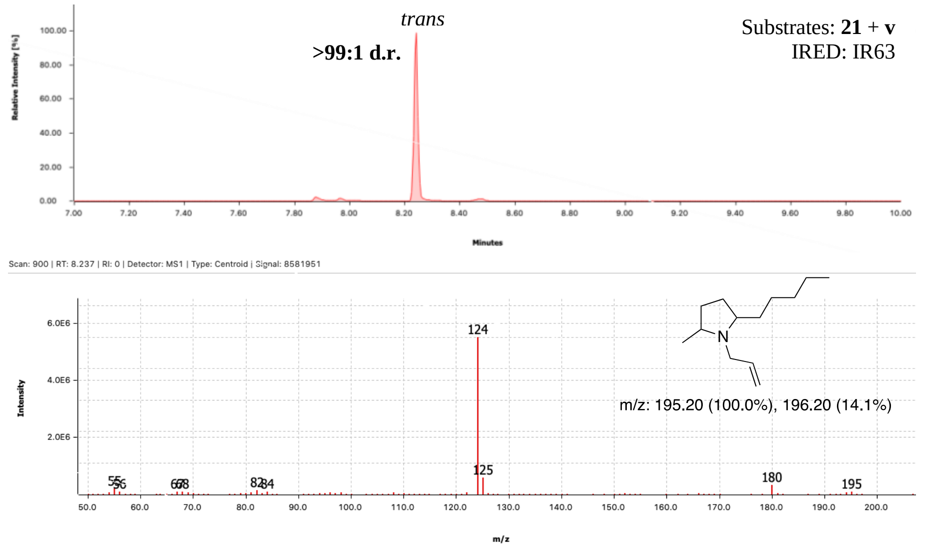

cis-*N*-propargyl-2-methyl-5-pentylpyrrolidine (cis-21vi)

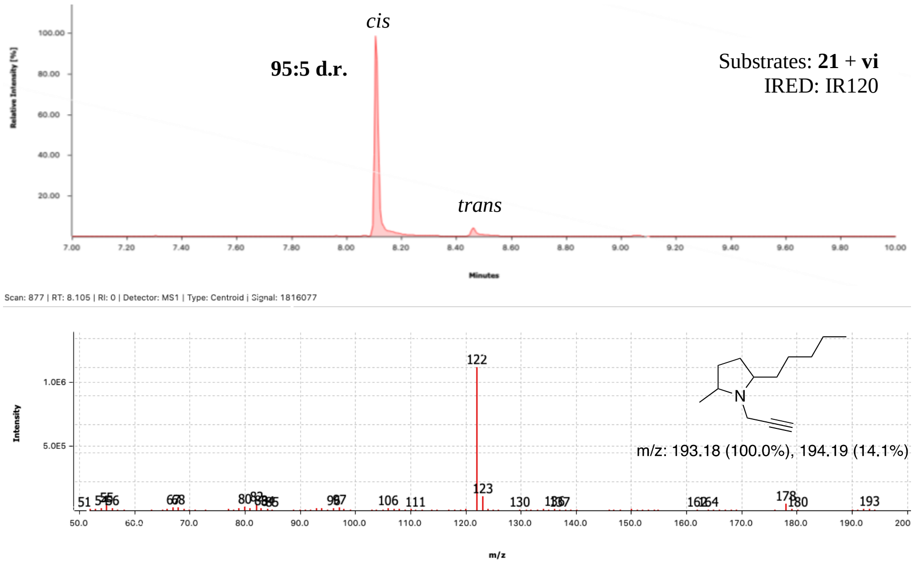

trans-*N*-propargyl-2-methyl-5-pentylpyrrolidine (trans-21vi)

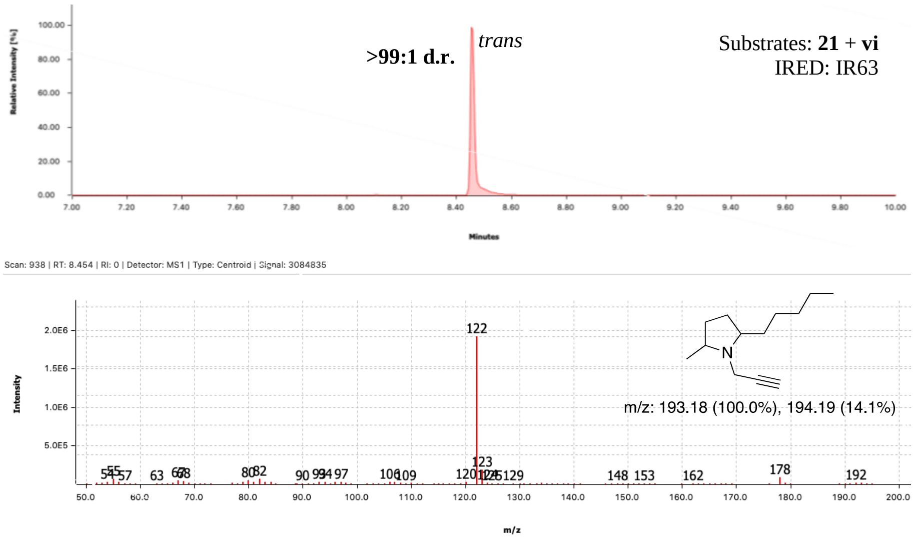

cis-*N*-cyclopropyl-2-methyl-5-phenylpyrrolidine (cis-22i)

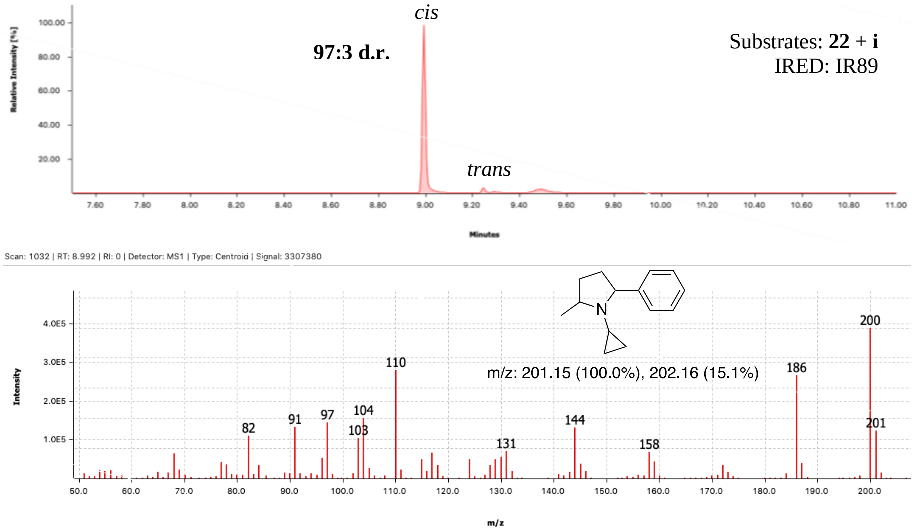

trans-*N*-cyclopropyl-2-methyl-5-phenylpyrrolidine (trans-22i)

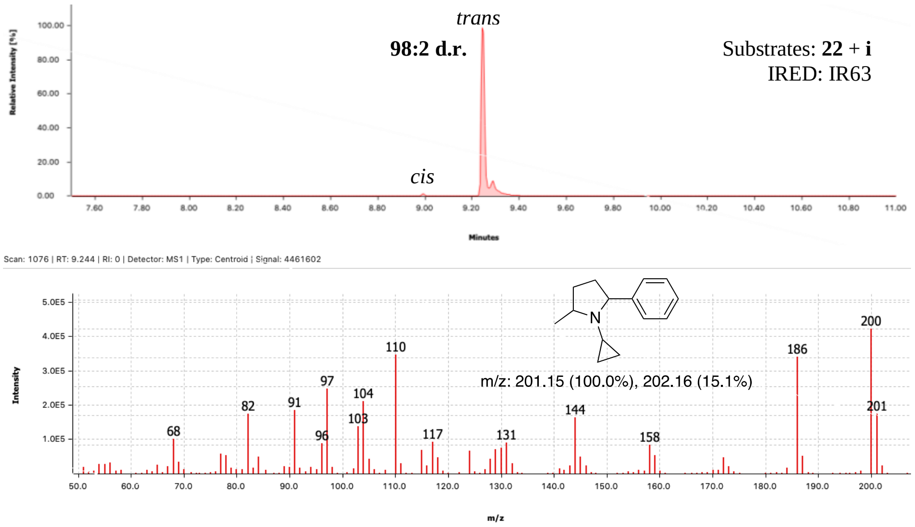

cis-*N*-methyl-2-methyl-5-phenylpyrrolidine (cis-22iii)

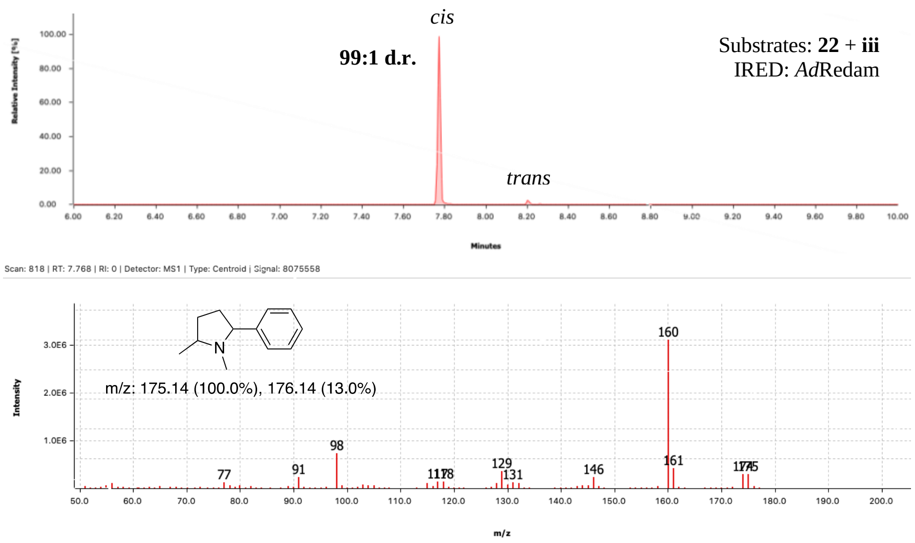

trans-*N*-methyl-2-methyl-5-phenylpyrrolidine (trans-22iii)

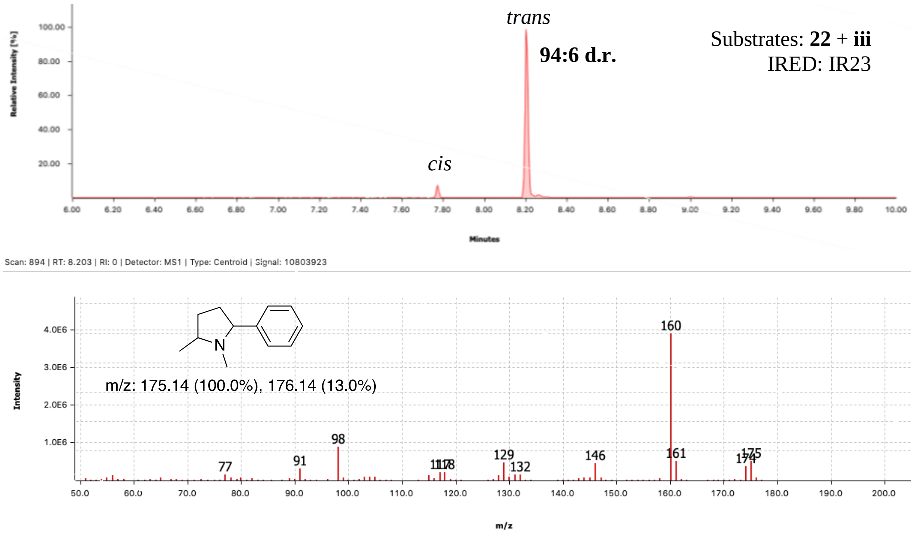

cis-*N*-allyl-2-methyl-5-phenylpyrrolidine (cis-22v)

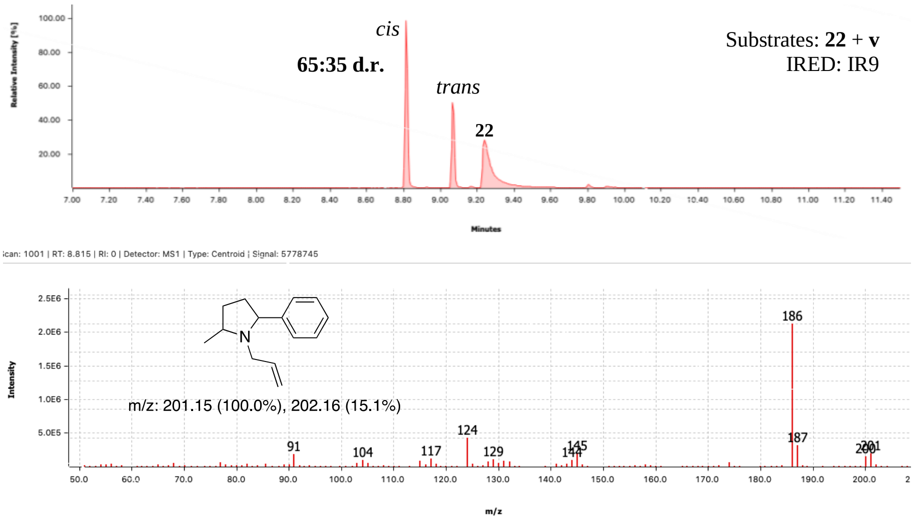

trans-*N*-allyl-2-methyl-5-phenylpyrrolidine (trans-22v)

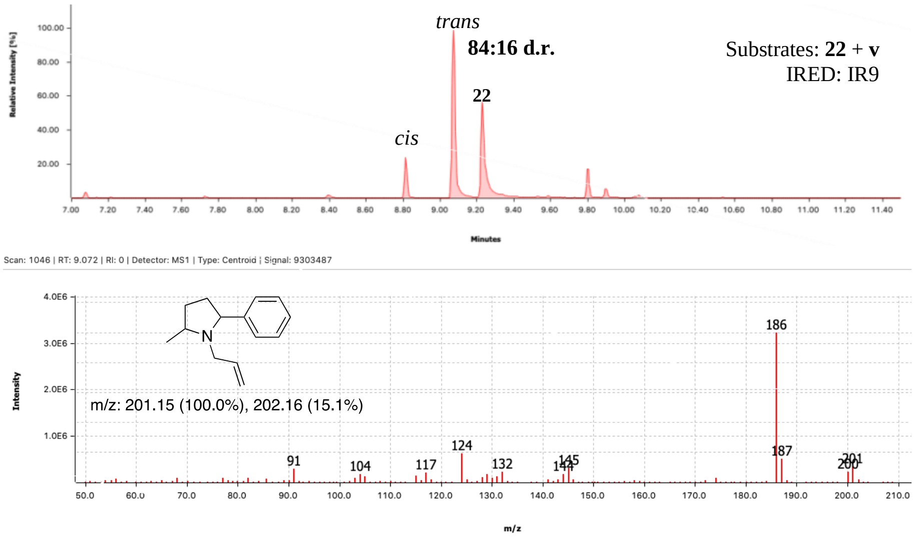

cis-*N*-propargyl-2-methyl-5-phenylpyrrolidine (cis-22vi)

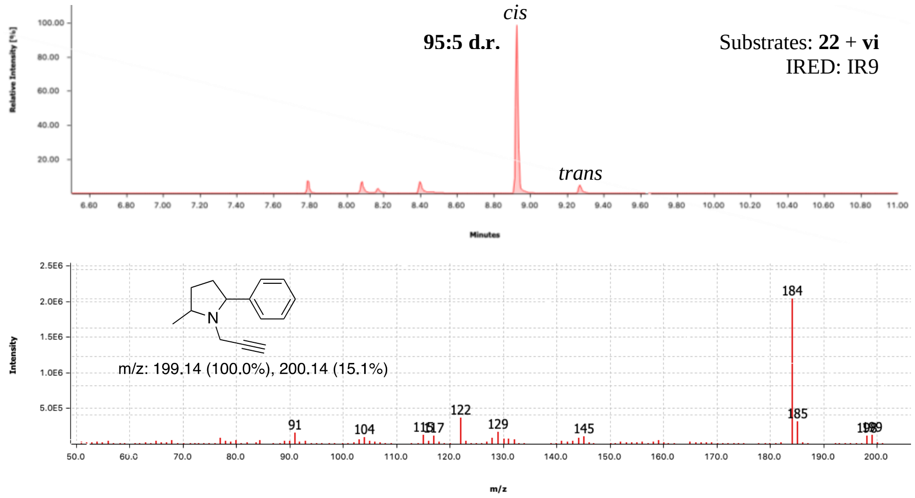

trans-*N*-propargyl-2-methyl-5-phenylpyrrolidine (trans-22vi)

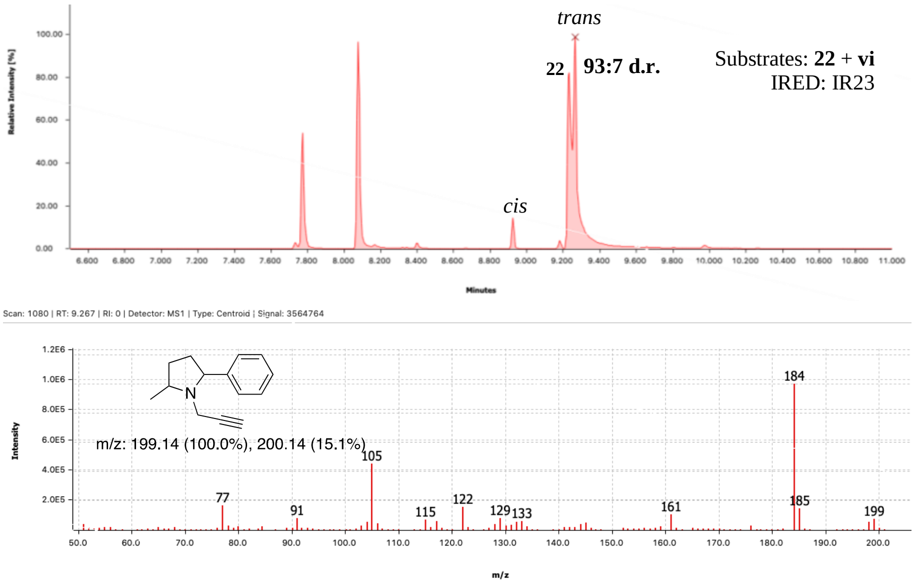

IRED-Catalysed Diketone Cyclisation: enamine and pyrrole by-products

- *Examples of GC-MS traces for biotransformations using diketones **21** and **22** as substrates where enamine or pyrrole by-products were observed.*
- *All structures were proposed based on EI-MS fragmentation.*
- *Enamines were not observed in any reactions using propargylamine (**vi**).*
- *Pyrrole were not observed when using diketone **22** and propargylamine (**vi**).*
- *For full screening and product/by-products distribution results see section "IRED Screening for Diketone Cyclisation"*
- *GC-MS method: Agilent HP-1ms column (30 m × 0.32 mm × 0.25 µm), 1 mL·min^{-1}, 50 °C (hold 3 min), then 50 to 290 °C at 10 °C·min^{-1}.*

N-cyclopropyl-2-methyl-5-pentyl-2,3-dihydro-1H-pyrrole

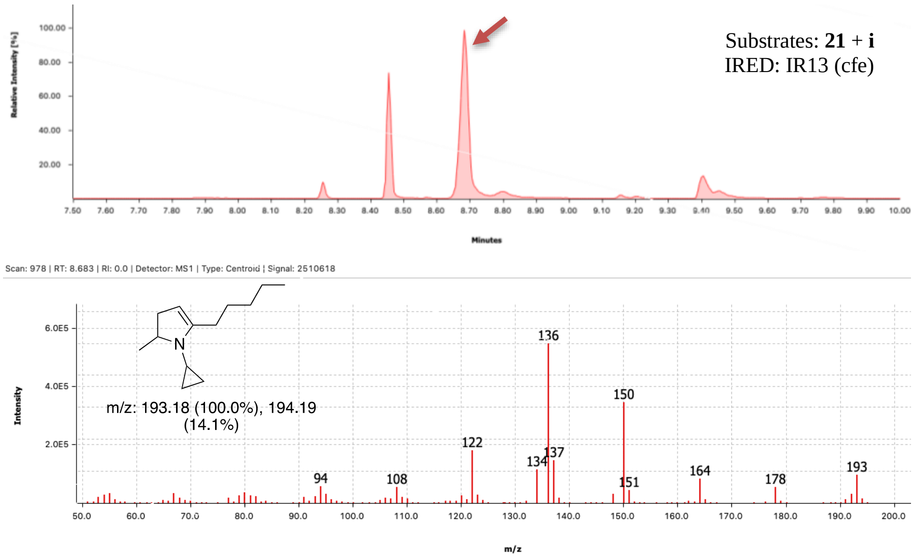

N-cyclopropyl-2-methyl-5-pentyl-1H-pyrrole

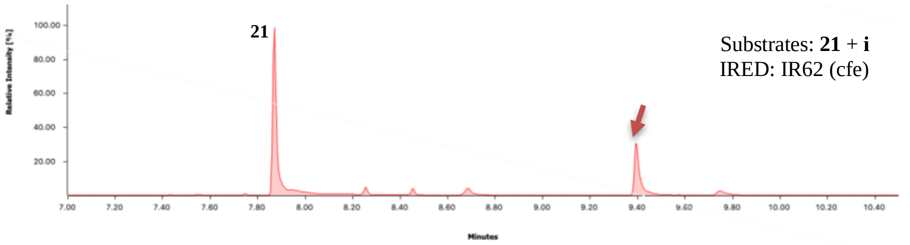

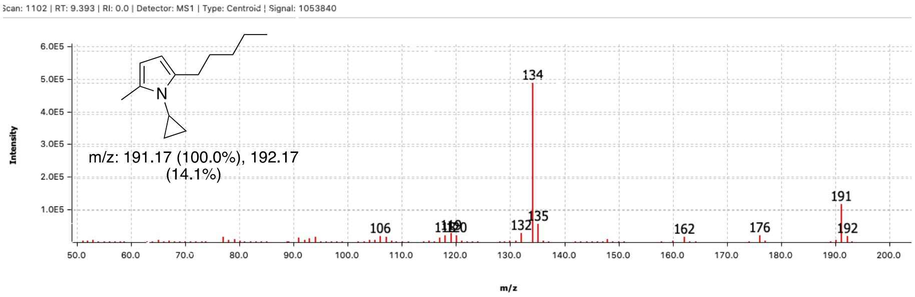

N-methyl-2-methyl-5-pentyl-2,3-dihydro-1H-pyrrole

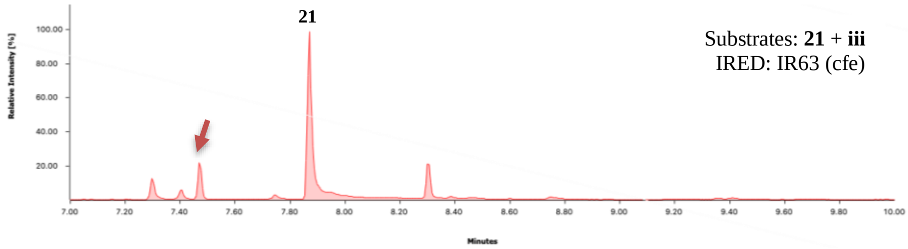

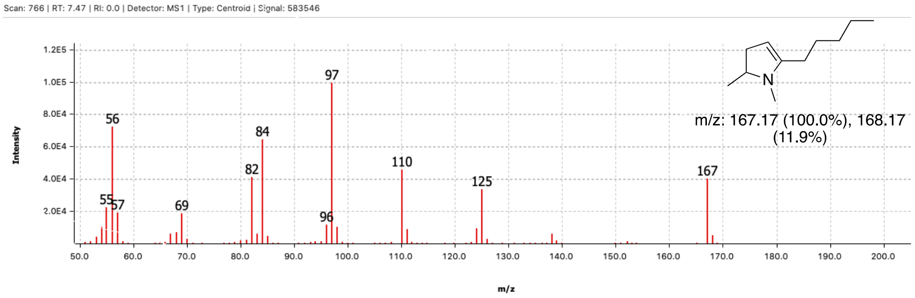

N-methyl-2-methyl-5-pentyl-1H-pyrrole

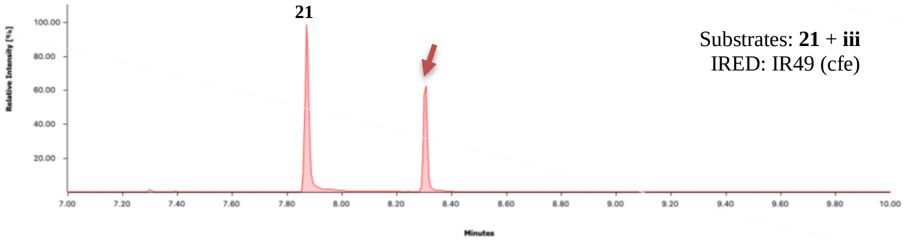

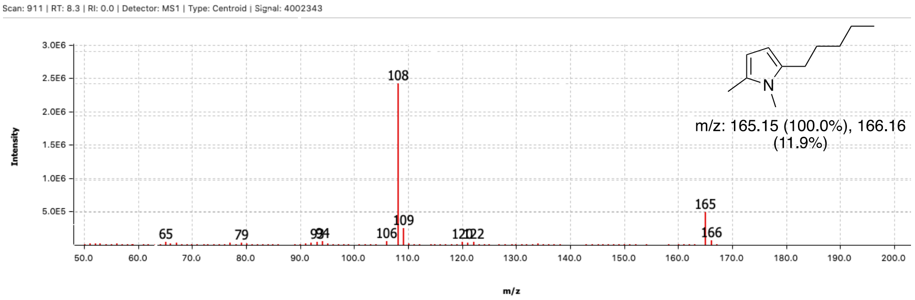

N-allyl-2-methyl-5-pentyl-2,3-dihydro-1H-pyrrole

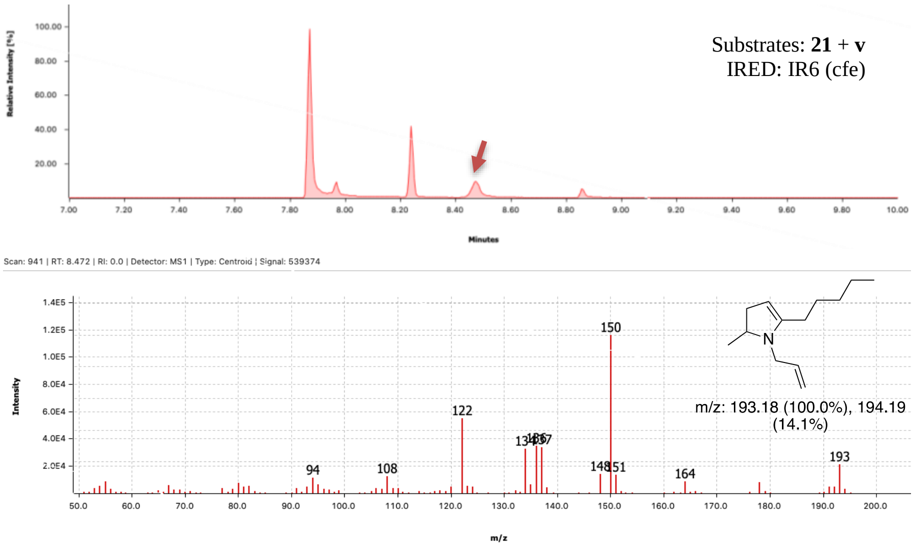

N-allyl-2-methyl-5-pentyl-1H-pyrrole

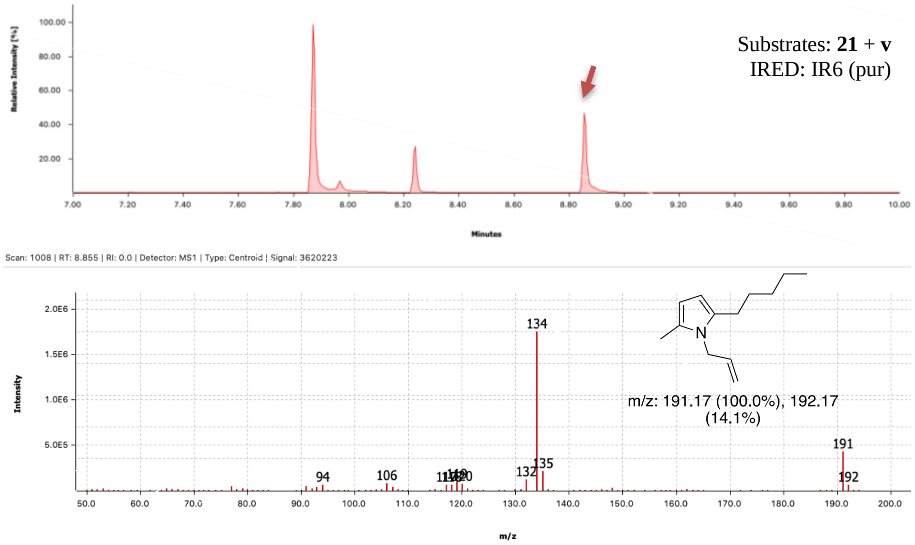

N-methyl-5-pentyl-1-(prop-2-yn-1-yl)-1H-pyrrole

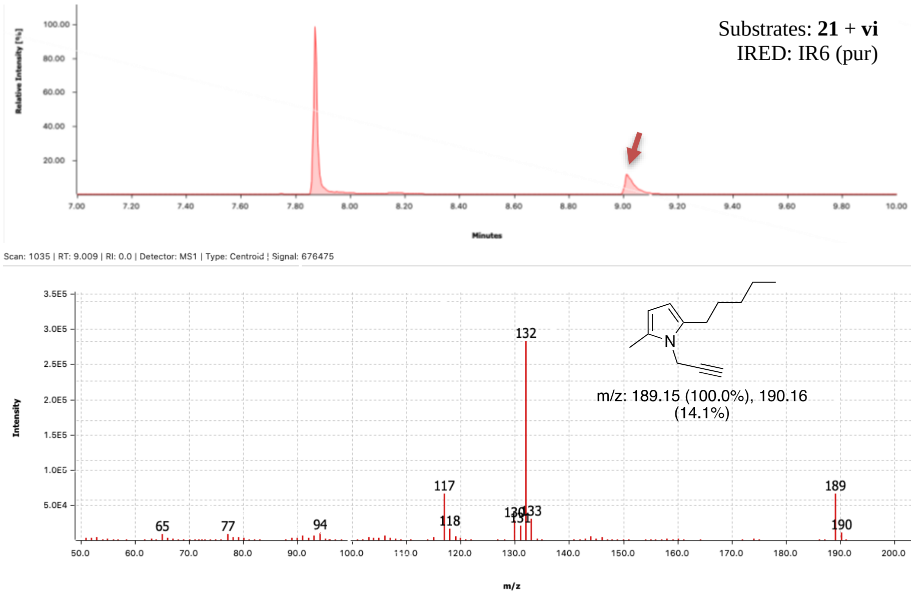

N-cyclopropyl-2-methyl-5-phenyl-2,3-dihydro-1H-pyrrole

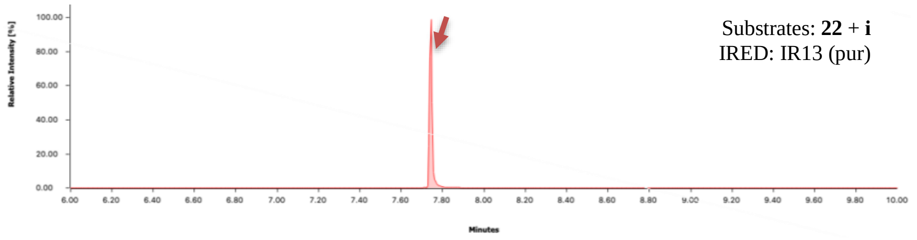

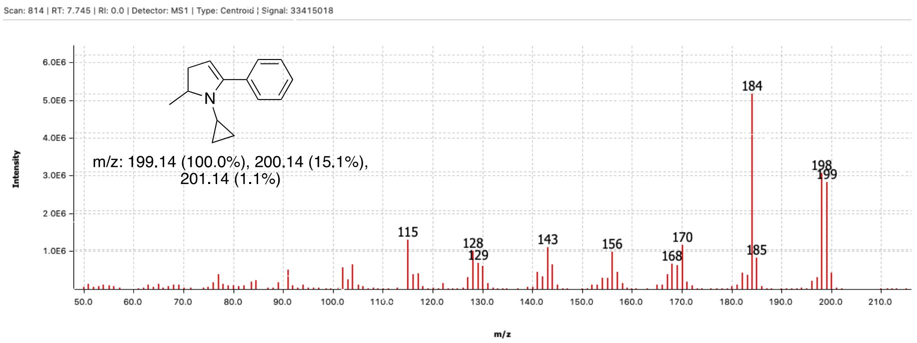

N-cyclopropyl-2-methyl-5-phenyl-1H-pyrrole

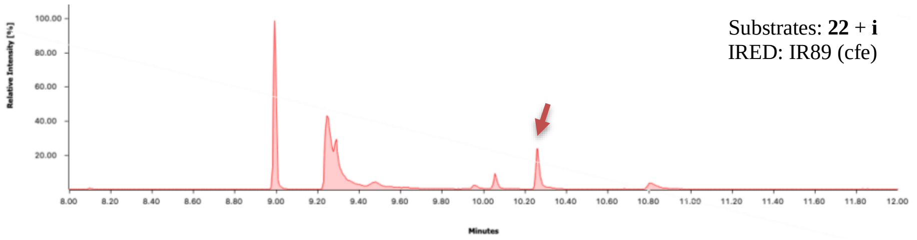

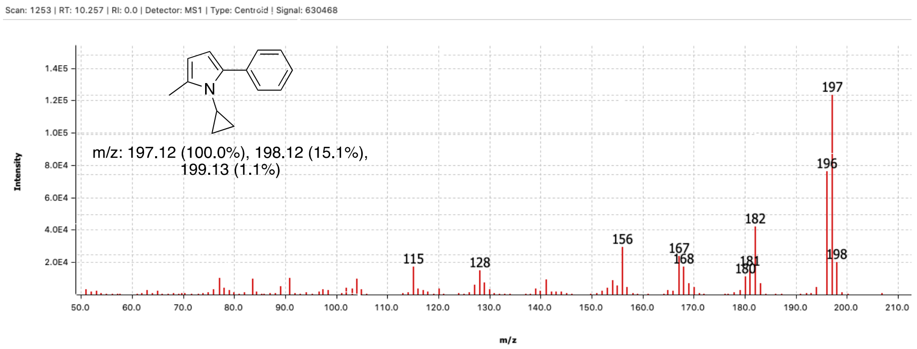

N-methyl-2-methyl-5-phenyl-2,3-dihydro-1H-pyrrole and *N*-methyl-2-methyl-5-phenyl-1H-pyrrole

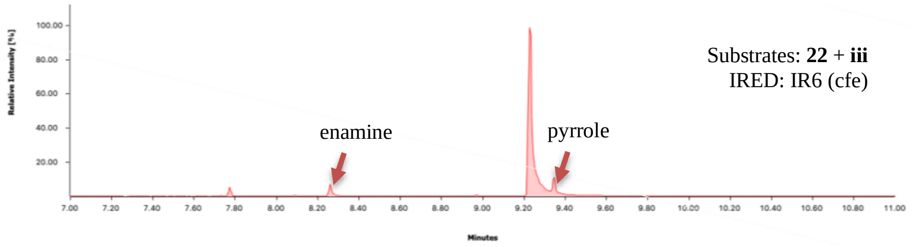

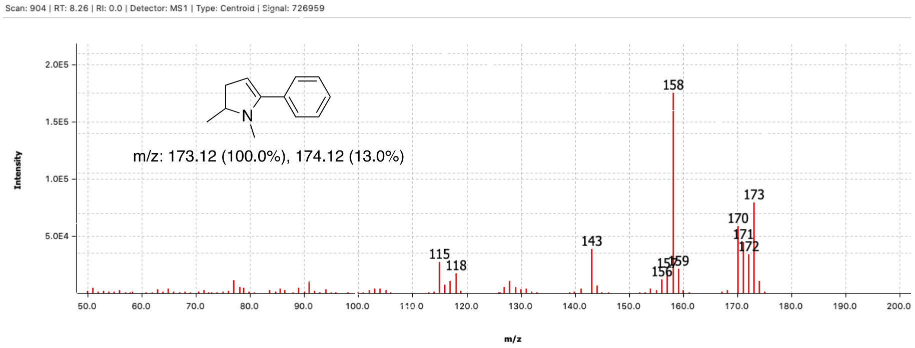

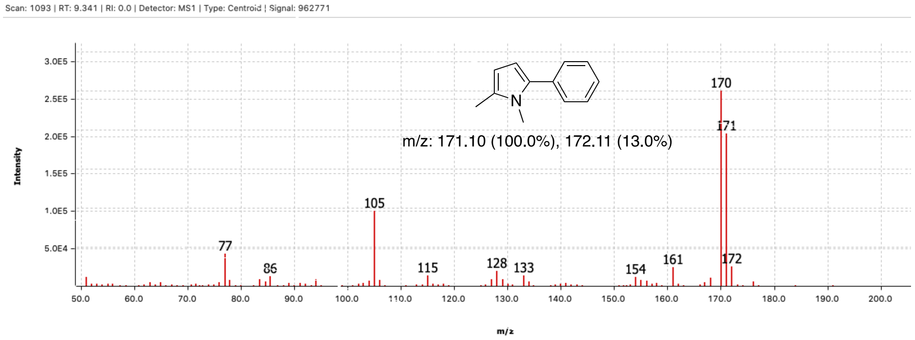

N-allyl-2-methyl-5-phenyl-2,3-dihydro-1H-pyrrole

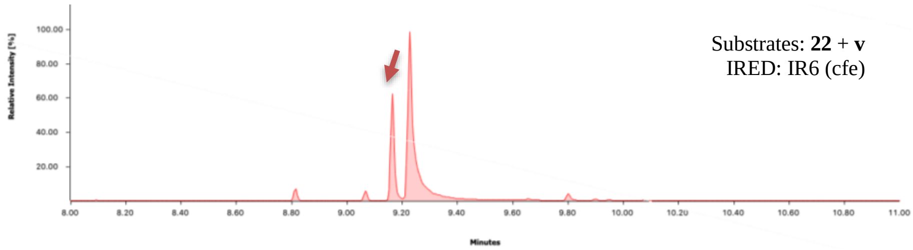

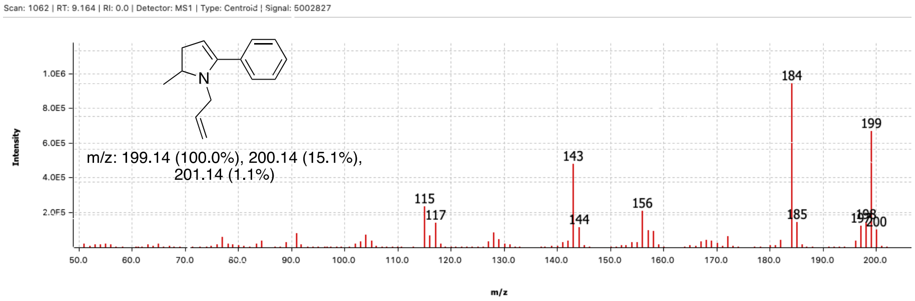

N-allyl-2-methyl-5-phenyl-1H-pyrrole

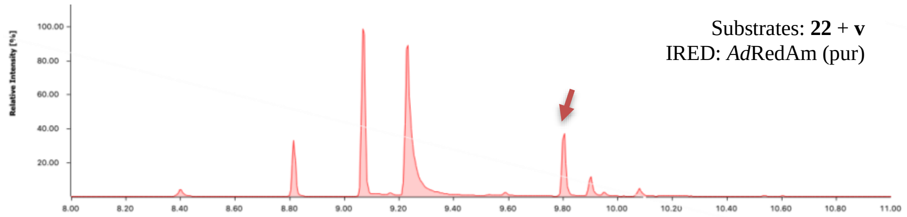

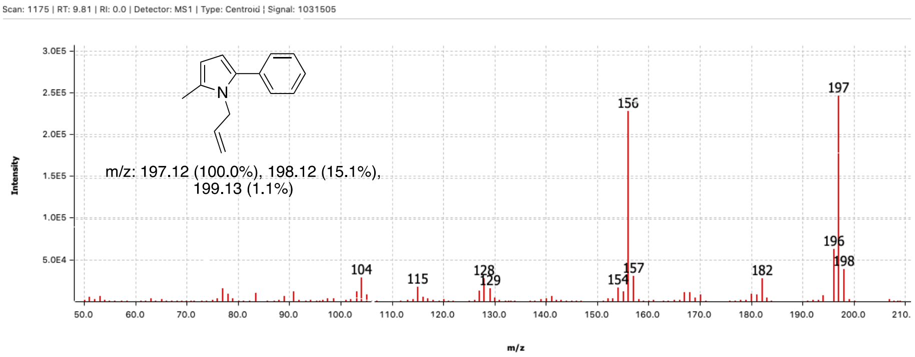

Determination of Enantiomeric Excess

For examples in which the cyclisation reaction afforded a chiral product, *ee* was determined through the use of either GC-FID or HPLC employing a chiral stationary phase. Chiral chemical standards were synthesised through the N-alkylation of enantiomerically pure or enriched starting materials obtained either by previously described enzymatic methods or commercially, as follows:

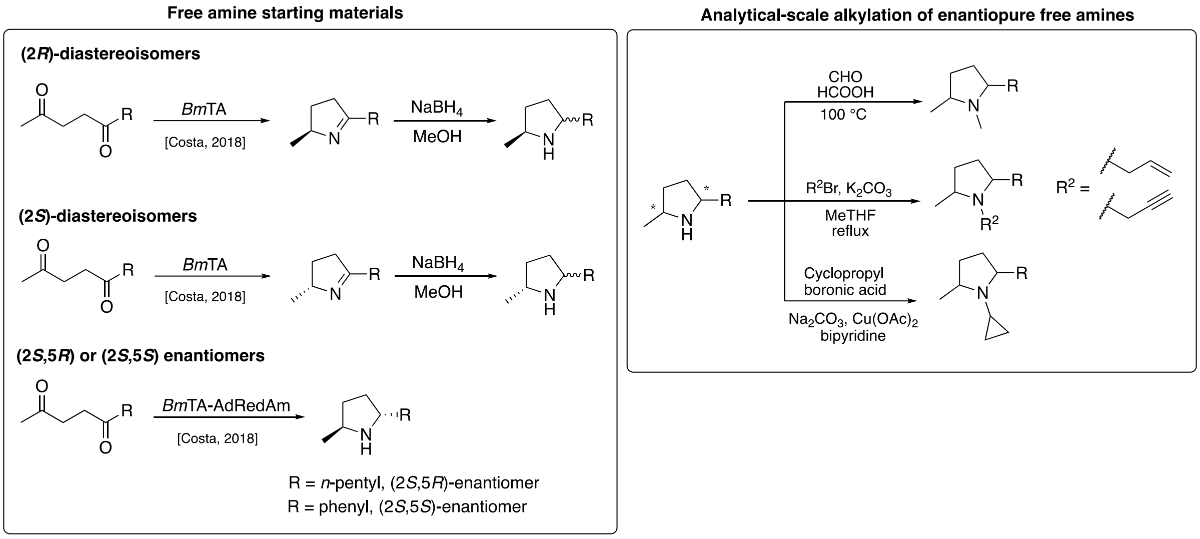

N-alkylation was performed on a 0.1 mmol scale using equivalent methods to those described in the synthesis of racemic standards. However, reactions were simply sampled for crude analysis rather than subjected to a full work-up and purification.

GC-FID Chromatograms

Methods:

***A**: Agilent CP-ChiraSil-DEX CB column (25 m × 0.25 mm × 0.25 µm), 1 mL·min^{-1}, 50 to 20 °C at 5 °C·min^{-1}, then hold for 2 min.*

***B**: Agilent CP-ChiraSil-DEX CB column (25 m × 0.25 mm × 0.25 µm), 1 mL·min^{-1}, 80 to 120 °C at 2 °C·min^{-1}, hold for 10 min, 120 to 180 °C at 20 °C·min^{-1}, hold for 3 min.*

N-propyl-2-methylpiperidine (13iii) [A]

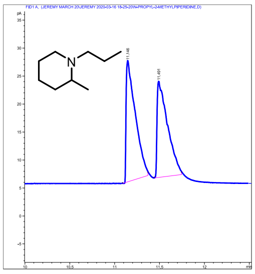

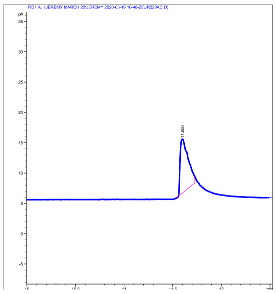

N-allyl-2-methylpiperidine (13v)[A]

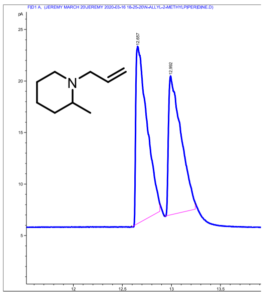

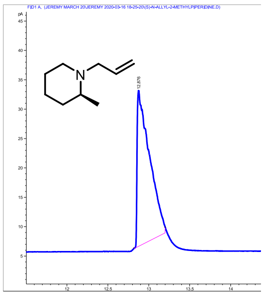

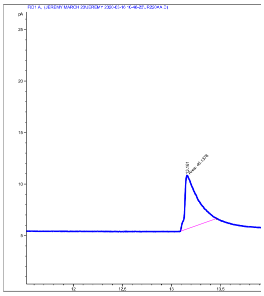

N-propargyl-2-methylpiperidine (13vi)[A]

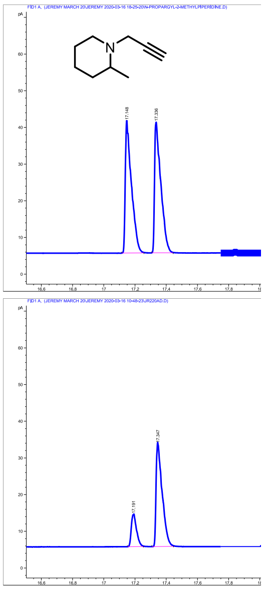

N-Cyclopropyl-2-methylpiperidine (13i)[A]

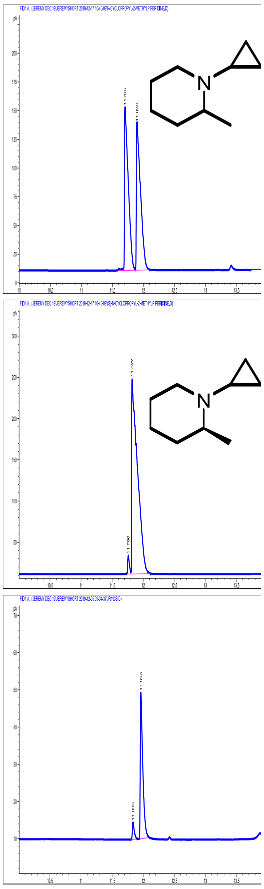

N-propargyl-2-methylazepane (14vi)[A]

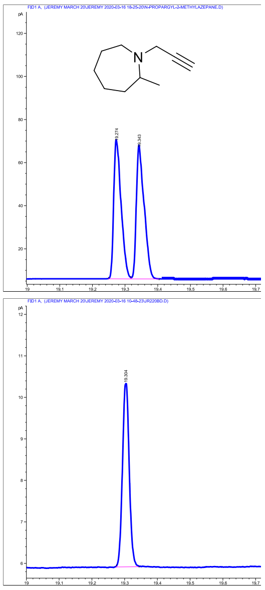

N-cyclopropyl-2-methyl-5-pentylpyrrolidine (21i)[B]

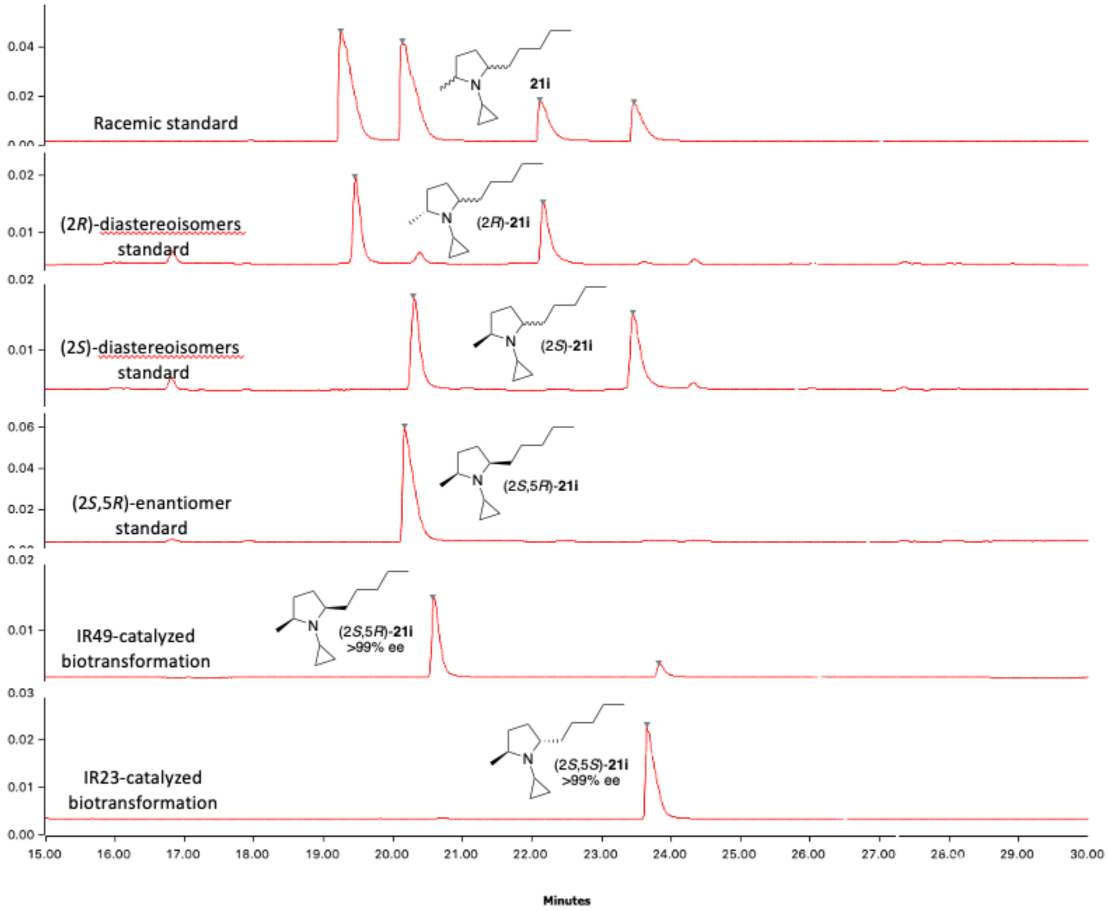

N-methyl-2-methyl-5-pentylpyrrolidine (21iii) [B]

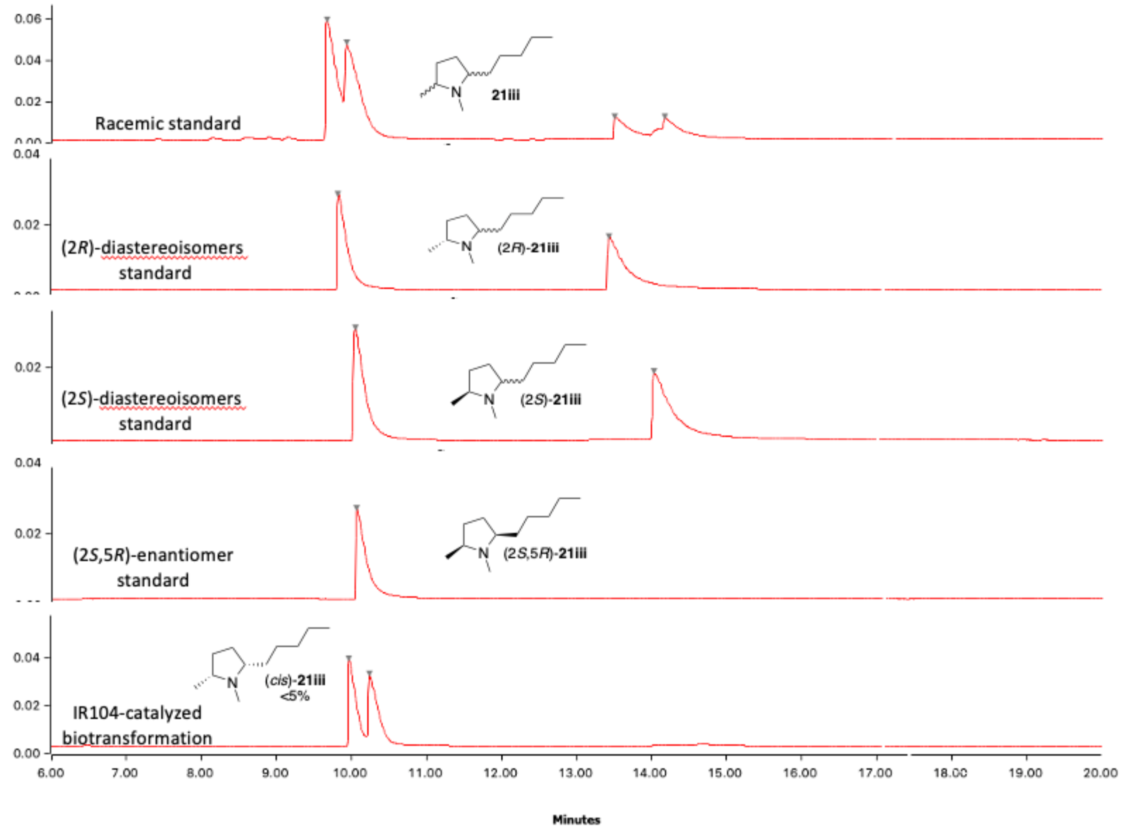

***N*-allyl-2-methyl-5-pentylpyrrolidine (21v)** [B]

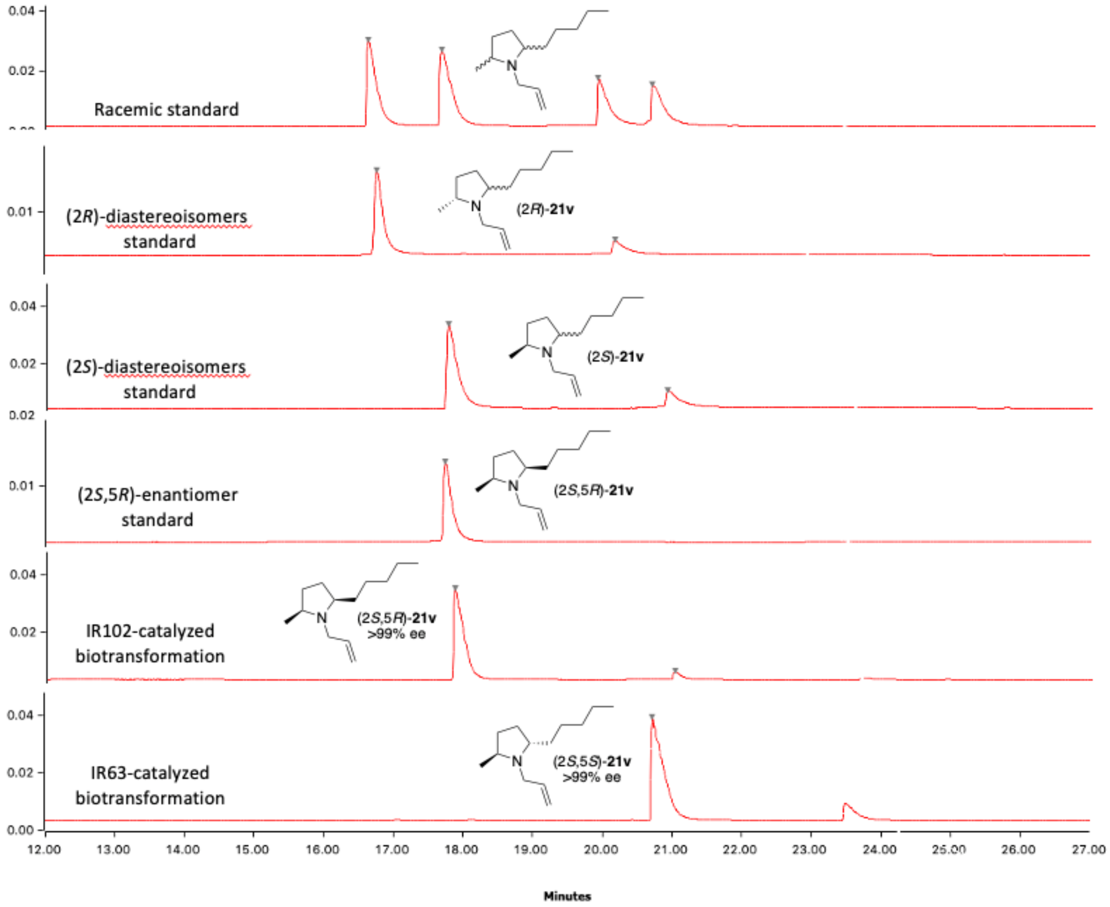

***N*-propargyl-2-methyl-5-pentylpyrrolidine (21vi)** [B]

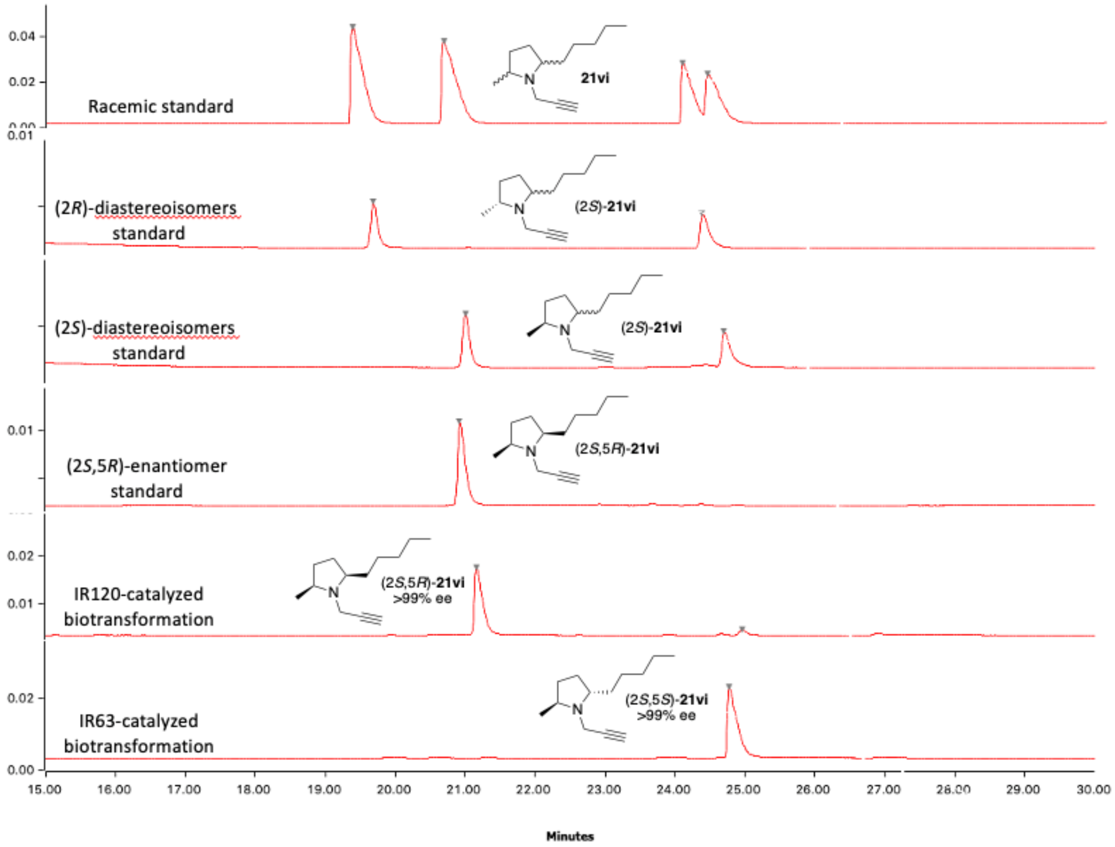

***N*-cyclopropyl-2-methyl-5-phenylpyrrolidine (22i)** [B]

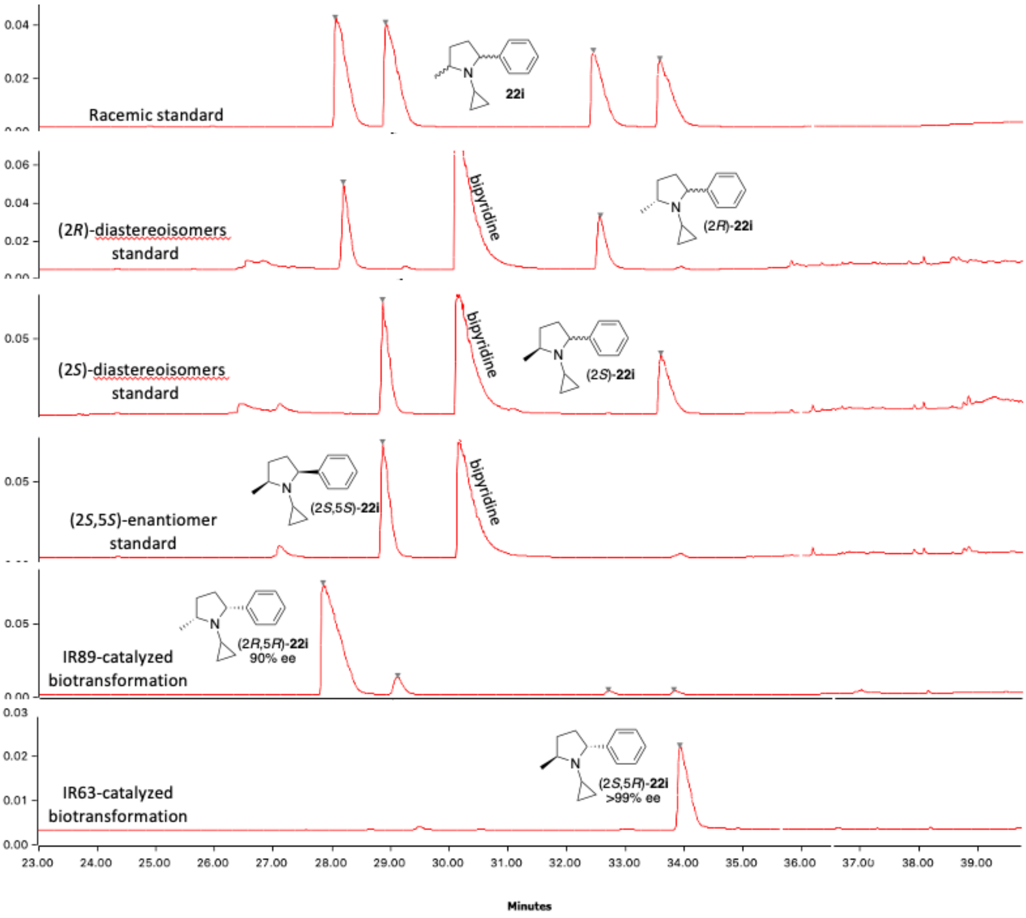

N-methyl-2-methyl-5-phenylpyrrolidine (22iii) [B]

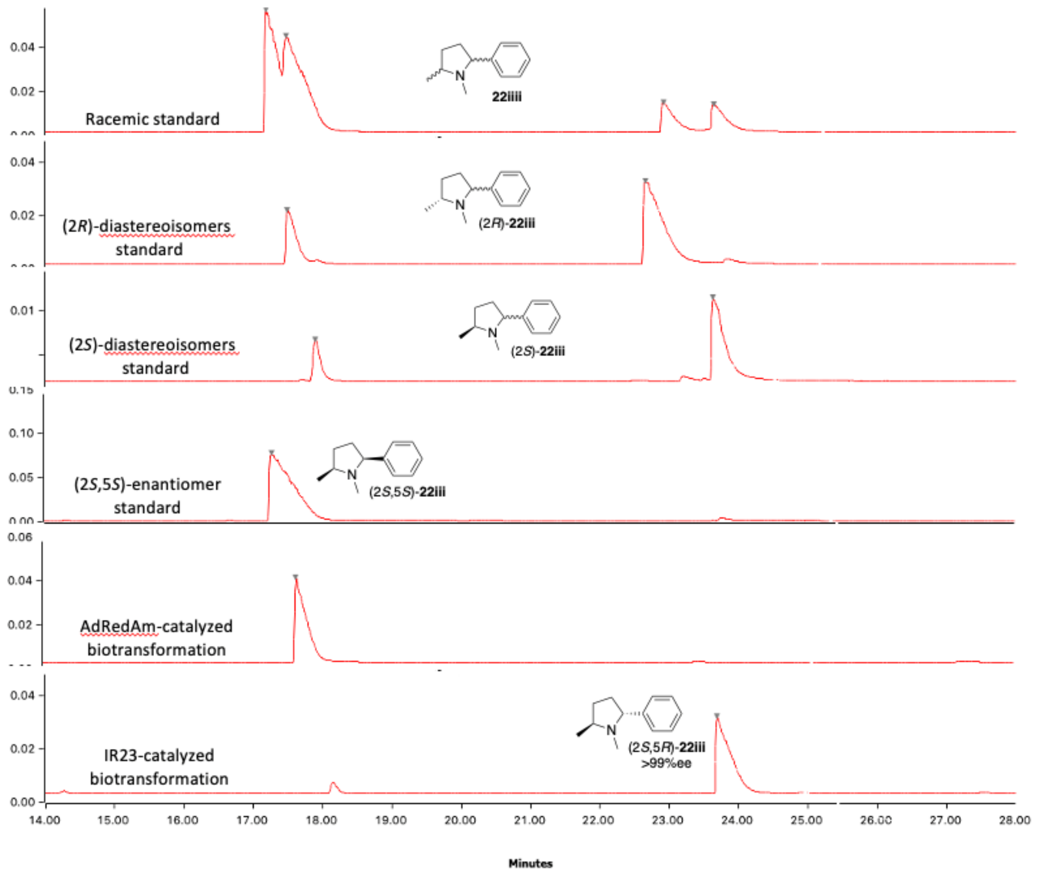

N-allyl-2-methyl-5-phenylpyrrolidine (22v) [B]

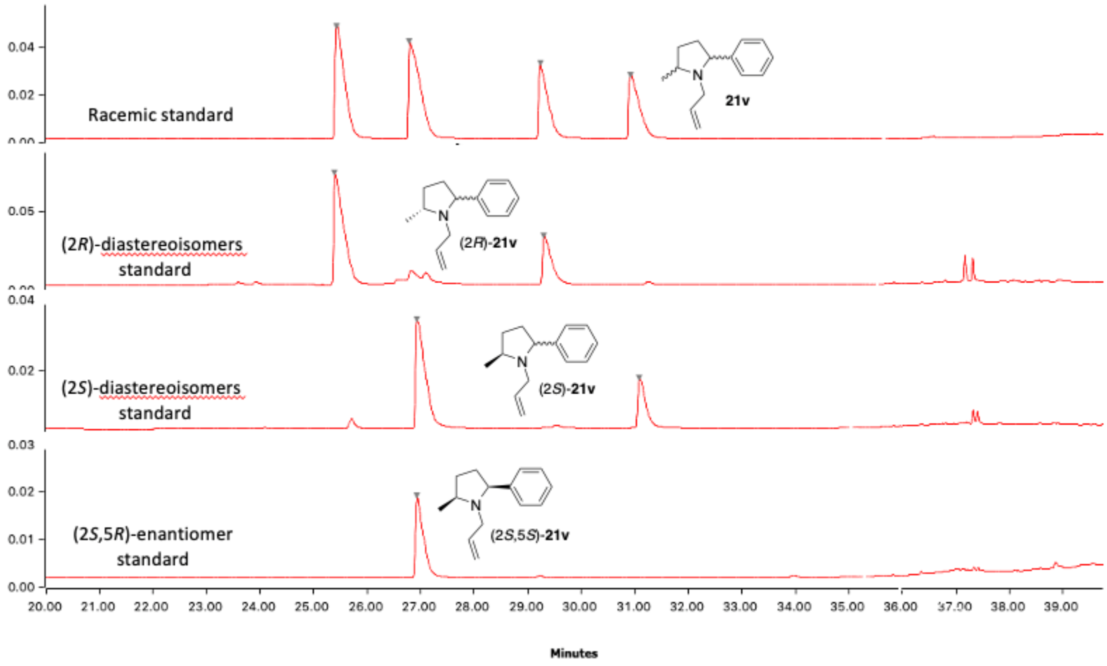

N-propargyl-2-methyl-5-phenylpyrrolidine (22vi) [B]

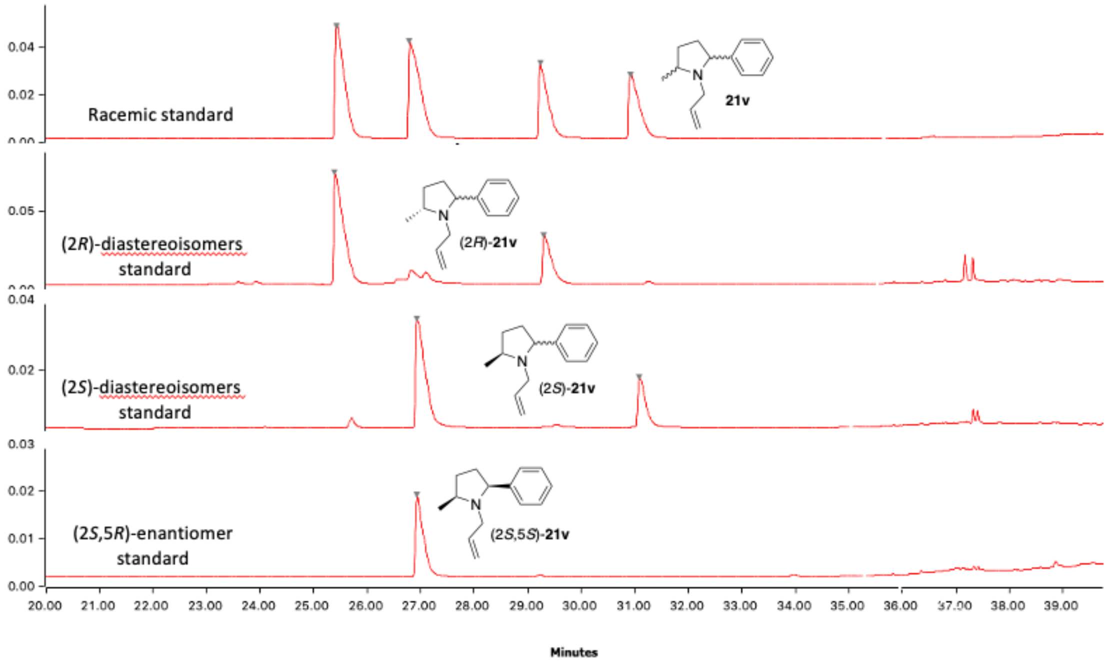

HPLC Chromatograms

A CHIRALCEL OD-H (Daicel, Osaka Japan) column (25 mm x 4.6 mm x 5 µm), an injection volume of 10 µL and UV-monitoring at 265 nm was used. All compounds were analysed using a mobile phase of *n*-hexane/diethylamine = 99.9/0.1 at a flow rate of 0.5 mL / min with samples dissolved in pure hexane, with the exception of nicotine where a mobile phase of *n*-hexane/isopropanol/diethylamine = 94.9/5/0.1 was used.

Chromatograms are presented in the order of biotransformation with ammonia borane addition, followed by "deracemised" biotransformations, followed by racemic chemical standard followed by asymmetric chemical standard.

_N_-methyl-2-phenylpiperidine (15iii)

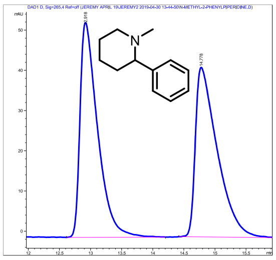

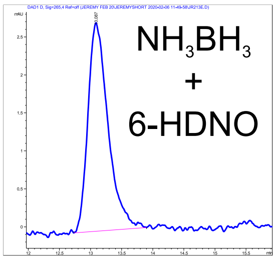

_N_-propyl-2-phenylpiperidine (15iv)

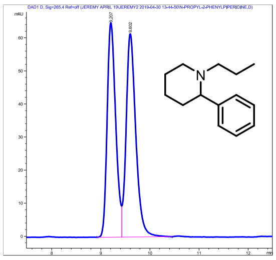

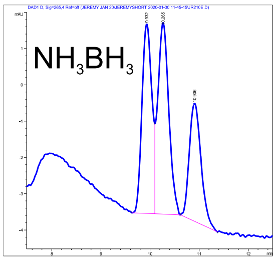

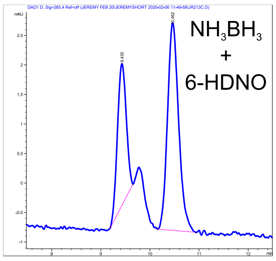

N-allyl-2-phenylpiperidine (15v)

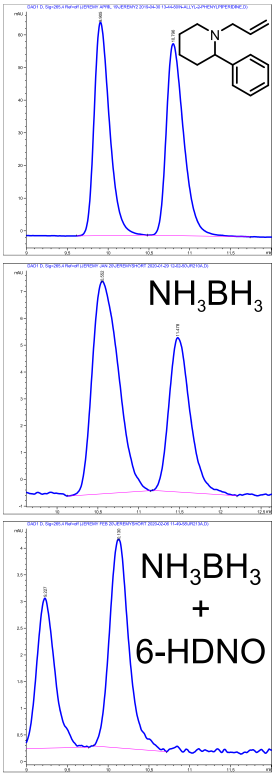

N-propargyl-2-phenylpiperidine (15vi)

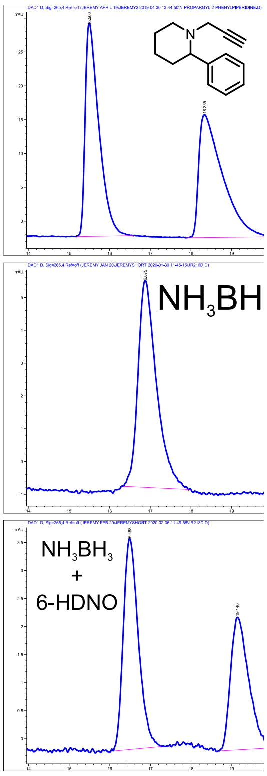

Nicotine (20)

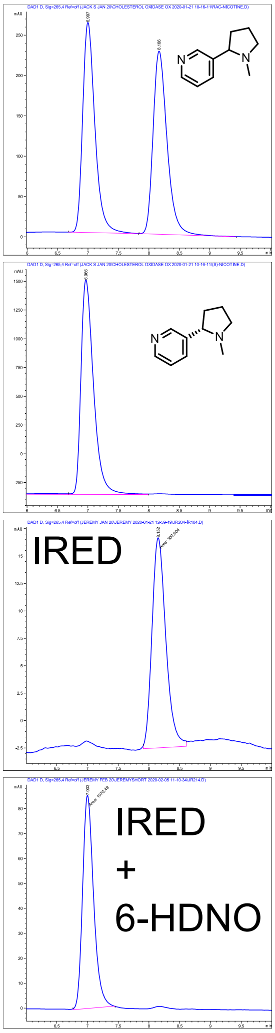

References

(1) Heath, R. S.; Pontini, M.; Bechi, B.; Turner, N. J. Development of an *R*-Selective Amine Oxidase with Broad Substrate Specificity and High Enantioselectivity. *ChemCatChem* **2014**, *6* (4), 996–1002. https://doi.org/10.1002/cctc.201301008.

(2) Hussain, S.; Leipold, F.; Man, H.; Wells, E.; France, S. P.; Mulholland, K. R.; Grogan, G.; Turner, N. J. An (*R*)-Imine Reductase Biocatalyst for the Asymmetric Reduction of Cyclic Imines. *ChemCatChem* **2015**, *7* (4), 579–583. https://doi.org/10.1002/cctc.201402797.

(3) Ramsden, J. I.; Heath, R. S.; Derrington, S. R.; Montgomery, S. L.; Mangas-Sanchez, J.; Mulholland, K. R.; Turner, N. J. Biocatalytic N-Alkylation of Amines Using Either Primary Alcohols or Carboxylic Acids via Reductive Aminase Cascades. *J. Am. Chem. Soc.* **2019**, *141* (3), 1201–1206. https://doi.org/10.1021/jacs.8b11561.
